# Synthesis and herbicidal activities of aryloxyacetic acid derivatives as HPPD inhibitors

**DOI:** 10.3762/bjoc.16.25

**Published:** 2020-02-19

**Authors:** Man-Man Wang, Hao Huang, Lei Shu, Jian-Min Liu, Jian-Qiu Zhang, Yi-Le Yan, Da-Yong Zhang

**Affiliations:** 1 School of Science, China Pharmaceutical University, Nanjing 211198, P. R. China

**Keywords:** aryloxyacetic acid, herbicidal activity, 4-hydroxyphenylpyruvate dioxygenase, modification, synthesis

## Abstract

A series of aryloxyacetic acid derivatives were designed and synthesized as 4-hydoxyphenylpyruvate dioxygenase (HPPD) inhibitors. Preliminary bioassay results reveal that these derivatives are promising *Arabidopsis thaliana* HPPD (*At*HPPD) inhibitors, in particular compounds **I12** (*K*_i_ = 0.011 µM) and **I23** (*K*_i_ = 0.012 µM), which exhibit similar activities to that of mesotrione, a commercial HPPD herbicide (*K*_i_ = 0.013 µM). Furthermore, the newly synthesized compounds show significant greenhouse herbicidal activities against tested weeds at dosages of 150 g ai/ha. In particular, **II4** exhibited high herbicidal activity for pre-emergence treatment that was slightly better than that of mesotrione. In addition, compound **II4** was safe for weed control in maize fields at a rate of 150 g ai/ha, and was identified as the most potent candidate for a novel HPPD inhibitor herbicide. The compounds described herein may provide useful guidance for the design of new HPPD inhibiting herbicides and their modification.

## Introduction

**Scheme 1:**
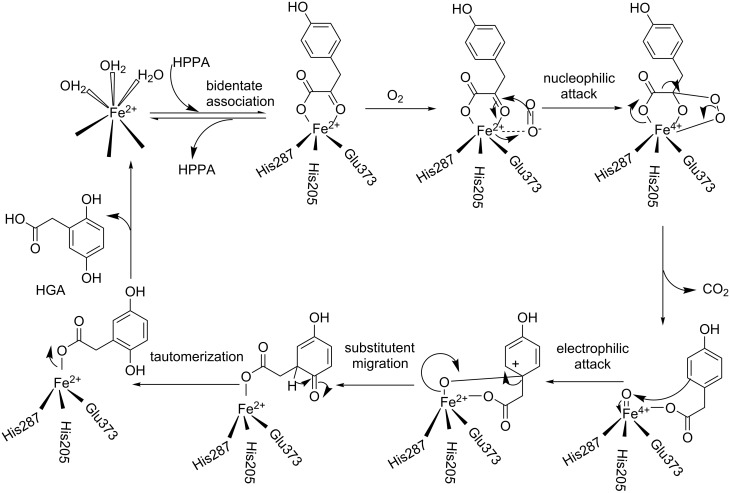
The commonly recognized HPPD catalytic reaction mechanism.

4-Hydroxyphenylpyruvate dioxygenase (EC 1.13.11.27, HPPD), which belongs to the family of non-heme Fe^II^-containing enzymes, is a vital enzyme for tyrosine catabolism. This enzyme is found in microbes, mammals, and plants, and has different functions in different organisms [1]. In the catalytic process of HPPD, 4-hydroxyphenylpyruvic acid (HPPA) and Fe^II^ form a chelate complex, from which the HPPA substrate is converted into homogentisic acid (HGA). The generally accepted catalytic mechanism for this process is shown in Scheme 1 [2–6]. The HPPD amino acid sequence homologies in plants and mammals are significantly different [7–8], and this difference affects the binding stability between an inhibitor and HPPD, leading to inhibitor activities that differ among various species and genera and providing a theoretical basis for the design of inhibitors that are highly selective and safe [2].

In plants, HPPD inhibitors competitively restrain HPPA from chelating to Fe^II^. The production of plastoquinone is inhibited and phytoene is accumulated when the transformation of HPPA to HGA is interfered with an HPPD inhibitor [9–10]; consequently, plants become severely damage when exposed to sunlight, ultimately resulting in bleaching symptoms followed by necrosis and death [11–12]. Therefore, HPPD inhibitors play important roles in the herbicide industry. In addition, HPPD inhibiting herbicides are advantageous because of their low toxicities, high efficiencies, broad-spectrum weed control, and safety toward crops and the environment [13–15]. However, the abuse of HPPD inhibitors has led to increased weed resistance and crop damage. Furthermore, the long-term applications of a single herbicide result in the resistance of the weed to the agent [14]. Therefore, exploring effective HPPD-inhibiting compounds for the control of resistant weeds is an emergent and important objective [2].

**Figure 1:**
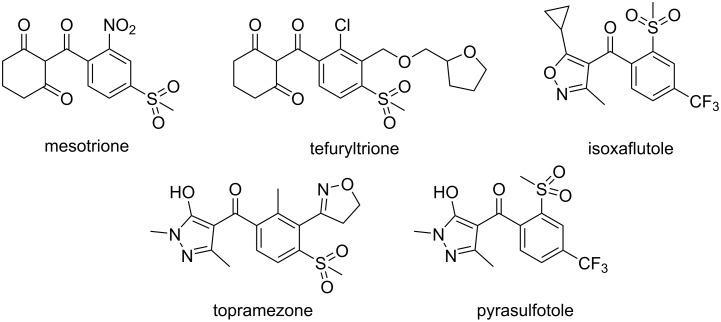
Chemical structures of the commercial HPPD inhibitors.

A considerable number of HPPD inhibiting herbicides have recently been commercialized and applied in the agrochemical industry. These herbicides are mainly divided into three categories: triketones, pyrazoles, and isoxazoles [9,15–16]. Figure 1 shows some HPPD-inhibiting herbicides, namely mesotrione, tefuryltrione, isoxaflutole, topramezone, and pyrasulfotole. Among them, mesotrione is a highly successful representative triketone HPPD herbicide. Figure 1 reveals that the HPPD inhibiting herbicides mostly contain 1,3-dicarbonyl or analogous structures [11,15]. *Arabidopsis thaliana* HPPD (*At*HPPD) and its inhibitors have been reported to interact in two ways: 1) through 1,3-dicarbonyl bidentate chelation with the active center metal, and 2) through favorable sandwich π–π stacking interactions between aromatic rings and the Phe360, Phe403 residues of the active site. Thus, 1,3-dicarbonyl and aromatic moieties are indispensable pharmacophores for potent HPPD-inhibiting compounds that interact with surrounding residues in *At*HPPD [16–19].

2,4-Dichlorophenoxyacetic acid (2,4-D), which acts as a plant growth hormone, was synthesized in 1941. It is a selective pre- and post-emergence herbicide that has applied to several crops [17]. 2,4-D interferes with the hormone balance of the plant, which interrupts nucleic-acid and protein metabolism, and is especially effective in broadleaf weeds, such as *Amaranthus retroflexus* and *Alfalfa*. The application of 2,4-D causes excessive growth that ultimately results in plant death. Consequently, 2,4-D has become one of the world’s major herbicides because low dosage is used and less investment costs are required.

**Figure 2:**
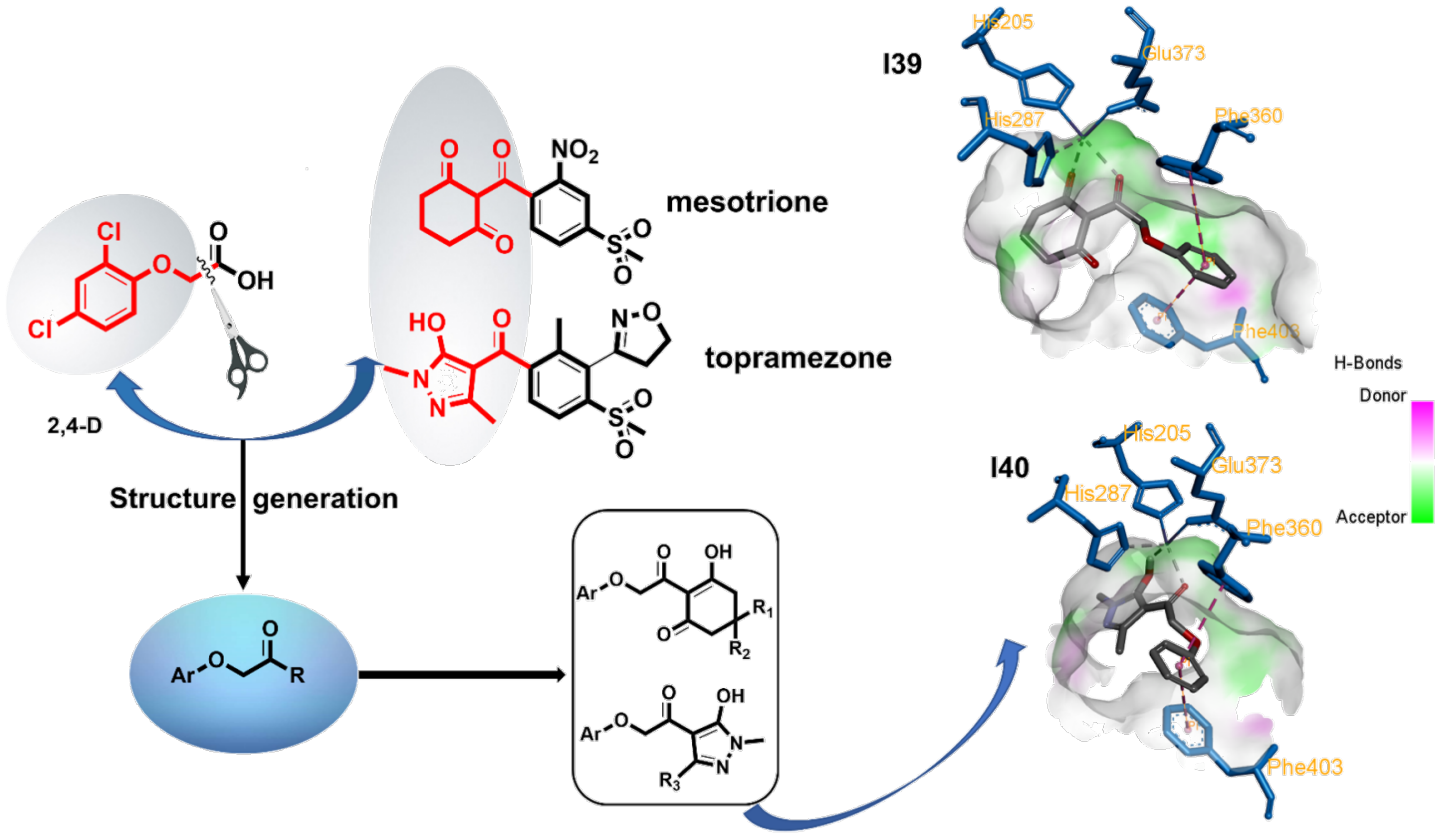
The design strategy of aryloxyacetic acid derivatives as HPPD inhibitors and simulate the binding modes of compound **I39** and **I40** in a target enzyme (*At*HPPD). The key residues in the active site are shown in blue sticks, the Fe^II^ is shown as a dark blue sphere, and compound **I39** and **I40** is shown in gray sticks.

Many researches in HPPD inhibitors have revealed that modifying of aromatic moieties is an eﬀective way of producing new HPPD inhibiting herbicides [20–23]. However, little eﬀort has been directed toward modifying pyrazole derivatives and the carbon−carbon bond between 1,3-dicarbonyl and aroyl moieties. Previously, a series of 2-(aryloxyacetyl)cyclohexane-1,3-diones was synthesized by Wang et al. [24]. We have been interested in inserting a carbon−oxygen bond between the triketone and aroyl moieties of HPPD inhibitors. Initially, molecular docking studies were performed on two representative compounds, namely **I39** and **I40** [25], in order to explore their binding modes. The result revealed the presence of two main interactions, the sandwich π−π interaction and the bidentate interaction, which are similar to those of commercial mesotrione. Inspired by the above revelations, we synthesized a group of new HPPD inhibitors that contain pyrazole and triketone moieties to study their bioactivities; the design strategy is shown in Figure 2. By combining the two bioactive structures, namely the aromatic moieties of 2,4-D and the 1,3-dicarbonyl unit, we designed and synthesized a series of novel aryloxyacetic acid derivatives. In this context, these derivatives were subjected to HPPD inhibition, herbicidal activity, crop safety and structure–activity relationship (SAR) studies. As expected, many of the title compounds displayed promising inhibitory activity against *Arabidopsis thaliana* HPPD (*At*HPPD) in vitro and excellent herbicidal activities at a rate of 150 g ai/ha.

## Results and Discussion

### Chemistry

**Scheme 2:**
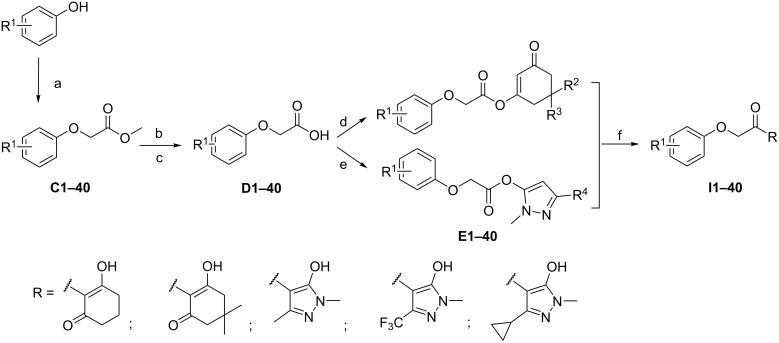
Synthetic route of the title compounds **I**. Reagents and conditions: (a) methyl chloroacetate, K_2_CO_3_, CH_3_CN, 65 °C; (b) K_2_CO_3_, H_2_O, 65 °C; (c) aqueous HCl solution (10%), rt; (d) substituted 1,3-cyclohexanediones, EDCI, DMAP, DCM, rt; (e) substituted 1,3-dimethyl-1*H*-pyrazol-5-ol, EDCI, DMAP, DCM, rt; (f) Et_3_N, acetone cyanohydrin, DCM, rt.

**Scheme 3:**
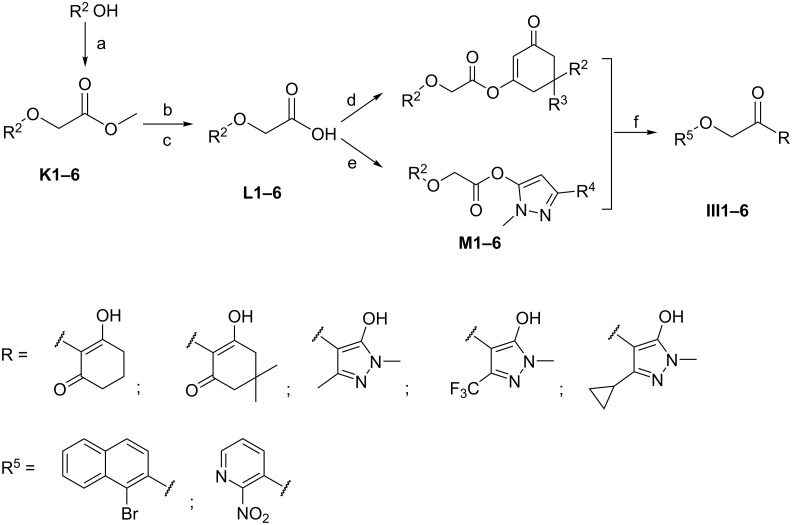
Synthetic route of the title compound **III**. Reagents and conditions: (a) methyl chloroacetate, K_2_CO_3_, CH_3_CN, 65 °C; (b) K_2_CO_3_, H_2_O, 65 °C; (c) aqueous HCl solution (10%), rt; (d) substituted 1,3-cyclohexanediones, EDCI, DMAP, DCM, rt; (e) substituted 1,3-dimethyl-1*H*-pyrazol-5-ol, EDCI, DMAP, DCM, rt; (f) Et_3_N, acetone cyanohydrin, DCM, rt.

**Scheme 4:**
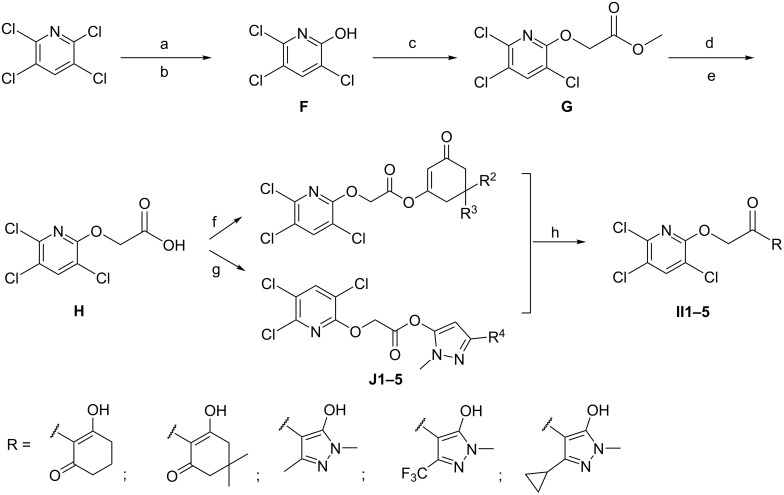
Synthetic route of the title compounds **II**. Reagents and conditions: (a) NaOH, TBAB, H_2_O, 100 °C; (b) concentrated HCl solution, rt; (c) methyl chloroacetate, K_2_CO_3_, CH_3_CN, 65 °C; (d) K_2_CO_3_, H_2_O, 65 °C; (e) aqueous HCl solution (10%), rt; (f) substituted 1,3-cyclohexanediones, EDCI, DMAP, DCM, rt; (g) substituted 1,3-dimethyl-1H-pyrazol-5-ol, EDCI, DMAP, DCM, rt; (h) Et_3_N, acetone cyanohydrin, DCM, rt.

Title compounds were classified into three series (I, II and III). The preparation of the title compounds is shown in Scheme 2, Scheme 3 and Scheme 4. The synthesis of compounds **I** and **III** was depicted in Scheme 2 and Scheme 3. The commercially available starting materials reacted with methyl chloroacetate in CH_3_CN and anhydrous potassium carbonate (K_2_CO_3_) as the base, and the corresponding products **C** and **K** were prepared. The products were hydrolyzed using K_2_CO_3_ as a base to yield the product **D** and **L** [26–30]. In the presence of 3-(ethyliminomethylideneamino)-*N*,*N*-dimethylpropane-1-amine, hydrochloride (EDCI), the aromatic oxyacetic acid reacted with substituted 1,3-cyclohexanediones or substituted 1,3-dimethyl-1*H*-pyrazole-5-ol, using DMAP as the catalyst. Subsequently, the key enol ester **E** and **M** were respectively obtained. Finally, Fries-type rearrangements were performed in anhydrous DCM at room temperature to afford the title compounds **I** and **III** [31].

As shown in Scheme 4, the title compounds **II** were obtained by a five-step synthetic route using the commercially available 2,3,5,6-tetrachloropyridine as the starting material. In the presence of TBAB, the starting material was hydrolyzed using NaOH in water at 100 °C. The resulting solution was cooled and hydrolyzed with HCl solution that yielded compound **F**. Subsequent preparations for compounds **G, H, J** and **II** were respectively the same as for compounds **C**, **D**, **E** and **I**.

**Figure 3:**
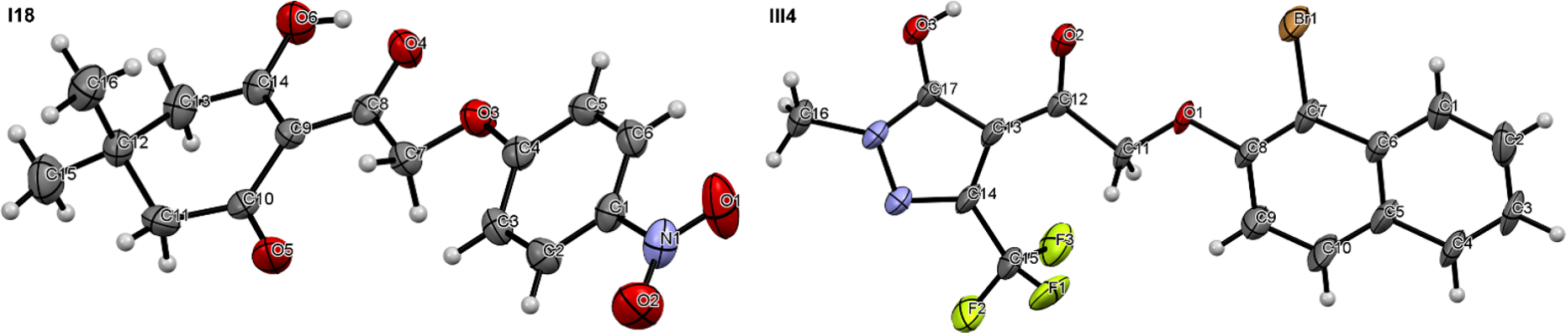
Crystal structures of **I18** and **III4**.

All intermediates were synthesized and characterized as detailed in Supporting Information File 1. The structures of all prepared compounds were conﬁrmed by ^1^H and ^13^C NMR spectroscopy, and HRMS. Furthermore, the structures of compounds **I18** and **III4** were veriﬁed by X-ray diﬀractometry (Figure 3). Crystallographic data for crystalline **I18** and **III4** have been deposited with the Cambridge Crystallographic Data Centre (CCDC 1959130, CCDC 1959152).

### HPPD inhibition

**Figure 4:**
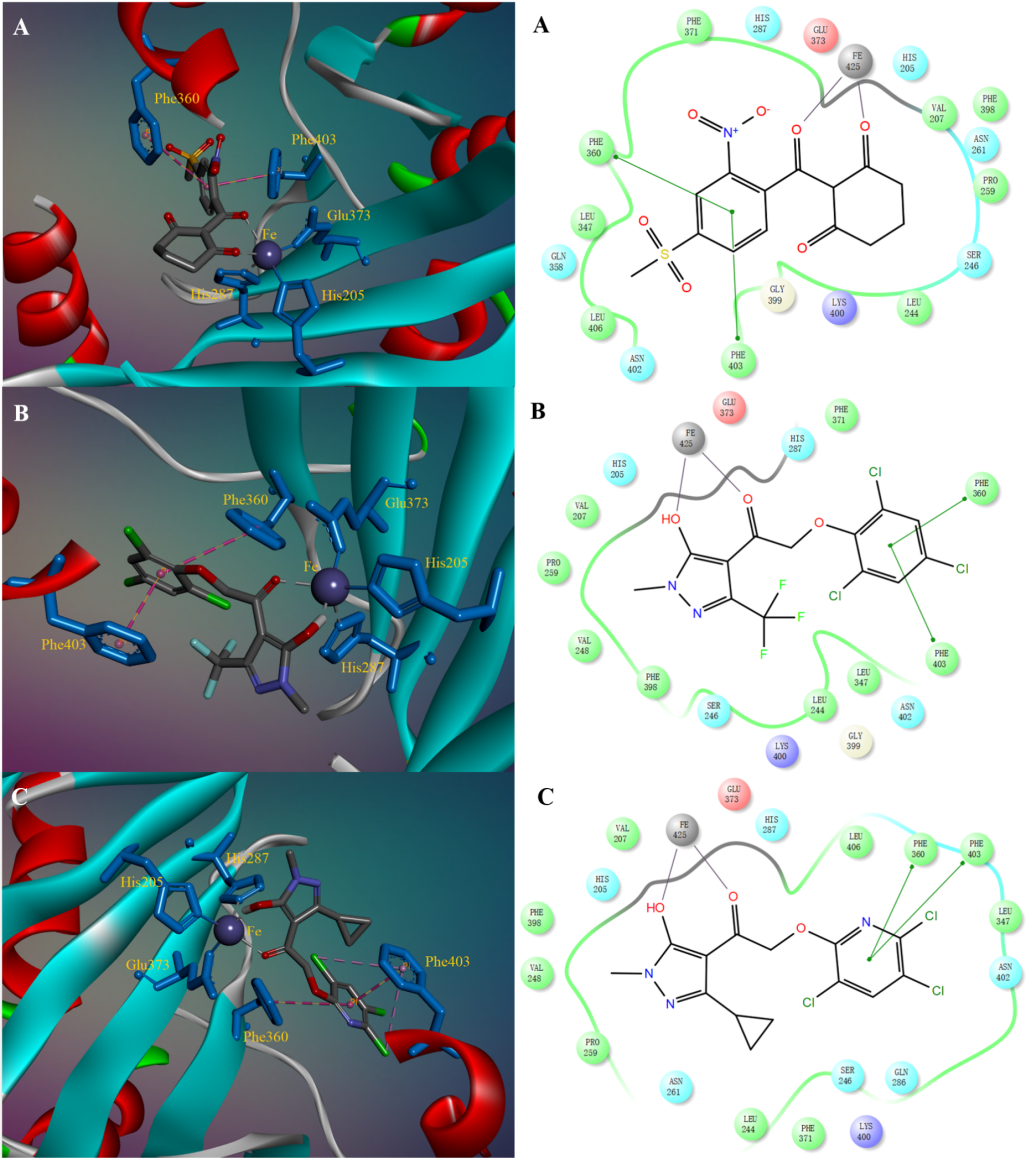
Simulated binding mode of mesotrione (A), compound **I12** (B) and compound **II4** (C) with *At*HPPD. The key residues in the active site are shown in blue sticks, and Fe^II^ is shown as a dark blue sphere. mesotrione, compound **I12,** and **II4** are shown in gray sticks.

The title compounds displayed promising *At*HPPD inhibitory activities, with Table 1 and Table 2 revealing that compounds **I12** (*K*_i_ = 0.011 µM) and **I23** (*K*_i_ = 0.012 µM) exhibit similar inhibitor potencies to that of mesotrione (*K*_i_ = 0.013 µM). Docking studies using the CDOCKER module within Discovery Studio 4.0 revealed the bioactive binding site positions of potential inhibitors within the targets active site. We modeled the interactions of **I12** and **II4** (C) with *At*HPPD (PDB ID: 1TFZ). The structure of *At*HPPD was taken from the PDB data bank. All molecular modeling studies were carried out as previously reported [10,19,32–34]. The results show that two main interactions exist between **I12** and the *At*HPPD active site (Figure 4*),* as was observed for mesotrione; the 1,3-dicarbonyl unit is chelated to the iron ion, and the aromatic ring moiety formed π–π interactions with Phe403 and Phe360.

**Table 1:** Chemical structures of title compound I and their biological activity against *At*HPPD.

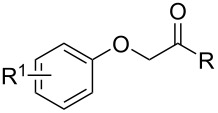 **I1–40**			

compound	R^1^	R	*At*HPPD inhibition *K*_i_ (μM)

**I1**	H	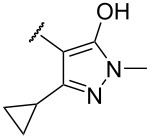	1.5 ± 0.031
**I2**	H	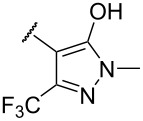	1.3 ± 0.017
**I3**	2-chloro	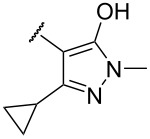	0.36 ± 0.012
**I4**	2-chloro	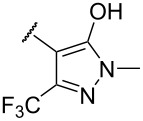	0.59 ± 0.043
**I5**	4-chloro	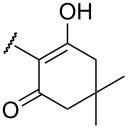	1.0 ± 0.036
**I6**	4-chloro	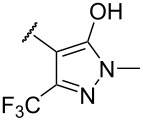	0.93 ± 0.032
**I7**	2,4-dichloro	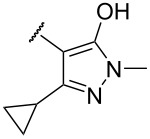	0.36 ± 0.012
**I8**	2,4-dichloro	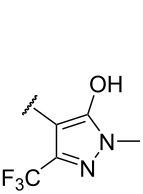	0.22 ± 0.023
**I9**	2,4,6-trichloro	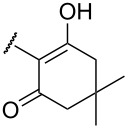	0.31 ± 0.048
**I10**	2,4,6-trichloro	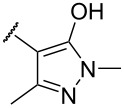	0.24 ± 0.003
**I11**	2,4,6-trichloro	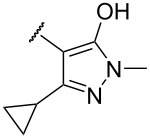	0.081 ± 0.001
**I12**	2,4,6-trichloro	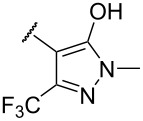	0.011 ± 0.012
**I13**	2-nitro	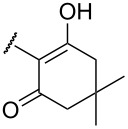	0.45 ± 0.033
**I14**	2-nitro	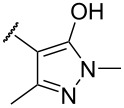	0.21 ± 0.042
**I15**	2-nitro	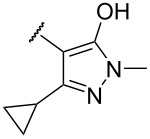	0.27 ± 0.004
**I16**	2-nitro	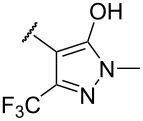	0.44 ± 0.013
**I17**	4-nitro	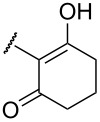	0.23 ± 0.004
**I18**	4-nitro	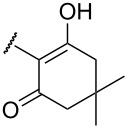	0.93 ± 0.006
**I19**	4-nitro	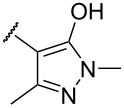	0.63 ± 0.002
**I20**	4-nitro	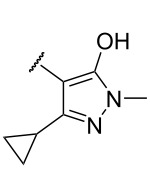	0.50 ± 0.003
**I21**	4-nitro	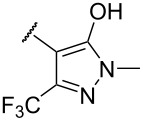	1.5 ± 0.041
**I22**	2-chloro-4-nitro	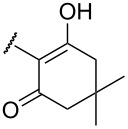	1.02 ± 0.009
**I23**	2-chloro-4-nitro	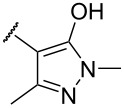	0.012 ± 0.009
**I24**	2-chloro-4-nitro	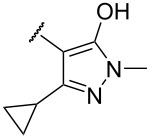	0.26 ± 0.012
**I25**	2-chloro-4-nitro	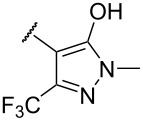	0.21 ± 0.043
**I26**	2-(2,4-dichlorophenoxy)-4-chloro	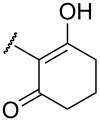	1.9 ± 0.001
**I27**	2-(2,4-dichlorophenoxy)-4-chloro	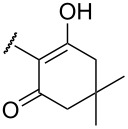	2.2 ± 0.041
**I28**	2-(2,4-dichlorophenoxy)-4-chloro	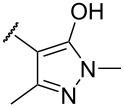	0.032 ± 0.002
**I29**	2-(2,4-dichlorophenoxy)-4-chloro	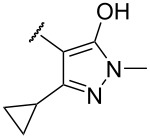	1.3 ± 0.022
**I30**	2,4-dimethyl	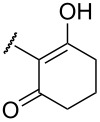	2.2 ± 0.034
**I31**	2,4-dimethyl	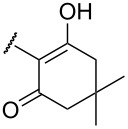	3.1 ± 0.34
**I32**	2,4-dmethyl	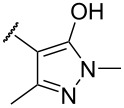	2.0 ± 0.009
**I33**	2,4-dimethyl	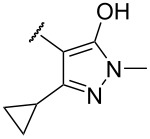	2.8 ± 0.045
**I34**	2,4-dimethyl	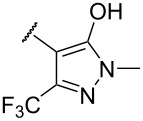	2.8 ± 0.67
**I35**	4-methyl-5-methoxy	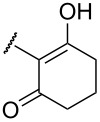	3.3 ± 0.14
**I36**	4-methyl-5-methoxy	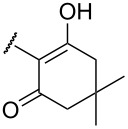	3.4 ± 0.21
**I37**	4-methyl-5-methoxy	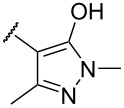	2.7 ± 0.53
**I38**	4-methyl-5-methoxy	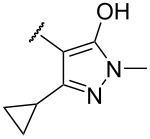	2.9 ± 0.038
**I39**	H	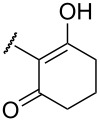	1.3 ± 0.056
**I40**	H	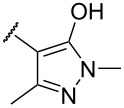	1.2 ± 0.031
mesotrione			0.013 ± 0.001

**Table 2:** Chemical structures of title compound **II**, **III** and their biological activity against *At*HPPD.

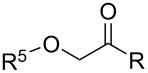 **II1–5, III1–6**			

compound	R^5^	R	*At*HPPD inhibition *K*_i_ (μM)

**II1**	2,3,5-trichloro-6-pyridyl	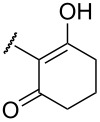	0.093 ± 0.007
**II2**	2,3,5-trichloro-6-pyridyl	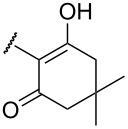	0.097 ± 0.010
**II3**	2,3,5-trichloro-6-pyridyl	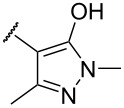	0.021 ± 0.004
**II4**	2,3,5-trichloro-6-pyridyl	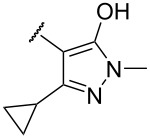	0.023 ± 0.006
**II5**	2,3,5-trichloro-6-pyridyl	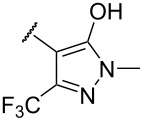	0.12 ± 0.003
**III1**	2-bromo-2-naphthyl	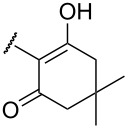	2.50 ± 0.011
**III2**	2-bromo-2-naphthyl	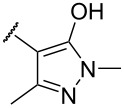	2.2 ± 0.090
**III3**	2-bromo-2-naphthyl	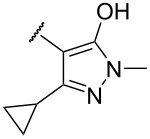	2.2 ± 0.13
**III4**	2-bromo-2-naphthyl	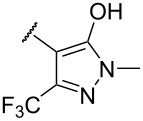	2.0 ± 0.012
**III5**	2-nitro-3-pyridyl	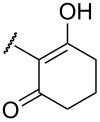	0.23 ± 0.011
**III6**	2-nitro-3-pyridyl	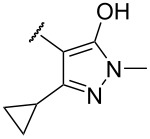	0.21 ± 0.042
mesotrione			0.013 ± 0.001

Electron-withdrawing and electron-donating groups were introduced onto the benzene ring of **I1**, which significantly inﬂuenced the HPPD inhibition activity. We found that electron-withdrawing groups improve the activity; for example, **I3** (*K*_i_ = 0.36 µM) and **I4** (*K*_i_ = 0.59 µM) were more potent than **I1** (*K*_i_ = 1.5 µM) and **I2** (*K*_i_ = 1.3 µM). In addition, the position of the electron-withdrawing group played an essential role in determining the HPPD inhibitory activity. In most cases, compounds with a chlorine atom at the 2-position (**I4**) were more active than those with the chlorine at the 4-position (**I6**), clearly an electron-withdrawing group at the 2-position provides enhanced activity compared to the 4-position. In addition, electron-donating groups were found to be detrimental to HPPD inhibition activity (**I1** > **I33**, **I38**). We observed that methyl groups at the 5-position of the 1,3-cyclohexane ring were unfavorable to activity (**I17** > **I18**, **I26** > **I27**, **II1** > **II2**), and that the introduction a nitro group led to more potent activity compared to that generated by a chlorine atom (except for **I6** and **I21**), such that **I15** > **I3**, **I18** > **I5**, and **I24** > **I7** in terms of activity. Generally speaking, compounds with a pyrazole ring exhibited better HPPD inhibitory activities than those with cyclohexanedione rings (**I28**, **I29** > **I26**, **I27**; **II3**, **II4** > **II1**, **II2**).

### Herbicidal activity

**Figure 5:**
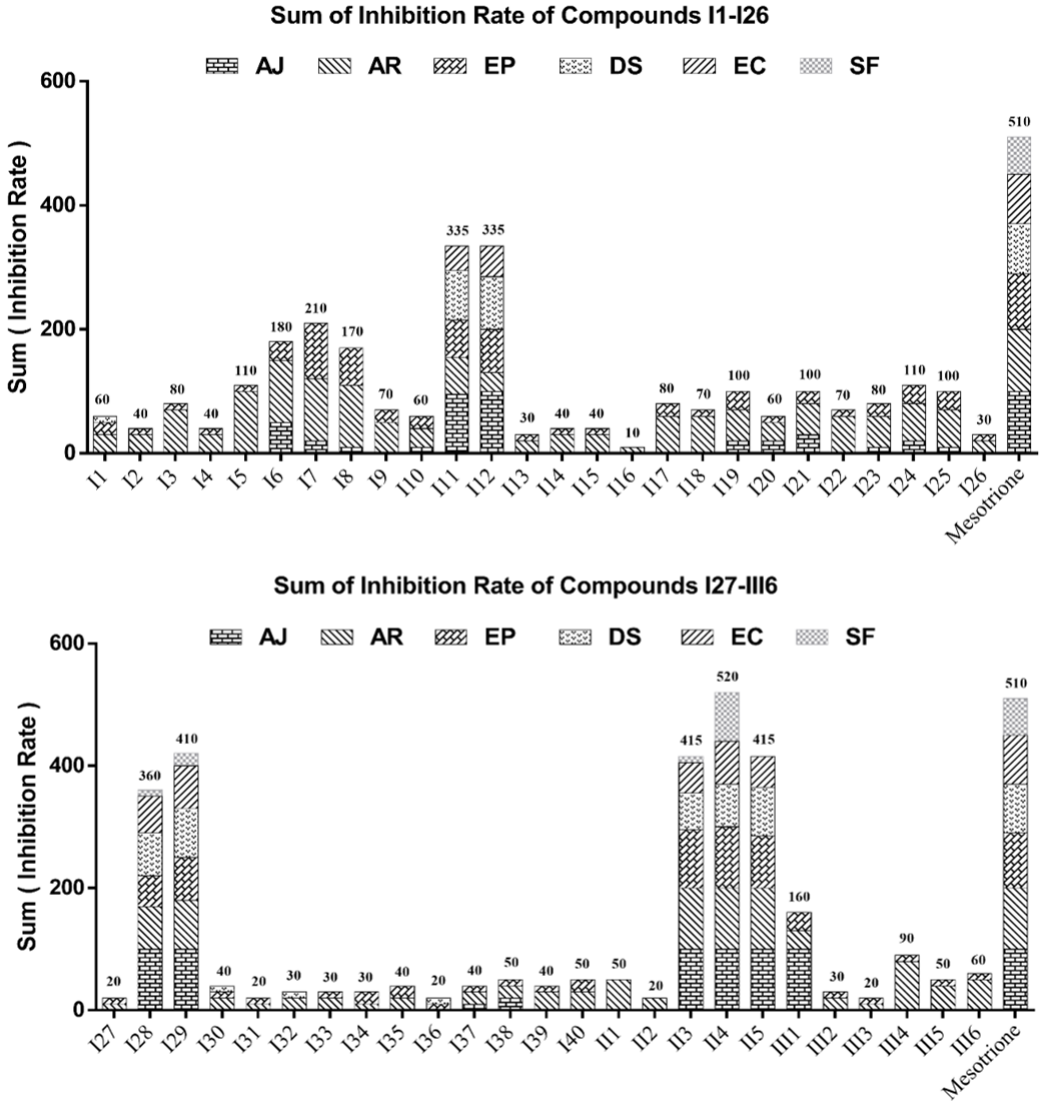
Sum of inhibition rate of title compounds at 150 g ai/ha. (Abbreviations: AJ, *Abutilon juncea*; AR, *Amaranthus retroflexus*; EP, *Eclipta prostrata*; DS, *Digitaria sanguinalis*; EC, *Echinochloa crus-galli*; SF, *Setaria faberii*.)

The post-emergence herbicidal activities of the title compounds are summarized in Figure 5. In our work, these weeds, *E. crus-galli* (EC), *S. faberii* (SF), *D. sanguinalis* (DS), *A. retroflexus* (AR), *E. prostrata* (EP), and *A. juncea* (AJ), were selected for evaluating the post-emergence herbicidal activities of the title compounds.

**Figure 6:**
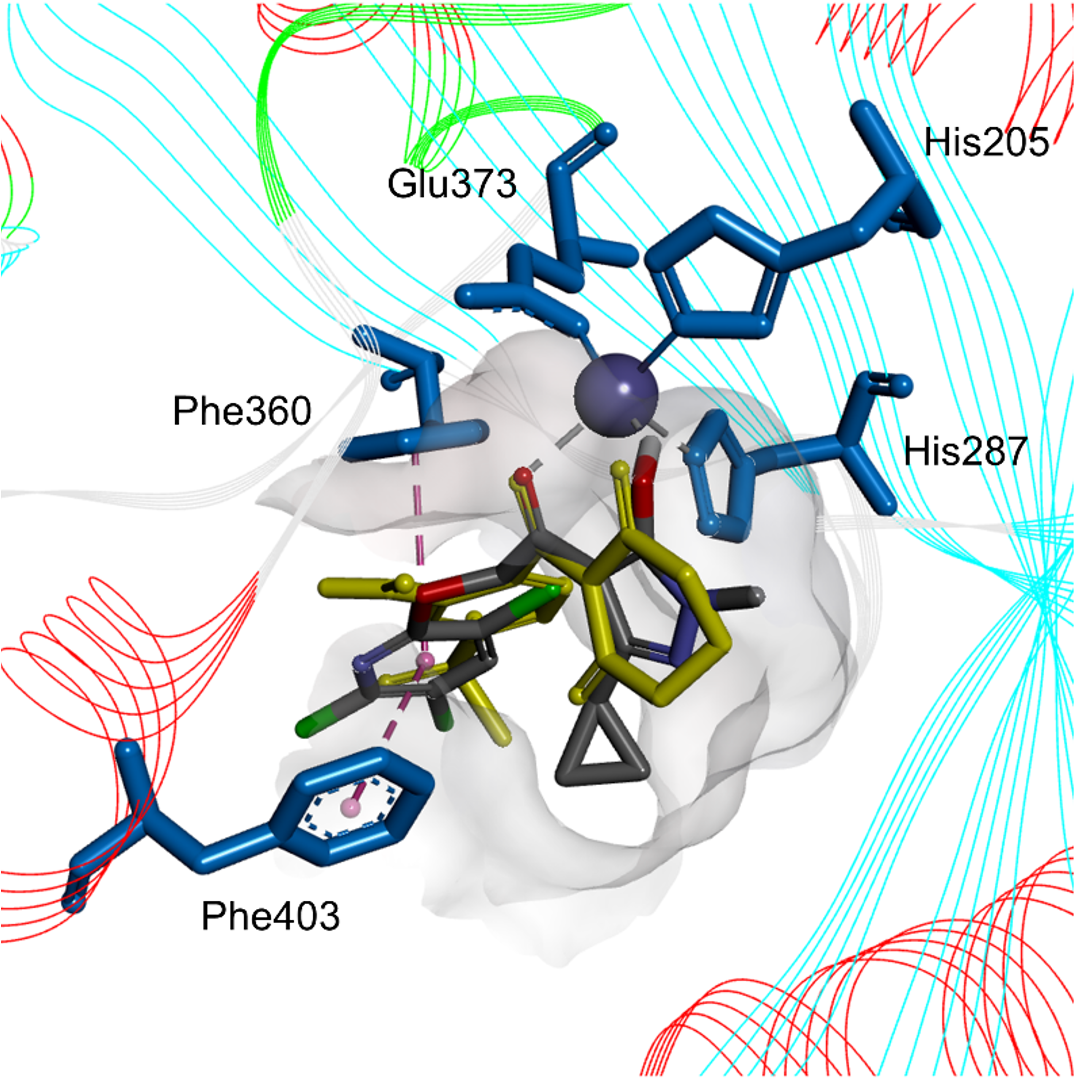
Simulated folding mode of mesotrione (yellow sticks) and compound **II4** (gray sticks) with *At*HPPD. The key residues in the active site are shown in blue sticks, and Fe^II^ is shown as a dark blue sphere.

Some of the synthesized compounds exhibited better control efficiencies for the test weed; among them, compounds **I28**, **I29**, **II3** and **II4** showed broad-spectrum herbicidal activities, with **II4** even showing a slightly higher herbicidal activity than mesotrione at a rate of 150 g ai/ha. When the structure of compound **II4** was superimposed onto that of mesotrione, the positive control drug, we observed that it perfectly fits into the active pocket, as shown in Figure 6.

In this work, two categories of HPPD inhibitors were synthesized, including triketone and pyrazole derivatives. Compared with the triketone derivatives, the pyrazole-containing derivatives were generally more herbicidally potent. For instance, pyrazole-containing compounds **I11** and **I12** displayed enhanced activities relative to compound **I9**, which contains a cyclohexanedione ring. We also observed that the introduction of methyl groups at the 5-position of the 1,3-cyclohexane ring was detrimental to herbicidal activity (**I17** > **I18**, **I26** > **I27**, **II1** > **II2**). Compounds with electron-withdrawing groups on the aromatic ring were found to displayed higher herbicidal activities than those with electron-donating groups (i.e., **I7** > **I33**, **I8** > **I34**), which is consistent with the observed *At*HPPD inhibitory activity, and **I28** and **I29** had significantly superior herbicidal activities. Thus, the introduction of large groups on the benzene ring appears to be beneficial to the activity and deserves further structural optimization.

The herbicidal activities of compounds containing other aromatic rings, compound **II** and **III** bearing a pyridine ring and a naphthalene ring, were examined. The results show that the chloro-substituted pyridine exhibited superior herbicidal activity, which provides a theoretical basis for the further development of highly effective HPPD herbicides. Some compounds with significant *At*HPPD inhibitory activities were found not to exhibit promising herbicidal activities. For example, compound **I12**, with the best *At*HPPD inhibition activity (*K*_i_ = 0.011 µM) exhibited poorer than expected herbicidal activity, which is possibly related to its stability and metabolism in the plant [2].

### Crop safety

Crop safety is one of the main considerations during herbicide discovery. Compound **II3**, **II4**, and **II5**, which exhibited excellent herbicidal activities, were chosen for further crop safety studies and to evaluate whether or not they have the potential to be developed as herbicides (Table 3). Commercial mesotrione was selected as the positive control HPPD herbicide. We found that wheat and maize showed high tolerance to compound **II3** at a dosage of 150 g ai/ha, however, its herbicidal activity could not compete with that of mesotrione. In addition, maize displayed tolerance to compound **II4**, indicating that **II4** had the potential to be developed as a postemergence herbicide for weed control in maize fields.

**Table 3:** Postemergence crop safety of compounds **II3**, **II4** and **II5** (150 g ai/ha).

compound	dosage(g ai/ha)	% injury					

		rice	wheat	maize	cotton	soybean	canola

**II3**	150	40	10	10	60	50	90
**II4**	150	30	50	10	60	30	100
**II5**	150	50	50	30	90	70	100
mesotrione	150	50	40	10	80	50	100

## Conclusion

A series of aryloxyacetic acid derivates was synthesized as novel HPPD inhibitors. The bioassay studies revealed that some of the title compounds, such as compound **I12** (*K*_i_ = 0.011 µM), **I23** (*K*_i_ = 0.012 µM), showed similar *At*HPPD inhibitor potencies to that of mesotrione (*K*_i_ = 0.013 µM). Moreover, several newly synthesized compounds displayed strong, broad spectrum weed control when dosed at 150 g ai/ha. Most importantly, compound **II4,** with good HPPD inhibition activity (*K*_i_ = 0.023 µM**),** exhibited a slightly higher herbicidal activity than mesotrione. In addition, **II4** was found to be safe for use on maize. These results suggest that compound **II4** is a promising HPPD inhibiting herbicide candidate deserving of further optimization studies.

## Experimental

The experimental details and analytical data for intermediates **C** to **M** and title compounds were given in Supporting Information File 1. The chemical structures of all title compounds were conﬁrmed by ^1^H and ^13^C NMR spectroscopic analyses and HRMS spectrometric analyses.

### X-ray diffraction

Single crystals of compounds **I18** and **III4** were cultivated for structure validation. Compound **I18** was recrystallized from a mixture of DCM/methanol to afford a colorless transparent crystal. It crystallized in the monoclinic space group: P–1 (2), cell: *a* = 6.425(4) Å, *b* = 9.854(6) Å, *c* = 13.205(8) Å, α = 93.974(7)°, β = 102.211(7)°, γ = 107.567(7)°, temperature: 298 K. Compound **III4** was recrystallized from a mixture of DCM/methanol to afford a colorless transparent crystal. It crystallized in the monoclinic space group: P–1 (2), cell: *a* = 5.191(5) Å, *b* = 12.133(12) Å, *c* = 13.576(14) Å, α = 80.141(13)°, β = 81.978(12)°, γ = 79.496(12)°, temperature: 296 K. X-ray crystal structure of compound **I18** and **III4** are shown in Figure 3.

Crystallographic data for crystal compounds **I18** and **III4** were deposited with the Cambridge Crystallographic Data Centre as supplementary publications with the deposition numbers CCDC 1959130 and CCDC 1959152, respectively. The data can be obtained free of charge from http://www.ccdc.cam.ac.uk/.

### Docking study

The docking study was conducted with the method reported previously [10,19,32–34]. Crystal structures of *Arabidopsis thaliana* HPPD (PDB ID: 1TFZ) with the native ligand, named DAS869 were downloaded from the Protein Data Bank. The docking was carried out using Discovery Studio 4.0. During the docking process, all water molecules were removed. The ligand and protein were prepared with the Dock Ligands tool before docking. By using Define and Edit Binding Site tool to identify the active site. Then the center of the native ligand was deleted. Utilizing the CDOCKER, the prepared ligand was docked into the protein receptor binding site. After the docking calculations were performed, the best binding modes were determined by docking scores and also compared with the simulated binding mode of mesotrione with *At*HPPD.

### Enzyme inhibition study

*At*HPPD was prepared and puriﬁed according to the reported methods in the literature [19–20,35–37]. The inhibition constant (*K*_i_) was obtained and shown in Table 1 and Table 2.

### Herbicidal activities

The post-emergence herbicidal activities of the title compounds were evaluated against monocotyledon weeds (*E. crus-galli*, *S. faberii*, and *D. sanguinalis*) and broadleaf weeds (*A. retroflexus*, *E. prostrata*, and *A. juncea*) in the greenhouse experiments. The commercial HPPD herbicide mesotrione was regarded as a control. All tested compounds were dissolved in DMF as 100 g/L emulsiﬁed concentrates, containing 1% Tween-80 as emulsiﬁer. Then the solvent was diluted with distilled water. Flowerpots with an inner diameter of 7.5 cm were ﬁlled with complex nutrient soil to three-fourths of their height. The above six weed targets were respectively grown in the pots and covered with soil to a thickness of 0.2 cm and grown in the greenhouse. When the weeds grew to about the three-leaf stage, they were treated by the title compounds at the rate of 150 g ai/ha. After 18 days of treatment with inhibitors, the herbicidal activities were surveyed and evaluated with two duplicates per experiment [19]. (Figure 5)

### Crop selectivity

The representative crops, rice, wheat, maize, cotton, soybean, and canola were selected to test the crop safety of compound **II3, II4,** and **II5**. The six crops were separately planted in ﬂowerpots (12 cm diameter) containing the composite nutrient soil and grown at room temperature. When the crops had reached the four-leaf stage, the safety experiments were conducted at the rate of 150 g ai/ha. After 15 days, the ﬁnal results of crop safety were evaluated with two duplicates per experiment (Table 3).

## Supporting Information

File 1 Additional experimental and analytical data, and NMR spectra of synthesized compounds.
