# Supplementary material for: Synthesis and herbicidal activities of aryloxyacetic acid derivatives as HPPD inhibitors
Source: Beilstein J Org Chem. 2020 Feb 19;16:233–47. doi: 10.3762/bjoc.16.25 (PMC7059547; doi:10.3762/bjoc.16.25)
Supplement: File 1 — Additional experimental and analytical data, and NMR spectra of synthesized compounds. [file Beilstein_J_Org_Chem-16-233-s001.pdf]

BEILSTEIN JOURNAL OF ORGANIC CHEMISTRY

Supporting Information

for

Synthesis and herbicidal activities of aryloxyacetic acid derivatives as HPPD inhibitors

Man-Man Wang, Hao Huang, Lei Shu, Jian-Min Liu, Jian-Qiu Zhang, Yi-Le Yan and Da-Yong Zhang

Beilstein J. Org. Chem. **2020,** *16,* 233–247. doi:10.3762/bjoc.16.25

Additional experimental and analytical data, and NMR spectra of synthesized compounds

License and Terms: This is a supporting information file under the terms of the Creative Commons Attribution License (https://creativecommons.org/licenses/by/4.0). Please note that the reuse, redistribution and reproduction in particular requires that the authors and source are credited.
The license is subject to the *Beilstein Journal of Organic Chemistry* terms and conditions: (https://www.beilstein-journals.org/bjoc)

Contents

1. **General information……………………………………………….S2**
2. **General procedure for intermediate……………………………S3–4**
3. **^{1}H NMR data for compounds C to M………………………… S5–55**
4. **^{1}H NMR, ^{13}C NMR and HRMS spectrometric data for title compounds………………………………………………………… S56–129**
5. **References……………………………………………………….S130–131**

General information

^{1}H NMR and ^{13}C NMR spectra were recorded with Bruker ARX-300 spectrometer with TMS as the internal standard in $CDCl_3$, DMSO-d_6. Peak multiplicities are abbreviated: singlet, s; doublet, d; triplet, t; multiplet, m. MS spectra were obtained on an Agilent 6120 quadrupole LC/MS (ESI). HR-ESI-MS was run on an Agilent Q-TOF mass spectrometer. The solvents used were all AR grade and were redistilled under a positive pressure of dry nitrogen atmosphere in the presence of proper desiccant when necessary. All commercially available reagents were used without further purification. Air- and/or moisture-sensitive reactions were carried out under nitrogen atmosphere. Silica gel (200–300 mesh, Qingdao city, China) was used for column chromatography. The progress of the reactions was monitored by analytical thin-layer chromatography (TLC) on HSGF254 precoated silica gel plates. X-ray diffraction analyses were measured on a Siemens P4 diffractometer.

General procedure for intermediate

Title compounds were classified into three series (**I**, **II** and **III**), and prepared by three different synthetic routes, including esterification, hydrolysis, condensation, and rearrangement reactions. The preparation of the title compounds was shown in **Scheme 1**, **Scheme 2** and **Scheme 3**.

General procedure for intermediate C1 – C40

Substituted phenol (0.1 mol), anhydrous CH_3CN (20 mL) and K_2CO_3 (0.11 mol) were added to a 100 mL dry eggplant flask. With stirring, methyl chloroacetate (0.11 mol) was added dropwise 3–6 h at 65 °C. The reaction progress was tracked using TLC. After the reaction completion, the reaction mixture was filtered and the filtrate was evaporated to give the compound **C**. The analytical data corresponded to the literature [1-8].

General procedure for intermediate D1 – D40

Compound **C** (0.1 mol), K_2CO_3 (0.15 mol) and water (50 mL) were added to a 100 mL clean eggplant flask. After the reaction mixture was stirred for 5–10 h at 65 °C, the reaction mixture was cooled to room temperature and was acidified with aqueous HCl solution (10%) to pH = 2–3. A white solid was afforded by filtration. After vacuum drying, product **D** was used in the next step. The analytical data corresponded to the literature [9-14].

General procedure for intermediate E1 – E40

In the presence of DMAP as the catalyst to speed up the reaction, substituted 1,3-cyclohexanediones or substituted 1,3-dimethyl-1H-pyrazol-5-ol (0.1 mol), EDCI (0.1 mol), anhydrous dichloromethane (DCM) (30 mL) and compound D (0.1 mol) were added to a 50 mL eggplant-shaped. The solution was stirred for 5–8 h. The progress of the reaction was monitored by TLC. After completion of the reaction, DCM was removed from the system under reduced pressure. Residues were purified via flash chromatography ($V_{ethylacetate}$: $V_{petroleumether}$ =1:3) to afford the enol ester.

General procedure for intermediate F.

2,3,5,6-Tetrachloropyridine (0.1 mol), NaOH, TBAB and 60mL H_2O were added to a

100 mL eggplant-shaped. The mixture was heated at 100 °C for 8 hours, and monitored by TLC. Upon the reaction completion, the mixture was poured into ice water (100 mL) and was acidified to pH = 5–6. After filtration and extraction, **F** was obtained as white solid. The analytical data corresponded to the literature [15, 16].

General procedure for intermediate G to J

By using the same methods as corresponding intermediates **C** to **E**, the compounds **G** to **J** could be obtained.

General procedure for intermediate K to M

By using the same methods as corresponding intermediates **C** to **E**, the compounds **K** to **M** could be prepared. The analytical data corresponded to the literature[17-20].

General procedure for title compounds

Title compounds were synthesized as previously reported. [21] The important intermediate enol ester (10 mmol), Et_3N (10 mmol), anhydrous dichloromethane (DCM) (30 mL) and acetone cyanohydrin were added to a 50 mL eggplant-shaped, stirred for 24 h at room temperature, and the reaction progress was tracked using TLC. After completion of the reaction, the mixture was concentrated under vacuum, subsequently acidified to pH 2–3 with 10% hydrochloric acid, filtered and the residue was recrystallization ($V_{dichloromethane}$: $V_{petroleumether}$ =1:2-2:1) to afford title compounds.

^{1}H NMR data for compounds C to M

Data and spectra for compounds C1–C40

methyl 2-phenoxyacetate (C1, C2):

Yield, 90%; white solid; ^{1}H NMR (300 MHz, $CDCl_3$) δ 7.32 (t, J = 8.0 Hz, 2H), 7.02 (t, J = 7.4 Hz, 1H), 6.94 (d, J = 8.0 Hz, 2H), 4.65 (s, 2H), 3.81 (s, 3H).

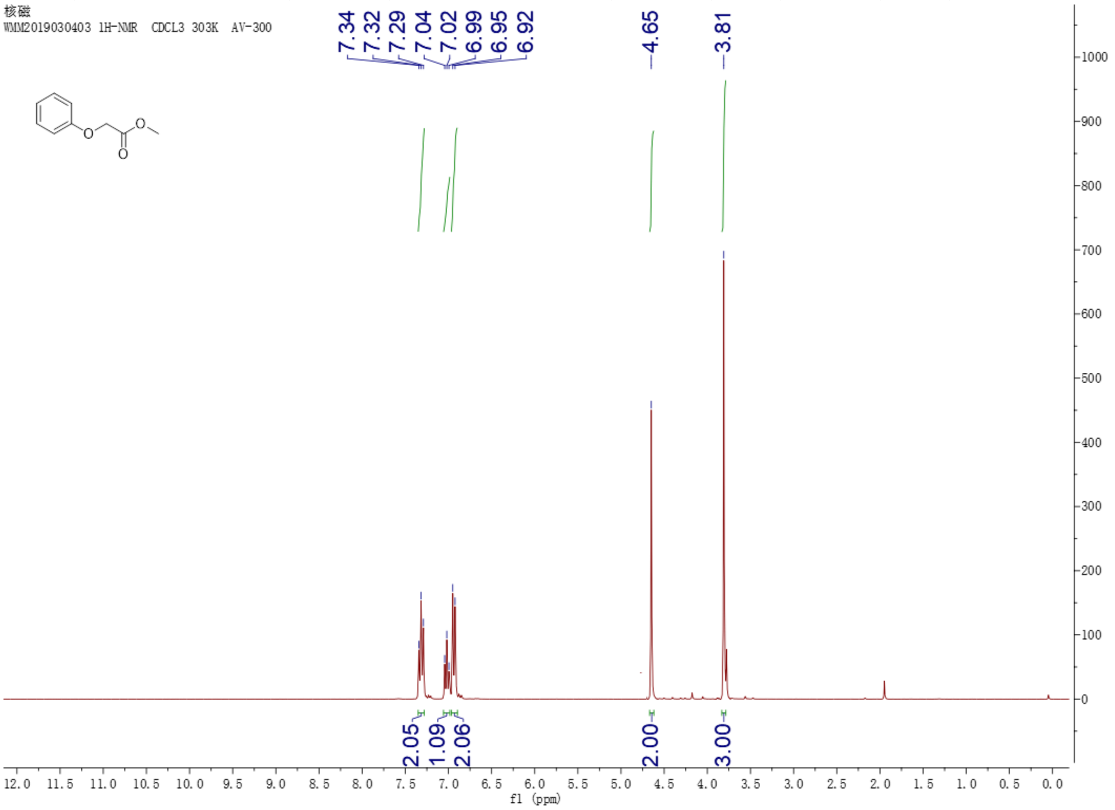

Methyl 2-(2,4,6-trichlorophenoxy)acetate (C9–C12):

Yield, 95%; white solid; ^{1}H NMR (300 MHz, $CDCl_3$) δ 7.33 (s, 1H), 4.63 (s, 1H), 3.86 (s, 2H).

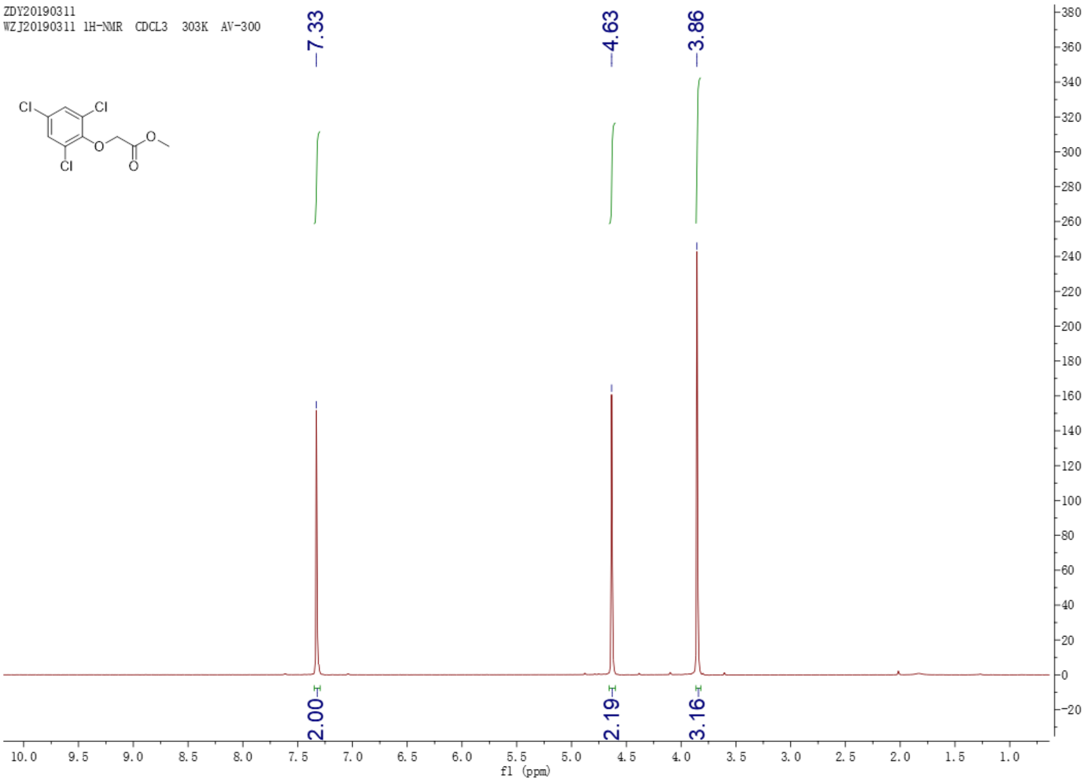

Methyl 2-(2-nitrophenoxy)acetate (C13–C16):

Yield, 92%; white solid; ^{1}H NMR (300 MHz, DMSO) δ 7.88 (d, J = 8.0 Hz, 1H), 7.62 (t, J = 7.6 Hz, 1H), 7.30 (d, J = 8.5 Hz, 1H), 7.15 (t, J = 7.7 Hz, 1H), 5.03 (s, 2H), 3.70 (s, 3H).

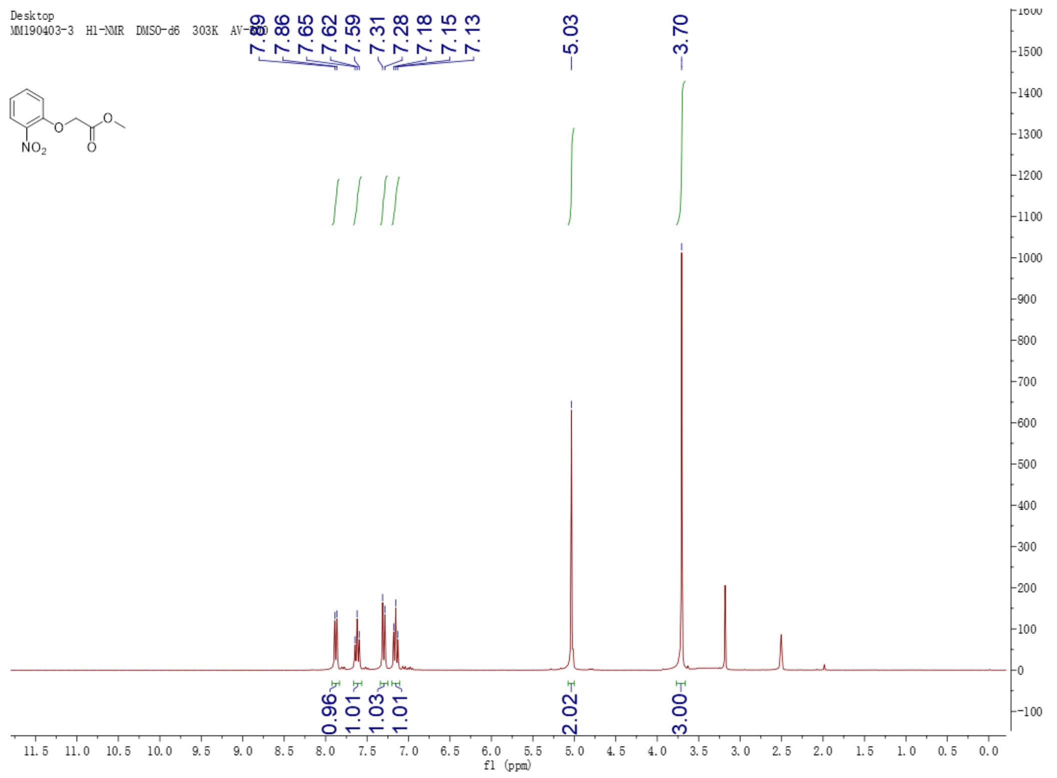

Methyl 2-(4-nitrophenoxy)acetate (C17–C21):

Yield, 86%; white solid; ^{1}H NMR (300 MHz, $CDCl_3$) δ 8.21 (d, J = 9.2 Hz, 2H), 6.99 (d, J = 9.2 Hz, 2H), 4.76 (s, 2H), 3.84 (s, 3H).

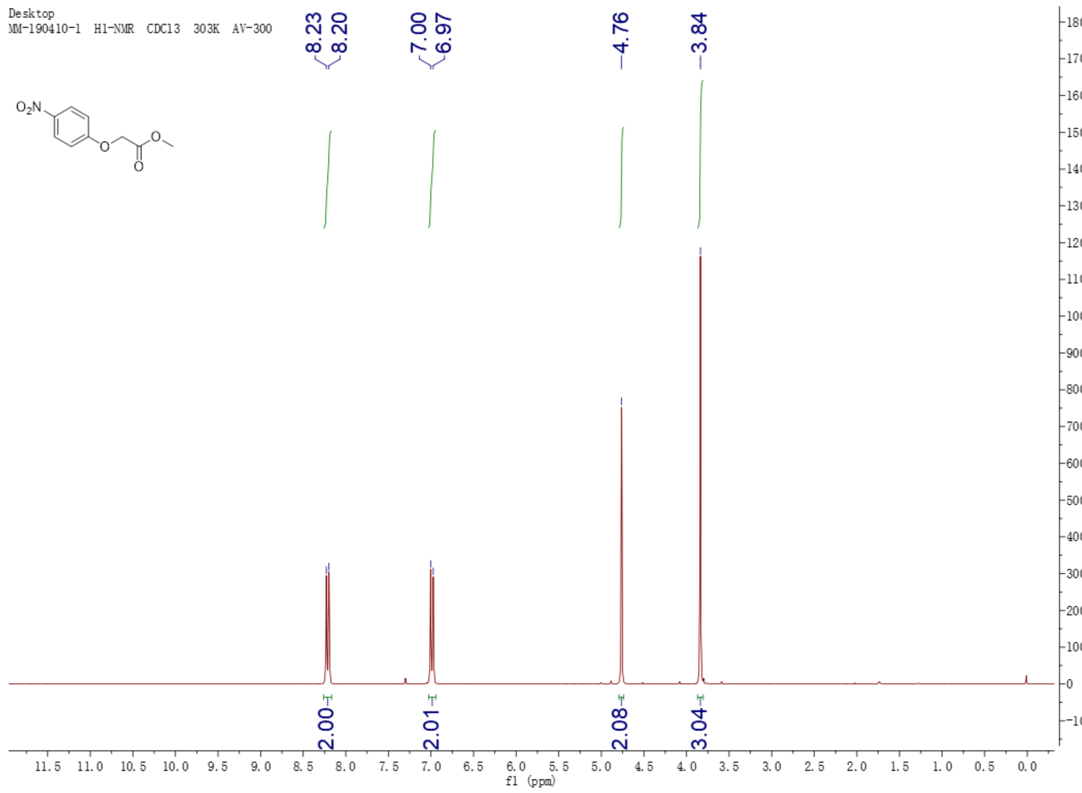

Methyl 2-(2-chloro-4-nitrophenoxy)acetate (C22–C25):

Yield, 90%; white solid; ^{1}H NMR (300 MHz, $CDCl_3$) δ 8.31 (d, J = 2.7 Hz, 1H), 8.13 (dd, J = 9.1, 2.7 Hz, 1H), 6.90 (d, J = 9.1 Hz, 1H), 4.86 (s, 2H), 3.84 (s, 3H).

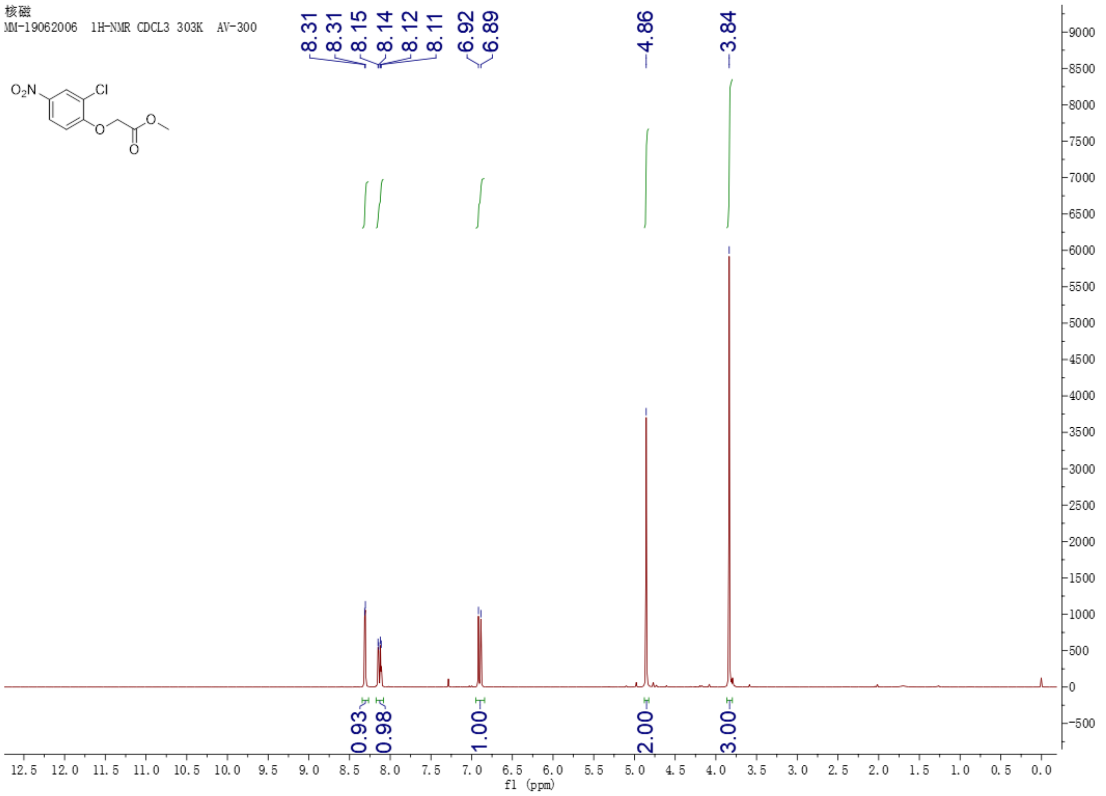

Methyl 2-phenoxyacetate (C39, C40):

Yield, 90%; white solid; ^{1}H NMR (300 MHz, $CDCl_3$) δ 7.32 (t, J = 8.0 Hz, 2H), 7.02 (t, J = 7.4 Hz, 1H), 6.94 (d, J = 8.0 Hz, 2H), 4.65 (s, 2H), 3.81 (s, 3H).

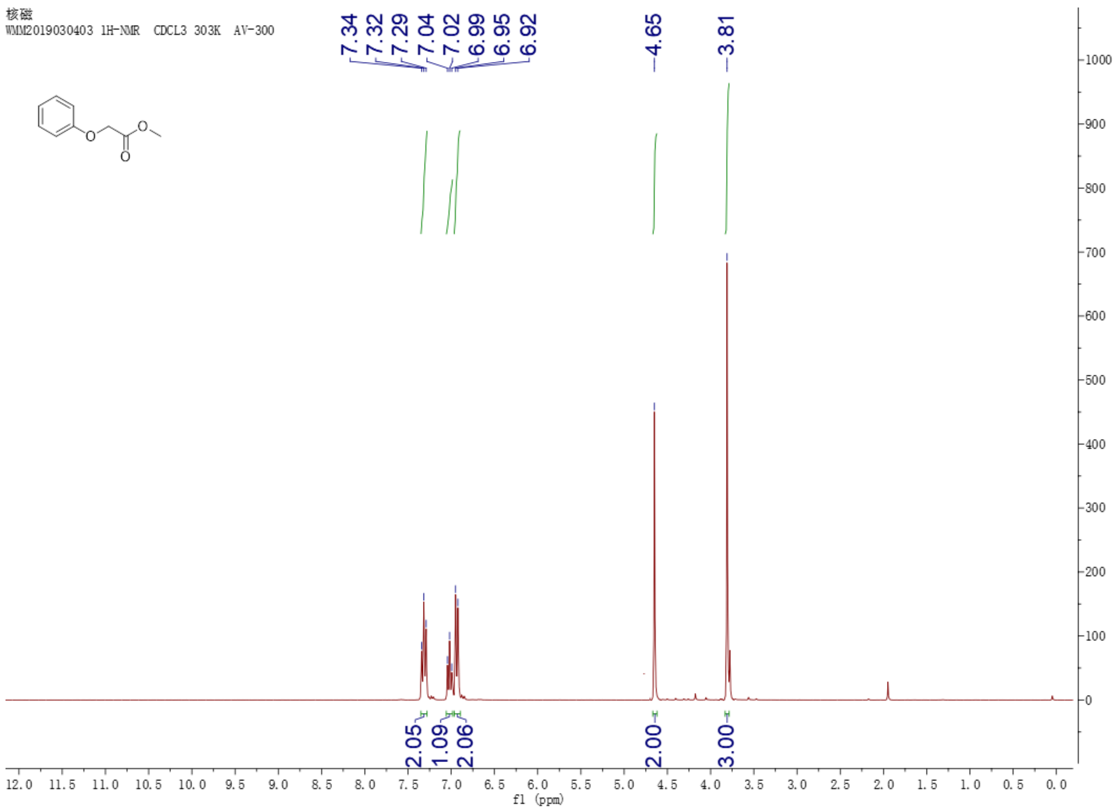

Characterization of selected intermediates D

2-Phenoxyacetic acid (D1, D2):

Yield, 90%; white solid; ^{1}H NMR (300 MHz, $CDCl_3$) δ 10.01 (s, 1H), 7.34 (t, J = 7.8 Hz, 2H), 7.05 (t, J = 7.3 Hz, 1H), 6.96 (d, J =7.8 Hz, 2H), 4.72 (s, 2H).

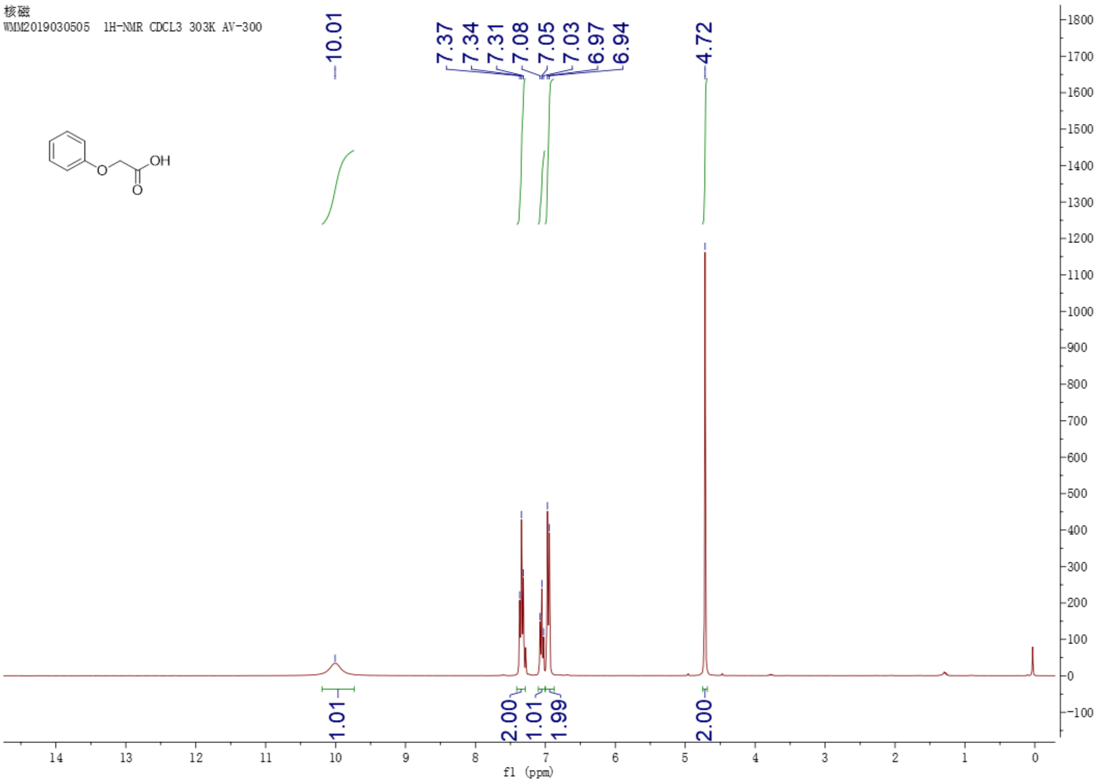

2-(2-Chlorophenoxy)acetic acid (D3, D4):

Yield, 95%; white solid; ^{1}H NMR (300 MHz, DMSO) δ 12.12 (s, 1H), 7.42 (d, J = 7.8 Hz, 1H), 7.26 (d, J = 7.6 Hz, 1H), 6.97 (dd, J = 18.8, 8.0 Hz, 2H), 4.76 (s, 2H).

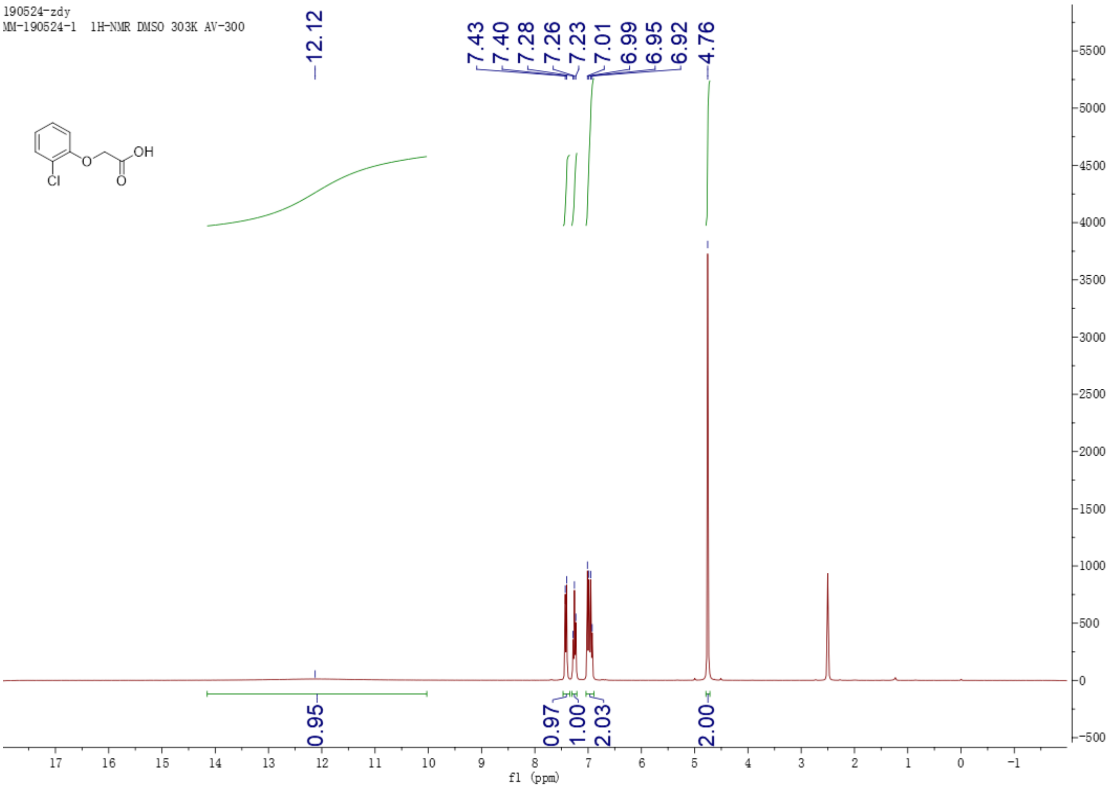

2-(4-Chlorophenoxy)acetic acid (D5, D6):

Yield, 91%; white solid; ^{1}H NMR (300 MHz, DMSO) δ 13.01 (s, 1H), 7.31 (d, J = 8.8 Hz, 2H), 6.94 (d, J = 8.8 Hz, 2H), 4.68 (s, 2H).

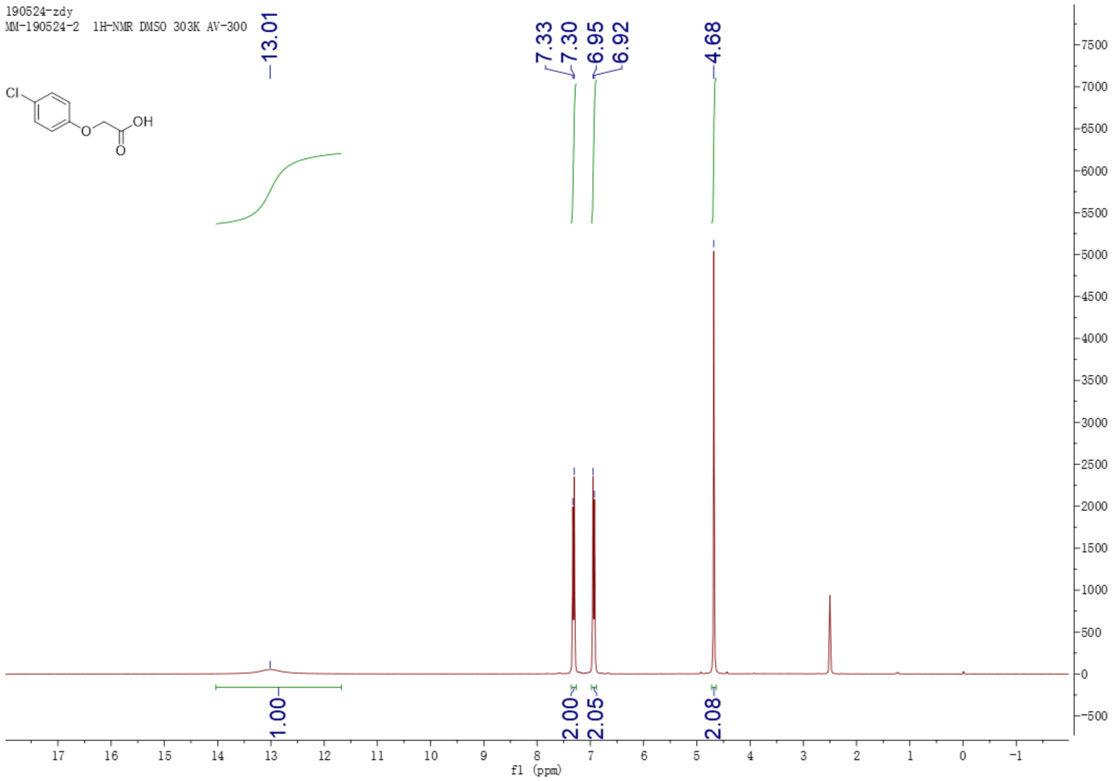

2-(2,4-Dichlorophenoxy)acetic acid (D7, D8):

Yield, 96%; white solid; ^{1}H NMR (300 MHz, DMSO) δ 13.14 (s, 1H), 7.55 (s, J = 2.2 Hz, 1H), 7.33 (d, J = 8.8 Hz, 1H), 7.06 (d, J = 8.8 Hz, 1H), 4.82 (s, 2H).

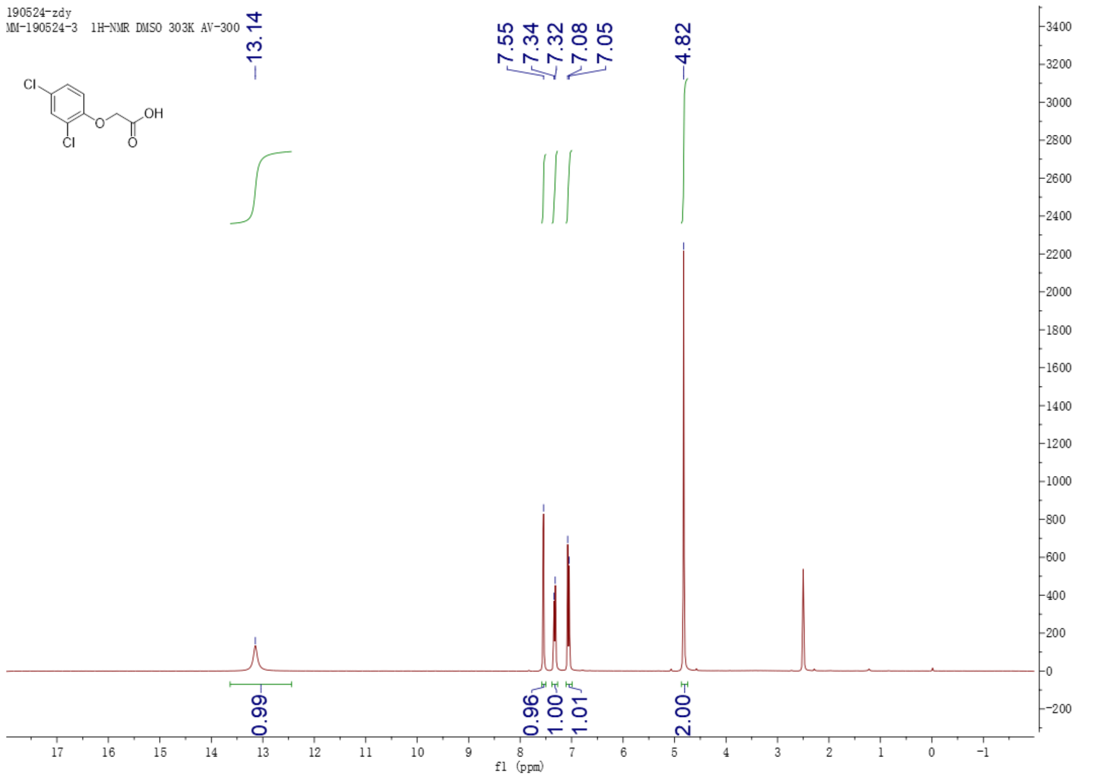

2-(2,4,6-Trichlorophenoxy)acetic acid (D9–D12):

Yield, 87%; white solid; ^{1}H NMR (300 MHz, DMSO) δ 13.09 (s, 1H), 7.63 (s, 2H), 4.57 (s, 2H).

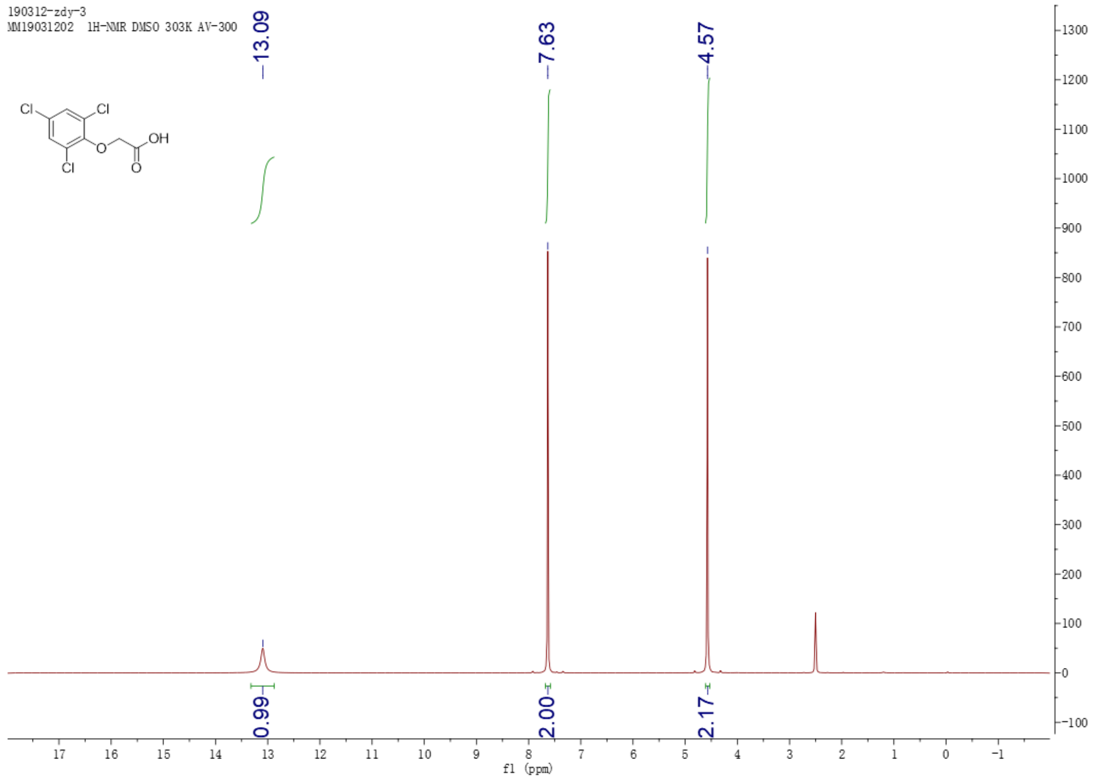

2-(2-Nitrophenoxy)acetic acid (D13–D16):

Yield, 83%; white solid;^{1}H NMR (300 MHz, DMSO) δ 13.19 (s, 1H), 7.85 (d, J = 8.0 Hz, 1H), 7.60 (t, J = 8.0 Hz, 1H), 7.24 (d, J = 8.5 Hz, 1H), 7.12 (t, J = 7.7 Hz, 1H), 4.90 (s, 3H).

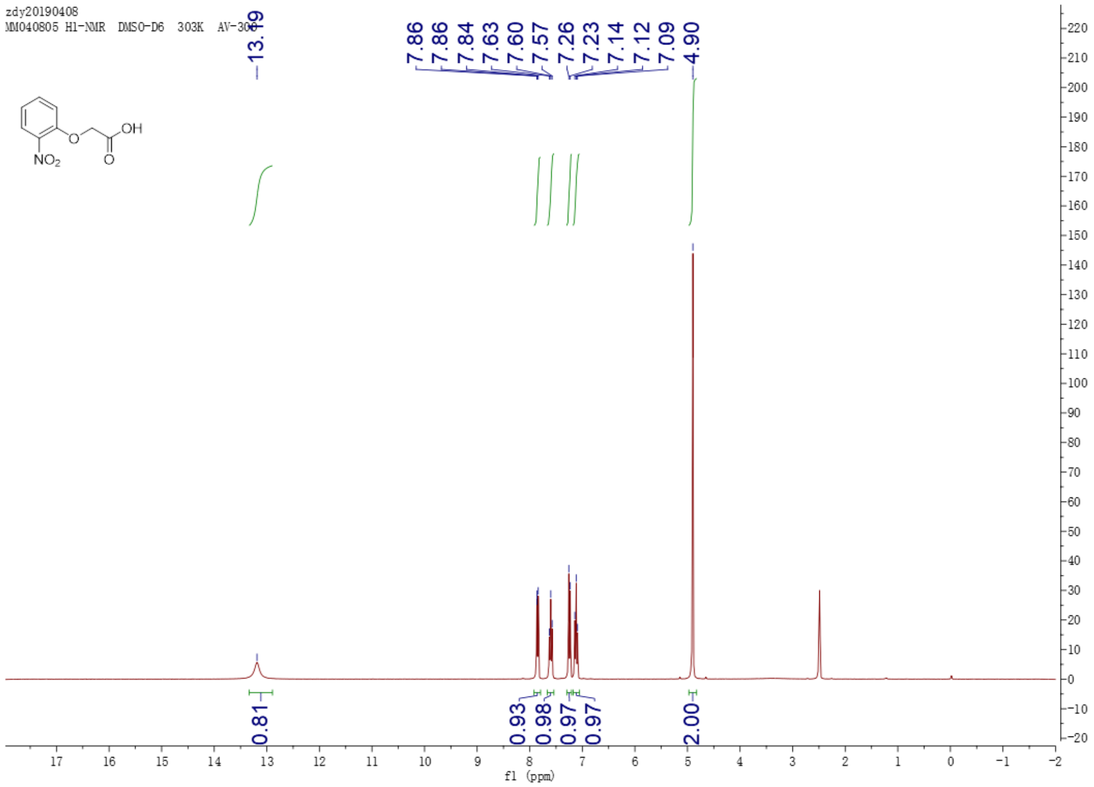

2-(4-Nitrophenoxy)acetic acid (D17–D21):

Yield, 95%; white solid; ^{1}H NMR (300 MHz, DMSO) δ 13.21 (s, 1H), 8.19 (d, J = 8.7 Hz, 2H), 7.12 (d, J = 8.7 Hz, 2H), 4.87 (s, 2H).

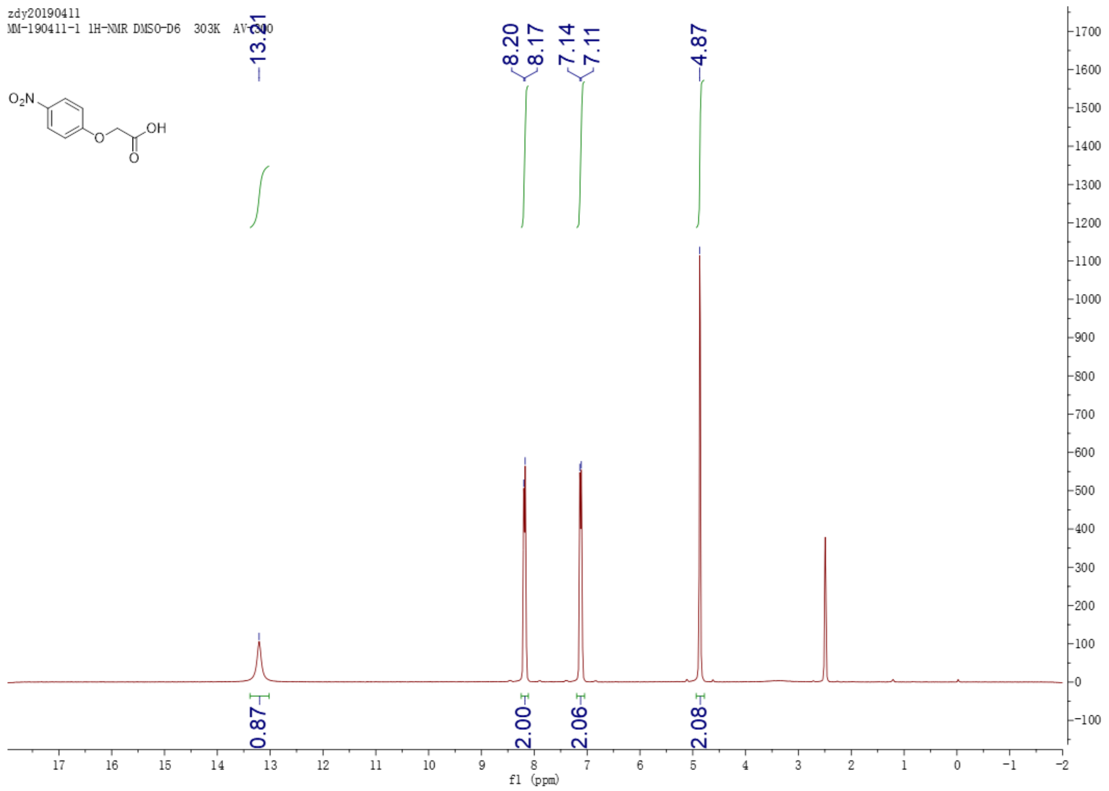

2-(2-Chloro-4-nitrophenoxy)acetic acid (D22–D25):

Yield, 81%; white solid; ^{1}H NMR (300 MHz, DMSO) δ 13.27 (s, 1H), 8.27 (d, J = 2.7 Hz, 1H), 8.16 (dd, J = 9.2, 2.7 Hz, 1H), 7.26 (d, J = 9.2 Hz, 1H), 5.00 (s, 2H).

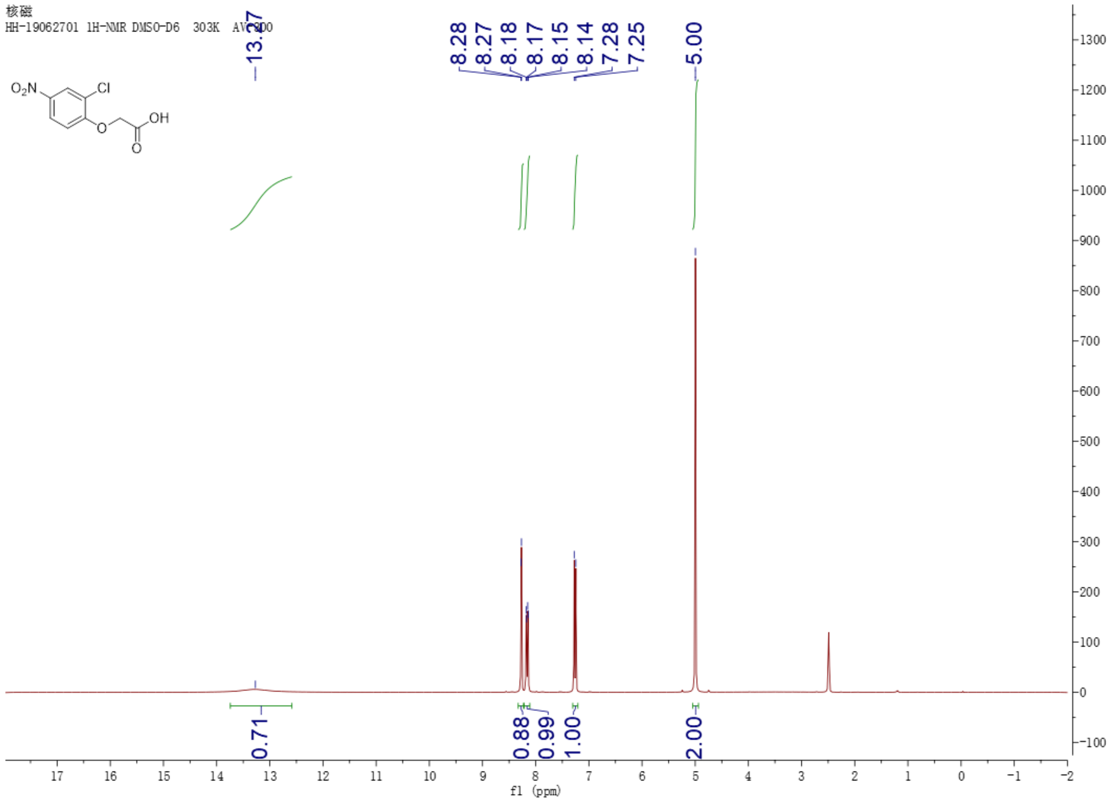

2-(5-Chloro-2-(2,4-dichlorophenoxy)phenoxy)acetic acid (D26–D29):

Yield, 90%; white solid; ^{1}H NMR (300 MHz, $CDCl_3$) δ 9.80 (s, 1H), 7.47 (d, J = 2.4 Hz, 1H), 7.18-7.01 (m, 1H), 7.02-6.87 (m, 2H), 6.89 (d, J = 8.8 Hz, 1H), 6.80 (d, J = 8.8 Hz, 1H), 4.75 (s, 2H). ^{13}C NMR (75 MHz, DMSO) δ 169.49, 151.78, 150.09, 142.30, 129.68, 129.46, 128.26, 126.98, 123.38, 122.18, 121.41, 118.65, 114.87, 65.22. MS (ESI) m/z 345.0 $[M-H]^-$.

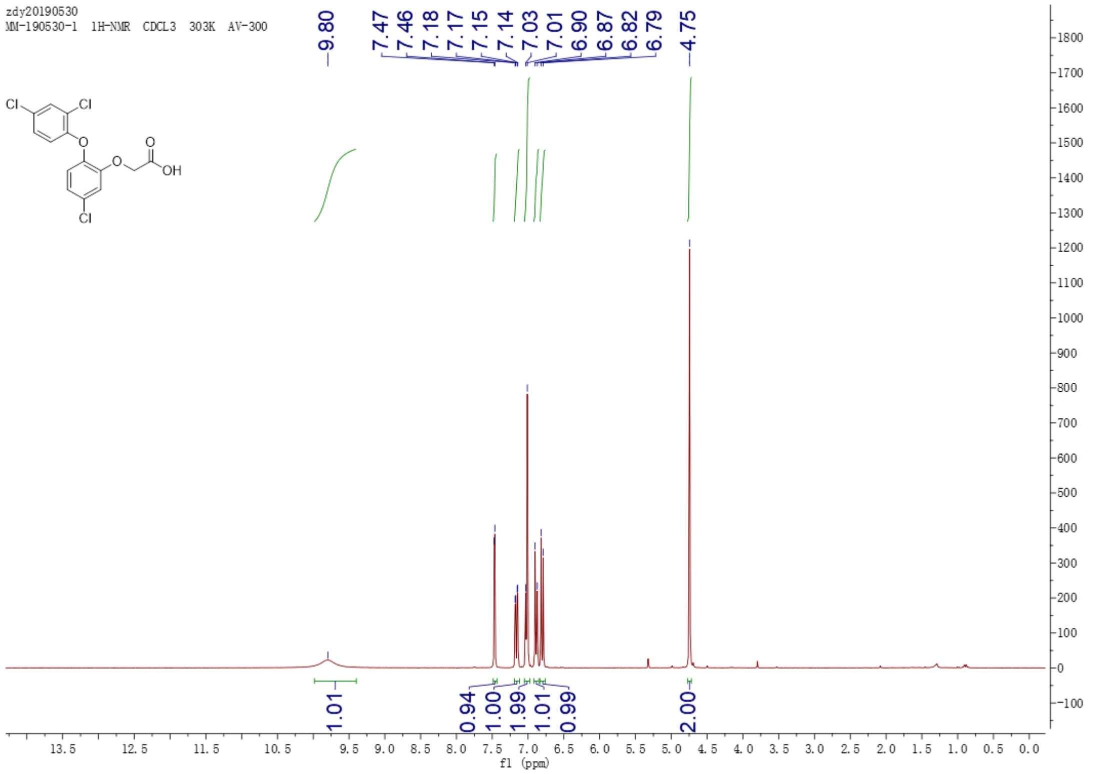

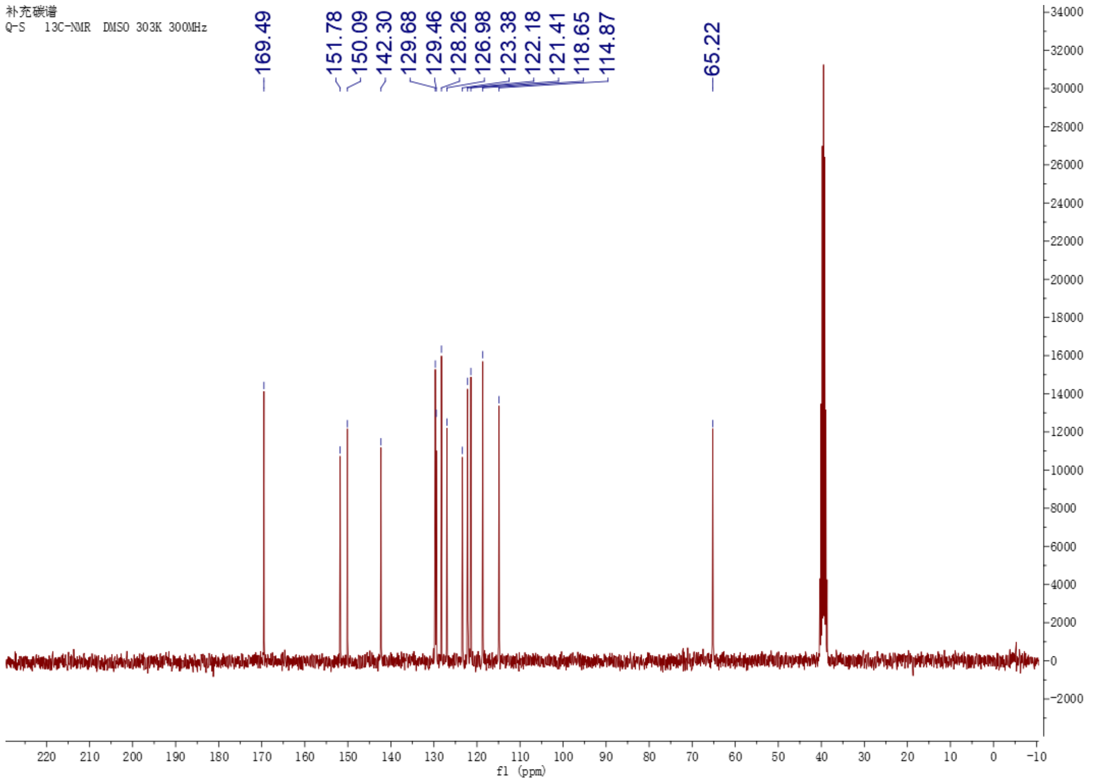

2-(2,4-Dimethylphenoxy)acetic acid (D30–D34):

Yield, 86%; white solid; ^{1}H NMR (300 MHz, DMSO) δ 12.90 (s, 1H), 6.93 (s, 1H), 6.89 (d, *J* = 8.3 Hz, 1H), 6.67 (d, *J* = 8.2 Hz, 1H), 4.61 (s, 2H), 2.18 (s, 3H), 2.15 (s, 3H).

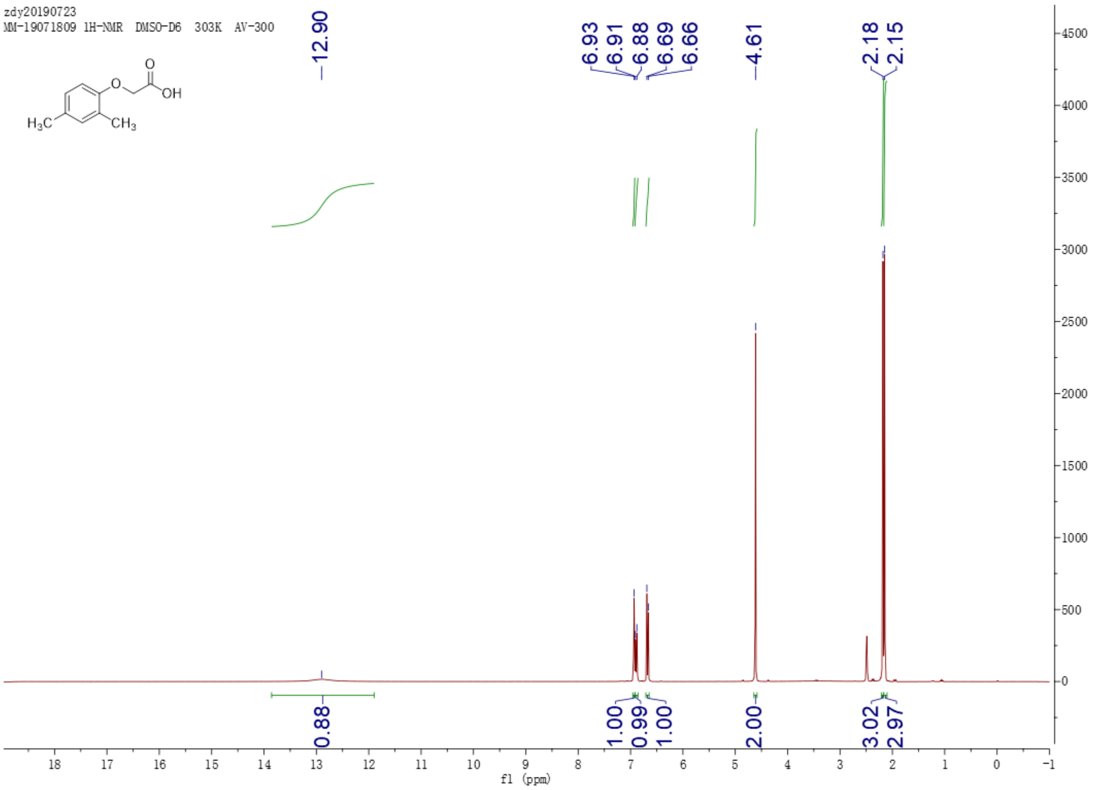

2-(2-Methoxy-4-methylphenoxy)acetic acid (D35–D38):

Yield, 88%; white solid; ^{1}H NMR (300 MHz, DMSO) δ 12.88 (s, 1H), 6.79 (d, J = 1.5 Hz, 1H), 6.73 (d, J = 8.1 Hz, 1H), 6.63 (dd, J = 8.1, 1.1 Hz, 1H), 4.57 (s, 2H), 3.74 (s, 3H), 2.22 (s, 3H).

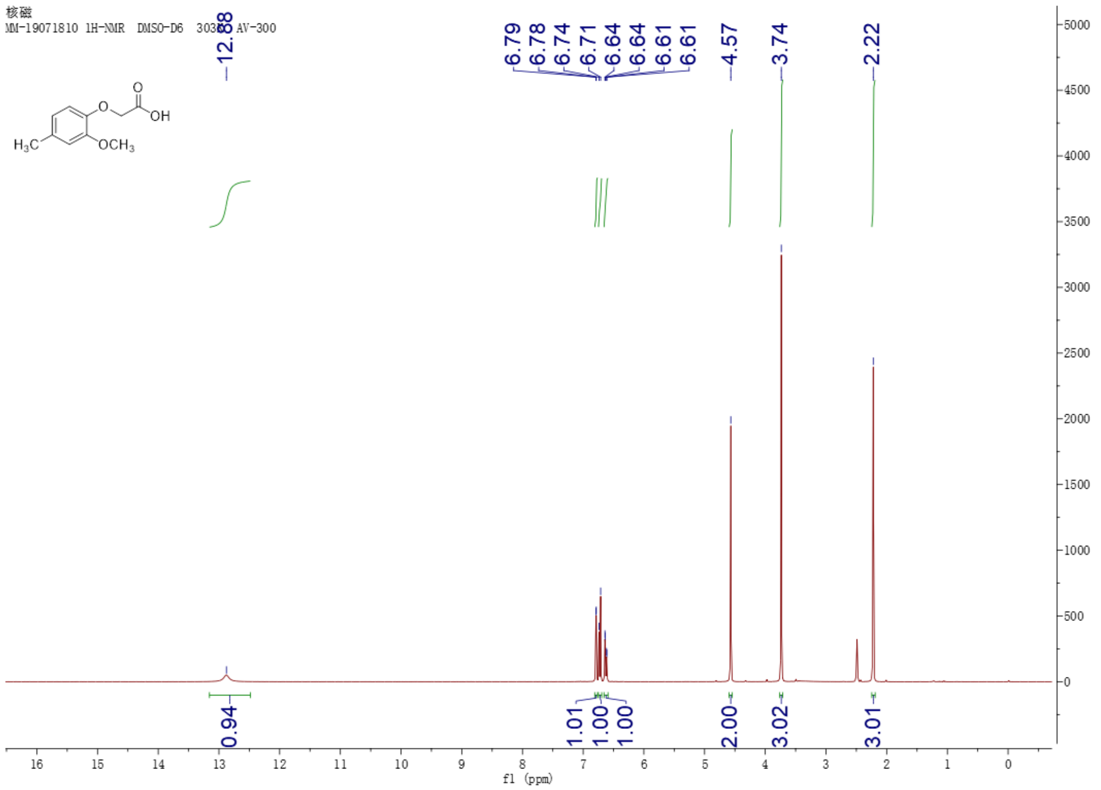

2-Phenoxyacetic acid (D39, D40):

Yield, 90%; white solid; ^{1}H NMR (300 MHz, $CDCl_3$) δ 10.01 (s, 1H), 7.34 (t, J = 7.8 Hz, 2H), 7.05 (t, J = 7.3 Hz, 1H), 6.96 (d, J =7.8 Hz, 2H), 4.72 (s, 2H).

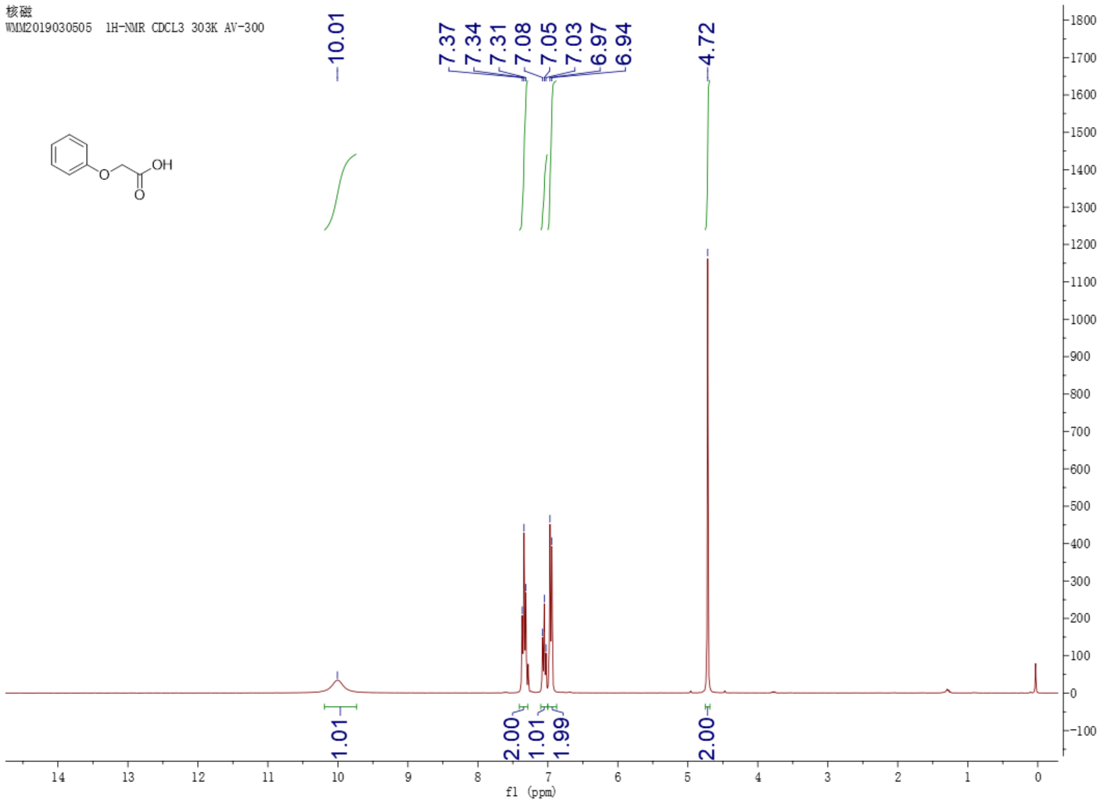

Characterization of selected intermediates E

3-Cyclopropyl-1-methyl-1*H*-pyrazol-5-yl 2-phenoxyacetate (E1):

Yield, 85%; white solid; ^{1}H NMR (300 MHz, $CDCl_3$) δ 7.35 (t, J = 7.9 Hz, 2H), 7.06 (t, J = 7.3 Hz, 1H), 6.98 (d, J = 7.9 Hz, 2H), 5.90 (s, 1H), 4.90 (s, 2H), 3.55 (s, 3H), 1.90-1.87 (m, 1H), 0.99 – 0.85 (m, 2H), 0.73 (dd, J = 7.6, 2.9 Hz, 2H). MS (ESI) *m/z* 273.1 $[M+H]^{-}$.

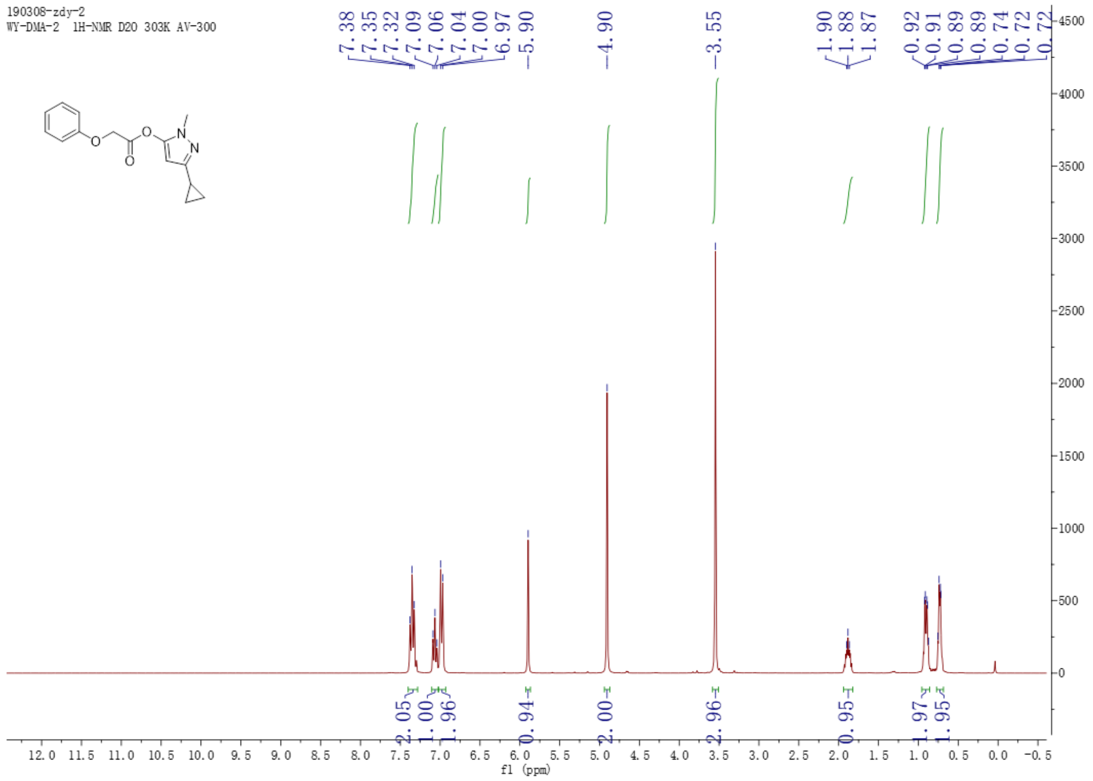

1-Methyl-3-(trifluoromethyl)-1*H*-pyrazol-5-yl 2-(2-chlorophenoxy)acetate (E4):

Yield, 76%; white solid; ^{1}H NMR (300 MHz, $CDCl_3$) δ 7.48 (dd, J = 7.9, 1.4 Hz, 1H), 7.35 (m, 1H), 7.06 (td, J = 7.8, 1.1 Hz, 1H), 6.98 (d, J = 8.2 Hz, 1H), 6.57 (s, 1H), 5.04 (s, 2H), 3.74 (s, 3H). MS (ESI) *m/z* 335.2 $[M+H]^{+}$.

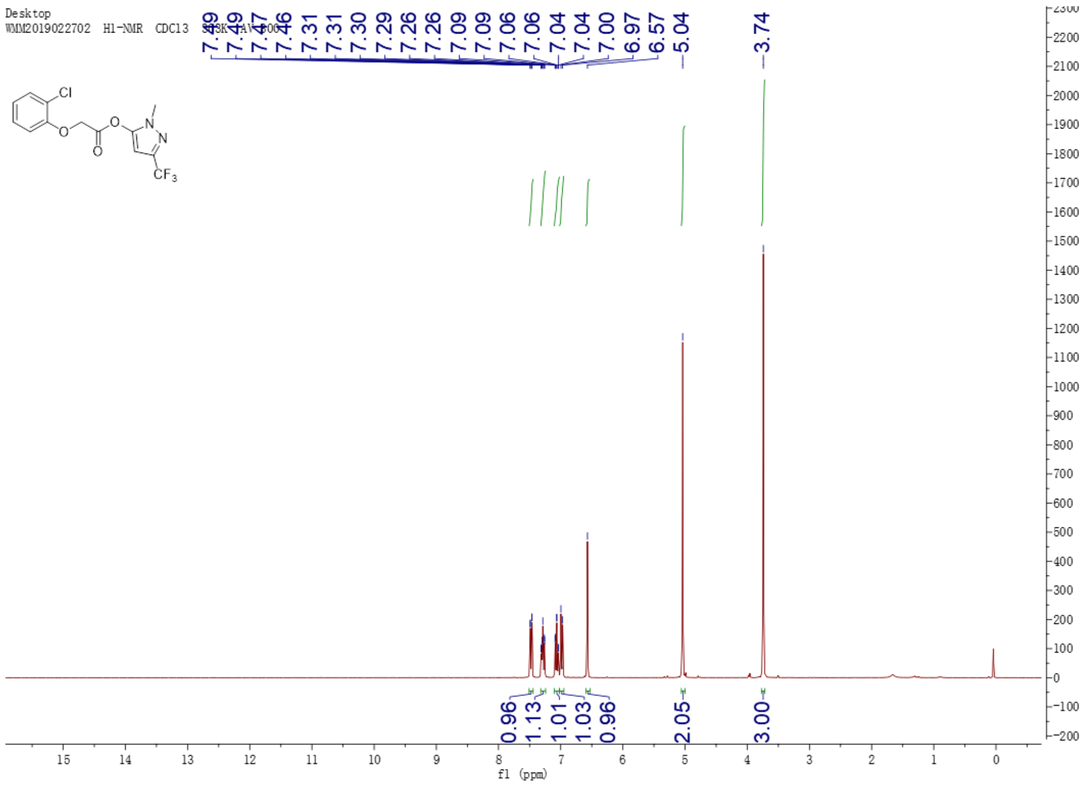

5,5-Dimethyl-3-oxocyclohex-1-en-1-yl 2-(4-chlorophenoxy)acetate (E5):

Yield, 70%; white solid; ^{1}H NMR (300 MHz, $CDCl_3$) δ 7.4(d, J = 8.9 Hz, 2H), 6.90 (d, J = 8.9 Hz, 1H), 5.99 (s, 1H), 4.78 (s, 1H), 2.46 (s, 2H), 2.32 (s, 2H), 1.15 (s, 6H). MS (ESI) m/z 308.3 $[M+H]^{+}$.

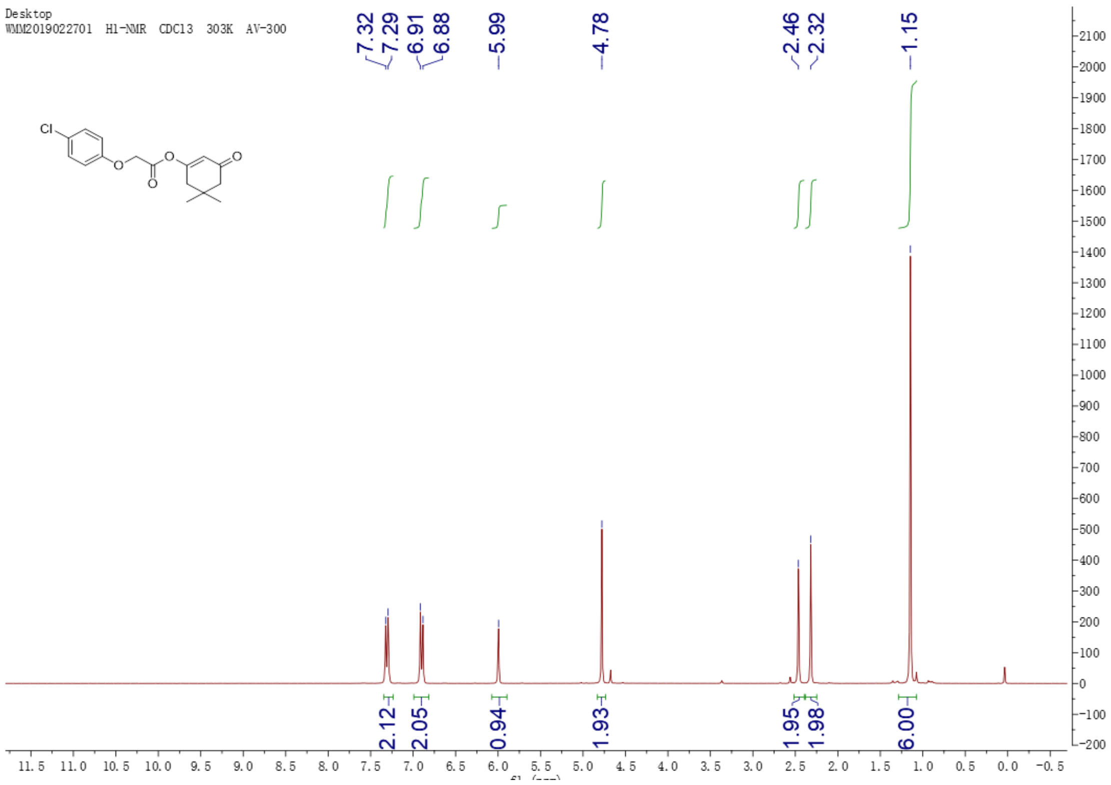

1-Methyl-3-(trifluoromethyl)-1*H*-pyrazol-5-yl 2-(4-chlorophenoxy)acetate (E6):

Yield, 67%; white solid; ^{1}H NMR (300 MHz, $CDCl_3$) δ 7.32 (d, J = 8.9 Hz, 2H), 6.93 (d, J = 8.9Hz, 2H), 6.54 (s, 2H), 4.94 (s, 1H), 3.73 (s, 3H). MS (ESI) m/z 357.1 $[M+Na]^+$.

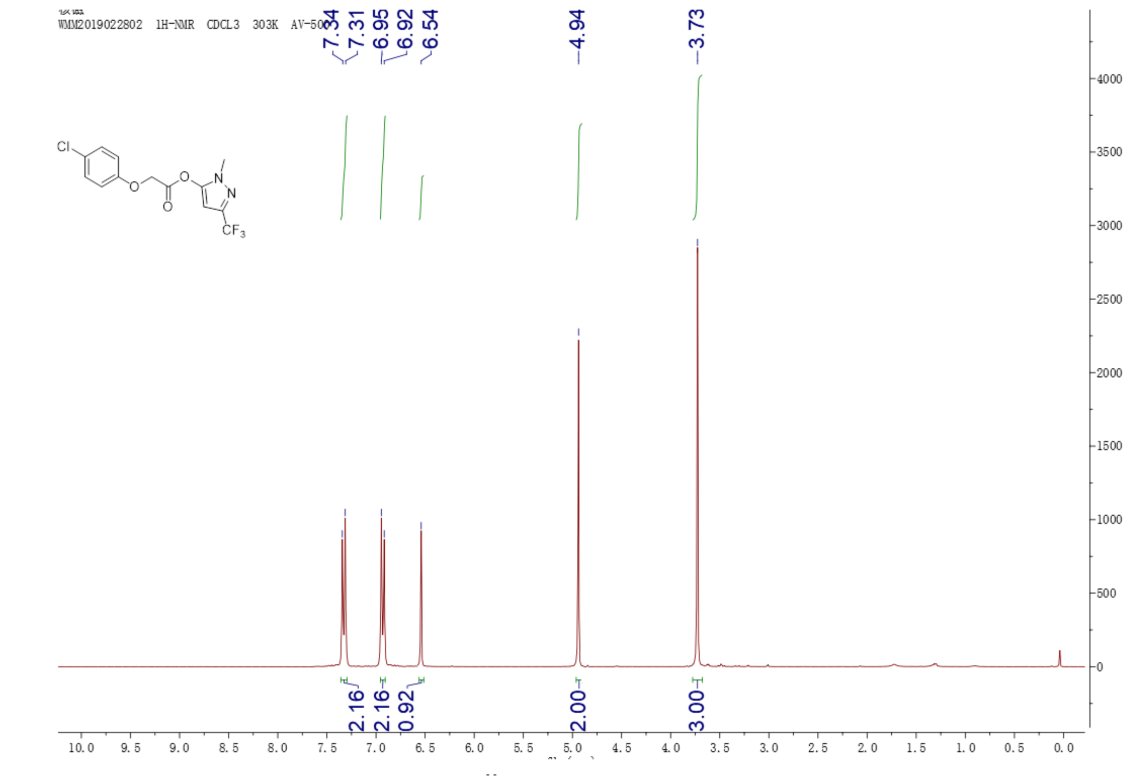

3-Cyclopropyl-1-methyl-1*H*-pyrazol-5-yl 2-(2,4-dichlorophenoxy)acetate (E7):

Yield, 69%; white solid; ^{1}H NMR (300 MHz, $CDCl_3$) δ 7.42 (s, 1H), 7.30 – 7.11 (d, J = 13.1 Hz ,1H), 6.82 (d, J = 13.1 Hz, 1H), 5.88 (s, 1H), 4.93 (s, 2H), 3.58 (s, 3H), 2.02 – 1.69 (m, 1H), 1.06 – 0.79 (m, 2H), 0.76 – 0.59 (m, 2H). MS (ESI) *m/z* 339.1 [M−H]$^{-}$.

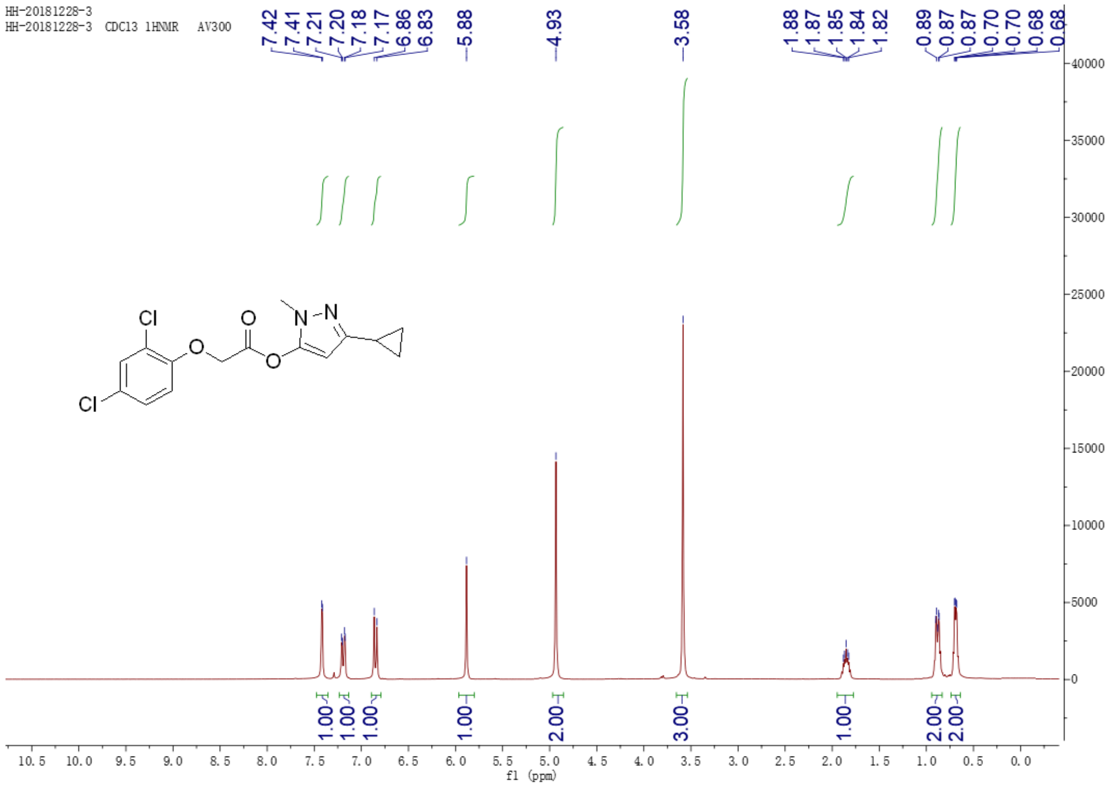

1-Methyl-3-(trifluoromethyl)-1*H*-pyrazol-5-yl-2-(2,4-dichlorophenoxy)acetate (E8):

Yield, 65%; white solid; ^{1}H NMR (300 MHz, $CDCl_3$) δ 7.47 (s, 1H), 7.24 (d, J = 8.8 Hz, 1H), 6.92 (d, J = 8.8 Hz, 1H), 6.56 (s, 1H), 5.01 (s, 2H), 3.77 (s, 3H). MS (ESI) m/z 367.1 $[M-H]^-$.

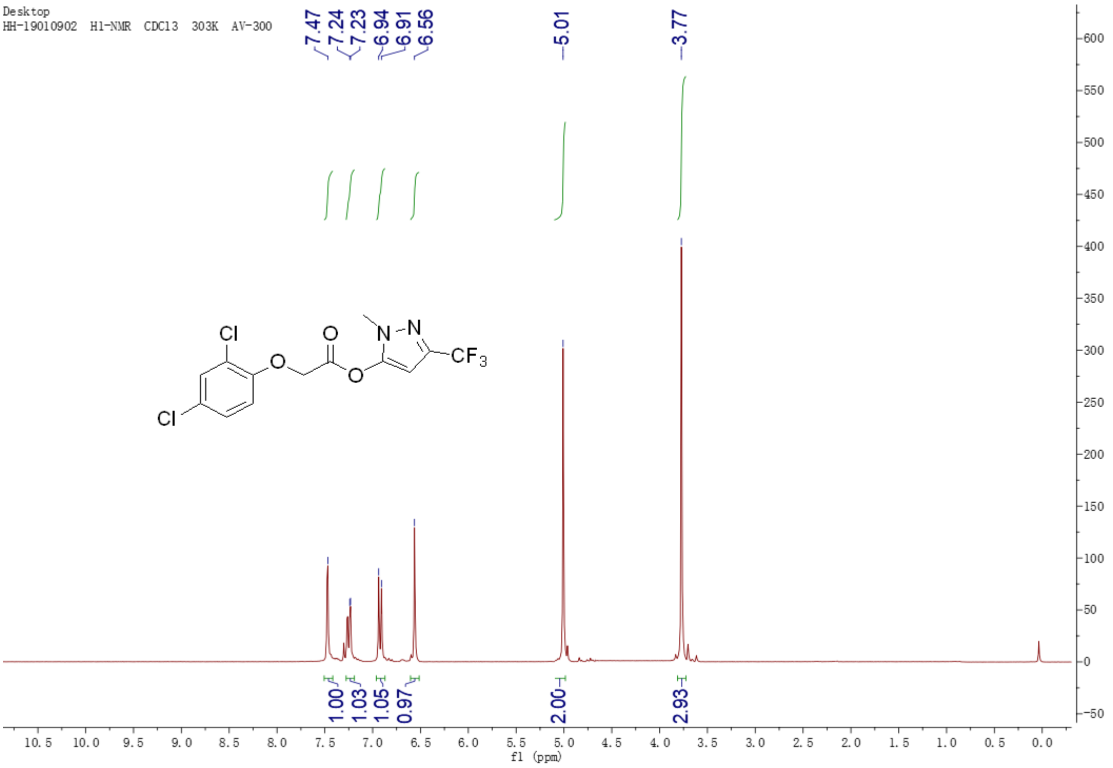

5,5-Dimethyl-3-oxocyclohex-1-en-1-yl 2-(2,4,6-trichlorophenoxy)acetate (E9):

Yield, 65%; white solid; ^{1}H NMR (300 MHz, $CDCl_3$) δ 7.37 (s, 2H), 6.04 (s, 1H), 4.78 (s, 2H), 2.52 (s, 2H), 2.33 (s, 2H), 1.16 (s, 6H). MS (ESI) m/z 399.0 $[M+Na]^+$.

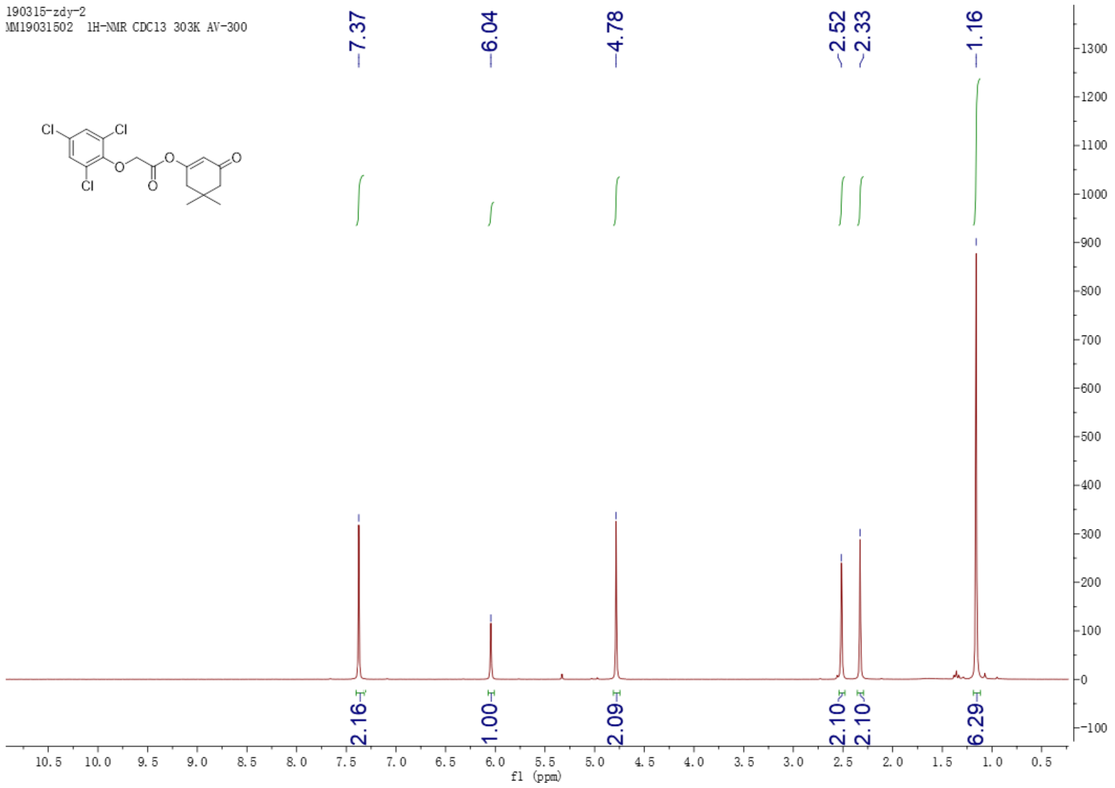

1,3-Dimethyl-1*H*-pyrazol-5-yl 2-(2,4,6-trichlorophenoxy)acetate (E10):

Yield, 60%; white solid; ^{1}H NMR (300 MHz, $CDCl_3$) δ 7.39 (s, 2H), 6.07 (s, 1H), 4.90 (s, 2H), 3.70 (s, 3H), 2.28 (s, 3H). MS (ESI) *m/z* 371.2 [M+Na] $^{+}$.

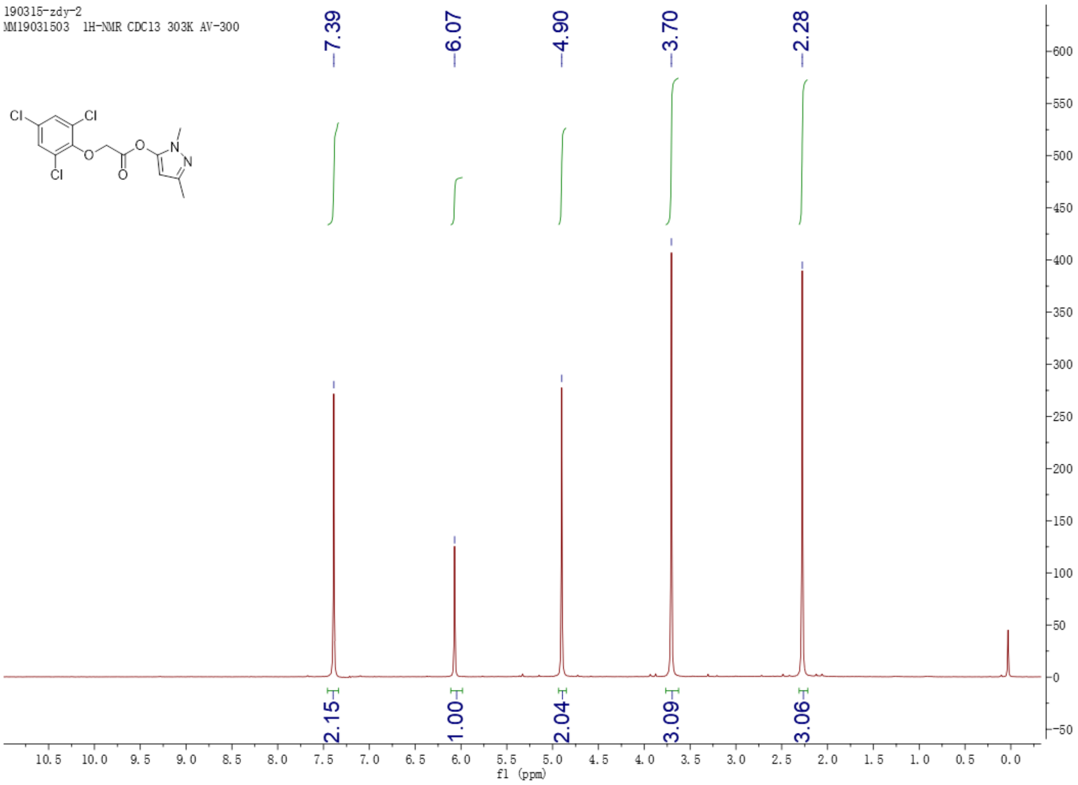

3-Cyclopropyl-1-methyl-1*H*-pyrazol-5-yl 2-(2,4,6-trichlorophenoxy)acetate (E11): Yield, 63%; white solid; ^{1}H NMR (300 MHz, $CDCl_3$) δ 7.39 (s, 2H), 5.95 (s, 1H), 4.89 (s, 2H), 3.69 (s, 3H), 2.07 – 1.78 (m, 1H), 0.98 – 0.84 (m, 2H), 0.76 (dd, J = 7.5, 3.1 Hz, 2H). MS (ESI) *m/z* 371.2 $[M+Na]^+$.

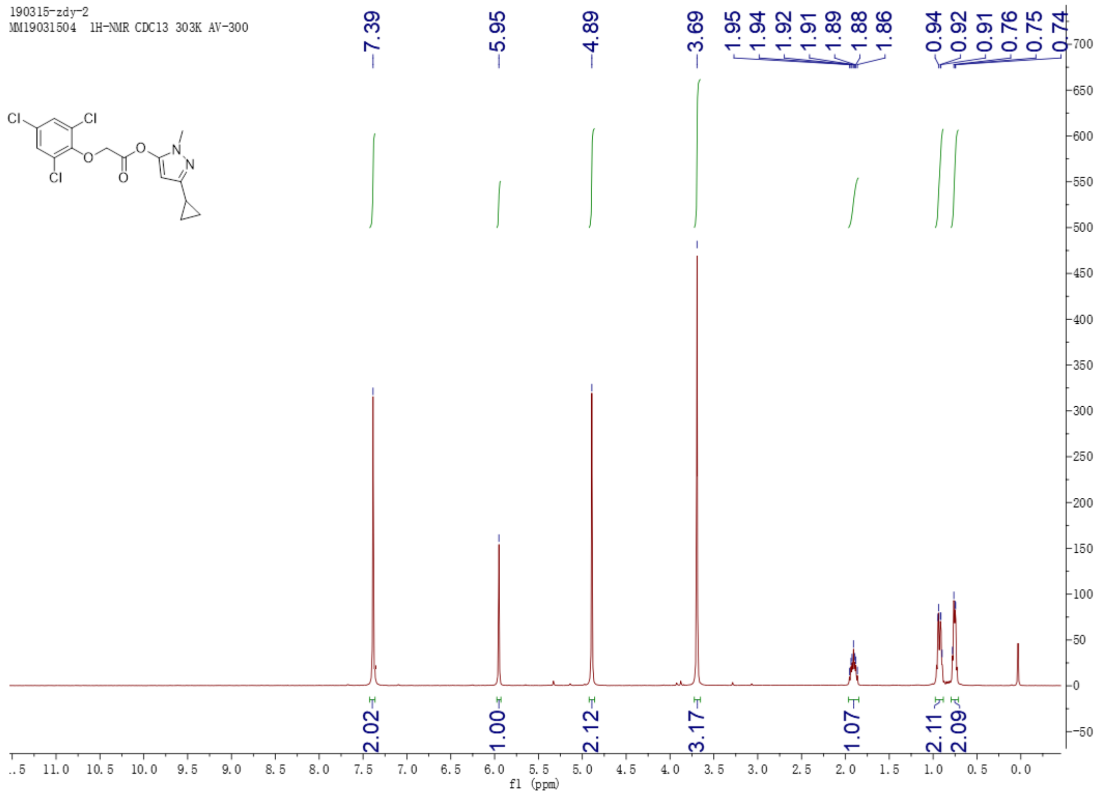

1-Methyl-3-(trifluoromethyl)-1*H*-pyrazol-5-yl 2-(2,4,6-trichlorophenoxy)acetate (E12):

Yield, 67%; white solid; ^{1}H NMR (300 MHz, $CDCl_3$) δ 7.40 (s, 1H), 6.60 (s, 1H), 4.94 (s, 1H), 3.85 (s, 2H). MS (ESI) *m/z* 425.2 [M+Na] $^{+}$.

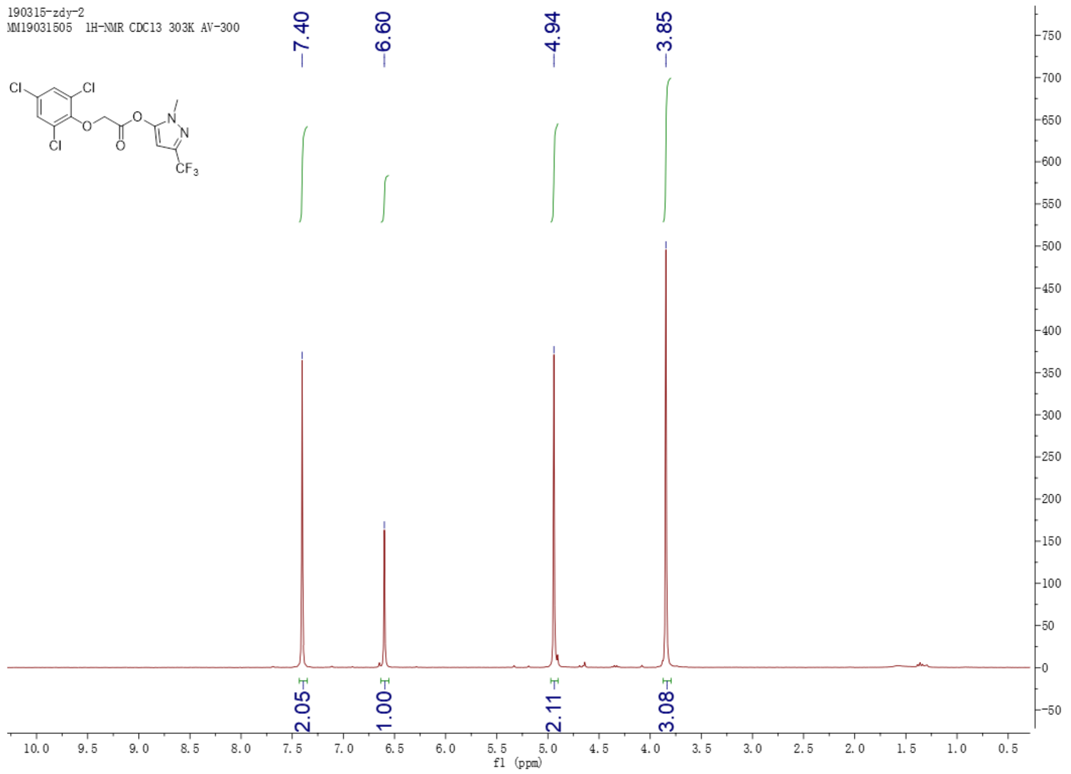

5,5-Dimethyl-3-oxocyclohex-1-en-1-yl 2-(2-nitrophenoxy)acetate (E13):

Yield, 57%; white solid; ^{1}H NMR (300 MHz, $CDCl_3$) δ 7.90 (d, J = 8.0 Hz, 1H), 7.58 (t, J = 7.9 Hz, 1H), 7.18 (t, J = 7.8 Hz, 1H), 7.07 (d, J = 8.3 Hz, 1H), 5.96 (s, 1H), 4.93 (s, 2H), 2.45 (s, 2H), 2.29 (s, 2H), 1.12 (s, 6H). MS (ESI) m/z 342.2 [M+Na]$^+$.

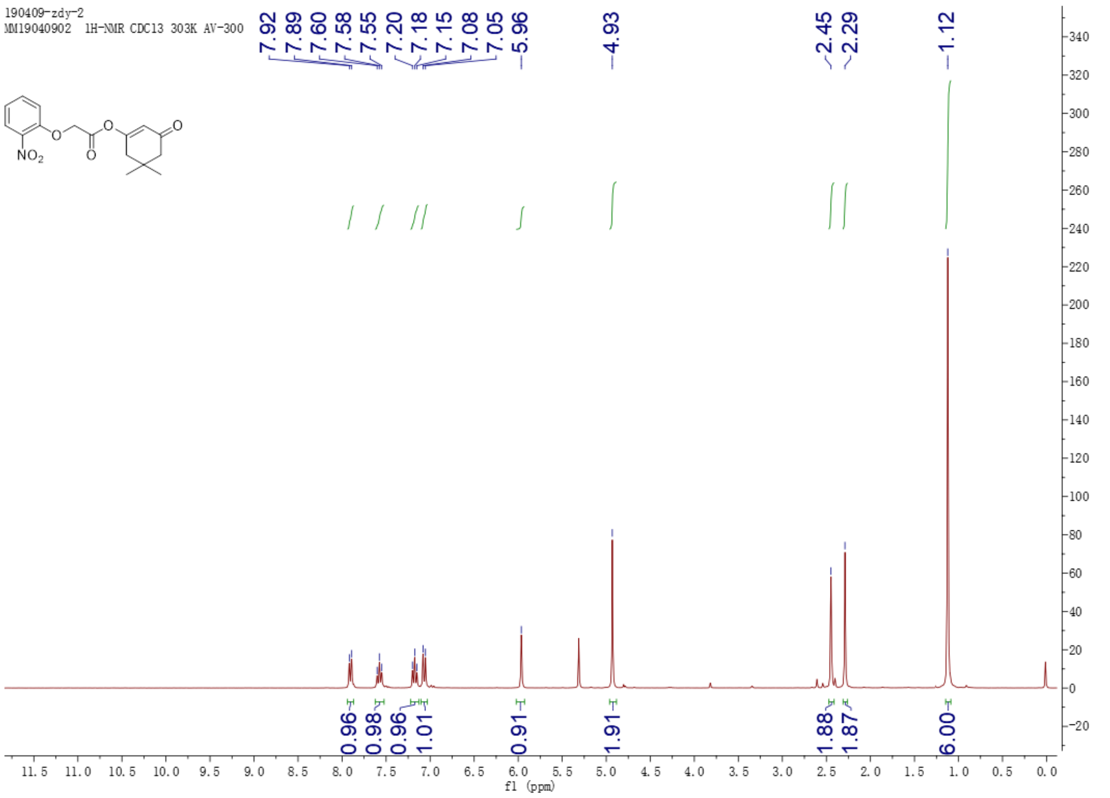

1,3-Dimethyl-1*H*-pyrazol-5-yl 2-(2-nitrophenoxy)acetate (E14):

Yield, 63%; white solid; ^{1}H NMR (300 MHz, $CDCl_3$) δ 7.90 (d, J = 8.2 Hz, 1H), 7.57 (t, J = 7.4 Hz, 1H), 7.18 (t, J = 7.8 Hz, 1H), 7.10 (d, J = 8.4 Hz, 1H), 6.01 (s, 1H), 5.04 (s, 2H), 3.61 (s, 3H), 2.22 (s, 3H). MS (ESI) m/z 314.3 $[M+Na]^+$.

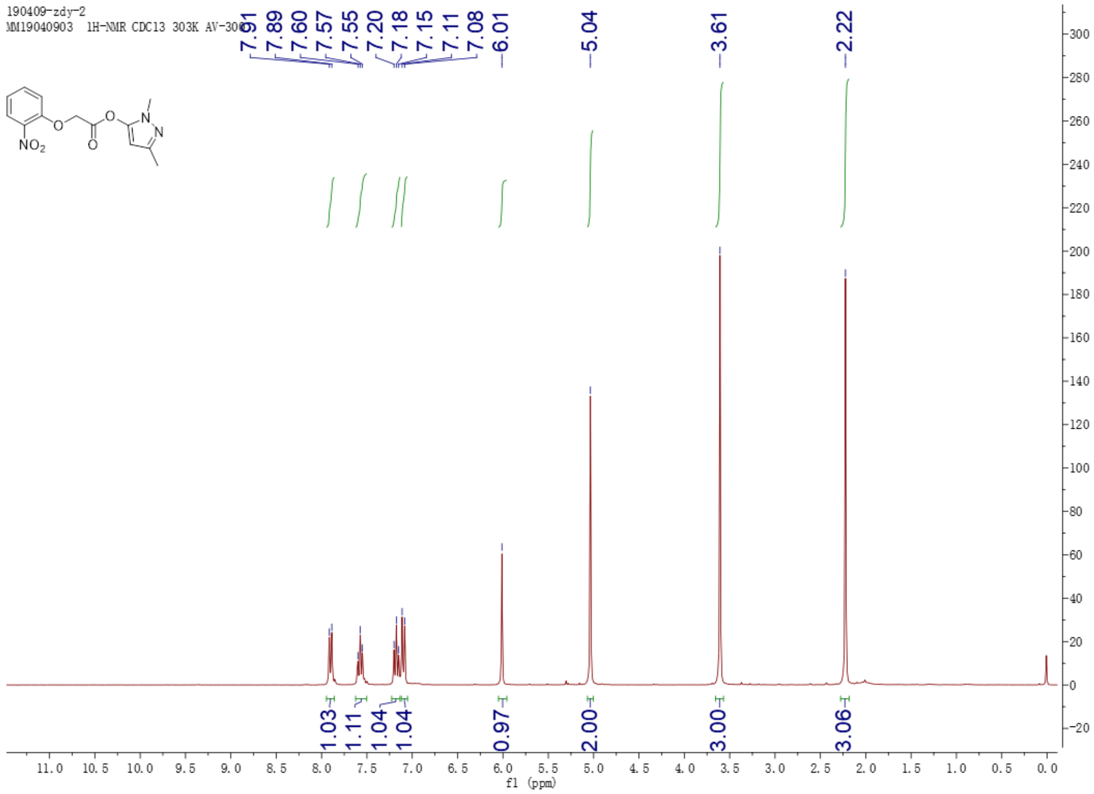

3-Cyclopropyl-1-methyl-1*H*-pyrazol-5-yl 2-(2-nitrophenoxy)acetate (E15):

Yield, 60%; white solid; ^{1}H NMR (300 MHz, $CDCl_3$) δ 7.91 (d, J = 8.1 Hz, 1H), 7.58 (t, J = 7.4 Hz, 1H), 7.18 (t, J = 7.8 Hz, 1H), 7.09 (d, J = 8.4 Hz, 1H), 5.91 (s, 1H), 5.03 (s, 2H), 3.60 (s, 3H), 2.03 – 1.73 (m, 1H), 0.95 – 0.83 (m, 2H), 0.76 – 0.66 (m, 2H). MS (ESI) m/z 340.1 [M+Na]$^{+}$.

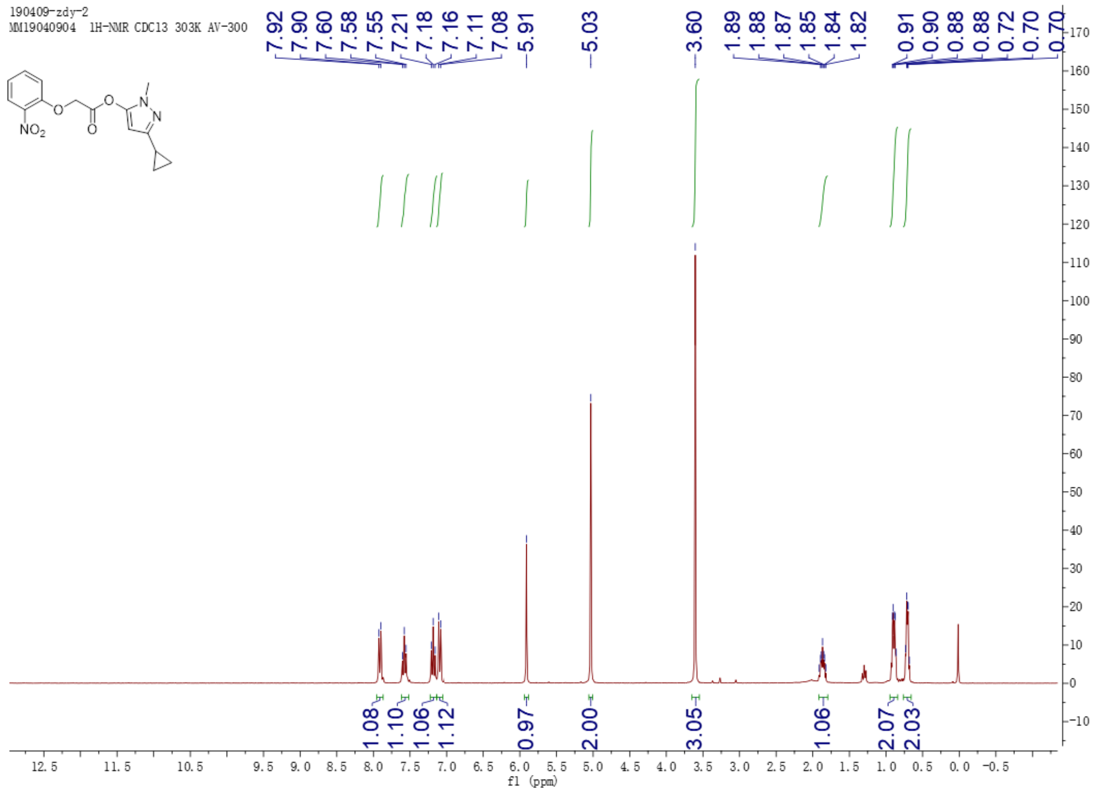

1-Methyl-3-(trifluoromethyl)-1*H*-pyrazol-5-yl 2-(2-nitrophenoxy)acetate (E16): Yield, 62%; white solid; ^{1}H NMR (300 MHz, $CDCl_3$) δ 7.93 (d, J = 7.8 Hz, 1H), 7.61 (t, J = 7.7 Hz, 1H), 7.22 (t, J = 7.7 Hz, 1H), 7.12 (d, J = 8.3 Hz, 1H), 6.56 (s, 1H), 5.08 (s, 2H), 3.78 (s, 3H). MS (ESI) m/z 368.3 [M+Na]$^+$.

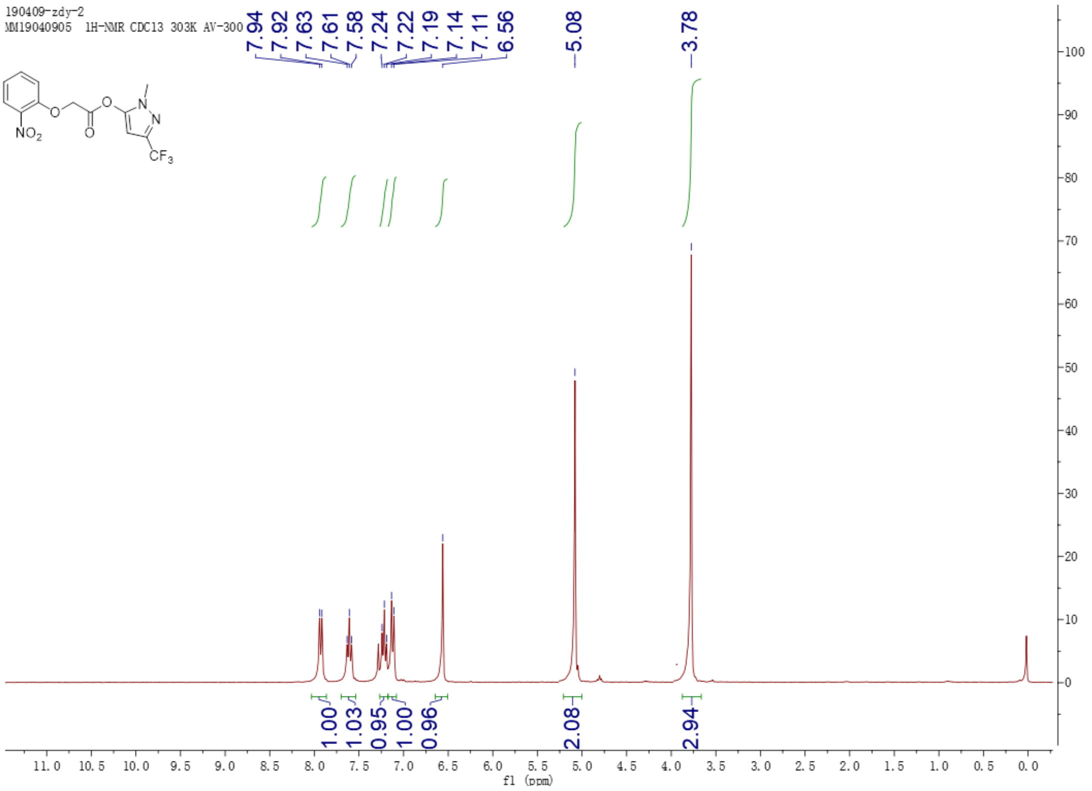

3-Oxocyclohex-1-en-1-yl 2-(4-nitrophenoxy)acetate (E17):

Yield, 60%; white solid; ^{1}H NMR (300 MHz, $CDCl_3$) δ 8.24 (d, J = 9.2 Hz, 2H), 7.03 (d, J = 9.2 Hz, 2H), 5.99 (s, 1H), 4.92 (s, 2H), 2.61-2.57(m,2H), 2.49–2.31(m,2H), 2.15-2.08(m,2H). MS (ESI) m/z 314.1 $[M+Na]^+$.

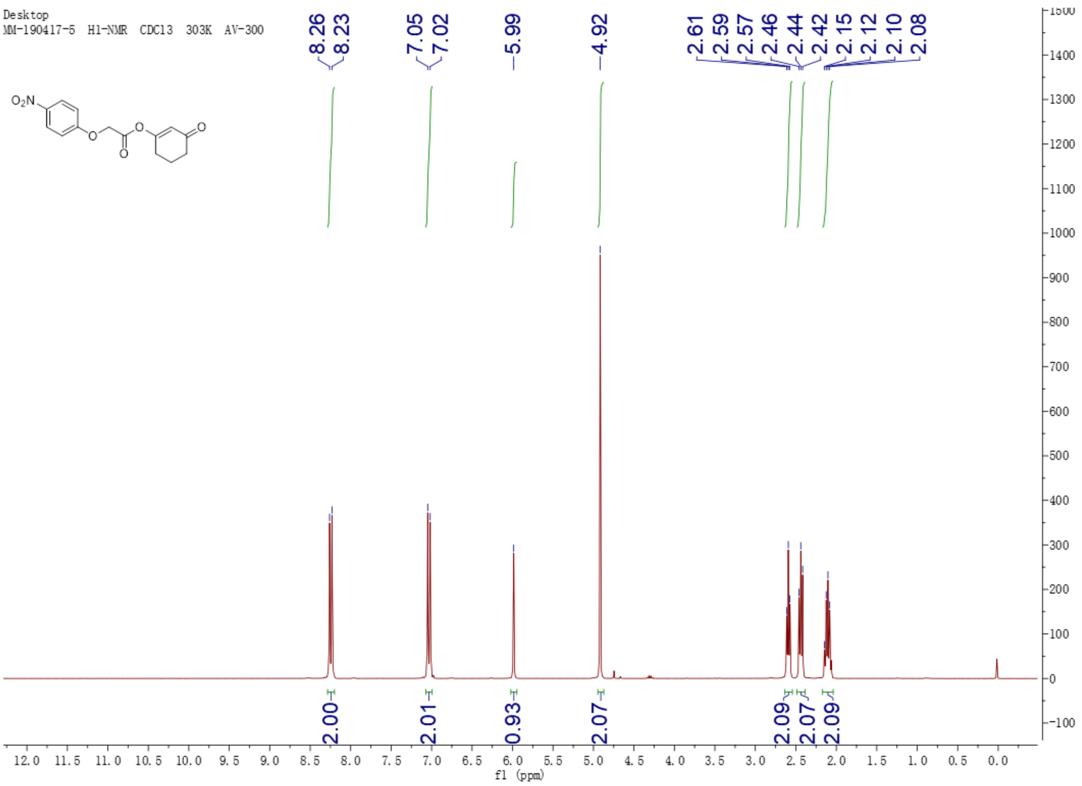

5,5-Dimethyl-3-oxocyclohex-1-en-1-yl 2-(4-nitrophenoxy)acetate (E18):

Yield, 58%; white solid; ^{1}H NMR (300 MHz, $CDCl_3$) δ 8.27 (d, J = 9.2 Hz, 1H), 7.04 (d, J = 9.2 Hz, 1H), 6.00 (s, 1H), 4.91 (s, 1H), 2.48 (s, 1H), 2.32 (s, 1H), 1.15 (s, 3H). MS (ESI) m/z 318.2 [M−H]$^-$.

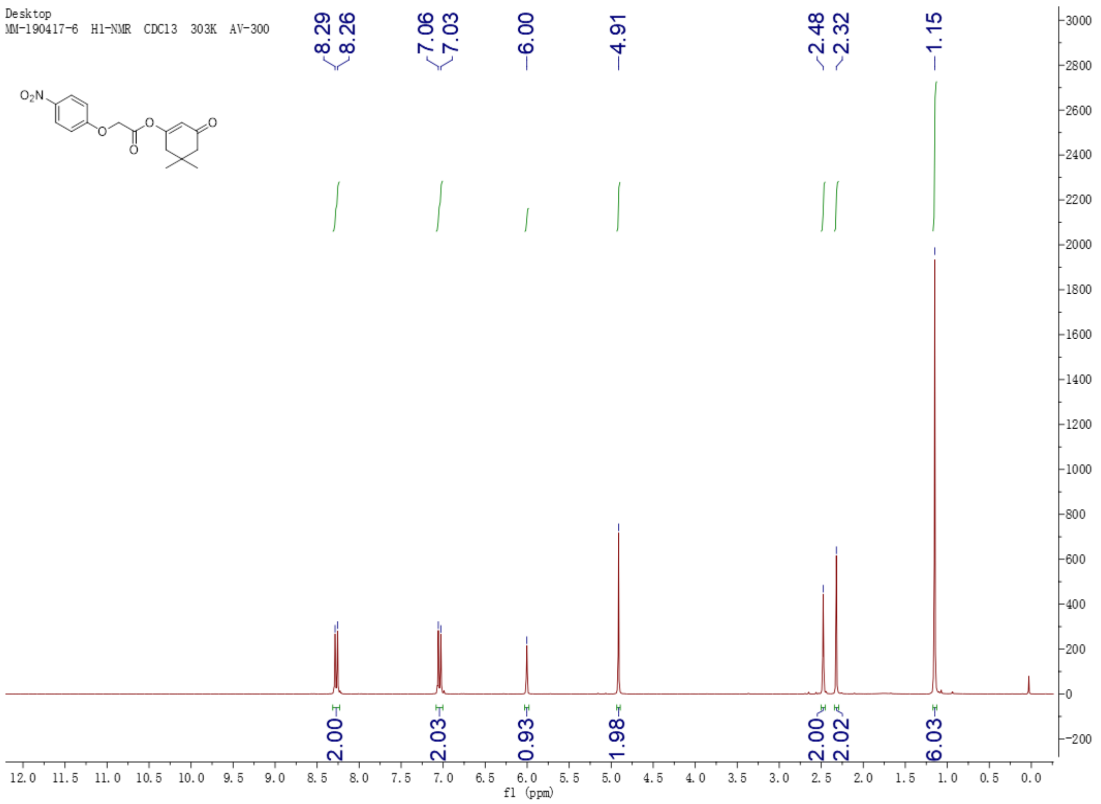

1,3-Dimethyl-1*H*-pyrazol-5-yl 2-(4-nitrophenoxy)acetate (E19):

Yield, 60%; white solid; ^{1}H NMR (300 MHz, $CDCl_3$) δ 8.27 (d, J = 9.2 Hz, 1H), 7.04 (d, J = 9.2 Hz, 1H), 6.00 (s, 1H), 4.91 (s, 1H), 2.48 (s, 1H), 2.32 (s, 1H), 1.15 (s, 3H). MS (ESI) m/z 292.1 [M+H] $^+$.

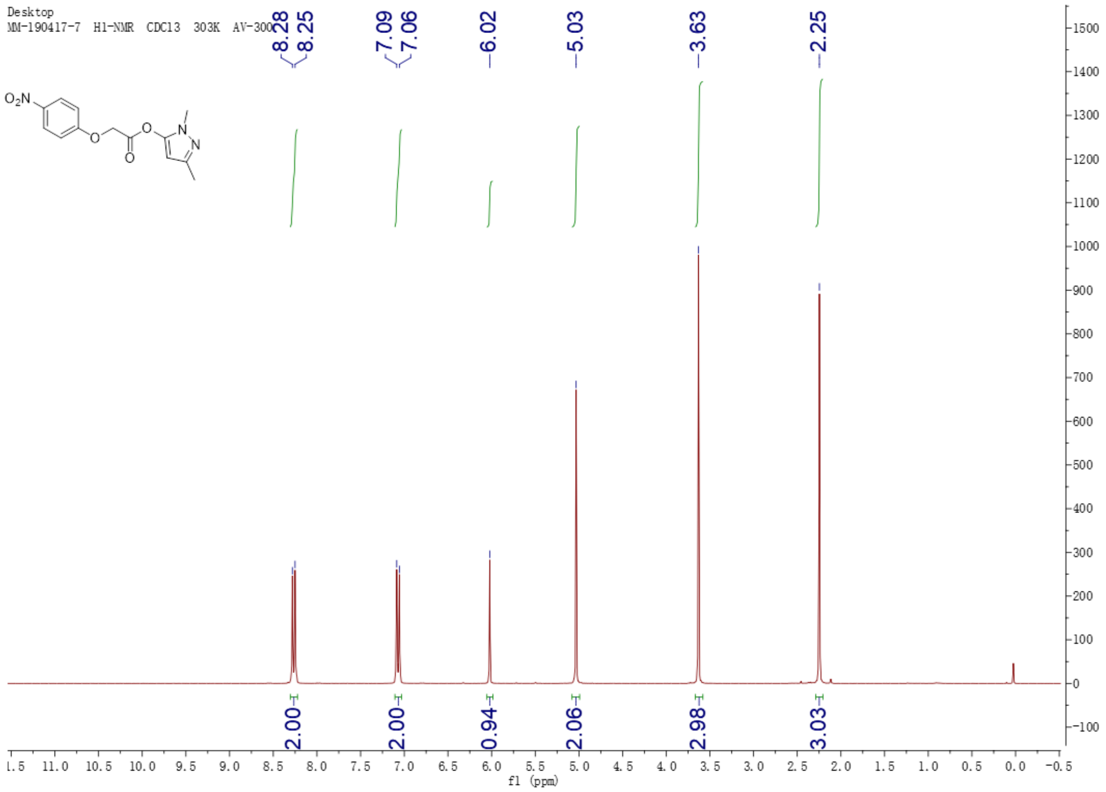

3-Cyclopropyl-1-methyl-1*H*-pyrazol-5-yl 2-(4-nitrophenoxy)acetate (E20):

Yield, 59%; white solid; ^{1}H NMR (300 MHz, $CDCl_3$) δ 8.26 (d, J = 9.2 Hz, 2H), 7.06 (d, J = 9.2 Hz, 2H), 5.90 (s, 1H), 5.02 (s, 2H), 3.61 (s, 3H), 2.02 – 1.62 (m, 1H), 0.99 – 0.78 (m, 2H), 0.78 – 0.65 (m, 2H). MS (ESI) *m/z* 318.2 [M+H] $^{+}$.

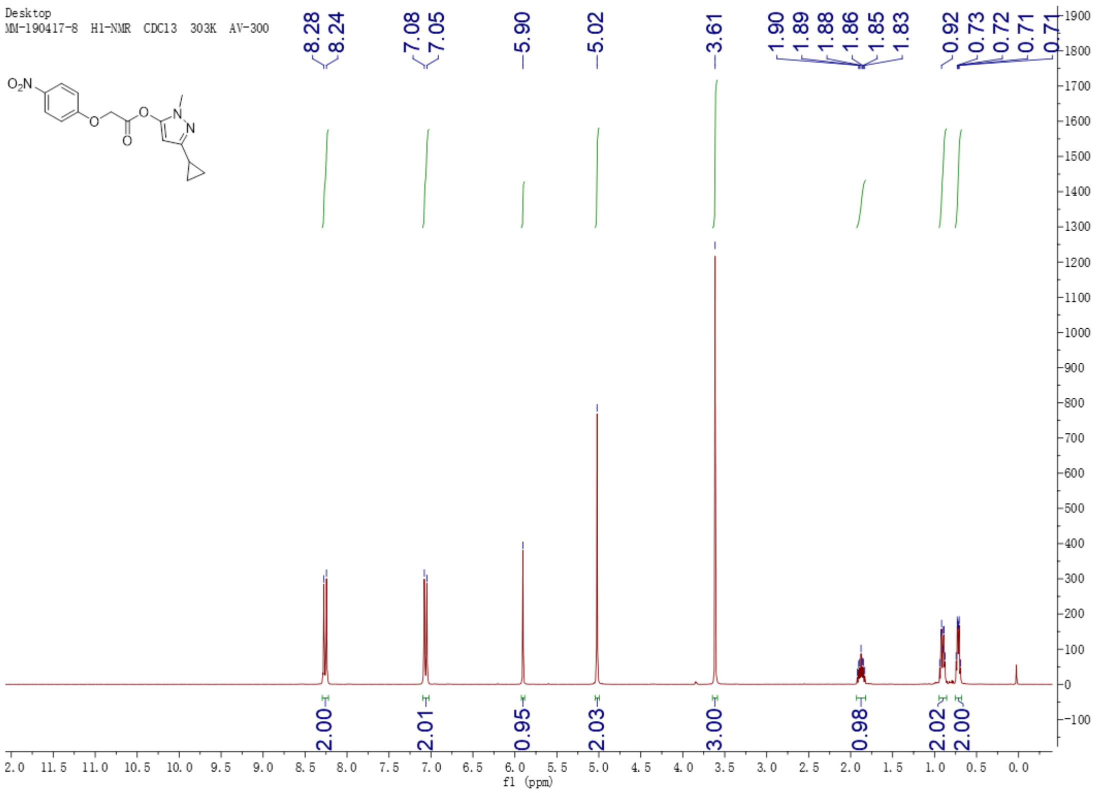

1-Methyl-3-(trifluoromethyl)-1*H*-pyrazol-5-yl 2-(4-nitrophenoxy)acetate (E21):

Yield, 69%; white solid; ^{1}H NMR (300 MHz, $CDCl_3$) δ 8.27 (d, J = 9.2 Hz, 2H), 7.09 (d, J = 9.2 Hz, 2H), 6.56 (s, 1H), 5.08 (s, 2H), 3.78 (s, 3H). MS (ESI) *m/z* 318.1 [M+H] $^{+}$.

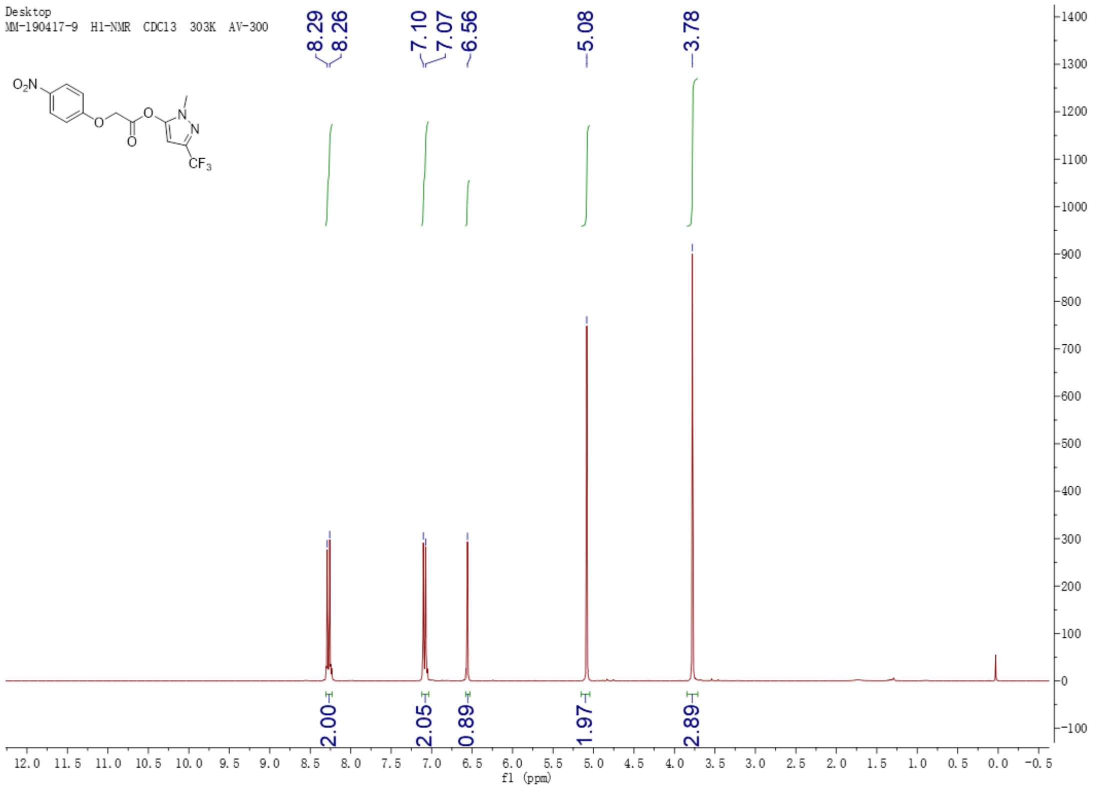

5,5-Dimethyl-3-oxocyclohex-1-en-1-yl 2-(2-chloro-4-nitrophenoxy)acetate (E22): Yield, 65%; white solid; ^{1}H NMR (300 MHz, $CDCl_3$) δ 8.33 (d, J = 2.5 Hz, 1H), 8.16 (dd, J = 9.1, 2.6 Hz, 1H), 6.98 (d, J = 9.1 Hz, 1H), 5.98 (s, 1H), 5.00 (s, 2H), 2.46 (s, 2H), 2.29 (s, 2H), 1.12 (s, 6H). MS (ESI) m/z 354.4 $[M+H]^+$.

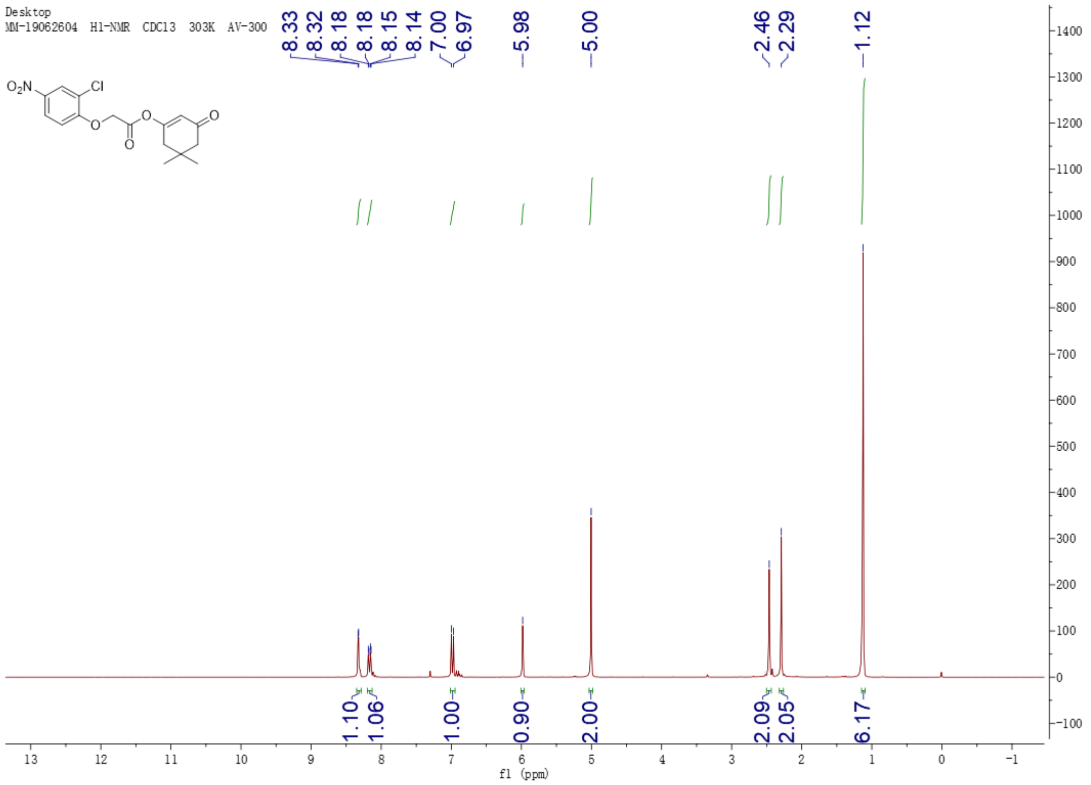

1,3-Dimethyl-1*H*-pyrazol-5-yl 2-(2-chloro-4-nitrophenoxy)acetate (E23):
Yield, 60%; white solid; ^{1}H NMR (300 MHz, $CDCl_3$) δ 8.37 (d, J = 2.5 Hz, 1H), 8.19 (dd, J = 9.1, 2.6 Hz, 1H), 7.02 (d, J = 9.1 Hz, 1H), 6.04 (s, 1H), 5.11 (s, 2H), 3.66 (s, 3H), 2.24 (s, 3H). MS (ESI) m/z 326.1 $[M+H]^+$.

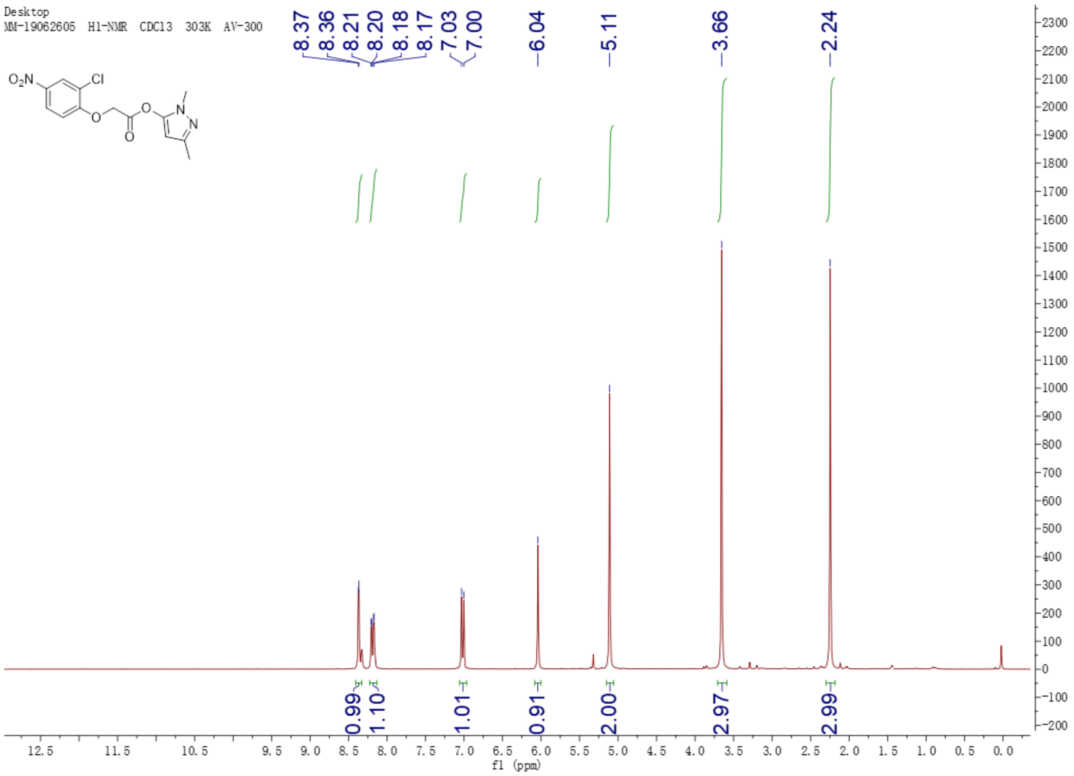

3-Cyclopropyl-1-methyl-1*H*-pyrazol-5-yl 2-(2-chloro-4-nitrophenoxy)acetate (E24):

Yield, 64%; yellow solid; ^{1}H NMR (300 MHz, DMSO) δ 8.34 (d, J = 2.7 Hz, 1H), 8.17 (dd, J = 9.1, 2.7 Hz, 1H), 7.01 (d, J = 9.1 Hz, 1H), 5.91 (s, 1H), 5.10 (s, 2H), 3.63 (s, 3H), 1.86 (m, 1H), 0.97 – 0.85 (m, 2H), 0.78 – 0.64 (m, 2H). MS (ESI) *m/z* 350.1 [M+H] $^+$.

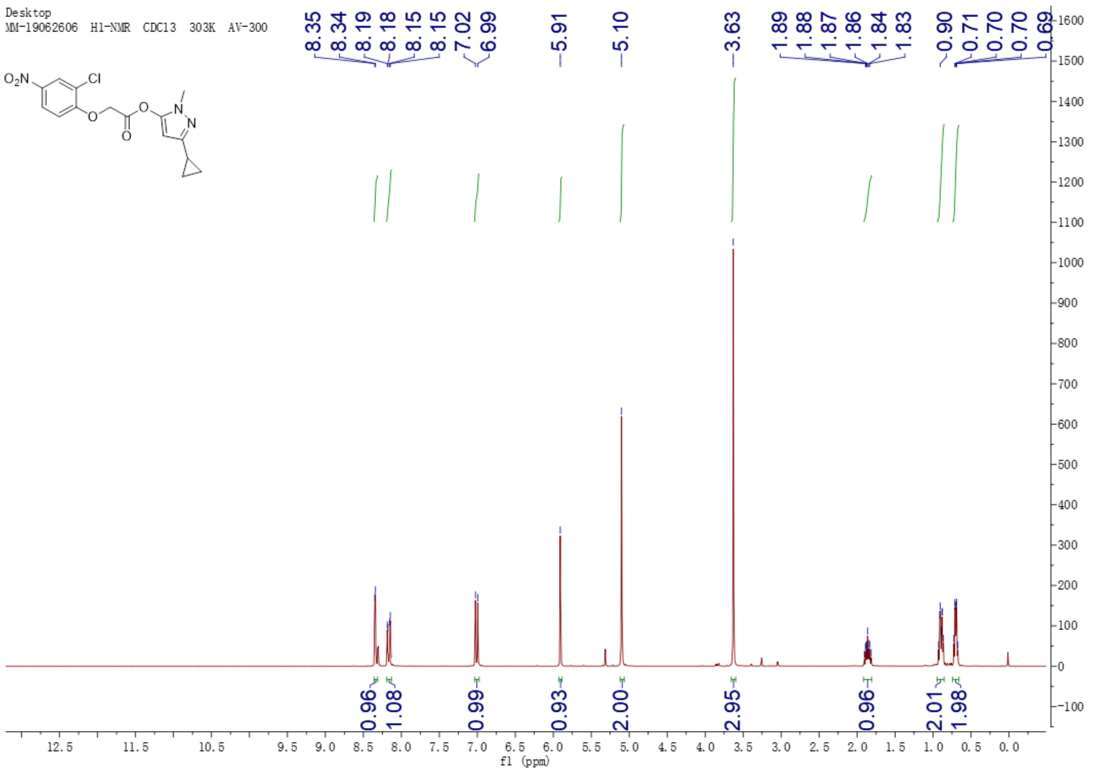

1-Methyl-3-(trifluoromethyl)-1*H*-pyrazol-5-yl 2-(2-chloro-4-nitrophenoxy)acetate (E25):

Yield, 68%; yellow solid; ^{1}H NMR (300 MHz, $CDCl_3$) δ 7.35 (s, 1H), 7.12 (d, J = 2.4 Hz, 1H), 6.80 (d, J = 8.8 Hz, 1H), 6.44 (s, 1H), 4.89 (s, 2H), 3.65 (s, 3H). MS (ESI) *m/z* 380.3 [M+H] $^{+}$.

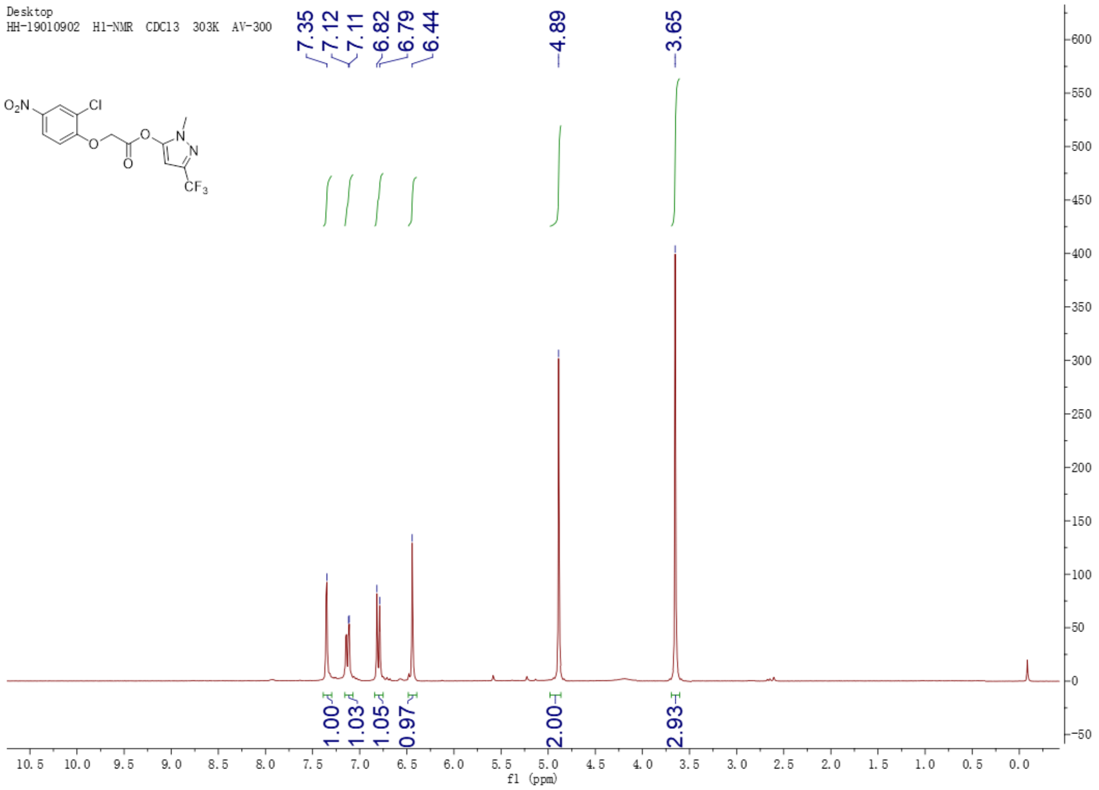

3-Oxocyclohex-1-en-1-yl 2-(5-chloro-2-(2,4-dichlorophenoxy)phenoxy)acetate (E26):

Yield, 60%; white solid; ^{1}H NMR (300 MHz, $CDCl_3$) δ 7.46 (d, J = 2.5 Hz, 1H), 7.17–7.15 (m, 1H), 7.07 – 6.99 (m, 2H), 6.91 (d, J = 8.8 Hz, 1H), 6.79 (d, J = 8.8 Hz, 1H), 5.94 (s, 1H), 4.82 (s, 2H), 2.56-2.52 (m, 2H), 2.47 – 2.39 (m, 2H), 2.14 – 2.03 (m, 2H). MS (ESI) m/z 441.0 $[M+H]^+$.

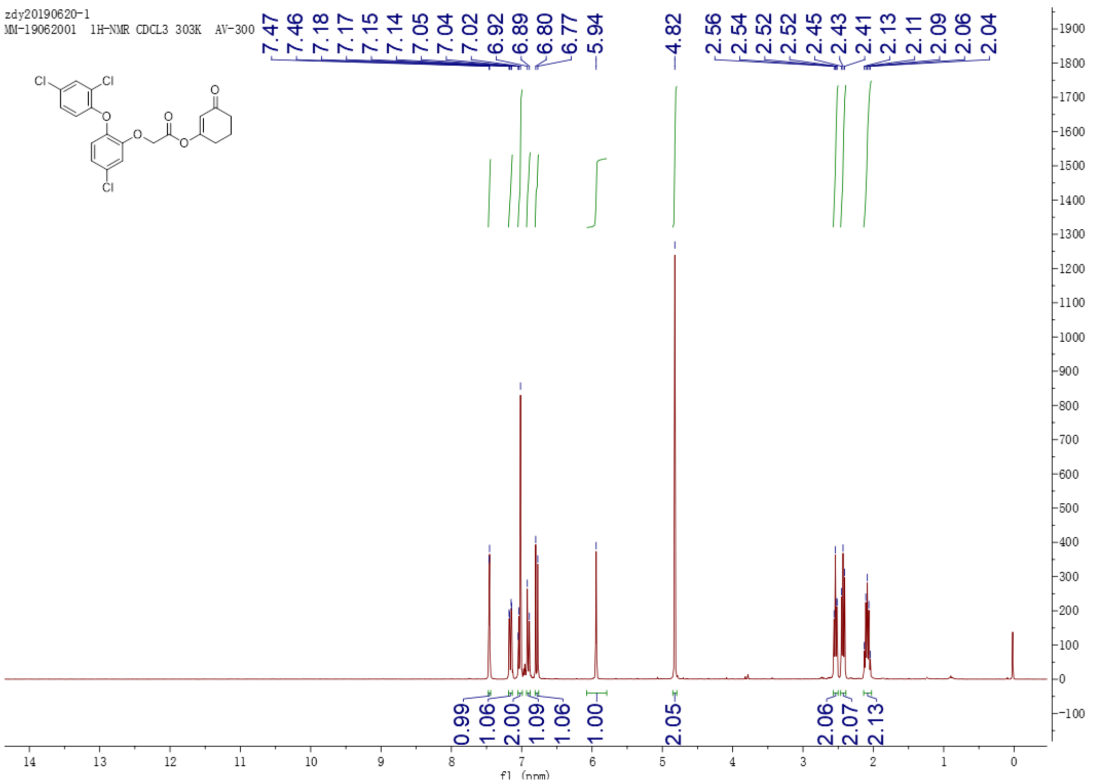

1,3-Dimethyl-1*H*-pyrazol-5-yl 2-(5-chloro-2-(2,4-dichlorophenoxy)phenoxy)acetate (E28):

Yield, 61%; yellow solid; ^{1}H NMR (300 MHz, $CDCl_3$) δ 7.46 (d, J = 2.5 Hz, 1H), 7.18-7.07 (m, 1H), 7.08 – 7.00 (m, 2H), 6.90 (d, J = 8.8 Hz, 1H), 6.80 (d, J = 8.8 Hz, 1H), 5.96 (s, 1H), 4.95 (s, 2H), 3.59 (s, 3H), 2.24 (s, 3H). MS (ESI) *m/z* 441.3 $[M+H]^{+}$.

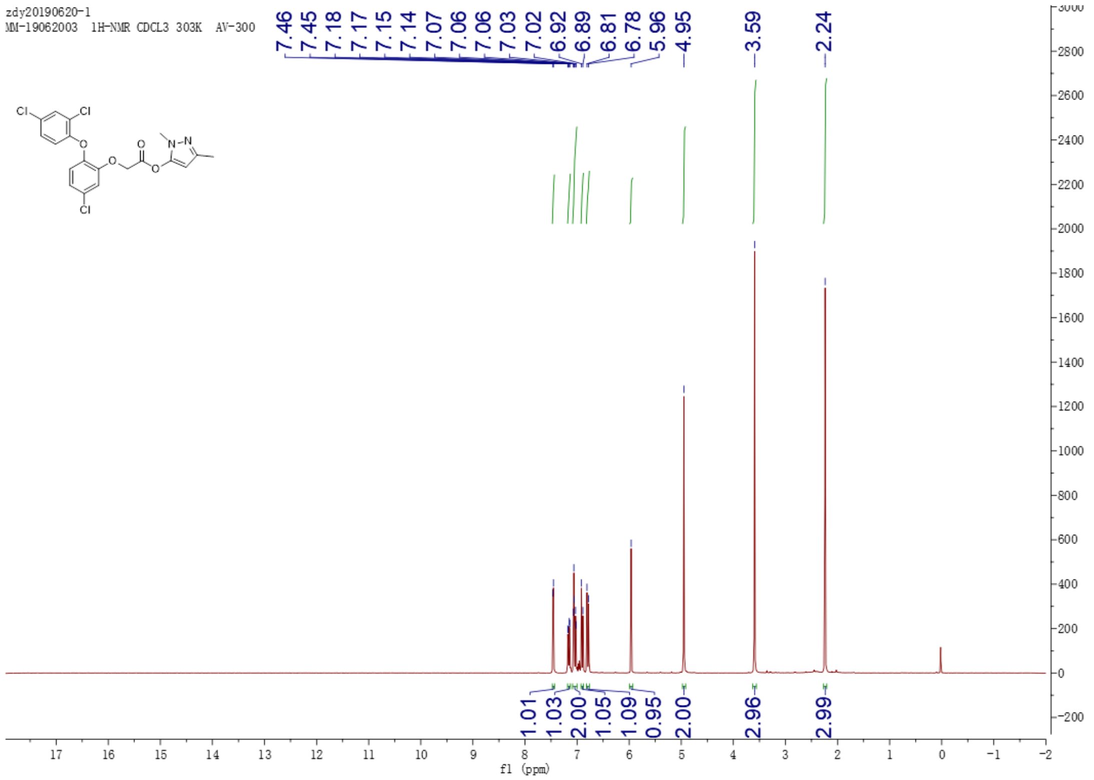

3-Cyclopropyl-1-methyl-1*H*-pyrazol-5-yl 2-(5-chloro-2-(2,4-dichlorophenoxy)phenoxy)acetate (E29):

Yield, 60%; yellow solid; ^{1}H NMR (300 MHz, $CDCl_3$) δ 7.46 (d, J = 2.4 Hz, 1H), 7.18-7.07 (m, 1H), 7.08 – 7.00 (m, 2H), 6.90 (d, J = 8.8 Hz, 1H), 6.79 (d, J = 8.8 Hz, 1H),4.94 (s, 2H), 3.58 (s, 3H), 1.96 – 1.79 (m, 1H), 0.98 – 0.84 (m, 2H), 0.79 – 0.61 (m, 2H). MS (ESI) m/z 467.1 $[M+H]^+$.

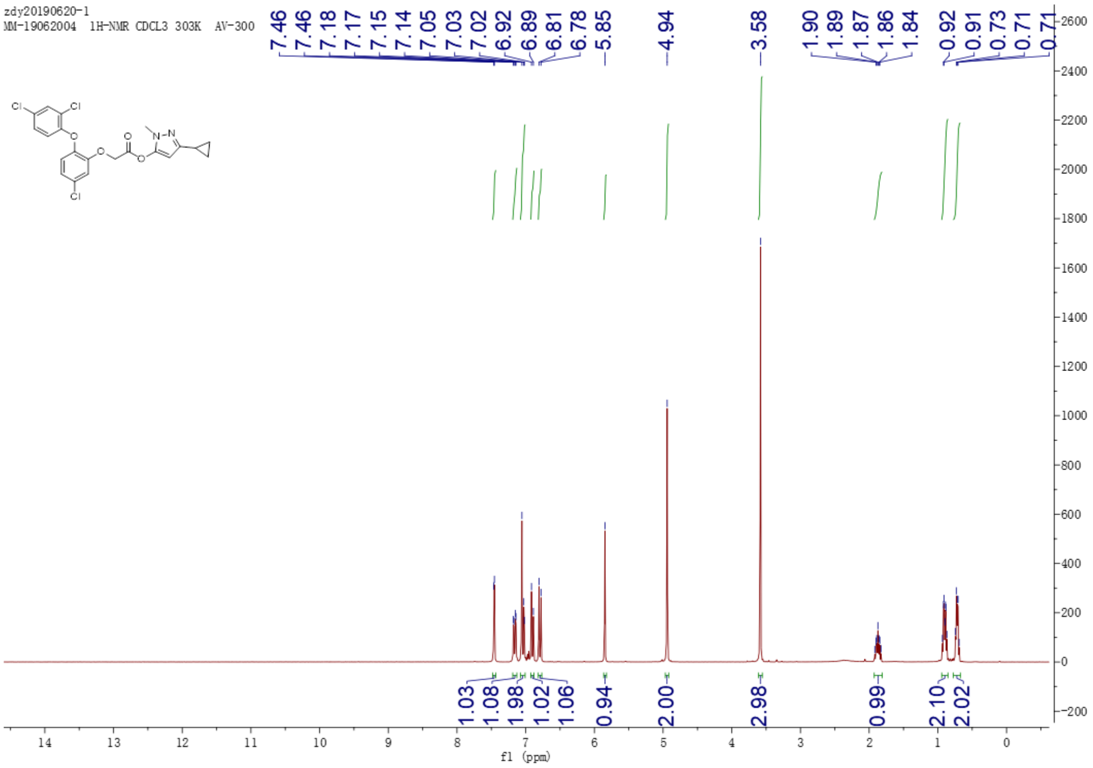

3-Oxocyclohex-1-en-1-yl 2-(2,4-dimethylphenoxy)acetate (E30):

Yield, 58%; white solid; ^{1}H NMR (300 MHz, $CDCl_3$) δ 7.01 (s, 1H), 6.96 (d, J = 8.3 Hz, 1H), 6.65 (d, J = 8.2 Hz, 1H), 5.98 (s, 1H), 4.77 (s, 2H), 2.57 (dd, J = 8.9, 3.5 Hz, 2H), 2.48 – 2.38 (m, 2H), 2.28 (s, 6H), 2.17 – 2.01 (m, 2H). MS (ESI) m/z 297.3 $[M+Na]^+$.

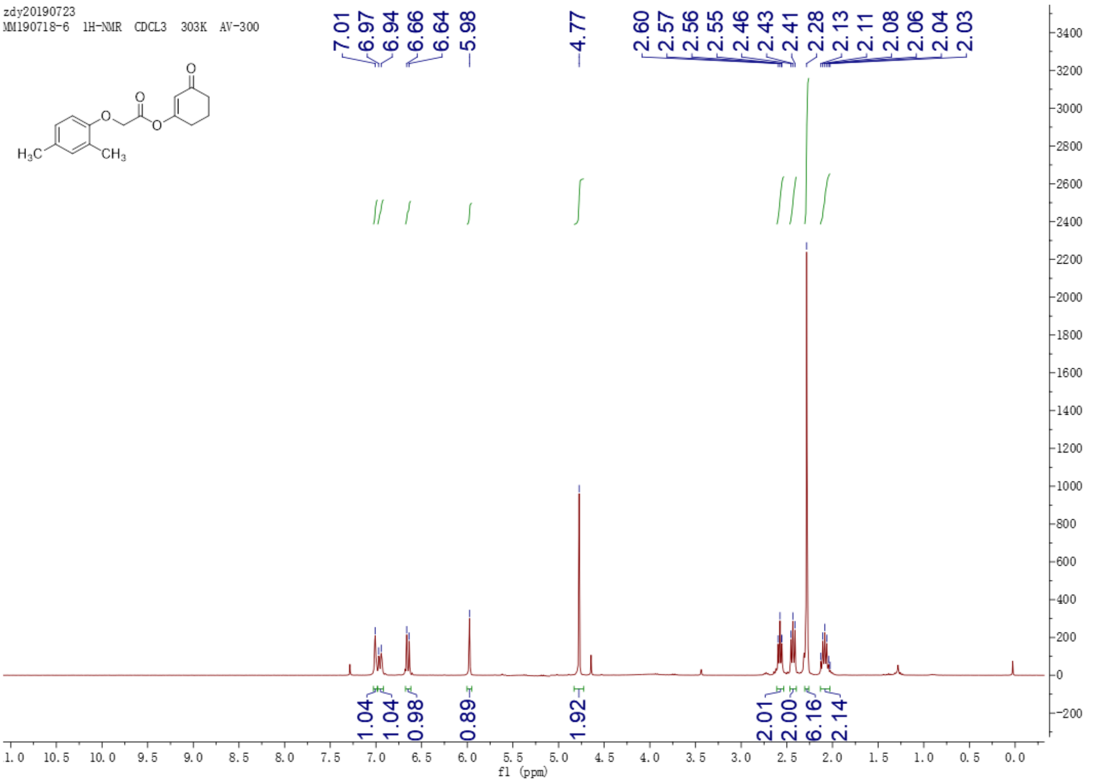

1,3-Dimethyl-1*H*-pyrazol-5-yl 2-(2,4-dimethylphenoxy)acetate (E32):

Yield, 64%; white solid; ^{1}H NMR (300 MHz, $CDCl_3$) δ 7.02 (s, 1H), 6.96 (d, J = 8.3 Hz, 1H), 6.68 (d, J = 8.2 Hz, 1H), 6.00 (s, 1H), 4.88 (s, 2H), 3.56 (s, 3H), 2.29 (d, J = 5.7 Hz, 6H), 2.24 (s, 3H). MS (ESI) *m/z* 297.3 [M+Na] $^+$.

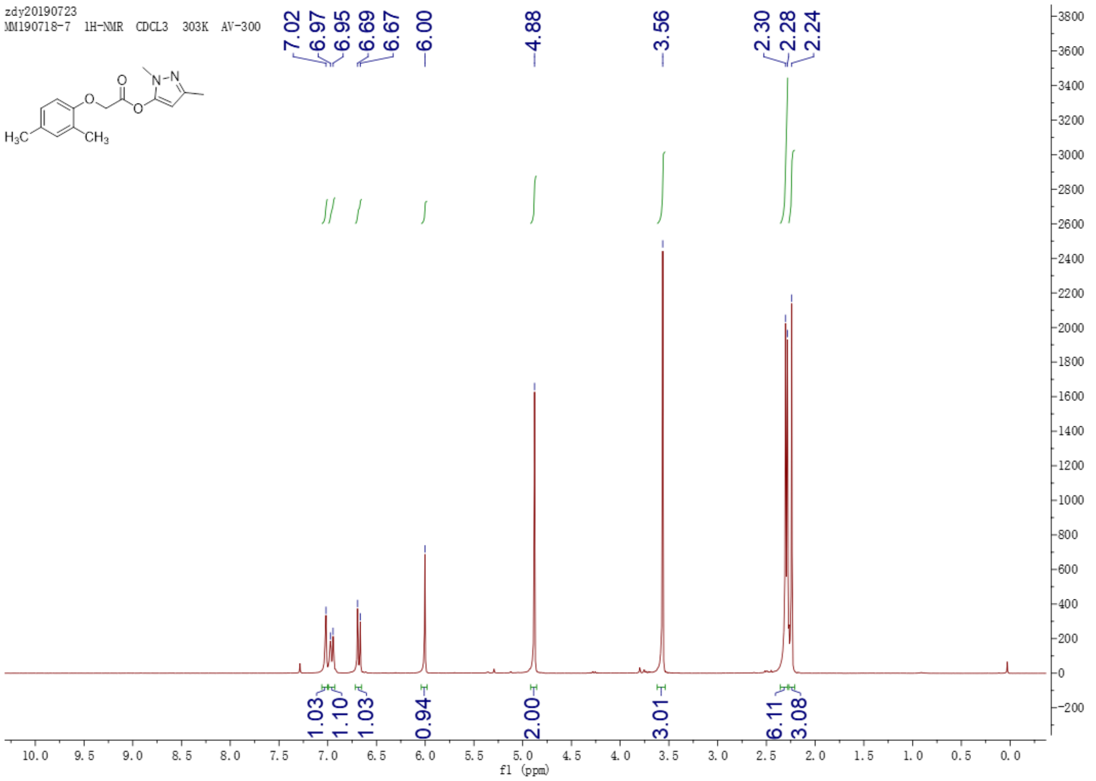

3-Cyclopropyl-1-methyl-1*H*-pyrazol-5-yl 2-(2,4-dimethylphenoxy)acetate (E33):

Yield, 54%; white solid; ^{1}H NMR (300 MHz, $CDCl_3$) δ 7.02 (s, 1H), 6.96 (d, J = 8.3 Hz, 1H), 6.68 (d, J = 8.2 Hz, 1H), 5.89 (s, 1H), 4.88 (s, 2H), 3.56 (s, 3H), 2.30 (s, 3H), 2.29 (s, 3H), 1.99 – 1.62 (m, 1H), 0.98 – 0.81 (m, 2H), 0.79 – 0.67 (m, 2H). MS (ESI) *m/z* 275.3 [M+H] $^+$.

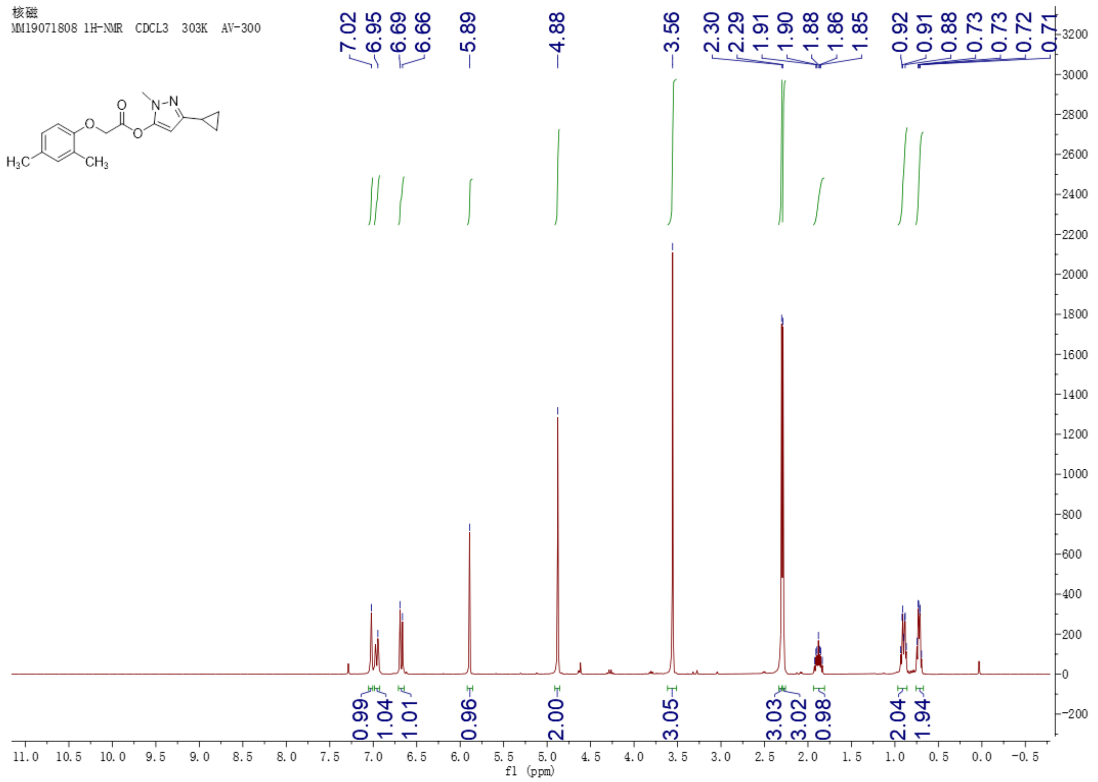

Characterization of selected intermediates F

3,5,6-Trichloropyridin-2-ol (F):

Yield, 87%; white solid; ^{1}H NMR (300 MHz, DMSO) δ 12.88 (s, 1H), 8.18 (s, 1H).

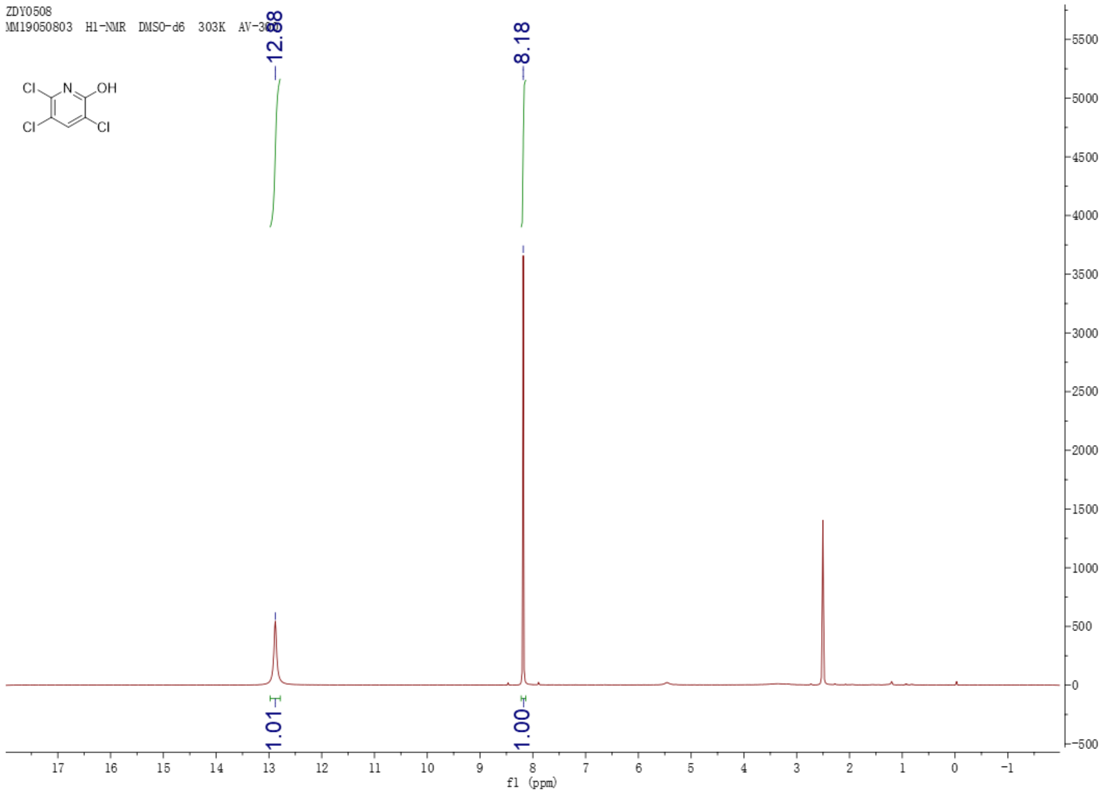

Characterization of selected intermediates G

Methyl 2-((3,5,6-trichloropyridin-2-yl)oxy)acetate (G):

Yield, 90%; white solid; ^{1}H NMR (300 MHz, $CDCl_3$) δ 7.79 (s, 1H), 4.99 (s, 2H), 3.82 (s, 3H).

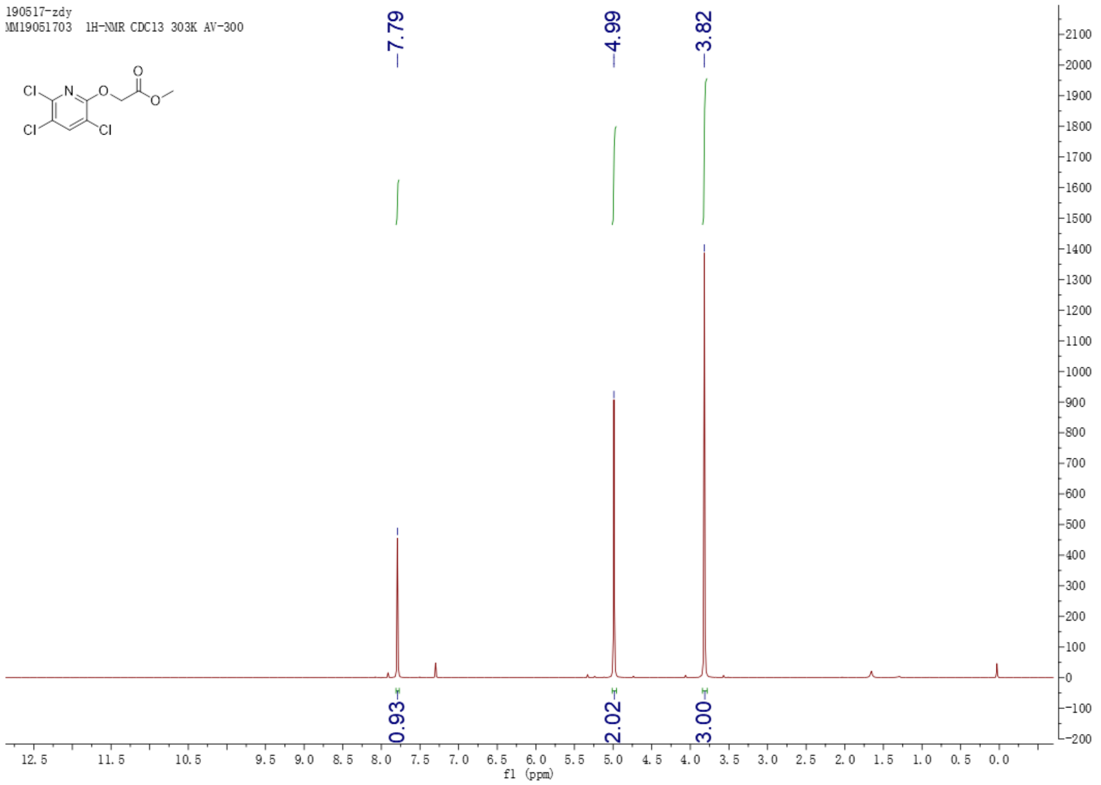

Characterization of selected intermediates H

2-((3,5,6-Trichloropyridin-2-yl)oxy)acetic acid (H):

Yield, 89%; white solid; ^{1}H NMR (300 MHz, DMSO) δ 8.35 (s, 1H), 4.79 (s, 2H).

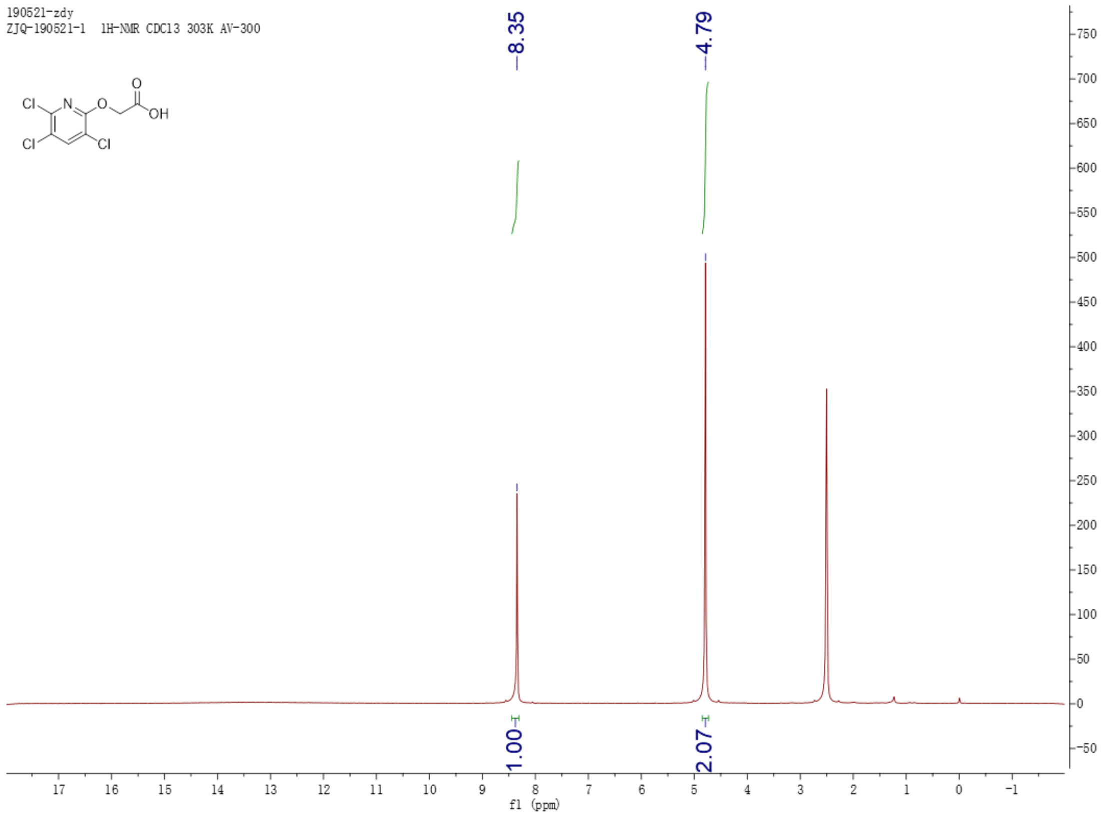

Characterization of selected intermediates J

1,3-Dimethyl-1*H*-pyrazol-5-yl 2-((3,5,6-trichloropyridin-2-yl)oxy)acetate (J3):

Yield, 85%; white solid; ^{1}H NMR (300 MHz, $CDCl_3$) δ 7.84 (s, 1H), 5.94 (s, 1H), 5.21 (s, 2H), 3.68 (s, 3H), 2.26 (s, 3H). MS (ESI) *m/z* 350.3 $[M+H]^+$.

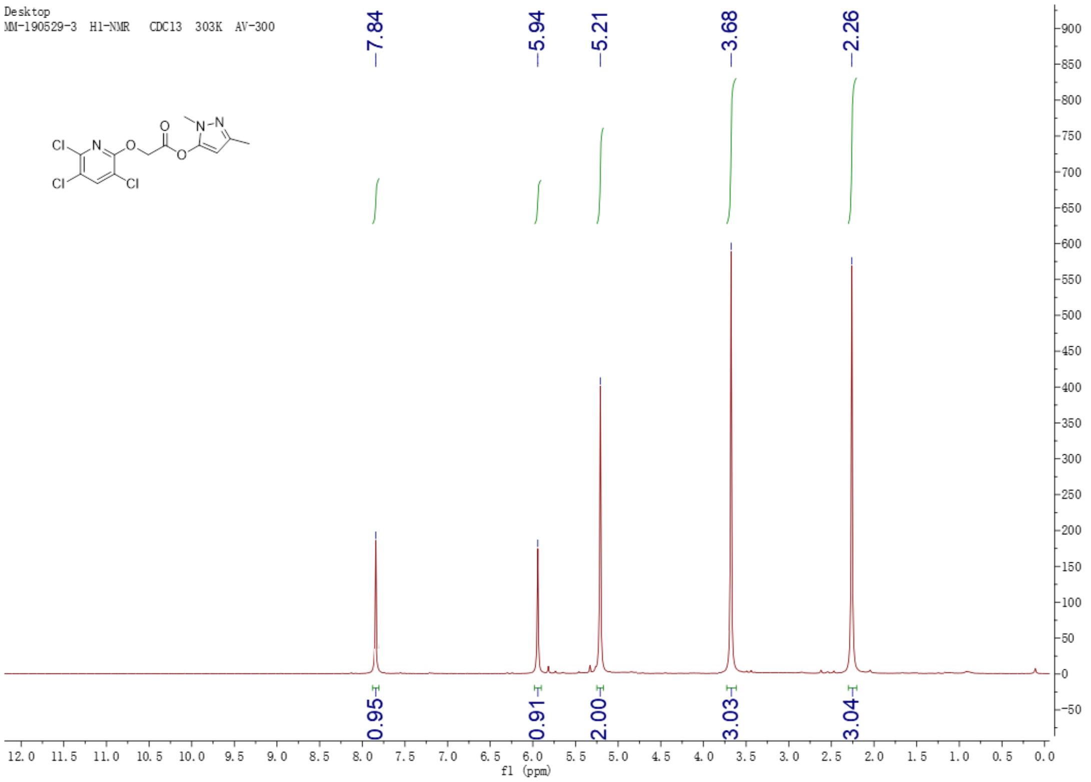

3-Cyclopropyl-1-methyl-1*H*-pyrazol-5-yl 2-((3,5,6-trichloropyridin-2-yl)oxy)acetate (J4):

Yield, 64%; white solid; ^{1}H NMR (300 MHz, $CDCl_3$) δ 7.83 (s, 1H), 5.82 (s, 1H), 5.19 (s, 2H), 3.65 (s, 3H), 2.16 – 1.69 (m, 1H), 1.01 – 0.76 (m, 2H), 0.79 – 0.54 (m, 2H). MS (ESI) *m/z* 376.0 [M+H] $^{+}$.

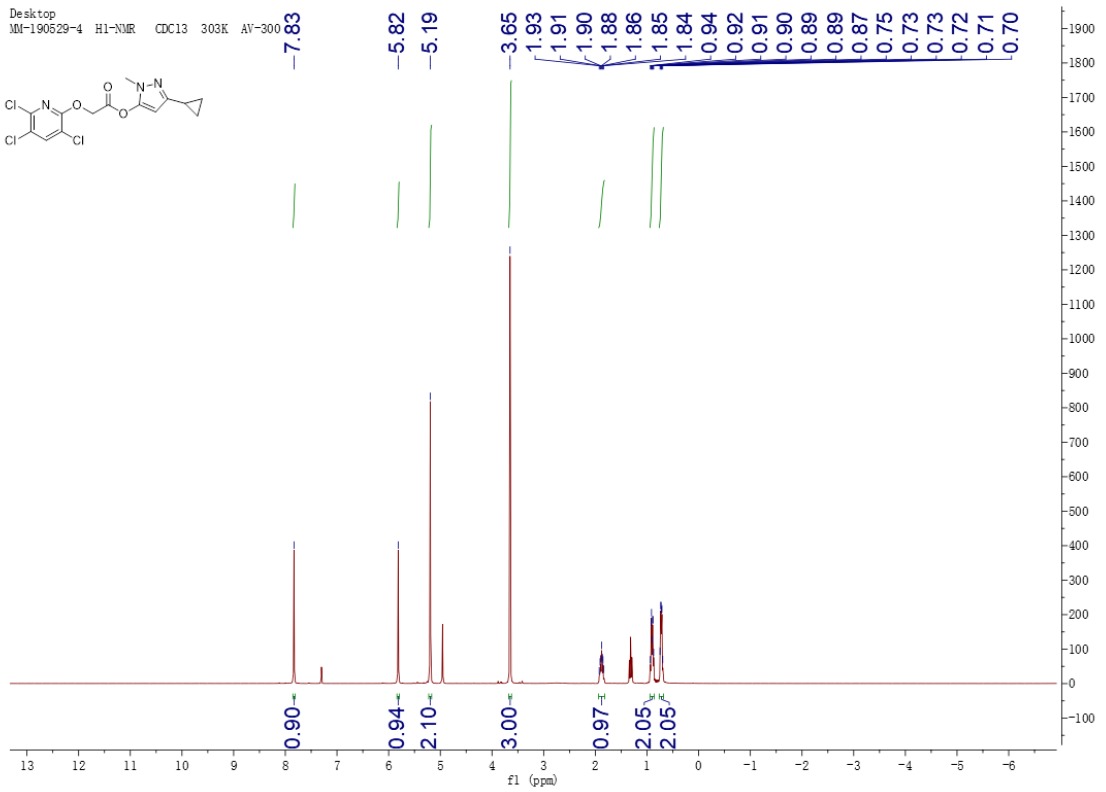

1-Methyl-3-(trifluoromethyl)-1*H*-pyrazol-5-yl 2-((3,5,6-trichloropyridin-2-yl)oxy)acetate (J5):

Yield, 57%; white solid; ^{1}H NMR (300 MHz, $CDCl_3$) δ 7.86 (s, 1H), 6.46 (s, 1H), 5.24 (s, 2H), 3.81 (s, 3H). MS (ESI) *m/z* 402.0 [M+H] $^{+}$.

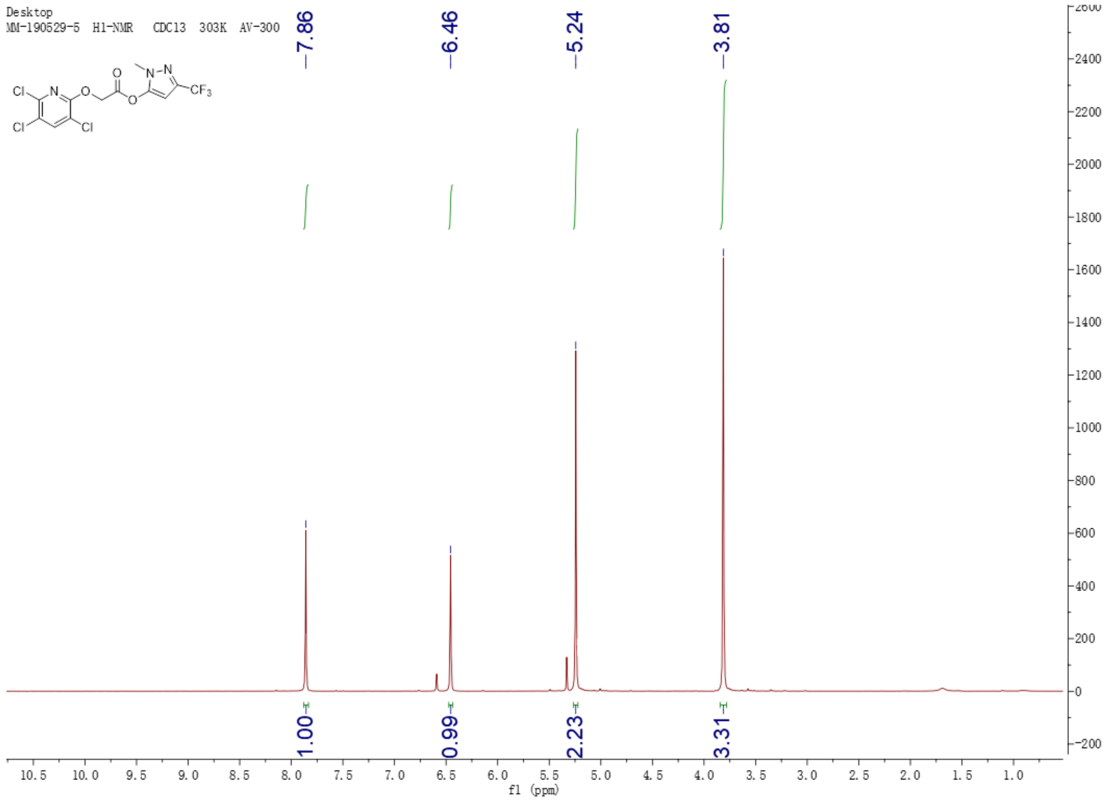

Characterization of selected intermediates L

2-((1-Bromonaphthalen-2-yl)oxy)acetic acid (L1–L4):

Yield, 95%; white solid; ^{1}H NMR (300 MHz, DMSO) δ 13.16 (s, 1H), 8.11 (d, J = 8.5 Hz, 1H), 8.00 – 7.86 (m, 2H), 7.62 (t, J = 7.6 Hz, 1H), 7.43 (dd, J = 17.3, 8.3 Hz, 2H), 4.98 (s, 2H).

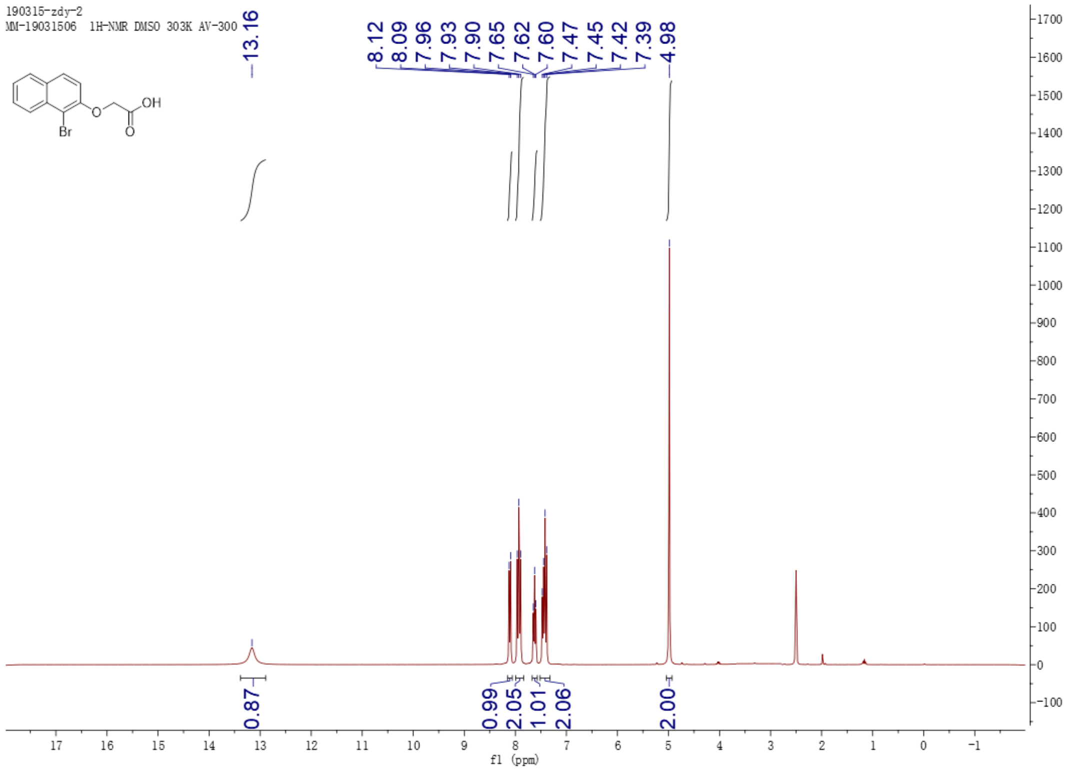

2-((2-Nitropyridin-3-yl)oxy)acetic acid (L5, L6):

Yield, 75%; white solid; ^{1}H NMR (300 MHz, DMSO) δ 13.15 (s, 1H), 8.11 (d, J = 3.8 Hz, 1H), 7.88 (d, J = 8.4 Hz, 1H), 7.72 (dd, J = 8.4, 3.8 Hz, 1H), 4.97 (s, 2H). ^{13}C NMR (75 MHz, DMSO) δ 168.89, 148.41, 145.42, 139.62, 129.04, 125.16, 65.27. MS (ESI) m/z 197.0 [M−H]$^{-}$.

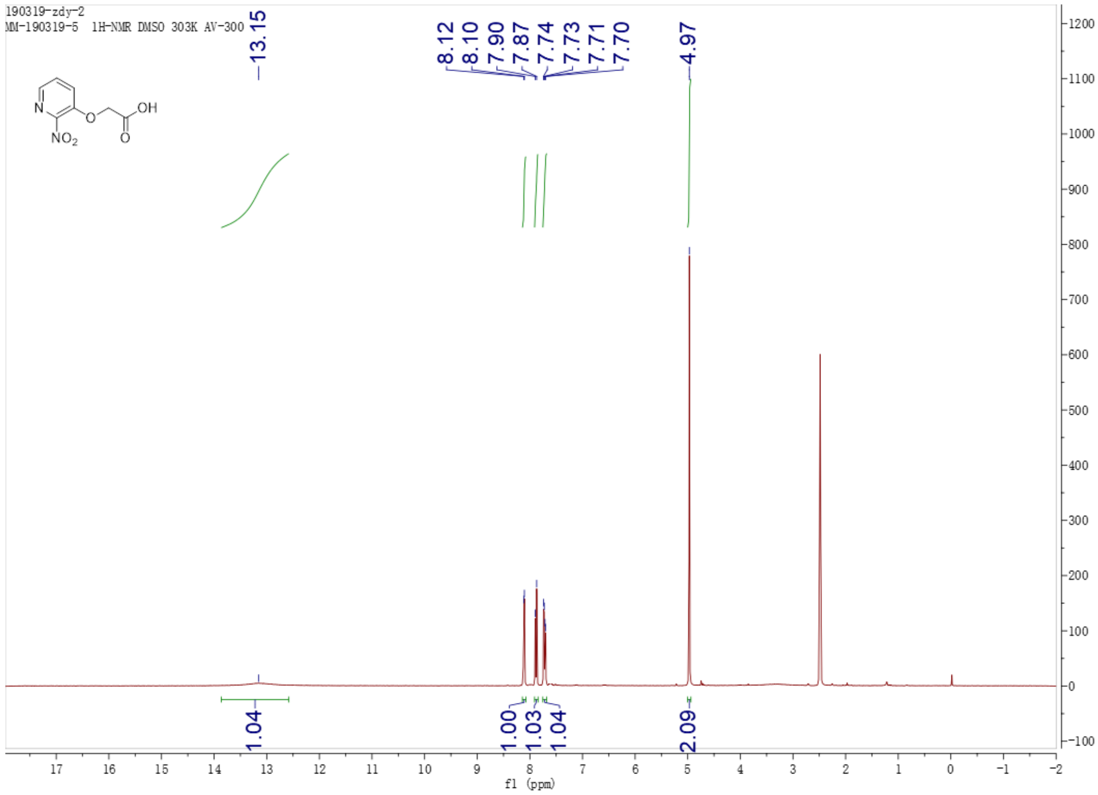

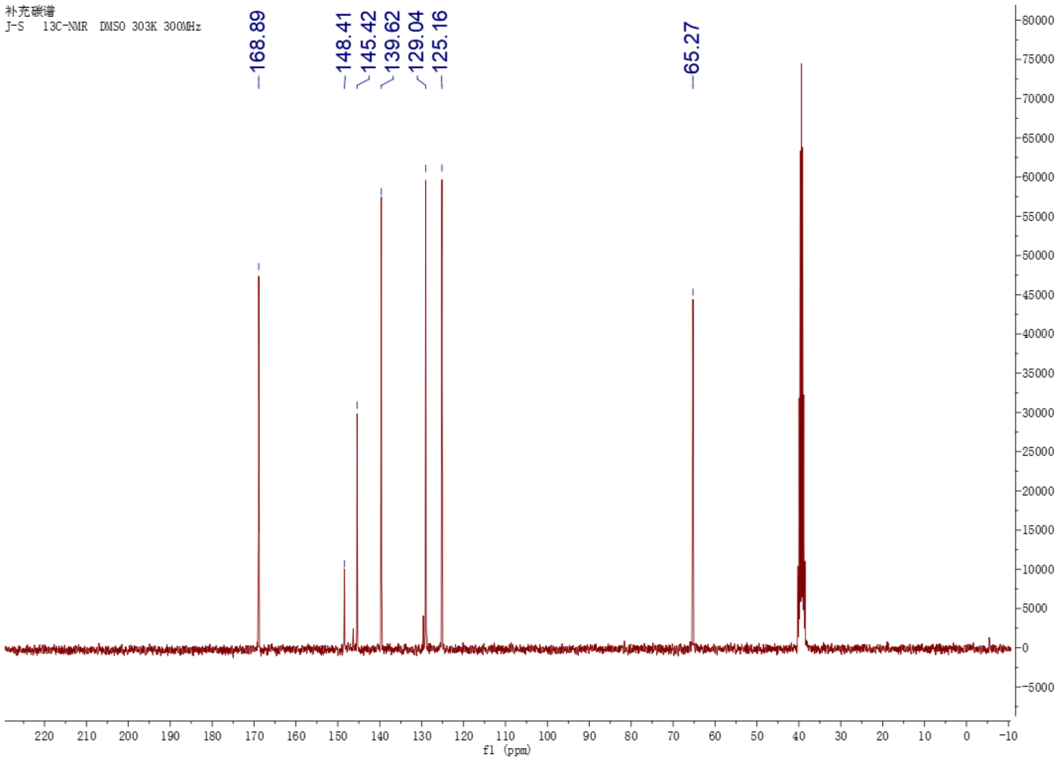

Characterization of selected intermediates M

1,3-Dimethyl-1*H*-pyrazol-5-yl 2-((1-bromonaphthalen-2-yl)oxy)acetate (M2):

Yield, 60%; white solid; ^{1}H NMR (300 MHz, $CDCl_3$) δ 8.29 (d, J = 8.5 Hz, 1H), 7.86-

7.82 (m, 2H), 7.64 (t, J = 7.5 Hz, 1H), 7.49 (t, J = 7.3 Hz, 1H), 7.27 (d, J = 9.3 Hz, 1H), 6.05 (s, 1H), 5.11 (s, 2H), 3.60 (s, 3H), 2.25 (s, 3H). MS (ESI) *m/z* 375.3 [M+H] $^+$.

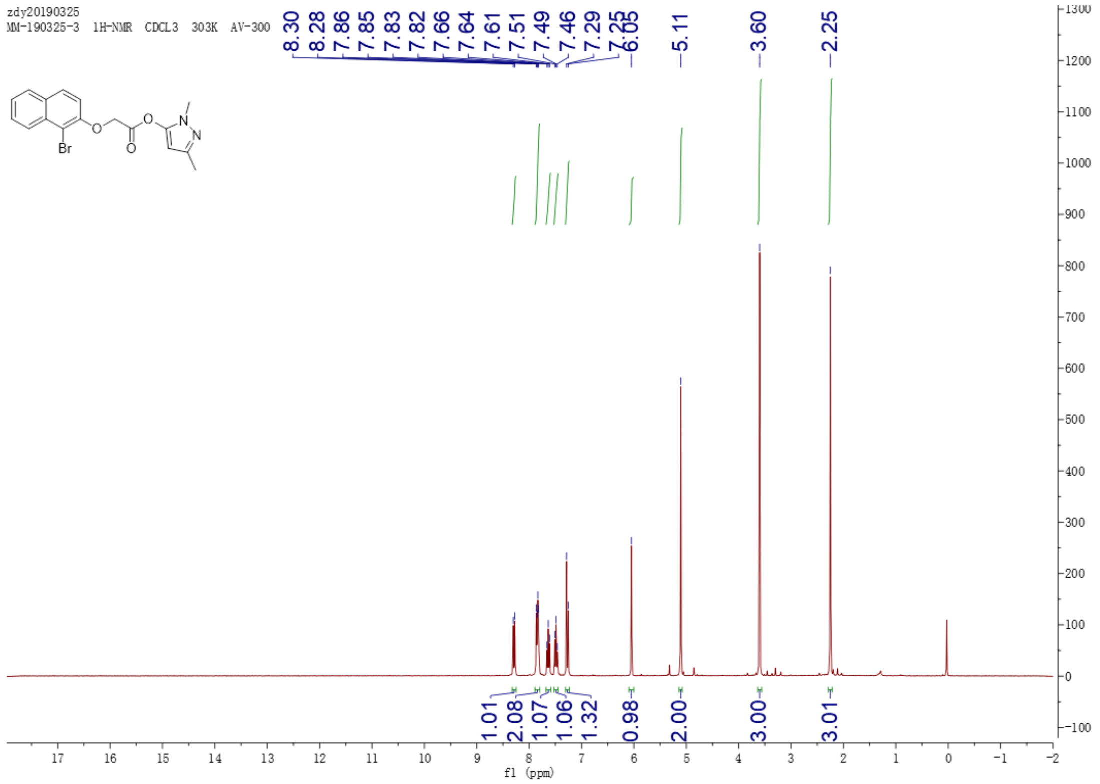

3-Cyclopropyl-1-methyl-1*H*-pyrazol-5-yl 2-((1-bromonaphthalen-2-yl)oxy)acetate (M3):

Yield, 55%; white solid; ^{1}H NMR (300 MHz, $CDCl_3$) δ 8.29 (d, J = 8.5 Hz, 1H), 7.85-7.82 (m, 2H), 7.63 (t, J = 7.7 Hz, 1H), 7.48 (t, J = 7.6 Hz, 1H), 7.25 (d, J = 9.0 Hz, 1H), 5.93 (s, 1H), 5.09 (s, 2H), 3.58 (s, 3H), 2.10 – 1.76 (m, 1H), 0.95 – 0.86 (m, 2H), 0.76 – 0.67 (m, 2H). MS (ESI) *m/z* 401.4 [M+H] $^+$.

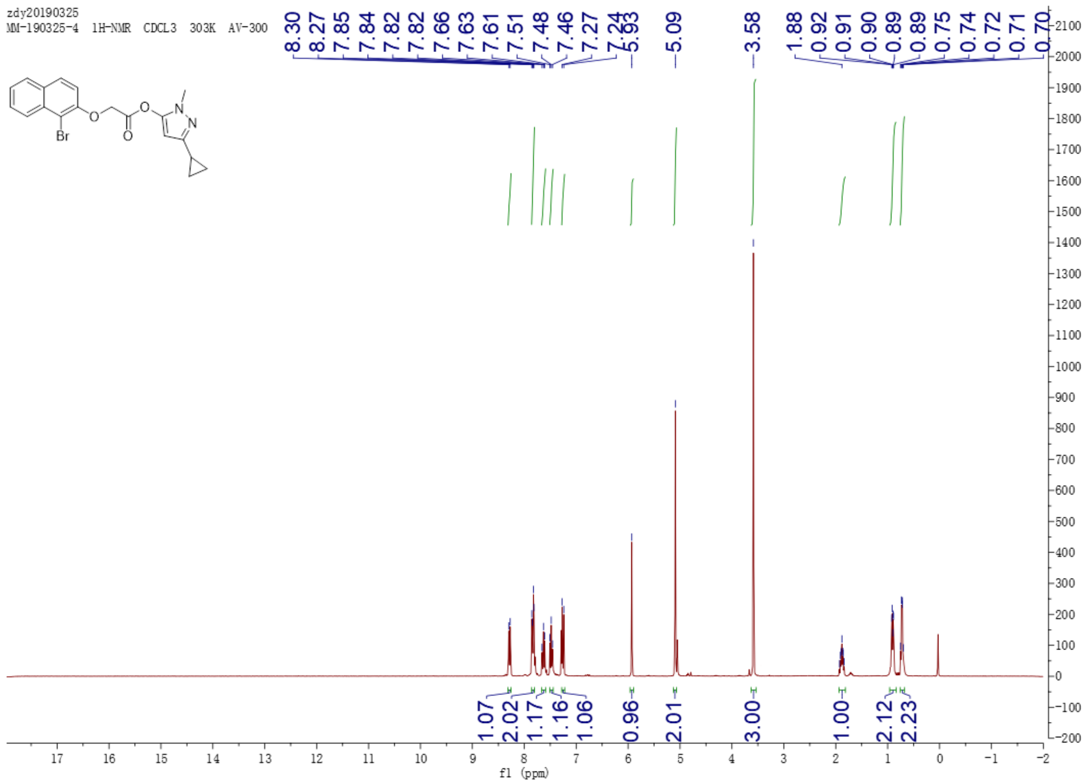

1-Methyl-3-(trifluoromethyl)-1*H*-pyrazol-5-yl-2-((1-bromonaphthalen-2-yl)oxy)acetate (M4):

Yield, 56%; white solid; ^{1}H NMR (300 MHz, $CDCl_3$) δ 8.29 (d, J = 8.6 Hz, 1H), 7.88-7.83 (m, 2H), 7.65 (t, J = 7.7 Hz, 1H), 7.50 (t, J = 7.5 Hz, 1H), 7.27 (d, J = 9.0 Hz, 1H), 6.58 (s, 1H), 5.14 (s, 2H), 3.73 (s, 3H). MS (ESI) m/z 429.3 $[M+H]^{+}$.

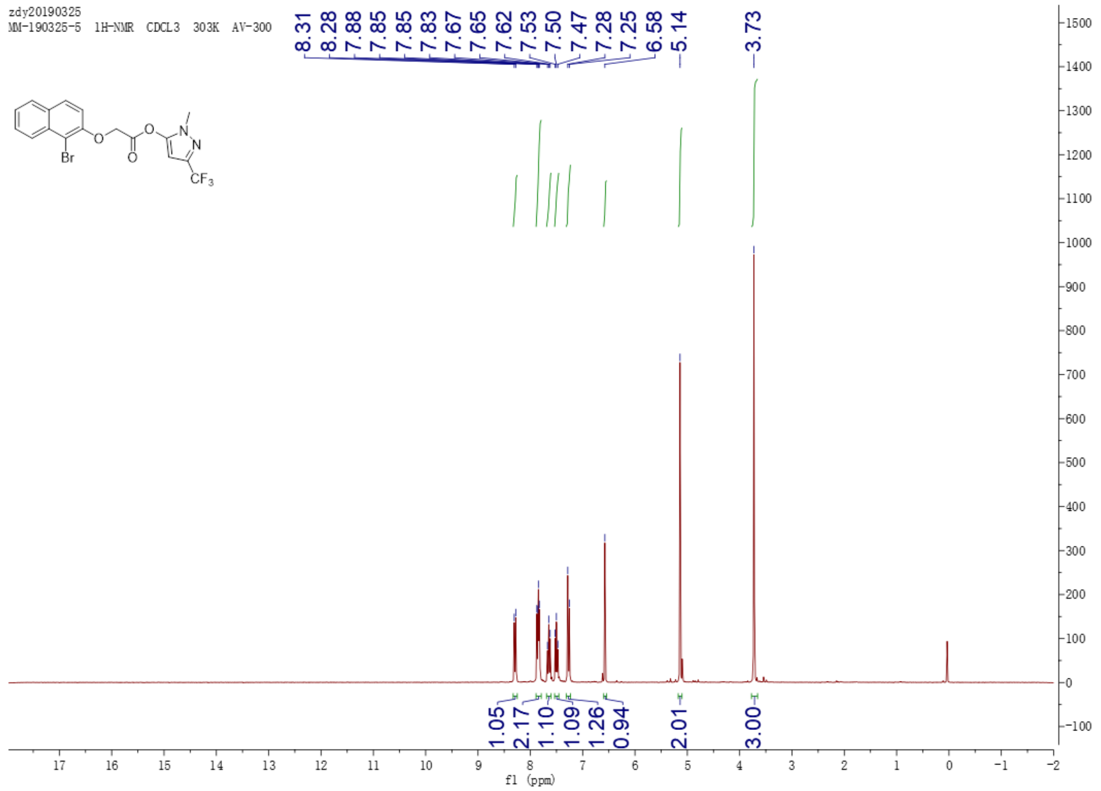

3-Cyclopropyl-1-methyl-1*H*-pyrazol-5-yl 2-((2-nitropyridin-3-yl)oxy)acetate (M6):

Yield, 65%; white solid; ^{1}H NMR (300 MHz, $CDCl_3$) δ 7.91 (d, J = 4.4 Hz, 1H), 7.59 (d, J = 8.5 Hz, 1H), 7.21 (dd, J = 8.5, 4.4 Hz, 1H), 5.91 (s, 1H), 5.03 (s, 2H), 3.60 (s, 3H), 2.06 – 1.70 (m, 1H), 1.02 – 0.84 (m, 2H), 0.73-0.68 (m, 1H). MS (ESI) *m/z* 341.1 [M+Na] $^+$.

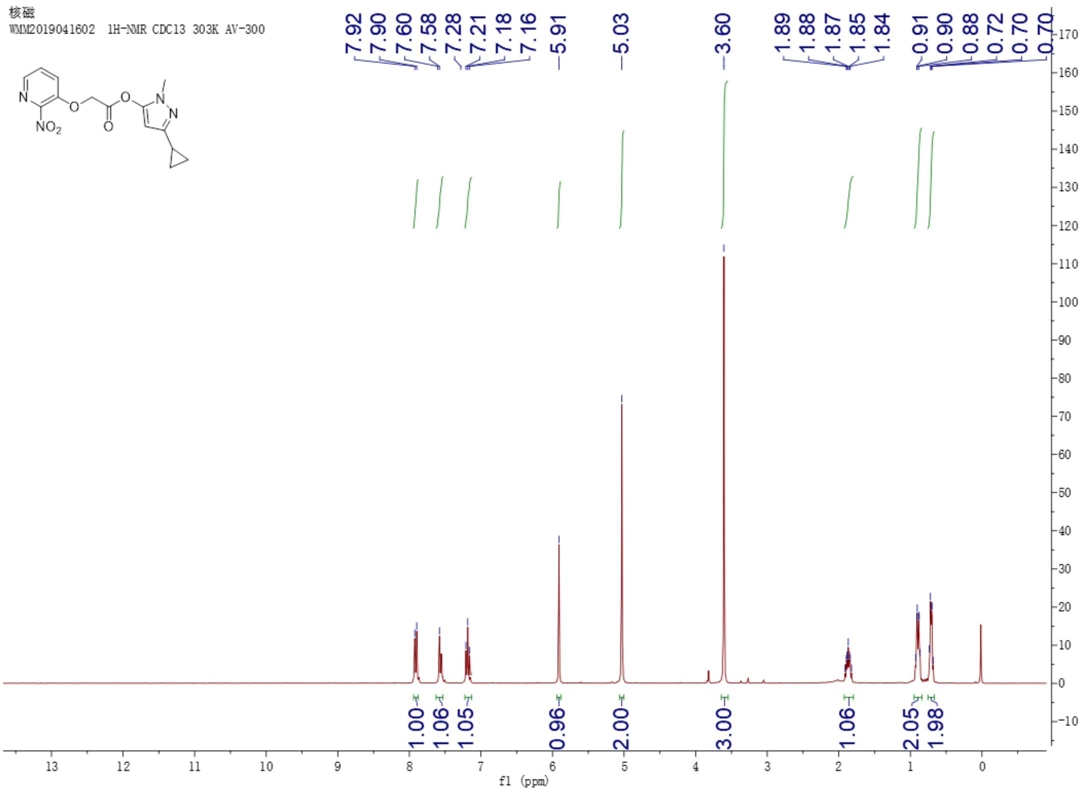
核磁
WMM2019041602 1H-NMR CDCl3 303K AV-300
7.92
7.90
7.60
7.58
7.28
7.21
7.18
7.16
5.91
5.03
3.60
1.89
1.88
1.87
1.85
1.84
0.91
0.90
0.88
0.72
0.70
0.70
1.00
1.06
1.05
0.96
2.00
3.00
1.06
2.05
1.98
f1 (ppm)

Data for target compounds

1-(3-Cyclopropyl-5-hydroxy-1-methyl-1*H*-pyrazol-4-yl)-2-phenoxyethan-1-one (I1):

Yield, 90%; white solid; ^{1}H NMR (300 MHz, $CDCl_3$) δ 17.14 (s, 1H), 7.29 (t, J = 7.9 Hz, 2H), 6.99 (d, J = 7.3 Hz, 1H), 6.94 (d, J = 8.1 Hz, 2H), 5.32 (s, 2H), 2.71 (t, J = 6.3 Hz, 2H), 2.57 – 2.43 (m, 2H), 2.02 (p, J = 6.4 Hz, 2H). ^{13}C NMR (75 MHz, DMSO) δ 189.23, 158.78, 153.47, 129.76, 120.90, 114.81, 103.34, 71.03, 31.64, 8.74, 8.71. HRMS m/z: calculated 273.11609 ($C_{15}H_{16}N_2O_3$ + H)$^+$, found 273.11617 ($C_{15}H_{16}N_2O_3$ + H)$^+$.

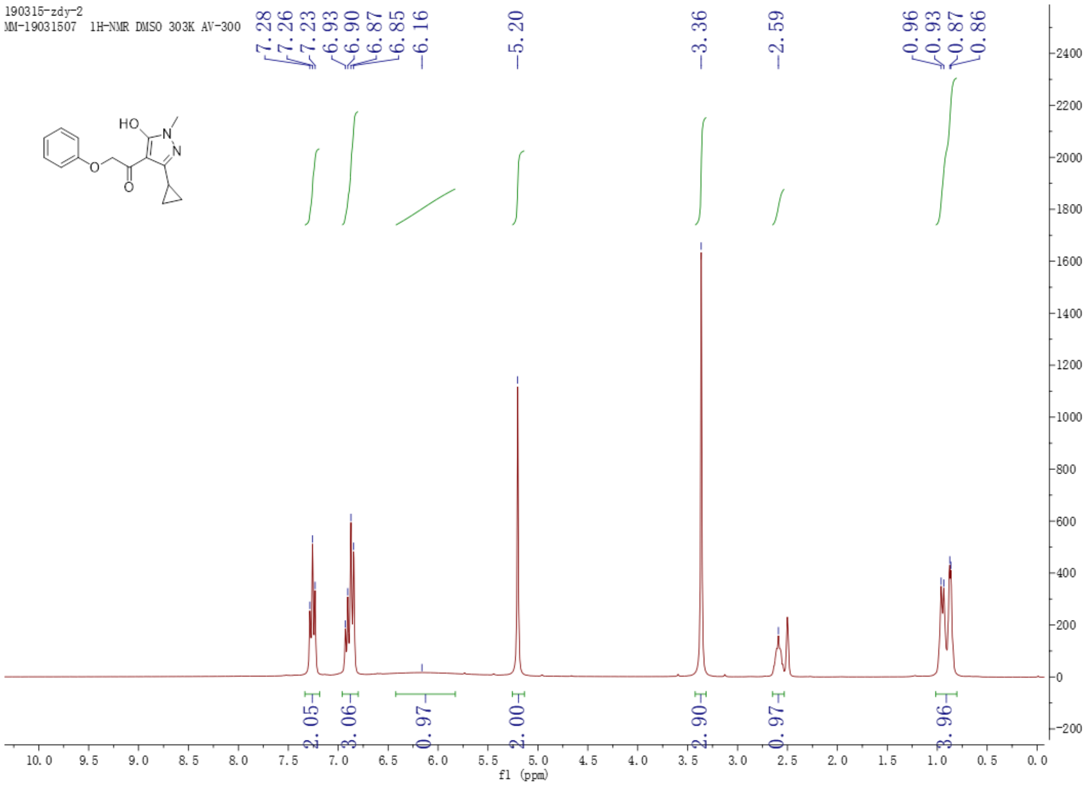

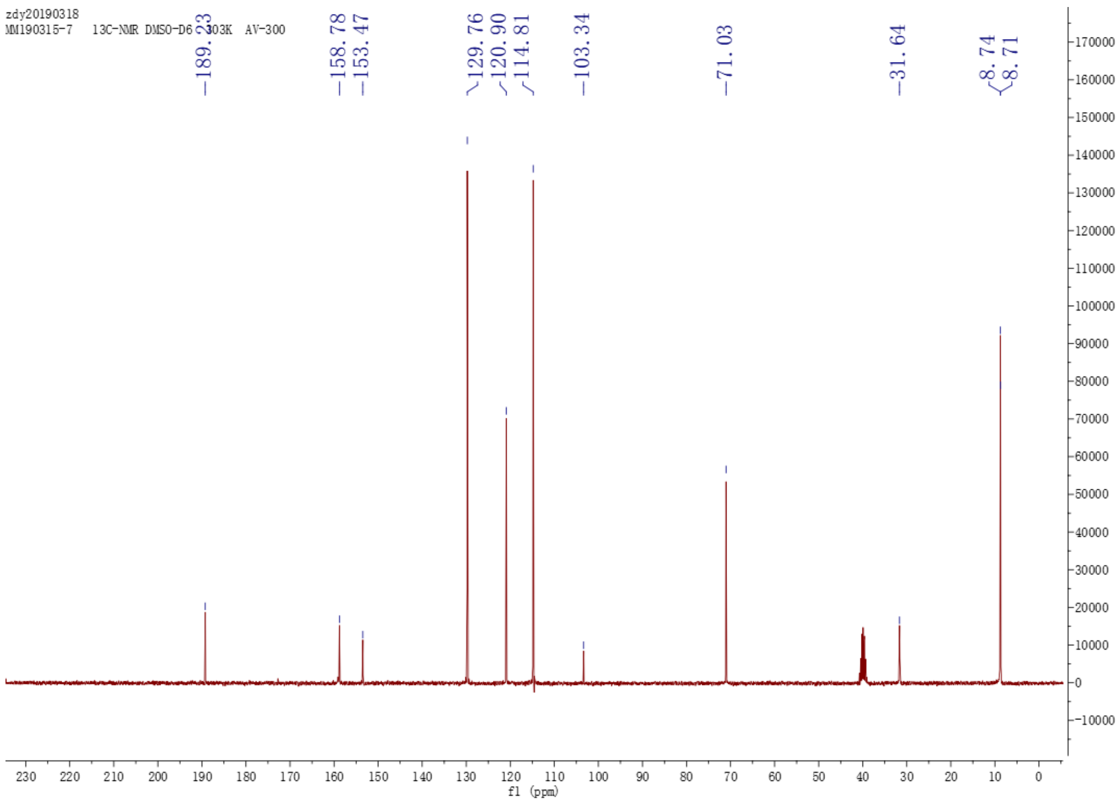

1-(5-Hydroxy-1-methyl-3-(trifluoromethyl)-1*H*-pyrazol-4-yl)-2-phenoxyethan-1-one (I2):

Yield, 93%; white solid; ^{1}H NMR (300 MHz, $CDCl_3$) δ 10.82 (s, 1H), 7.34 (t, J = 8.0 Hz, 2H), 7.06 (d, J = 7.3 Hz, 1H), 6.96 (d, J = 8.0 Hz, 2H), 5.14 (s, 2H), 3.78 (s, 3H). ^{13}C NMR (75 MHz, $CDCl_3$) δ 192.08, 159.70, 157.71, 129.62, 122.16, 121.86, 118.59, 114.56, 97.76, 69.78, 33.60. MS (ESI) HRMS m/z: calculated 301.07218 $(C_{13}H_{11}F_3N_2O_3 + H)^+$, found 301.07218 $(C_{13}H_{11}F_3N_2O_3 + H)^+$.

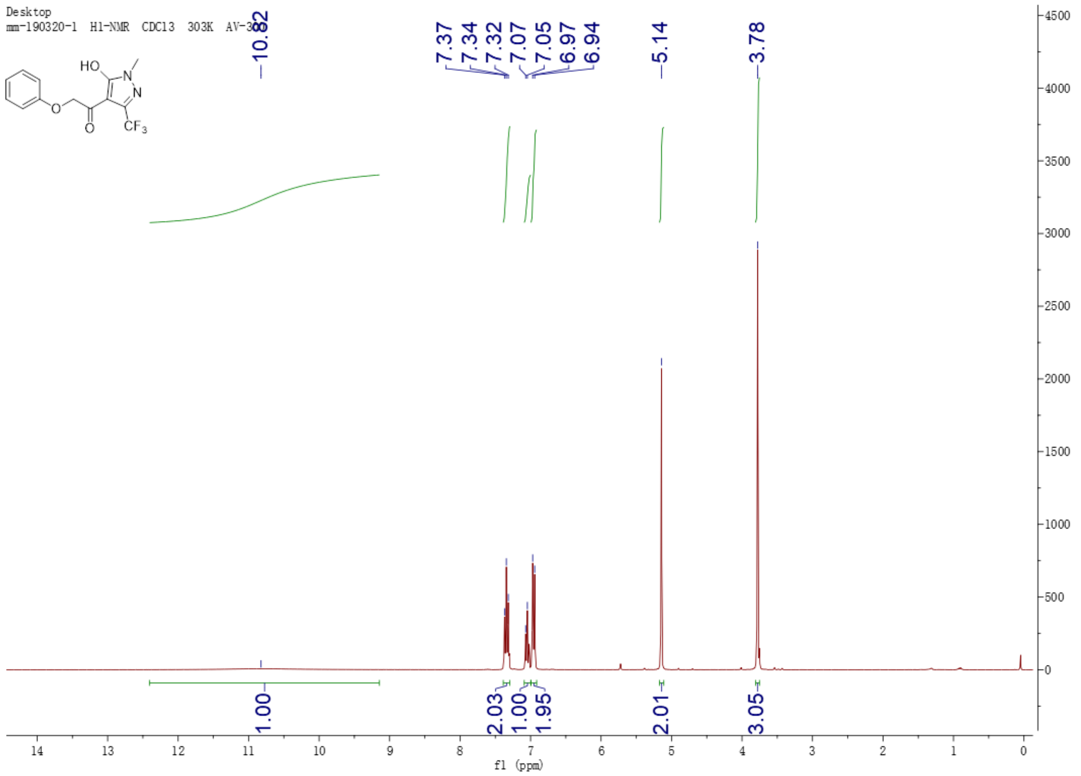

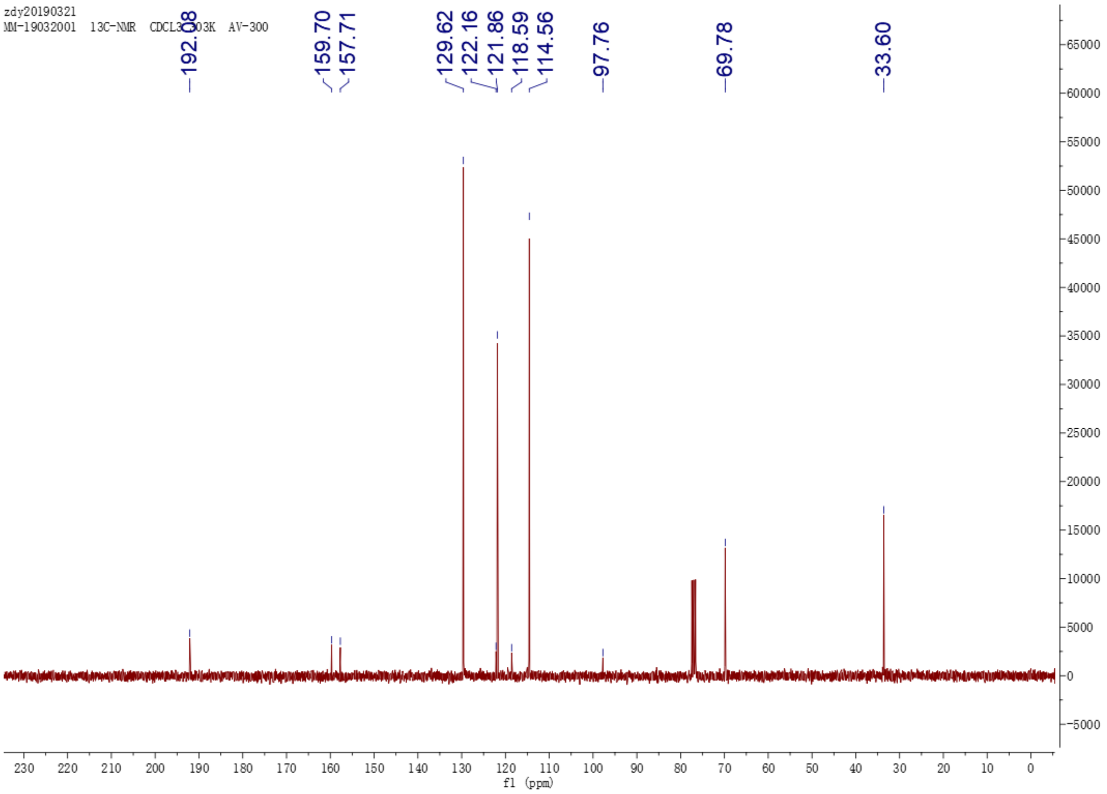

2-(2-Chlorophenoxy)-1-(3-cyclopropyl-5-hydroxy-1-methyl-1*H*-pyrazol-4-yl)ethan-1-one (I3):

Yield, 90%; white solid; ^{1}H NMR (300 MHz, $CDCl_3$) δ 7.41 (d, J = 7.7 Hz, 1H), 7.20

(t, J = 7.7 Hz, 1H), 6.96 (t, J = 7.6 Hz, 1H), 6.88 (d, J = 8.2 Hz, 1H), 6.20 (s, 2H), 5.26 (s, 3H), 3.58 (s, 3H), 2.16 – 1.73 (m, 1H), 0.98 (d, J = 6.4 Hz, 4H). ^{13}C NMR (75 MHz, $CDCl_3$) δ 191.23, 159.04, 153.78, 150.84, 130.63, 127.64, 122.45, 113.93, 101.35, 71.09, 32.56, 9.80, 6.47. HRMS m/z: calculated 307.07712 ($C_{15}H_{15}ClN_2O_3$ + H)$^+$, found 307.07712 ($C_{15}H_{15}ClN_2O_3$ + H)$^+$.

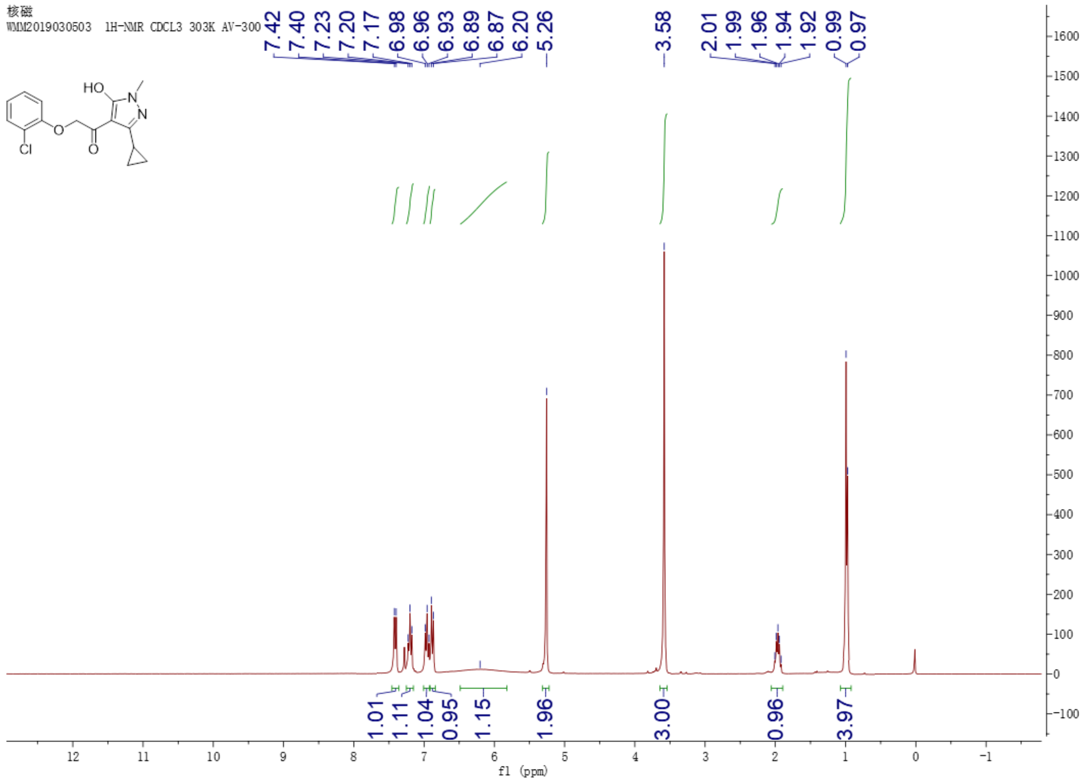

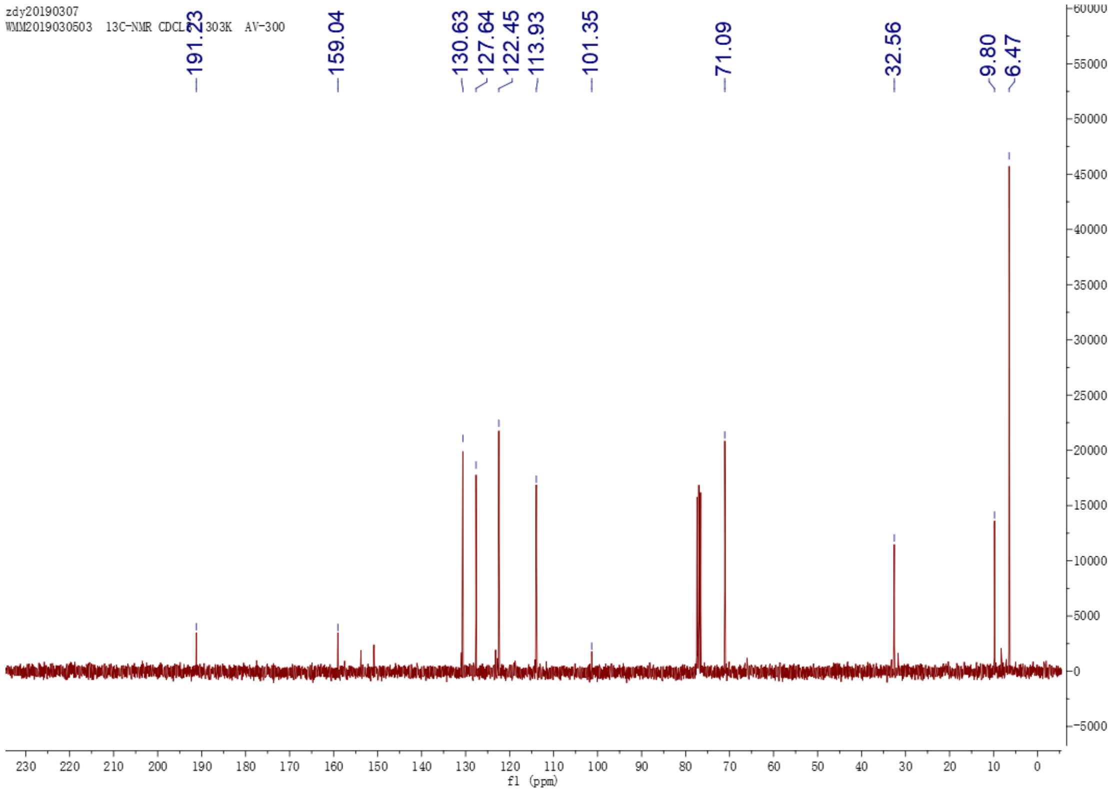

2-(2-Chlorophenoxy)-1-(5-hydroxy-1-methyl-3-(trifluoromethyl)-1*H*-pyrazol-4-yl)ethan-1-one (I4):

Yield, 90%; yellow solid; ^{1}H NMR (300 MHz, $CDCl_3$) δ 10.03 (s, 1H), 7.44 (dd, J = 7.9, 1.5 Hz, 1H), 7.27 – 7.16 (m, 1H), 7.00 (dd, J = 10.9, 4.4 Hz, 1H), 6.83 (d, J = 8.2 Hz, 2H), 5.22 (s, 2H), 3.78 (s, 3H). ^{13}C NMR (75 MHz, $CDCl_3$) δ 190.59, 159.12, 152.91, 130.16, 127.24, 127.12, 122.71, 122.50, 122.11, 121.59, 118.02, 113.75, 113.23, 76.90, 76.47, 76.05, 69.99, 65.41, 33.10. HRMS m/z: calculated 335.03320 $(C_{13}H_{10}ClF_3N_2O_3 + H)^+$, found 335.03330 $(C_{13}H_{10}ClF_3N_2O_3 + H)^+$.

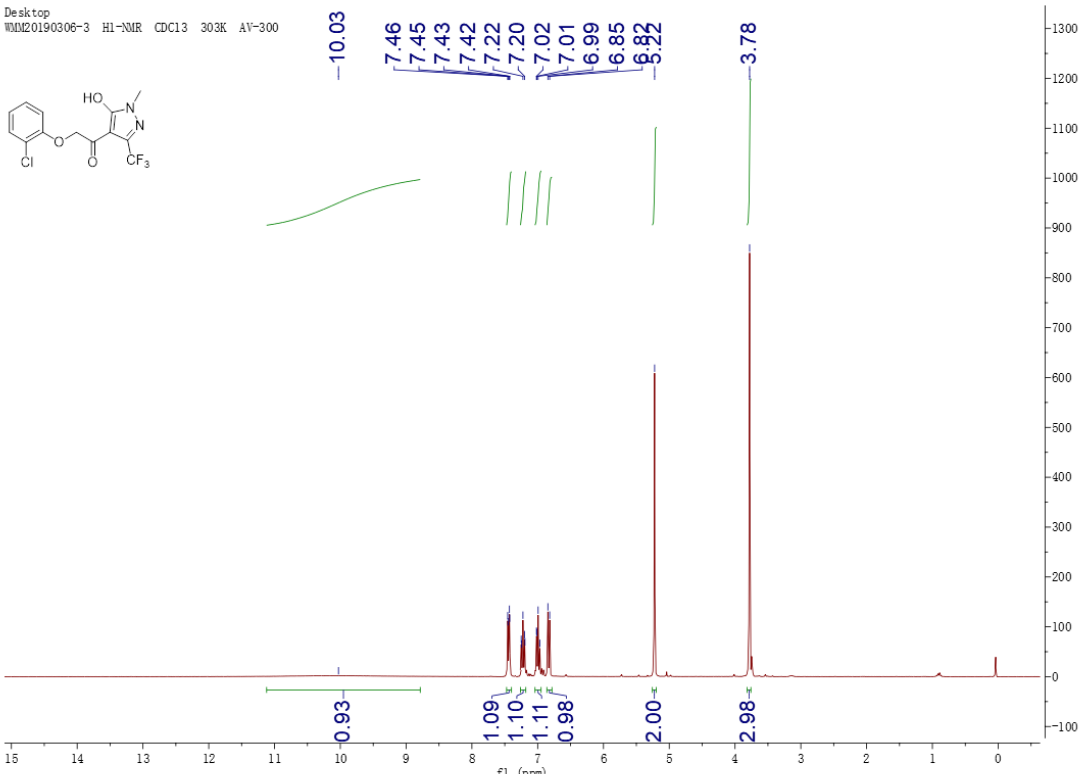

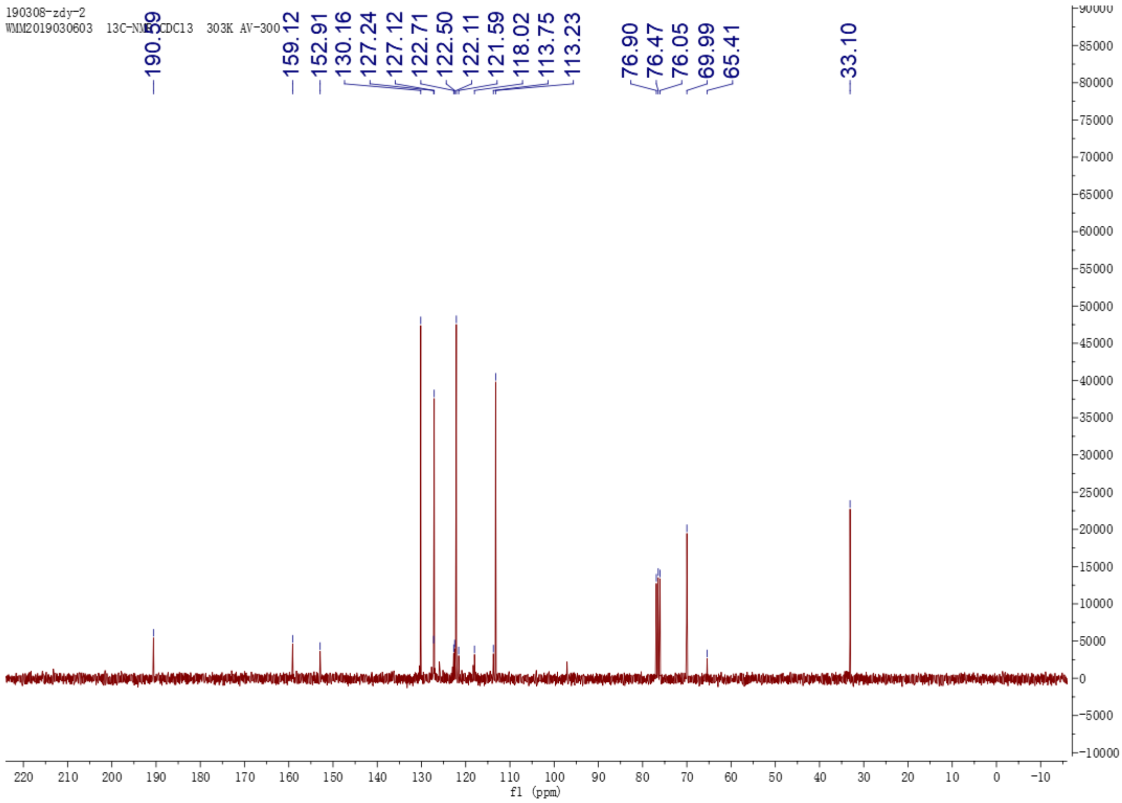

2-(2-(4-Chlorophenoxy)acetyl)-3-hydroxy-5,5-dimethylcyclohex-2-en-1-one (I5): Yield, 75%; white solid; ^{1}H NMR (300 MHz, $CDCl_3$) δ 16.72 (s, 1H), 7.25 (d, J = 8.9 Hz, 2H), 6.88 (d, J = 8.9 Hz, 2H), 5.30 (s, 2H), 2.51 (s, 4H), 1.13 (s, 6H). ^{13}C NMR

(75 MHz, CDCl$_3$) δ 199.41, 194.96, 156.21, 128.80, 128.72, 125.74, 115.55, 110.72, 71.35, 45.06, 30.60, 27.58, 7.94. HRMS m/z: calculated 309.08154 ($C_{16}H_{17}ClO_4$ + H)$^+$, found 309.08168 ($C_{16}H_{17}ClO_4$ + H)$^+$.

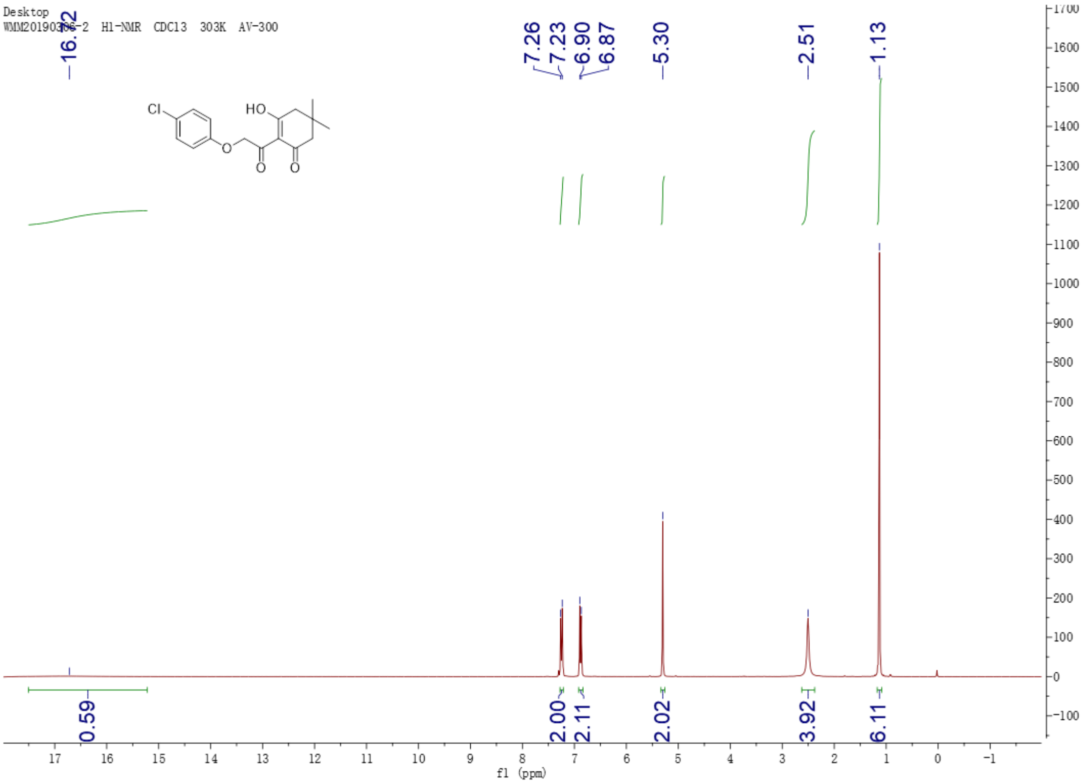

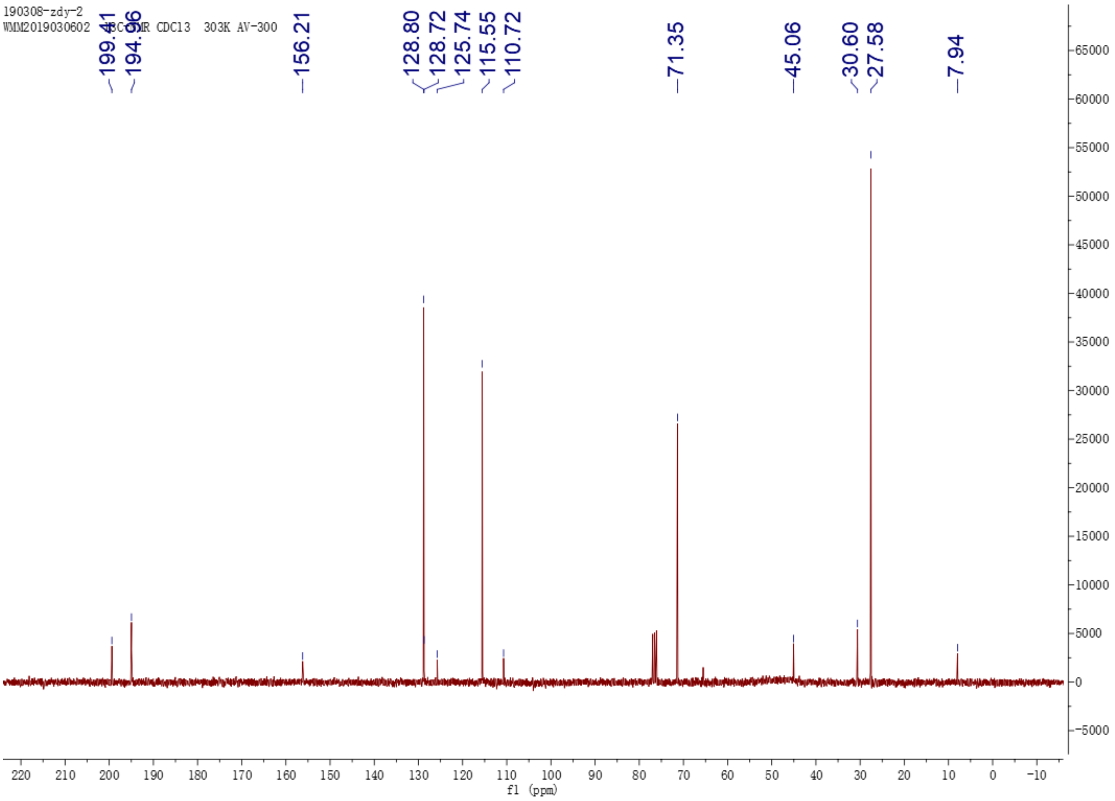

2-(4-Chlorophenoxy)-1-(5-hydroxy-1-methyl-3-(trifluoromethyl)-1*H*-pyrazol-4-yl)ethan-1-one (I6):

Yield, 85%; white solid; ^{1}H NMR (300 MHz, $CDCl_3$) δ 10.66 (s, 1H), 7.26 (d, J = 8.9 Hz, 2H), 6.85 (d, J = 8.9 Hz, 2H), 5.09 (s, 2H), 3.76 (s, 3H). ^{13}C NMR (75 MHz, $CDCl_3$) δ 191.72, 159.79, 156.44, 129.57, 126.89, 122.19, 118.62, 116.07, 115.96, 70.07,65.10,33.69. HRMS m/z: calculated 335.03320 $(C_{13}H_{10}ClF_3N_2O_3 + H)^+$, found 335.03347 $(C_{13}H_{10}ClF_3N_2O_3 + H)^+$.

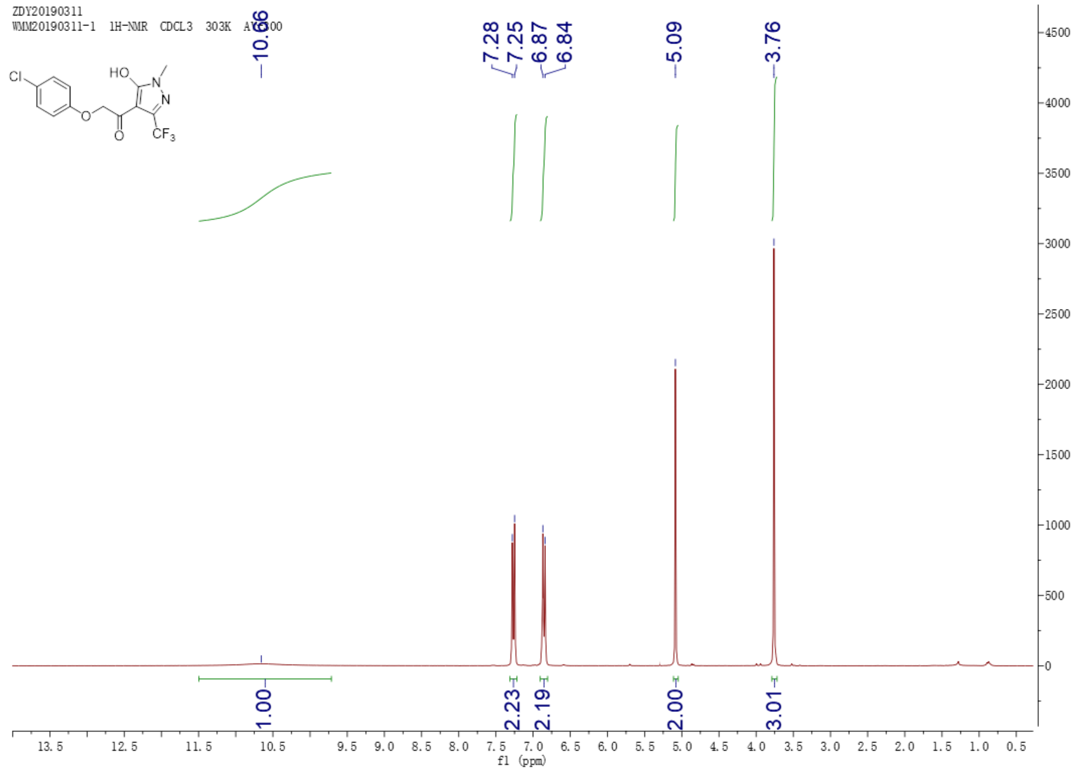

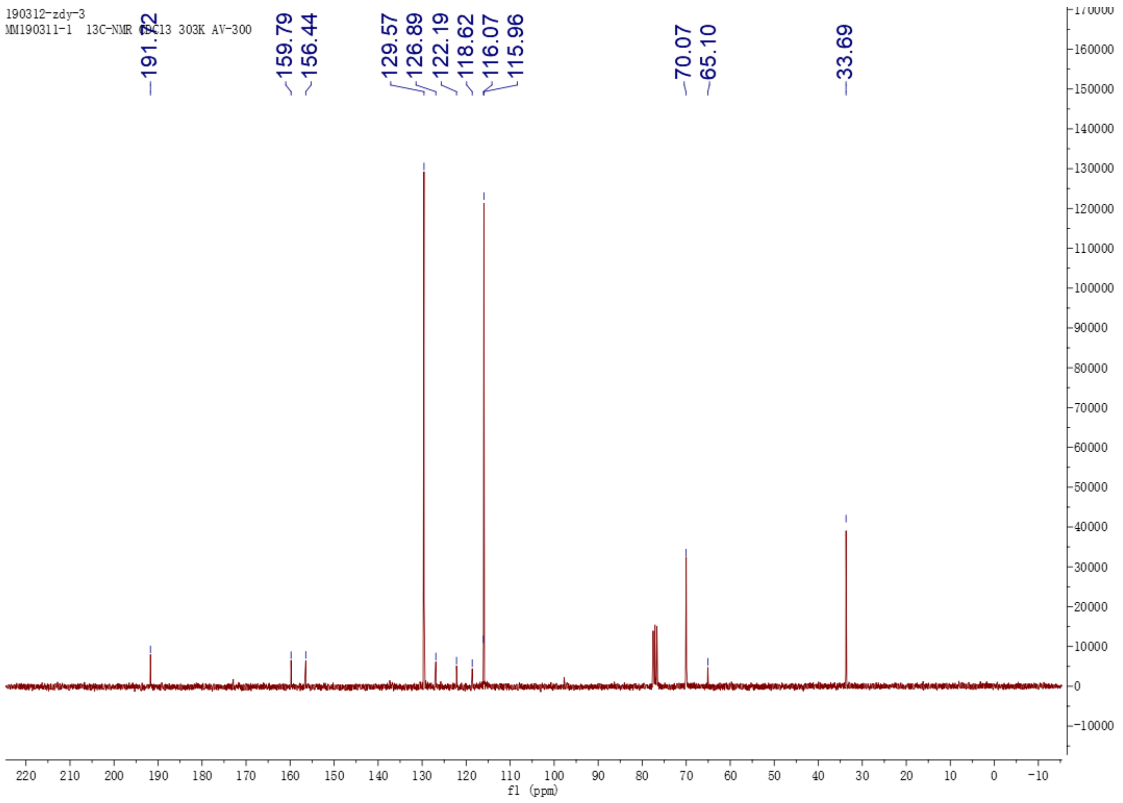

1-(3-Cyclopropyl-5-hydroxy-1-methyl-1*H*-pyrazol-4-yl)-2-(2,4-dichlorophenoxy)ethan-1-one (I7):

Yield, 95%; white solid; ^{1}H NMR (300 MHz, $CDCl_3$) δ 11.73 (s, 1H), 7.32 (s, 1H), 7.10 (d, J = 8.8 Hz, 1H), 6.76 (d, J = 8.8 Hz, 1H), 5.25 (s, 2H), 3.53 (s, 2H), 3.17 – 2.86 (m, 1H), 1.36 (t, J = 7.3 Hz, 2H), 0.99 (d, J = 8.9 Hz, 2H). ^{13}C NMR (75 MHz, DMSO) δ 189.23, 158.78, 153.47, 129.76, 120.90, 114.81, 103.34, 71.03, 31.64, 8.74, 8.71. HRMS m/z: calculated 341.03815 ($C_{15}H_{14}Cl_2N_2O_3$ + H)$^+$, found 341.03823 ($C_{15}H_{14}Cl_2N_2O_3$ + H)$^+$.

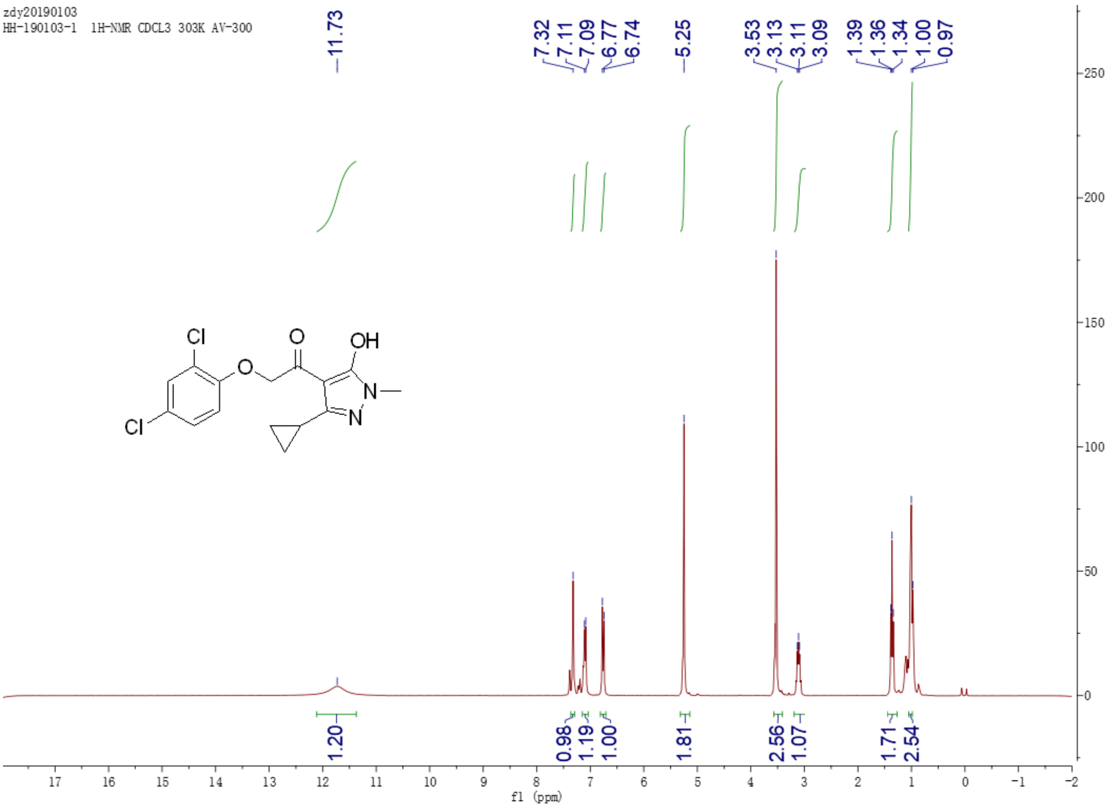

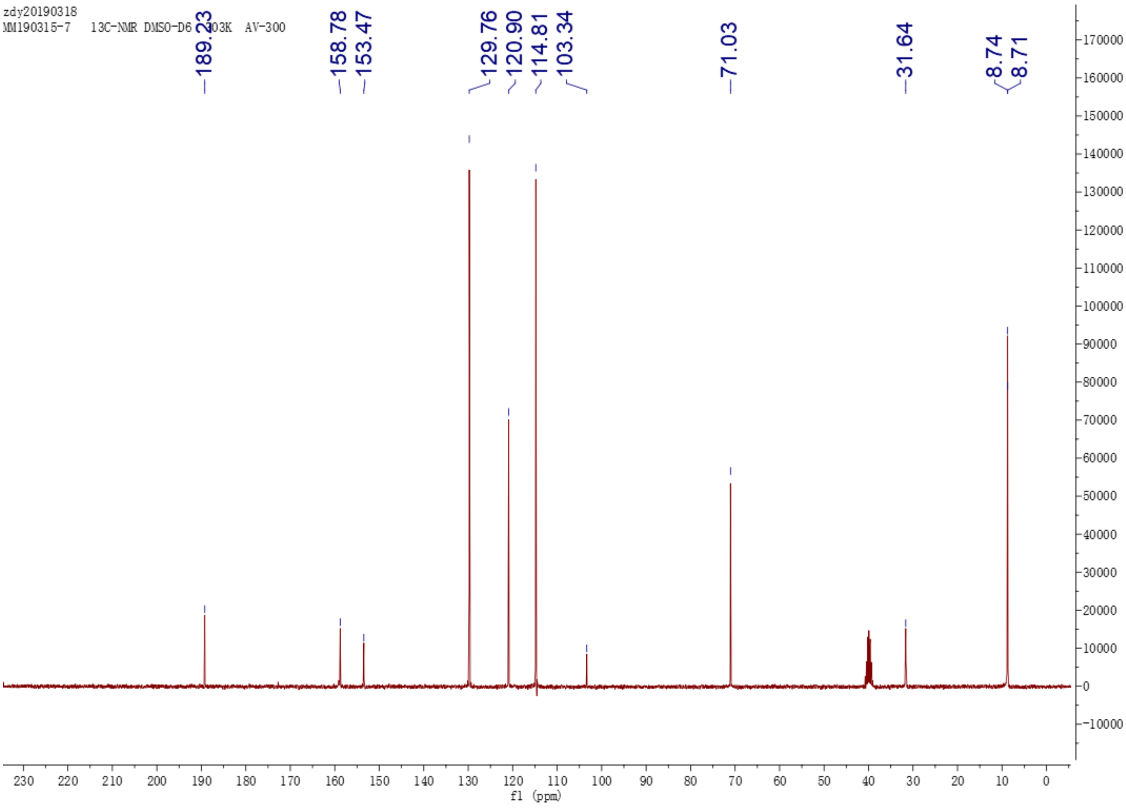

2-(2,4-Dichlorophenoxy)-1-(5-hydroxy-1-methyl-3-(trifluoromethyl)-1*H*-pyrazol-4-yl)ethan-1-one (I8):

Yield, 75%; white solid; ^{1}H NMR (300 MHz, $CDCl_3$) δ 10.56 (s, 1H), 7.39 (s, 1H),

7.15 (d, *J* = 6.7 Hz, 1H), 6.72 (d, *J* = 8.8 Hz, 1H), 5.16 (s, 2H), 3.75 (s, 3H). ^{13}C NMR (75 MHz, $CDCl_3$) δ 190.18, 159.14, 151.83, 129.82, 126.99, 123.57, 121.56, 117.99, 114.04, 96.99, 70.11, 33.10. HRMS m/z: calculated 368.99423 ($C_{13}H_9Cl_2F_3N_2O_3$ + H)$^+$, found 368.99525 ($C_{13}H_9Cl_2F_3N_2O_3$ + H)$^+$.

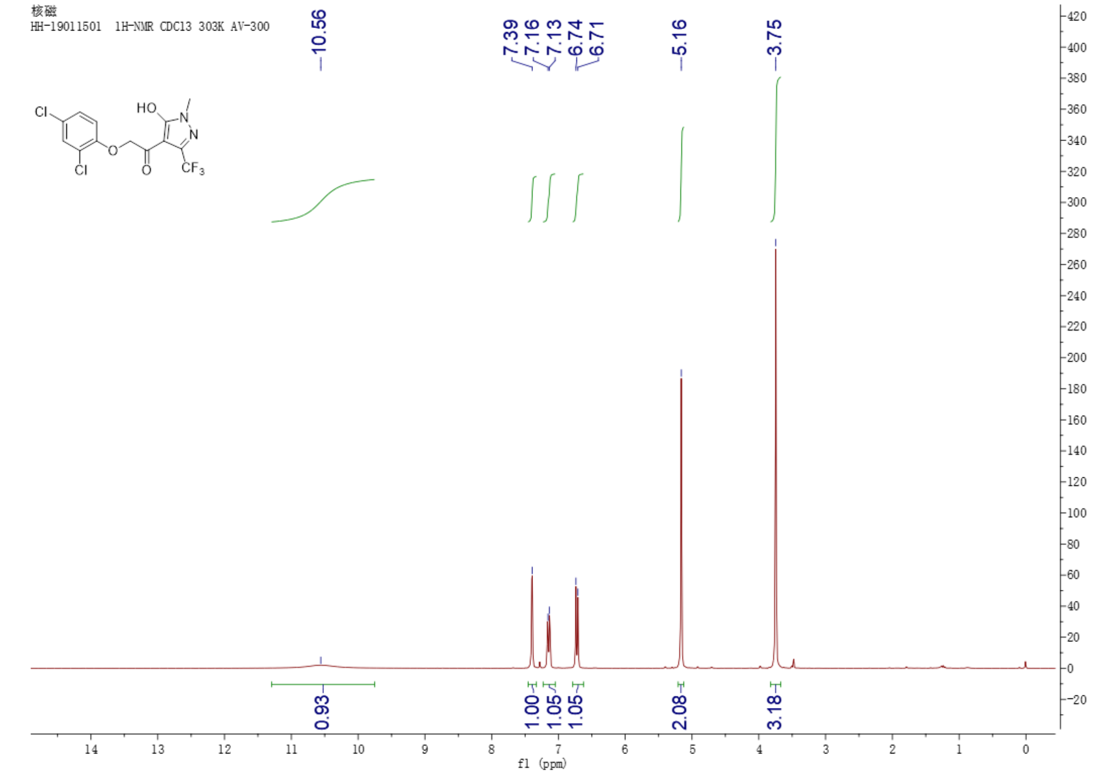

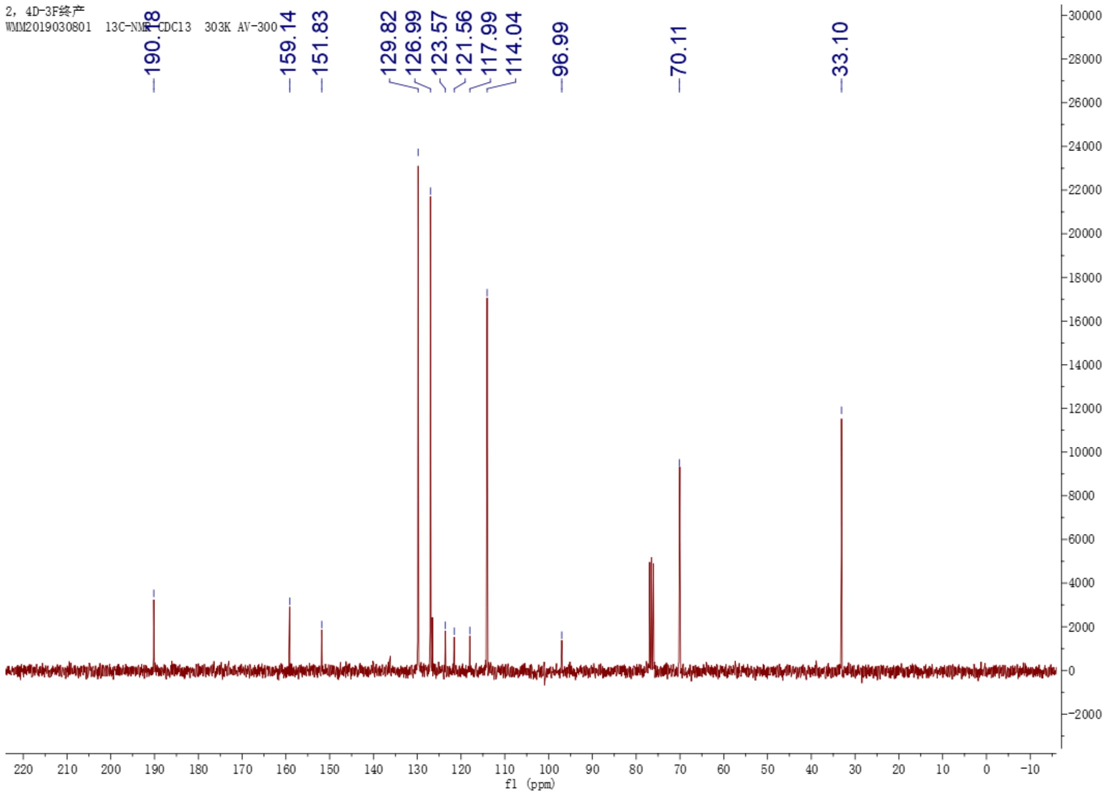

3-Hydroxy-5,5-dimethyl-2-(2-(2,4,6-trichlorophenoxy)acetyl)cyclohex-2-en-1-one (I9):

Yield, 86%; white solid; ^{1}H NMR (300 MHz, $CDCl_3$) δ 17.08 (s, 1H), 7.32 (s, 2H), 5.24 (s, 2H), 2.60 (s, 2H), 2.35 (s, 2H), 1.10 (s, 6H). ^{13}C NMR (75 MHz, $CDCl_3$) δ 198.01, 194.96, 194.78, 149.23, 129.57, 129.45, 128.31, 110.56, 75.06, 51.26, 44.93, 30.68, 27.64. HRMS m/z: calculated 377.00359 $(C_{16}H_{15}Cl_3O_4 + H)^+$, found 377.00367 $(C_{16}H_{15}Cl_3O_4 + H)^+$.

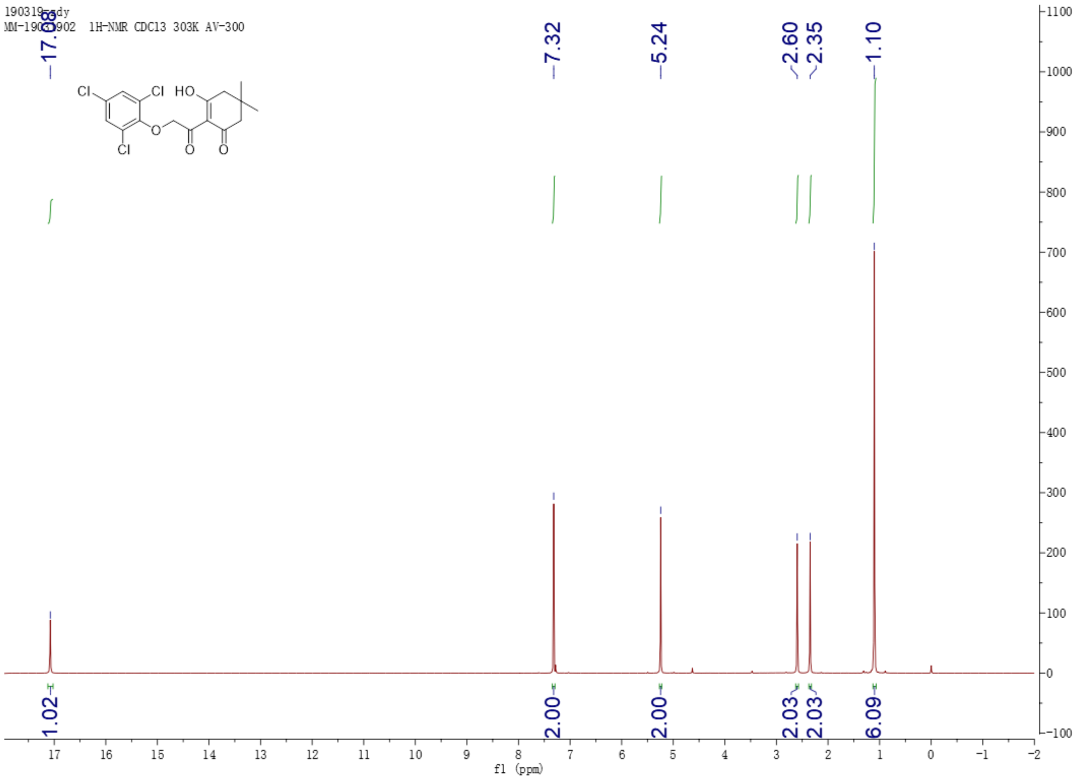

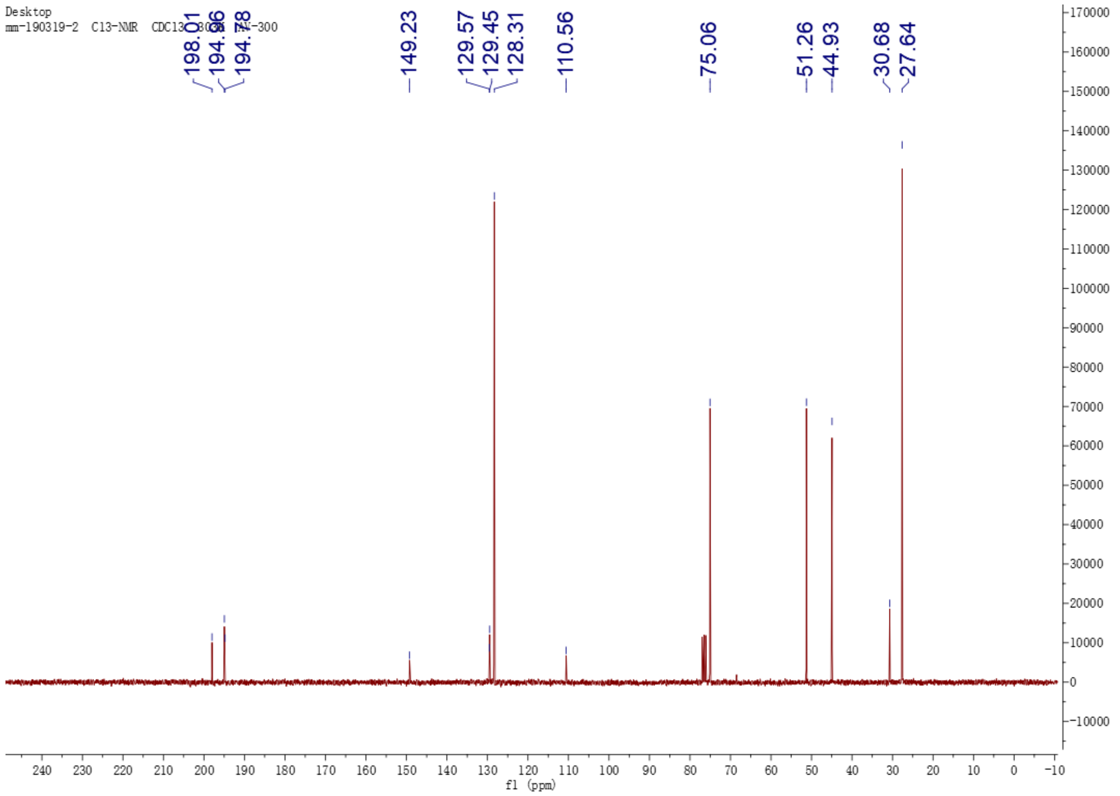

1-(5-Hydroxy-1,3-dimethyl-1*H*-pyrazol-4-yl)-2-(2,4,6-trichlorophenoxy)ethan-1-one (I10):

Yield, 79%; white solid; ^{1}H NMR (300 MHz, DMSO) δ 7.64 (s, 2H), 5.07 (s, 2H), 3.28 (s, 3H), 2.33 (s, 3H). ^{13}C NMR (75 MHz, DMSO) δ 186.50, 159.74, 149.93, 147.46, 128.95, 128.84, 128.74, 101.77, 75.36, 30.30, 13.28. HRMS m/z: calculated 348.98353 ($C_{13}H_{11}Cl_3N_2O_3$ + H)$^+$, found 348.98367 ($C_{13}H_{11}Cl_3N_2O_3$ + H)$^+$.

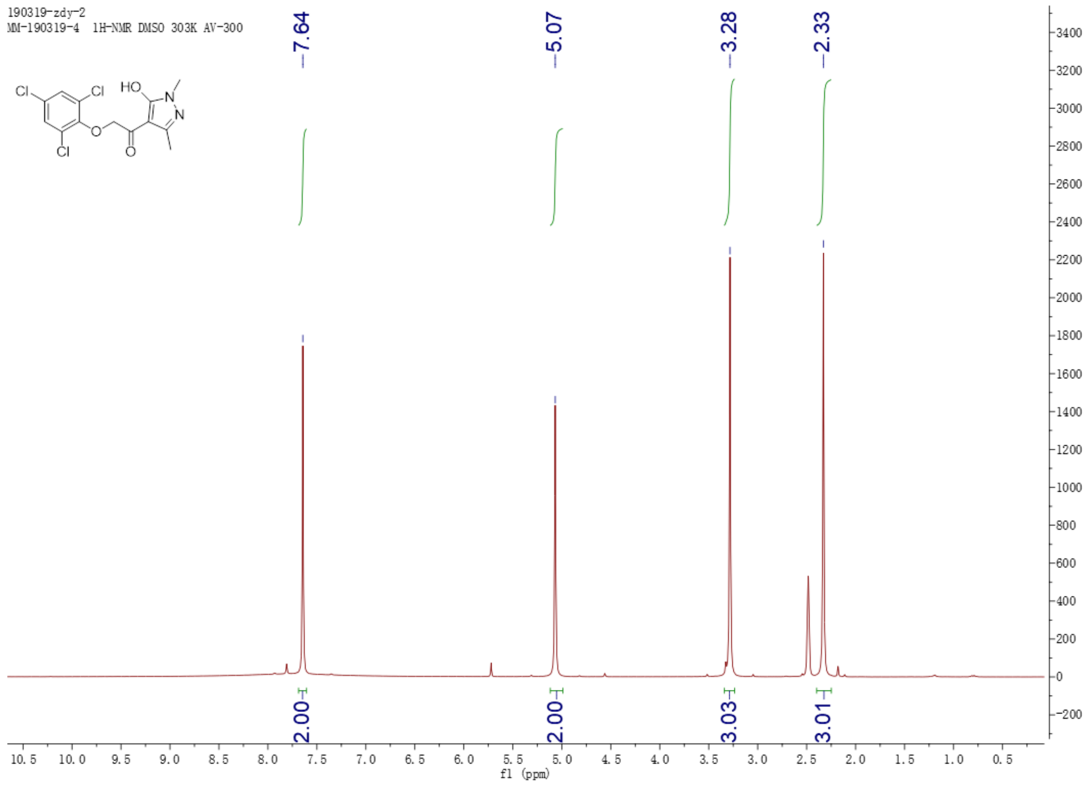

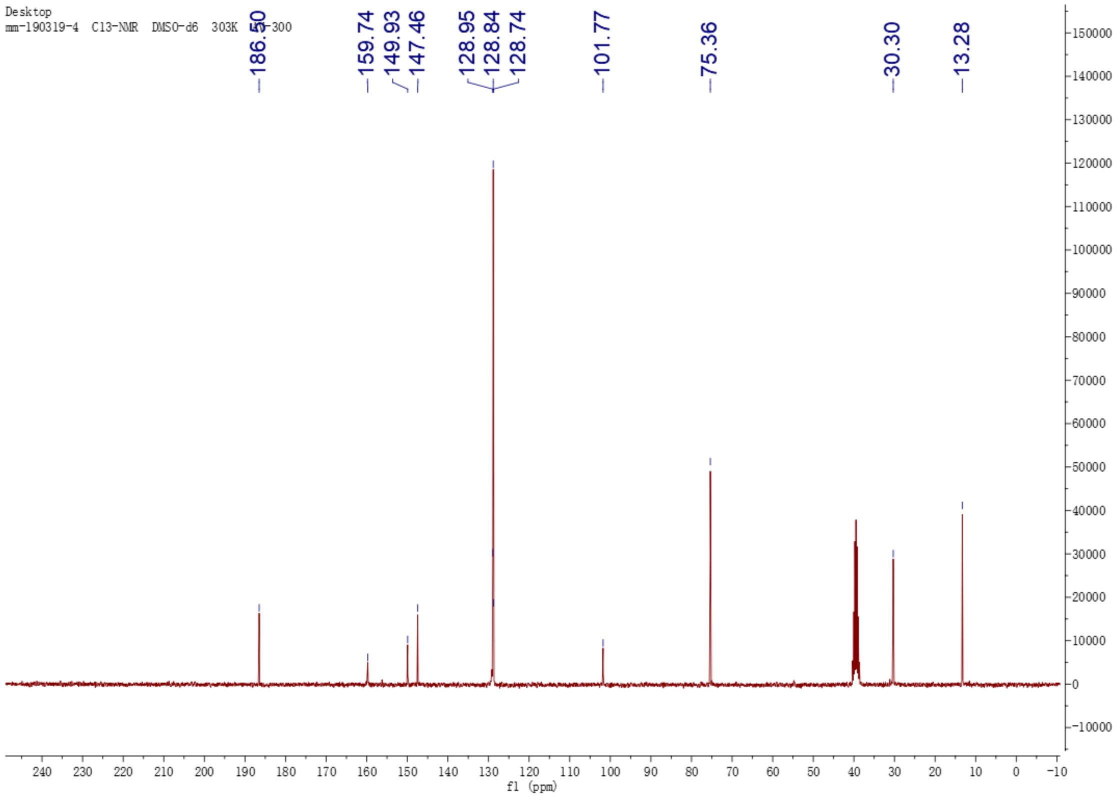

1-(3-Cyclopropyl-5-hydroxy-1-methyl-1*H*-pyrazol-4-yl)-2-(2,4,6-trichlorophenoxy)ethan-1-one (I11):

Yield, 87%; white solid; ^{1}H NMR (300 MHz, DMSO) δ 7.66 (s, 2H), 5.13 (s, 2H),

3.30 (s, 3H), 2.66 (s, 1H), 0.98 (m, 2H), 0.87 (m, 2H). ^{13}C NMR (75 MHz, DMSO) δ 186.55, 156.70, 153.21, 149.95, 129.14, 128.85, 102.43, 75.33, 30.83, 8.50, 8.06. HRMS m/z: calculated 374.99918 $(C_{15}H_{13}Cl_3N_2O_3 + H)^+$, found 374.99967 $(C_{15}H_{13}Cl_3N_2O_3 + H)^+$.

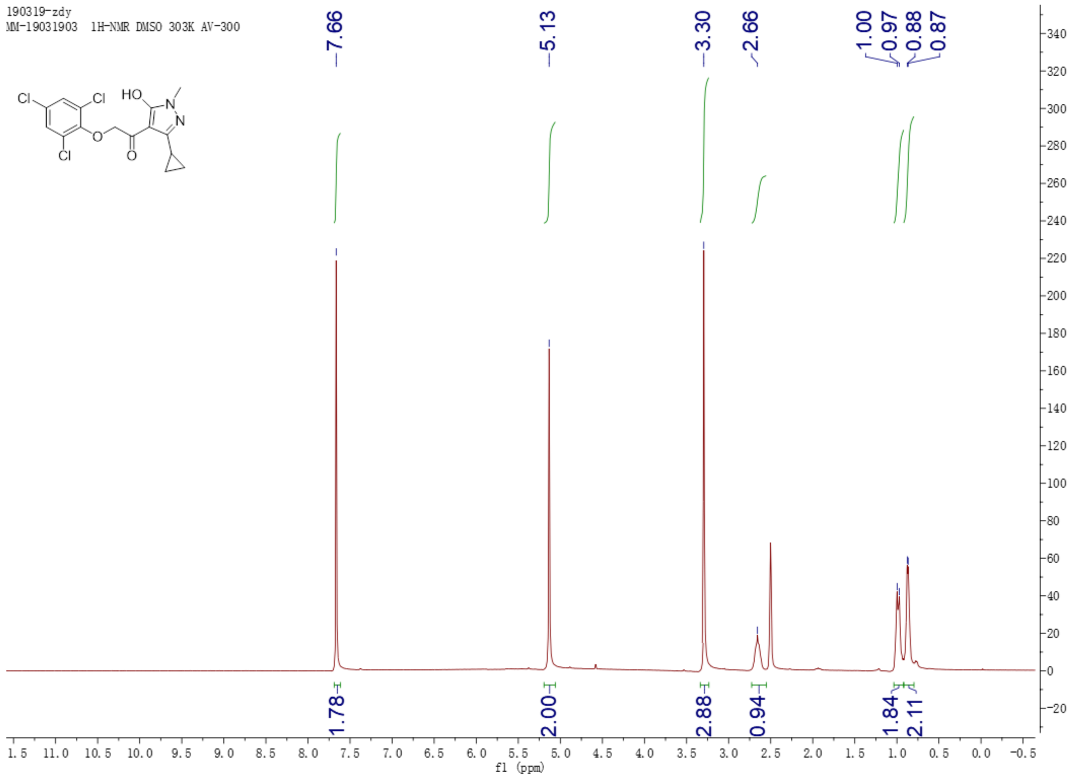

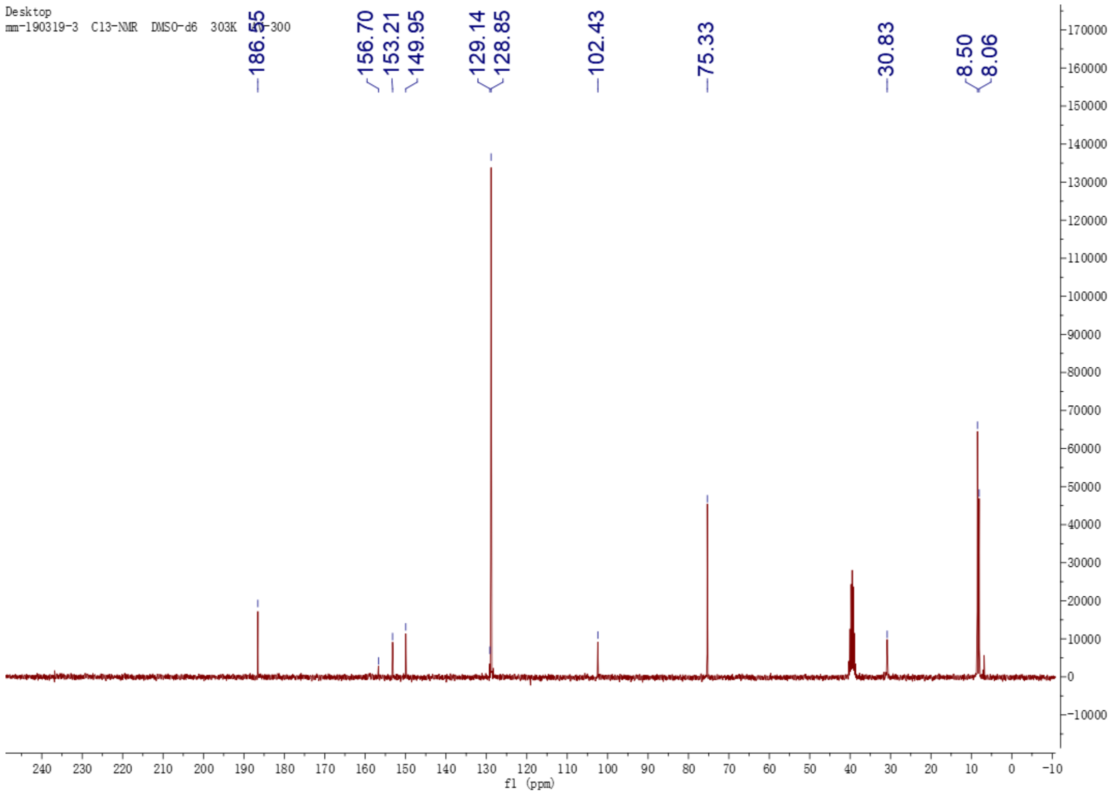

1-(5-Hydroxy-1-methyl-3-(trifluoromethyl)-1*H*-pyrazol-4-yl)-2-(2,4,6-trichlorophenoxy)ethan-1-one(I12):

Yield, 90%; white solid; ^{1}H NMR (300 MHz, $CDCl_3$) δ 10.87 (s, 1H), 7.37 (s, 2H), 5.11 (s, 2H), 3.78 (s, 3H). ^{13}C NMR (75 MHz, $CDCl_3$) δ 190.07, 159.72, 149.32, 130.43, 129.70, 128.97, 122.00, 118.43, 97.46, 73.31, 33.62. HRMS m/z: calculated 402.95526 ($C_{13}H_8Cl_3F_3N_2O_3$ + H)$^+$, found 402.95623 ($C_{13}H_8Cl_3F_3N_2O_3$ + H)$^+$.

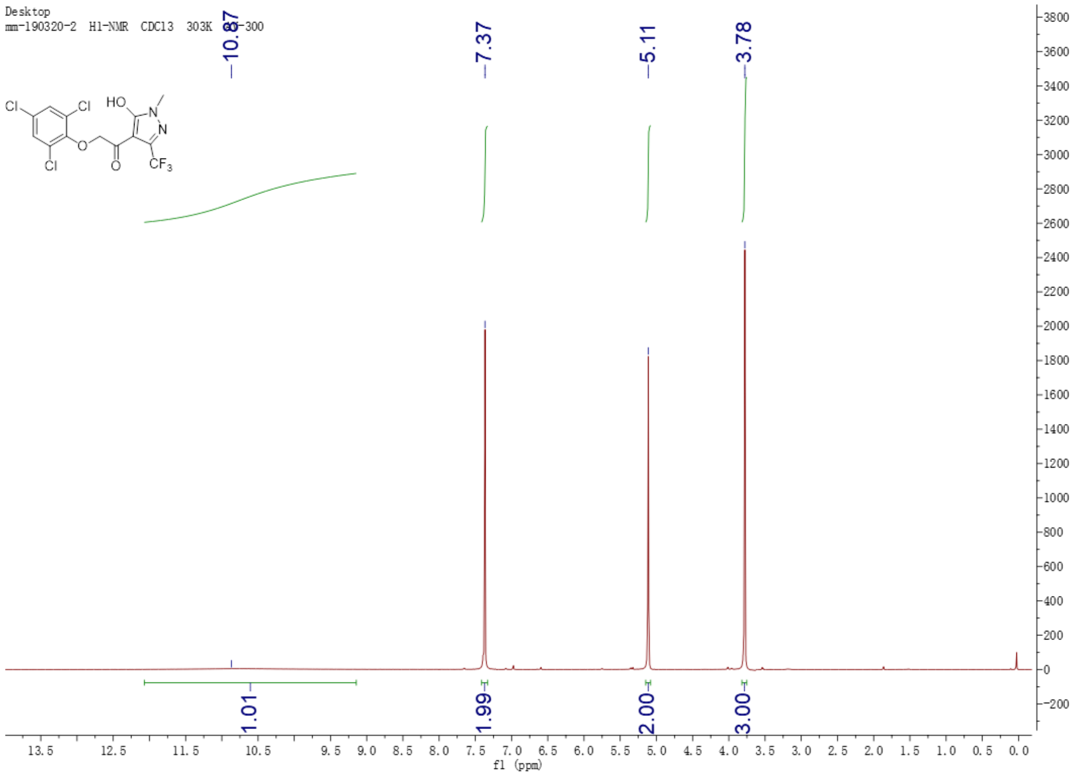

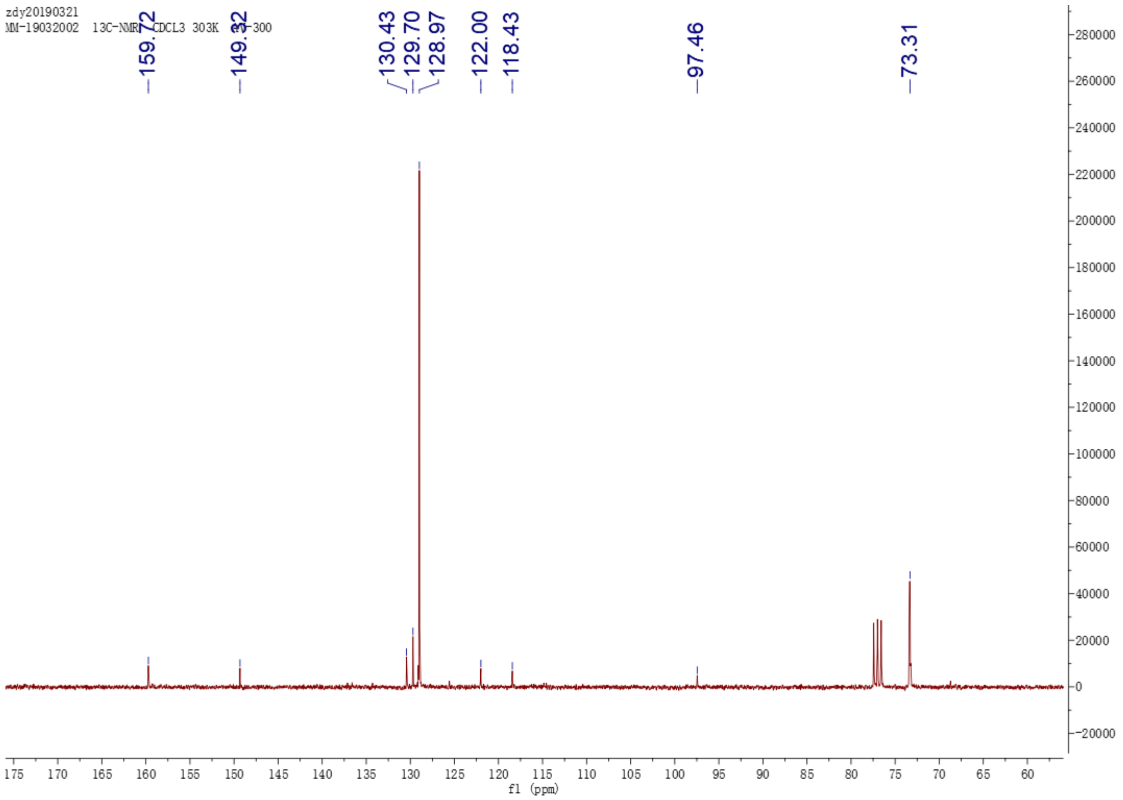

3-Hydroxy-5,5-dimethyl-2-(2-(2-nitrophenoxy)acetyl)cyclohex-2-en-1-one (I13):

Yield, 89%; white solid; ^{1}H NMR (300 MHz, $CDCl_3$) δ 16.75 (s, 1H), 7.84 (d, J = 6.6

Hz, 1H), 7.48 (t, J = 7.1 Hz, 1H), 7.05 (t, J = 7.4 Hz, 1H), 6.95 (d, J = 8.4 Hz, 1H), 5.44 (s, 2H), 2.59 (s, 2H), 2.38 (s, 2H), 1.10 (s, 6H). ^{13}C NMR (75 MHz, $CDCl_3$) δ 198.13, 195.05, 151.17, 133.45, 125.20, 120.66, 114.67, 110.78, 72.16, 51.25, 44.81, 30.72, 27.63. HRMS m/z: calculated 320.10559 ($C_{16}H_{17}NO_6$ + H)$^+$, found 320.10549 ($C_{16}H_{17}NO_6$ + H)$^+$.

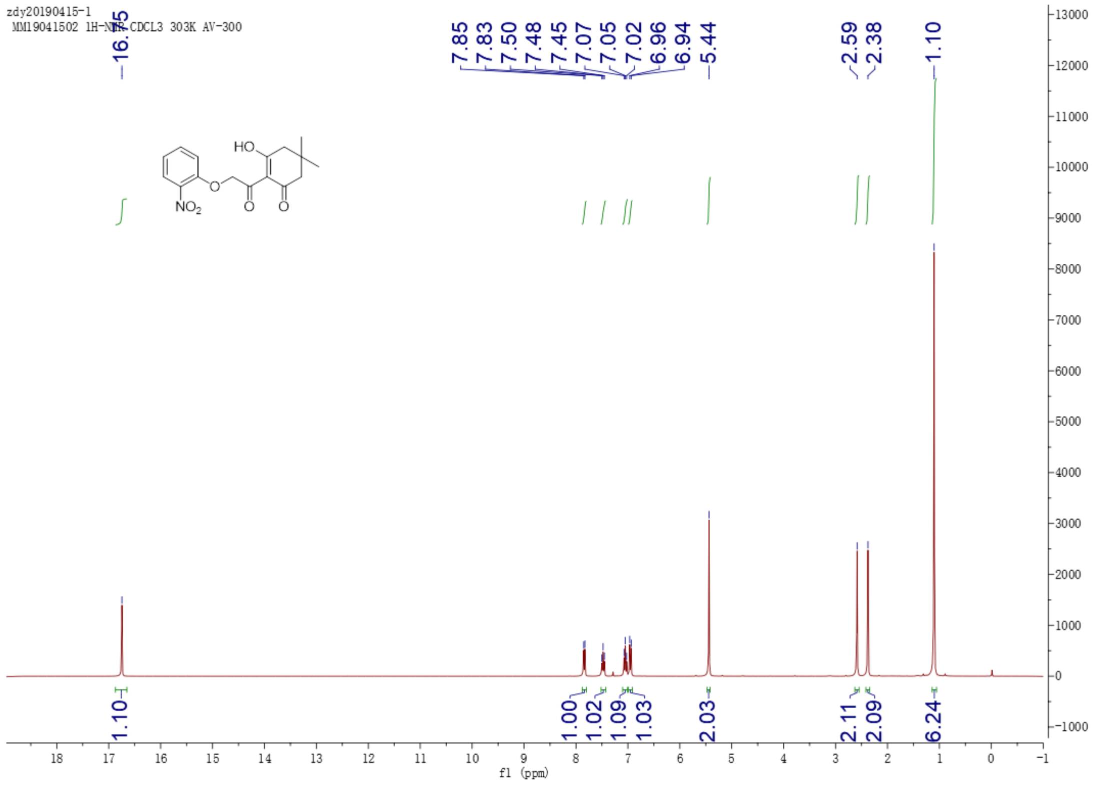

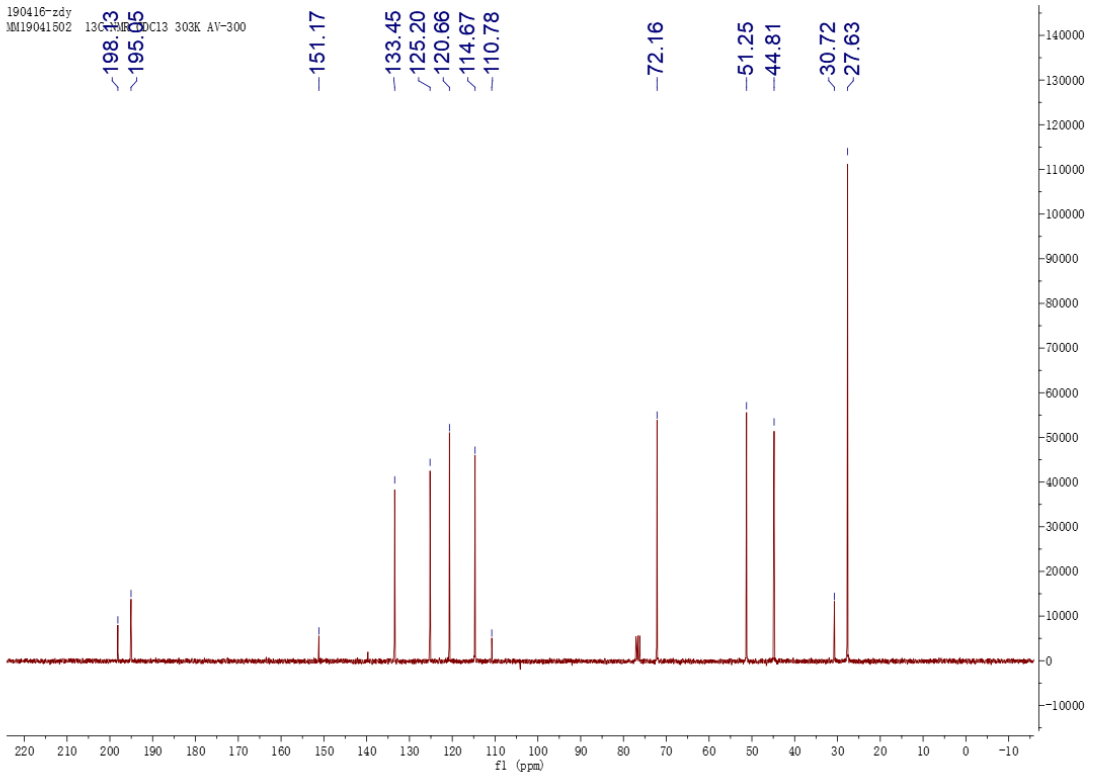

1-(5-Hydroxy-1,3-dimethyl-1*H*-pyrazol-4-yl)-2-(2-nitrophenoxy)ethan-1-one (I14):

Yield, 87%; white solid; ^{1}H NMR (300 MHz, DMSO) δ 7.84 (d, J = 8.3 Hz, 1H), 7.54 (t, J = 7.9 Hz, 1H), 7.08 (t, J = 8.0 Hz, 1H), 7.05 (d, J = 7.6 Hz, 1H), 5.37 (s, 2H), 3.35 (s, 3H), 2.29 (s, 3H). ^{13}C NMR (75 MHz, DMSO) δ 187.34, 151.24, 147.47, 139.50, 133.99, 124.79, 120.43, 115.13, 102.02, 71.47, 30.59, 13.42. HRMS m/z: calculated 292.08552 ($C_{13}H_{13}N_{3}O_{5}$ + H)$^{+}$, found 292.08542 ($C_{13}H_{13}N_{3}O_{5}$ + H)$^{+}$.

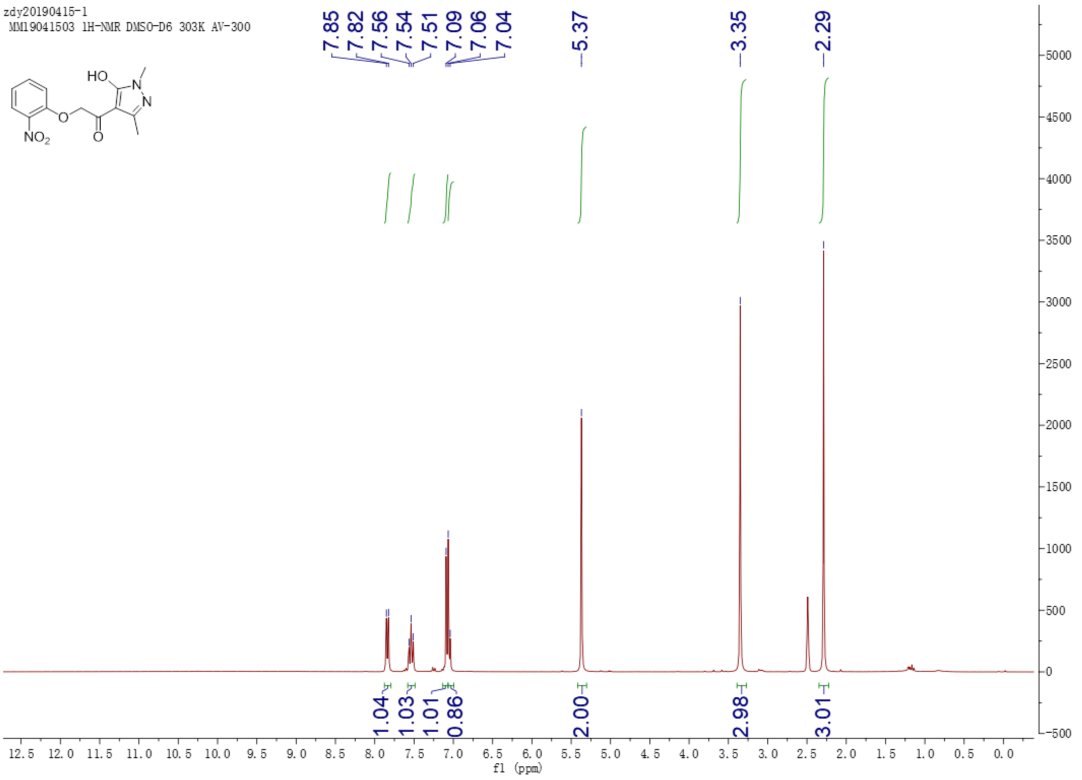

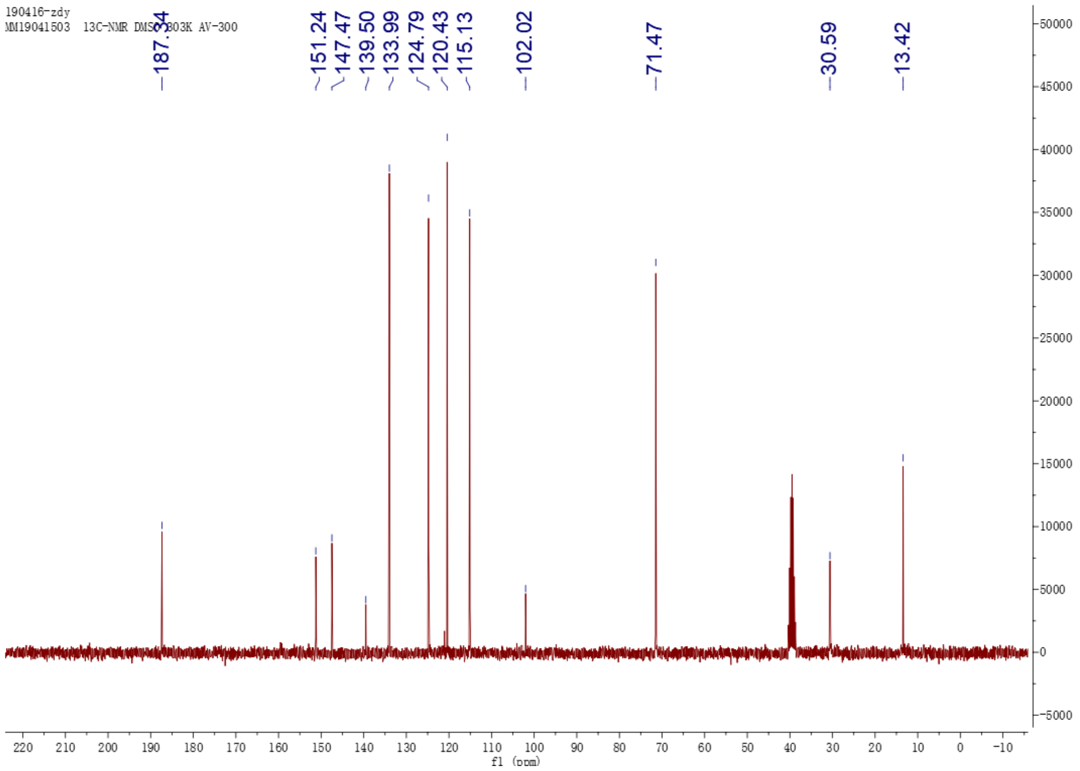

1-(3-Cyclopropyl-5-hydroxy-1-methyl-1*H*-pyrazol-4-yl)-2-(2-nitrophenoxy)ethan-1-one (I15):

Yield, 88%; white solid; ^{1}H NMR (300 MHz, DMSO) δ 7.84 (d, J = 7.0 Hz, 1H), 7.55

(t, J = 7.4 Hz, 1H), 7.10 (t, J = 7.9 Hz, 1H), 7.06 (d, J = 7.7 Hz, 1H), 5.41 (s, 2H), 3.35 (s, 3H), 2.62 – 2.45 (m, 1H), 1.03 – 0.77 (m, 4H). ^{13}C NMR (75 MHz, DMSO) δ 187.32, 169.25, 153.11, 151.27, 139.51, 134.00, 124.79, 120.43, 115.14, 102.63, 71.48, 65.32, 31.13, 8.35, 8.20. HRMS m/z: calculated 318.10117 ($C_{15}H_{15}N_3O_5$ + H)$^+$, found 318.10216 ($C_{15}H_{15}N_3O_5$ + H)$^+$.

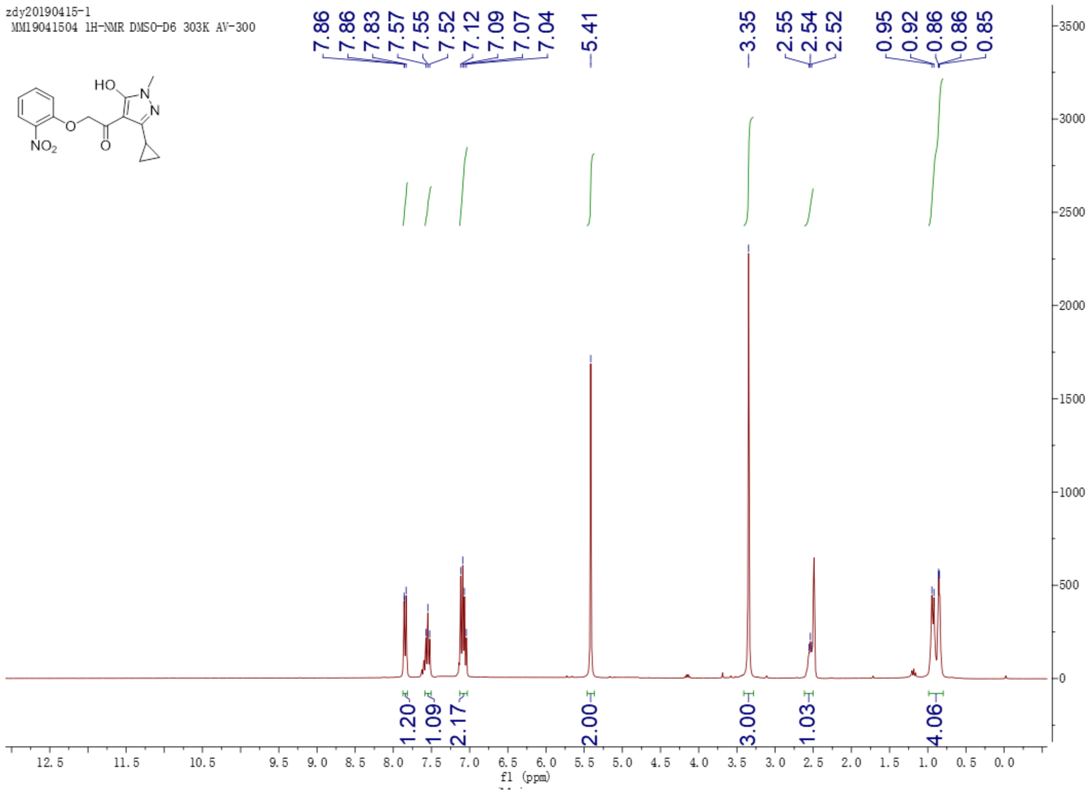

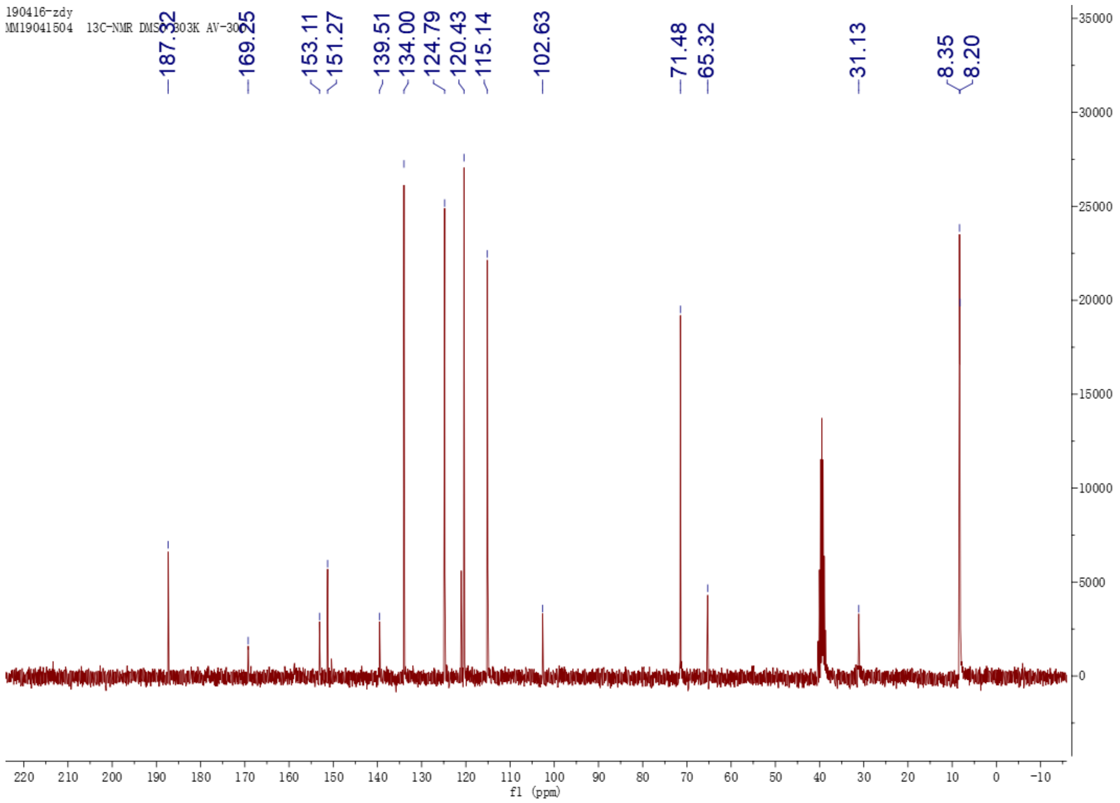

1-(5-Hydroxy-1-methyl-3-(trifluoromethyl)-1*H*-pyrazol-4-yl)-2-(2-nitrophenoxy)ethan-1-one (I16):

Yield, 75%; white solid; ^{1}H NMR (300 MHz, $CDCl_3$) δ 10.98 (s, 1H), 7.91 (d, J = 6.7 Hz, 1H), 7.53 (t, J = 8.7 Hz, 1H), 7.13 (t, J = 7.7 Hz, 1H), 6.95 (d, J = 8.4 Hz, 1H), 5.28 (s, 2H), 3.77 (s, 3H). ^{13}C NMR (75 MHz, $CDCl_3$) δ 189.67, 159.20, 150.75, 133.52, 125.44, 121.64, 121.27, 118.07, 114.44, 99.34, 70.24, 33.25. HRMS m/z: calculated 346.05726 ($C_{13}H_{10}F_3N_3O_5$ + H)$^+$, found 346.05726 ($C_{13}H_{10}F_3N_3O_5$ + H)$^+$.

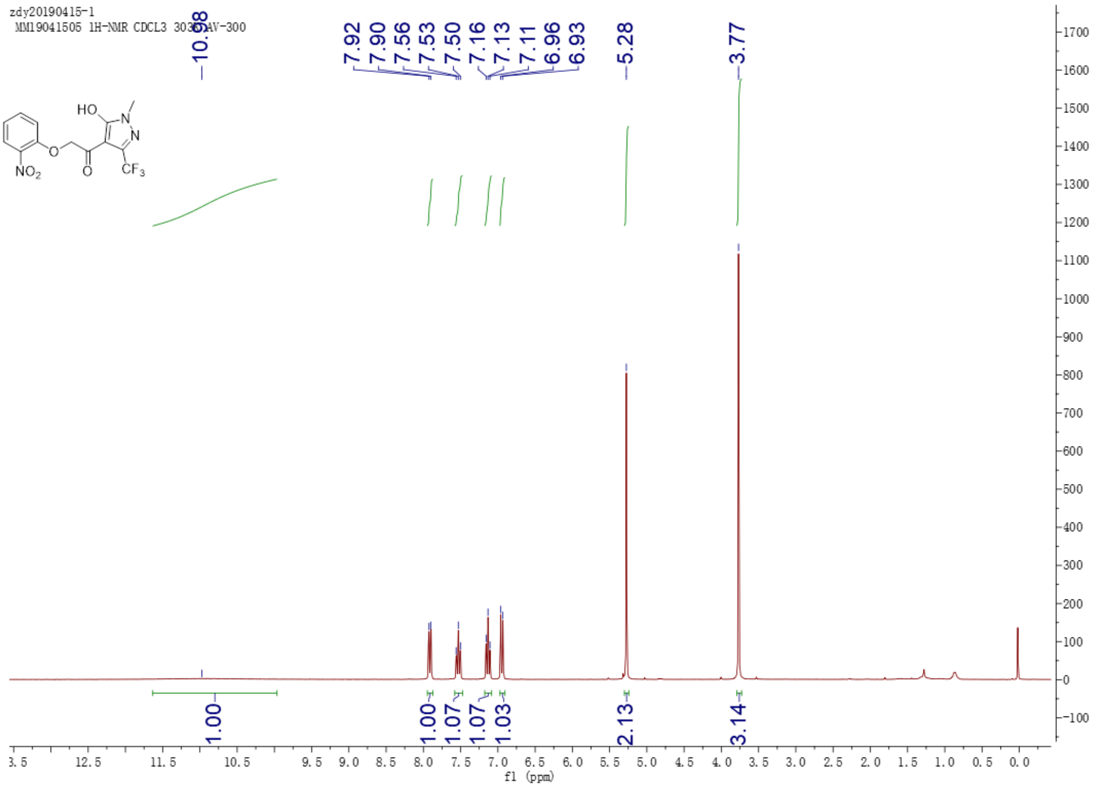

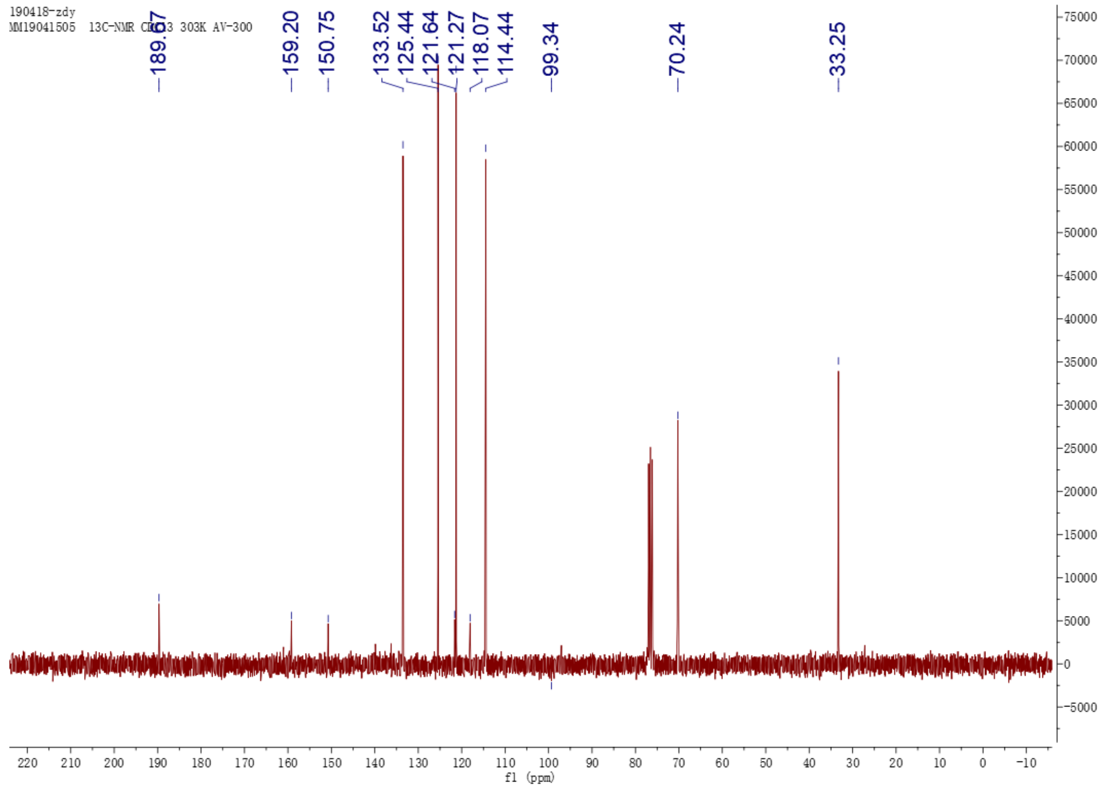

3-Hydroxy-2-(2-(4-nitrophenoxy)acetyl)cyclohex-2-en-1-one (I17):

Yield, 89%; white solid; ^{1}H NMR (300 MHz, DMSO) δ 16.39 (s, 1H), 8.15 (d, J = 9.1 Hz, 2H), 7.08 (d, J = 9.1 Hz, 2H), 5.44 (s, 2H), 2.58 – 2.51 (m, 4H), 2.06 – 1.85 (m,

2H). ^{13}C NMR (75 MHz, DMSO) δ 196.08, 179.30, 163.79 142.49, 126.14, 115.52, 71.93, 19.20. HRMS m/z: calculated 292.07429 ($C_{14}H_{13}NO_6$ + H)$^+$, found 292.07571 ($C_{14}H_{13}NO_6$ + H)$^+$.

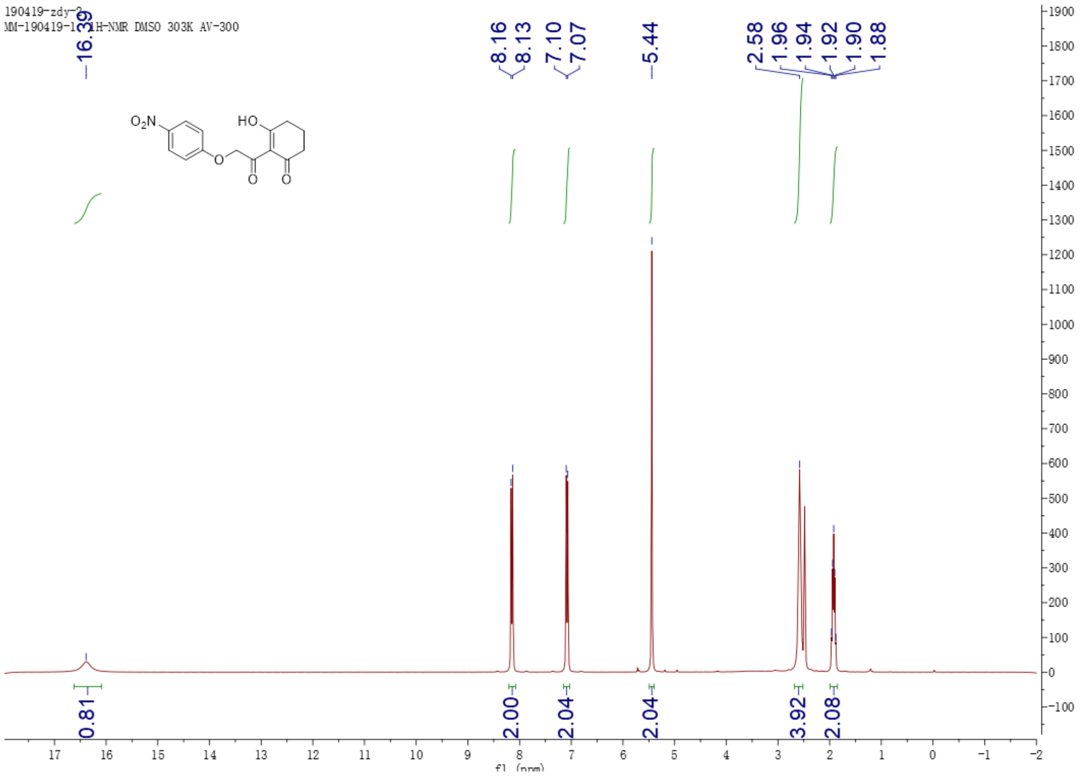

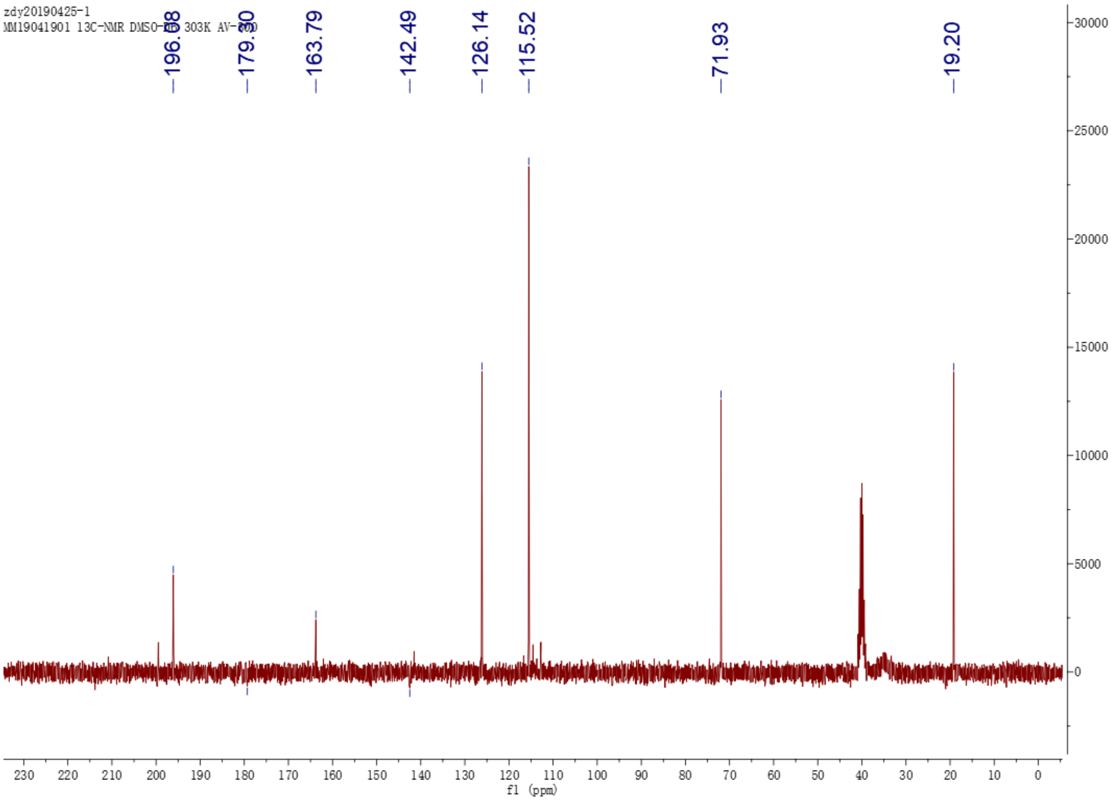

3-Hydroxy-5,5-dimethyl-2-(2-(4-nitrophenoxy)acetyl)cyclohex-2-en-1-one (I18): Yield, 80%; white solid; ^{1}H NMR (300 MHz, CDCl3) δ 16.76 (s, 1H), 8.20 (d, J = 9.2 Hz, 2H), 6.98 (d, J = 9.2 Hz, 2H), 5.41 (s, 2H), 2.62 (s, 2H), 2.42 (s, 2H), 1.14 (s, 6H). ^{13}C NMR (75 MHz, CDCl3) δ 198.75, 195.65, 163.10, 141.95, 125.84, 114.70, 71.78, 51.76, 45.32, 31.20, 28.12. HRMS m/z: calculated 320.10559 ($C_{16}H_{17}NO_6$ + H)$^+$, found 320.10529 ($C_{16}H_{17}NO_6$ + H)$^+$.

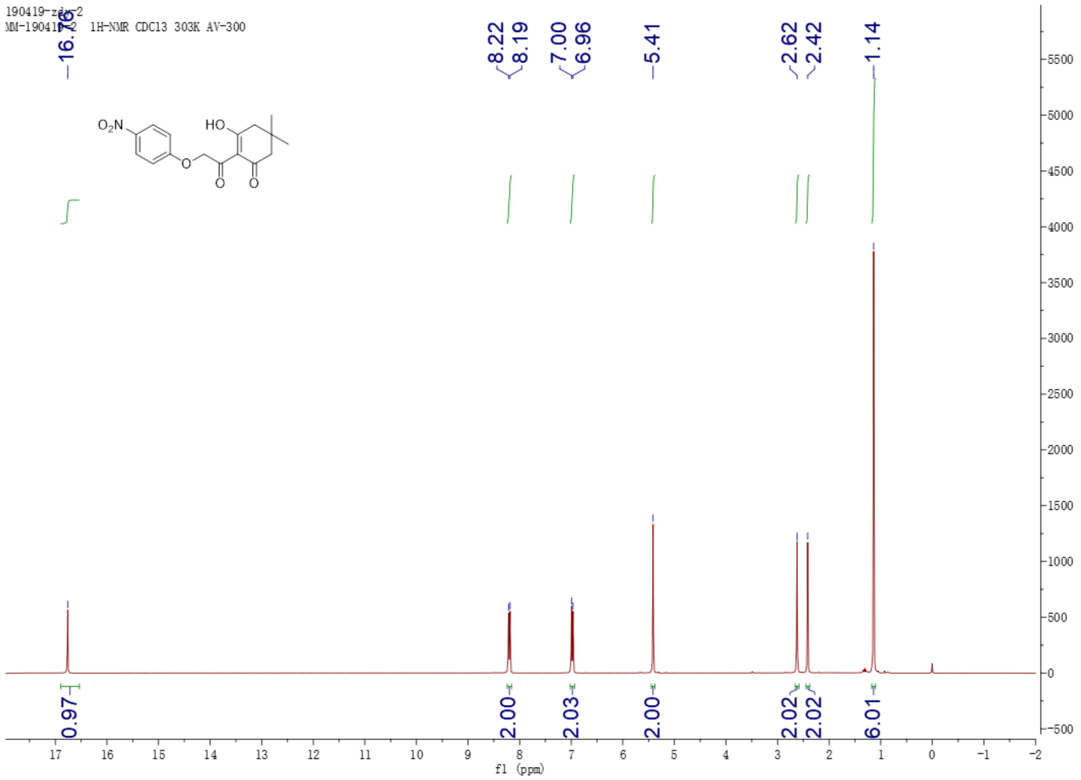

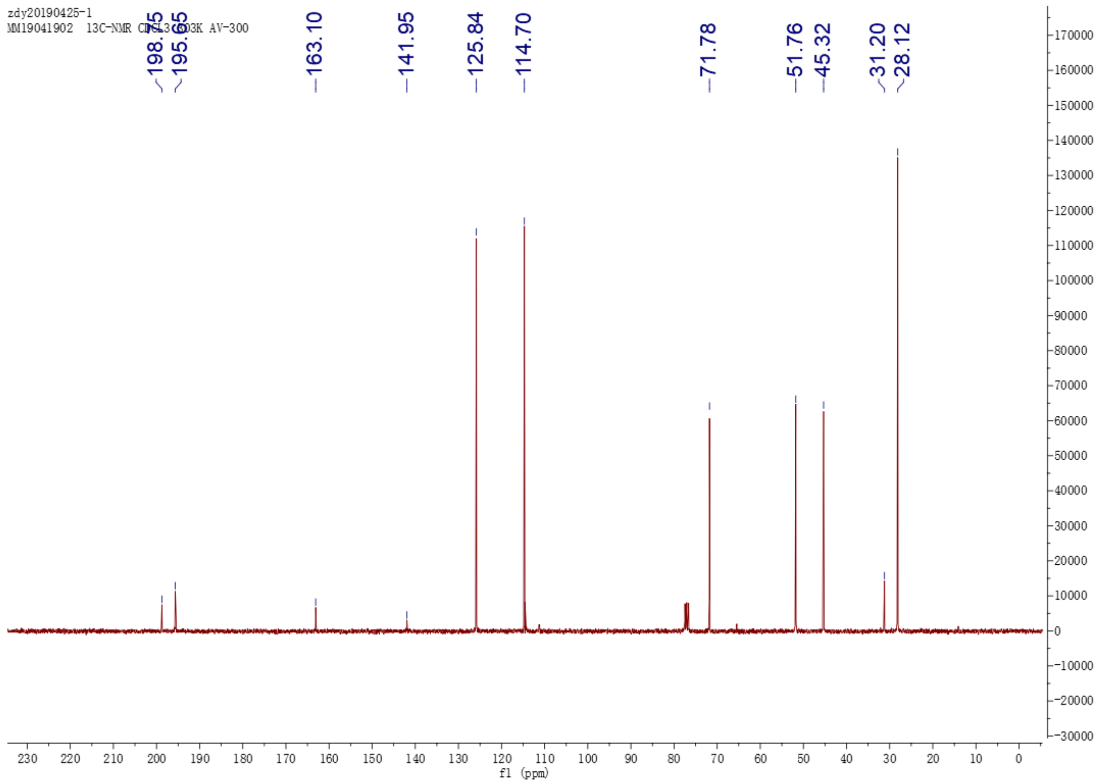

1-(5-Hydroxy-1,3-dimethyl-1*H*-pyrazol-4-yl)-2-(4-nitrophenoxy)ethan-1-one (I19):

Yield, 85%; white solid; ^{1}H NMR (300 MHz, DMSO) δ 11.29 (s, 1H), 8.14 (d, J = 9.1 Hz, 2H), 7.01 (d, J = 9.1 Hz, 2H), 5.32 (s, 2H), 3.34 (s, 3H), 2.28 (s, 3H). ^{13}C NMR (75 MHz, DMSO) δ 187.77, 164.25, 160.05, 147.93, 141.18, 126.09, 115.39, 102.51, 71.67, 30.97, 13.82. HRMS m/z: calculated 292.08552 $(C_{13}H_{13}N_3O_5 + H)^+$, found 292.08556 $(C_{13}H_{13}N_3O_5 + H)^+$.

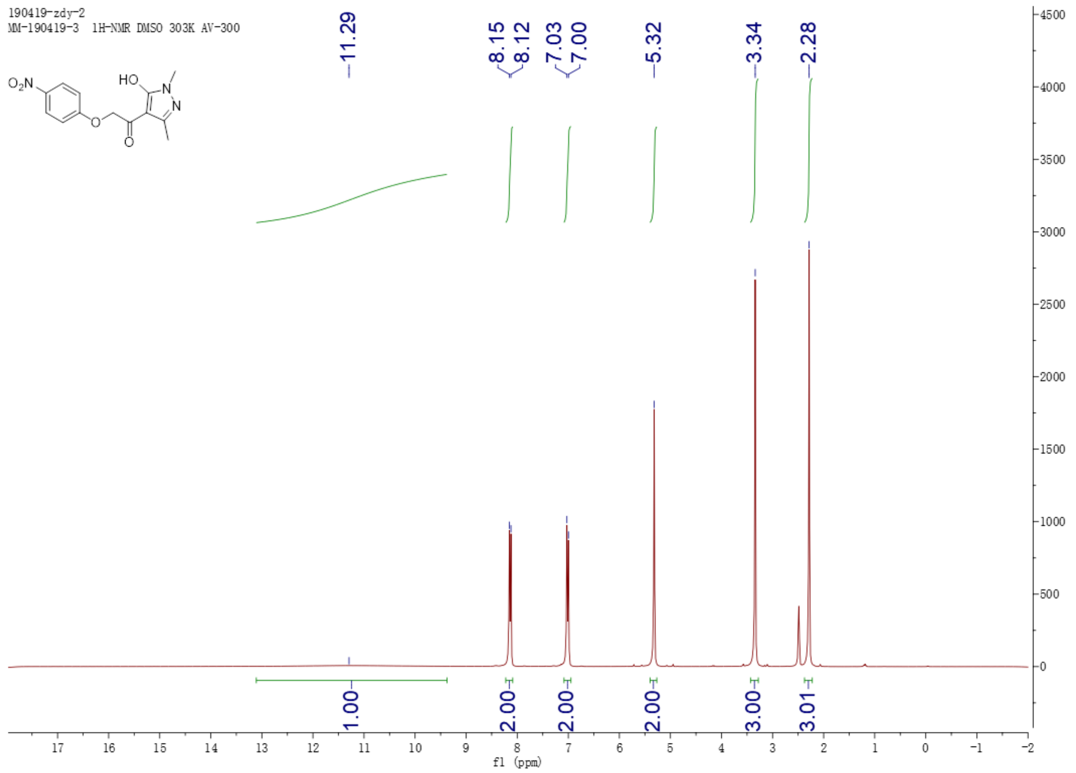

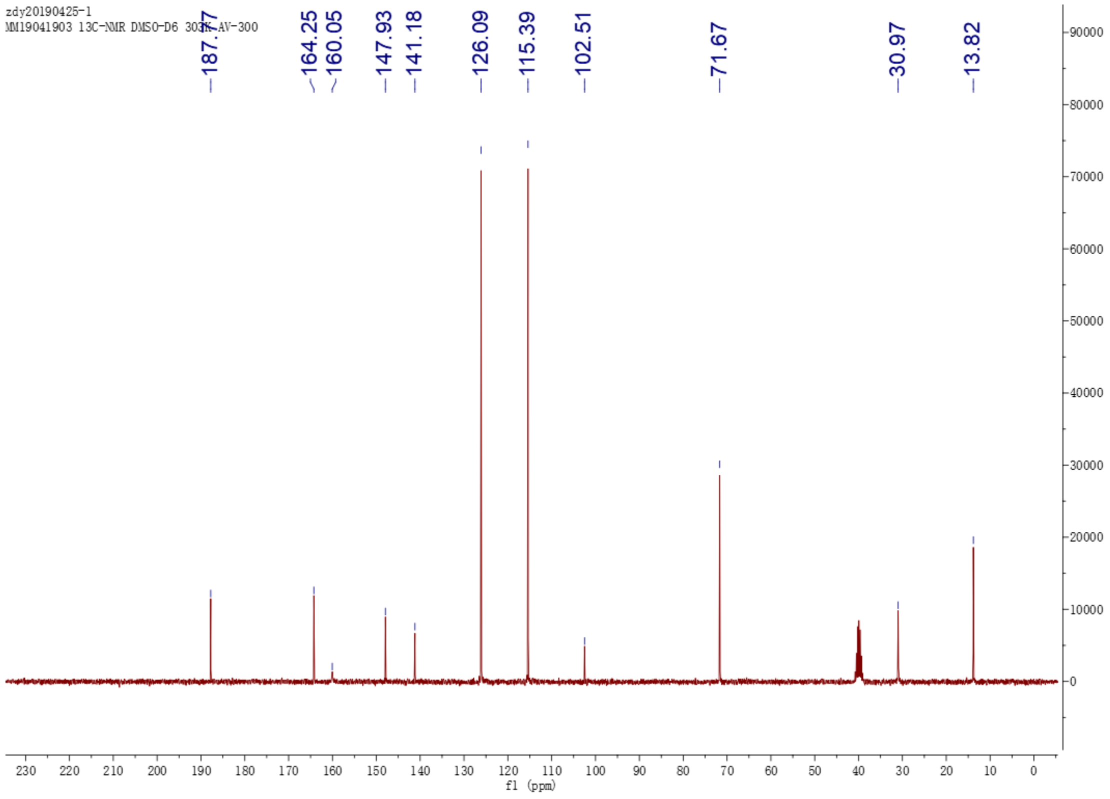

1-(3-Cyclopropyl-5-hydroxy-1-methyl-1*H*-pyrazol-4-yl)-2-(4-nitrophenoxy)ethan-1-one (I20):

Yield, 80%; white solid; ^{1}H NMR (300 MHz, DMSO) δ 8.15 (d, J = 9.1 Hz, 2H), 7.03

(d, J = 9.1 Hz, 2H), 5.36 (s, 2H), 3.34 (s, 3H), 2.55 (s, 1H), 0.89 (dd, J = 26.1, 5.5 Hz,4H). ^{13}C NMR (75 MHz, DMSO) δ 187.78, 164.28, 153.65, 141.19, 126.12, 115.42, 103.14, 71.68, 31.48, 8.89, 8.61. HRMS m/z: calculated 318.10117 ($C_{15}H_{15}N_3O_5$ + H)$^+$, found 318.10256 ($C_{15}H_{15}N_3O_5$ + H)$^+$.

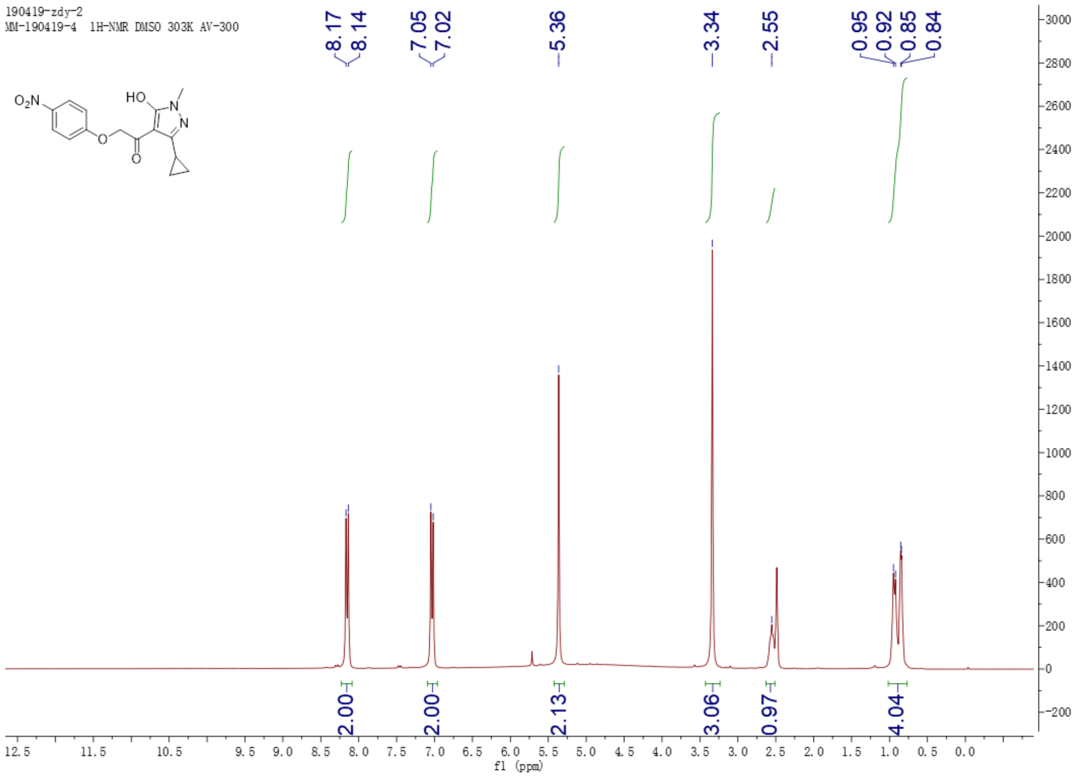

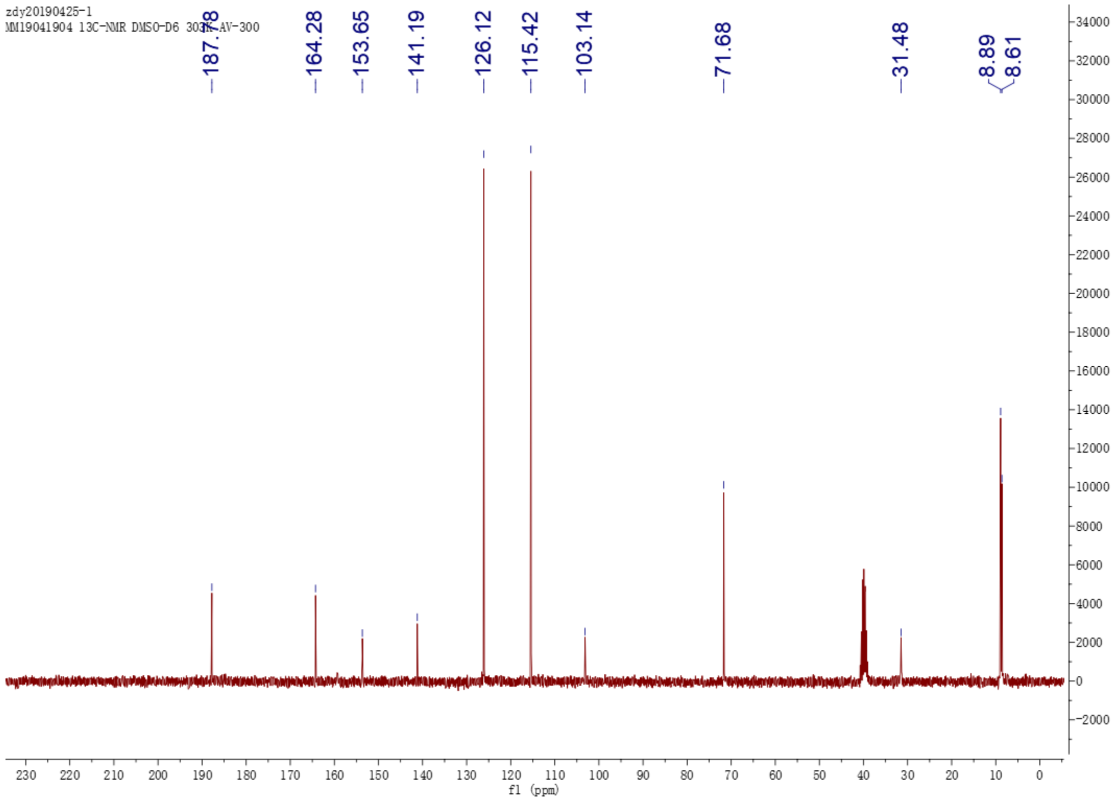

1-(5-Hydroxy-1-methyl-3-(trifluoromethyl)-1*H*-pyrazol-4-yl)-2-(4-nitrophenoxy)ethan-1-one (I21):

Yield, 89%; white solid; ^{1}H NMR (300 MHz, $CDCl_3$) δ 9.67 (s, 1H), 8.21 (d, J = 9.0 Hz, 2H), 6.97 (d, J = 9.0 Hz, 2H), 5.20 (s, 2H), 3.76 (s, 3H). ^{13}C NMR (75 MHz, DMSO) δ 190.38, 162.61, 159.75, 142.27, 125.89, 122.10, 118.53, 114.62, 69.77, 33.69. HRMS m/z: calculated 346.05726 $(C_{13}H_{10}F_3N_3O_5 + H)^+$, found 346.05711 $(C_{13}H_{10}F_3N_3O_5 + H)^+$.

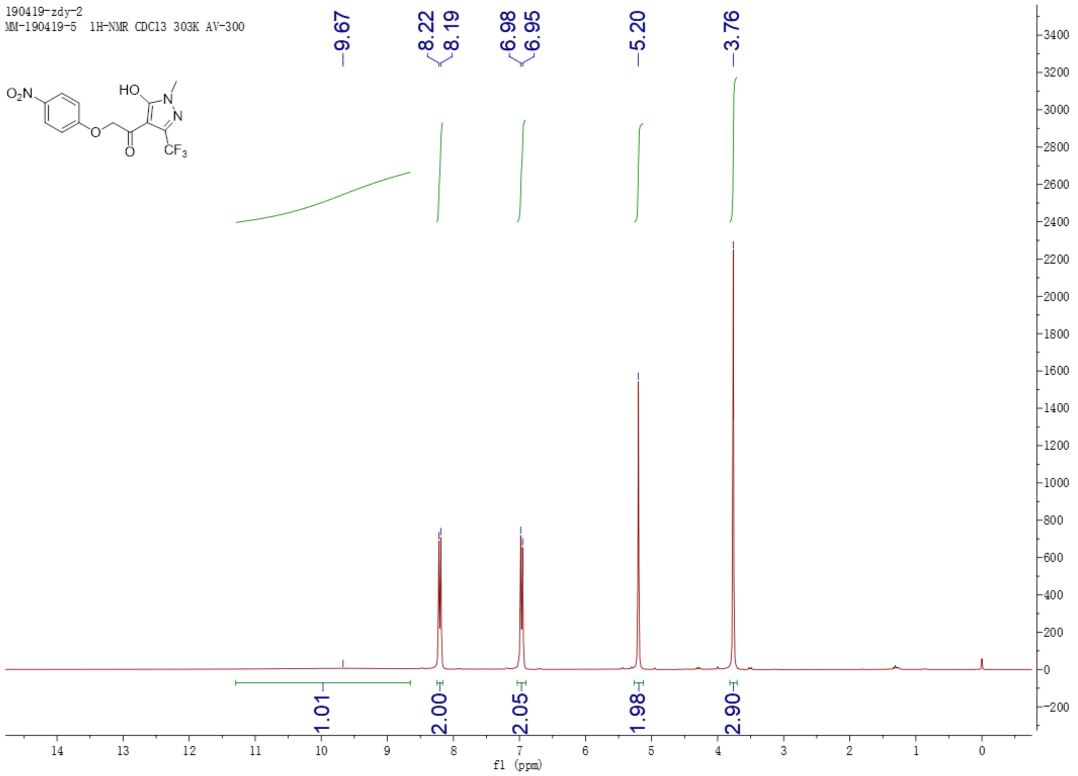

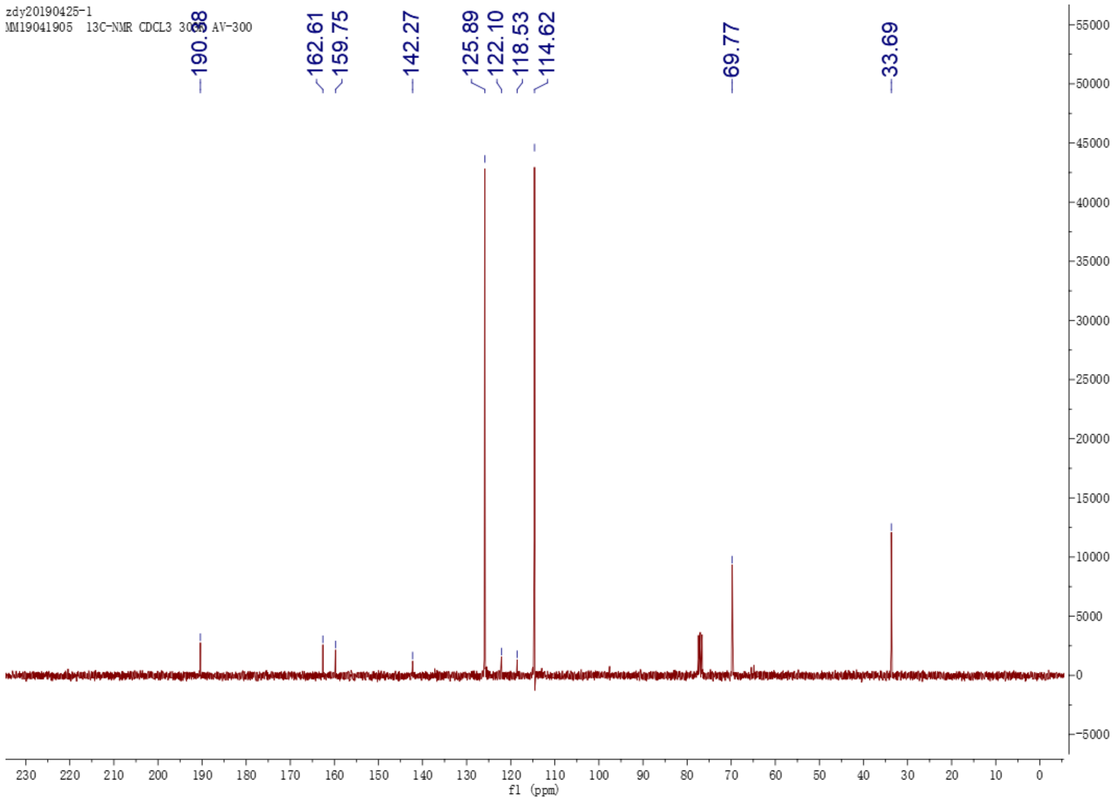

2-(2-(2-Chloro-4-nitrophenoxy)acetyl)-3-hydroxy-5,5-dimethylcyclohex-2-en-1-one (I22):

Yield, 75%; white solid; ^{1}H NMR (300 MHz, $CDCl_3$) δ 16.65 (s, 1H), 8.34 (s, 1H),

8.12 (d, J = 9.0 Hz, 1H), 6.88 (d, J = 9.0 Hz, 1H), 5.53 (s, 1H), 2.65 (s, 2H), 2.44 (s, 2H), 1.16 (s, 6H). ^{13}C NMR (75 MHz, $CDCl_3$) δ 202.93, 198.10, 197.84, 160.60, 131.98, 123.90, 117.27, 113.79, 74.14, 54.40, 48.00, 33.64, 30.65. HRMS m/z: calculated 354.06661 ($C_{16}H_{16}ClNO_6$ + H)$^+$, found 354.06688 ($C_{16}H_{16}ClNO_6$ + H)$^+$.

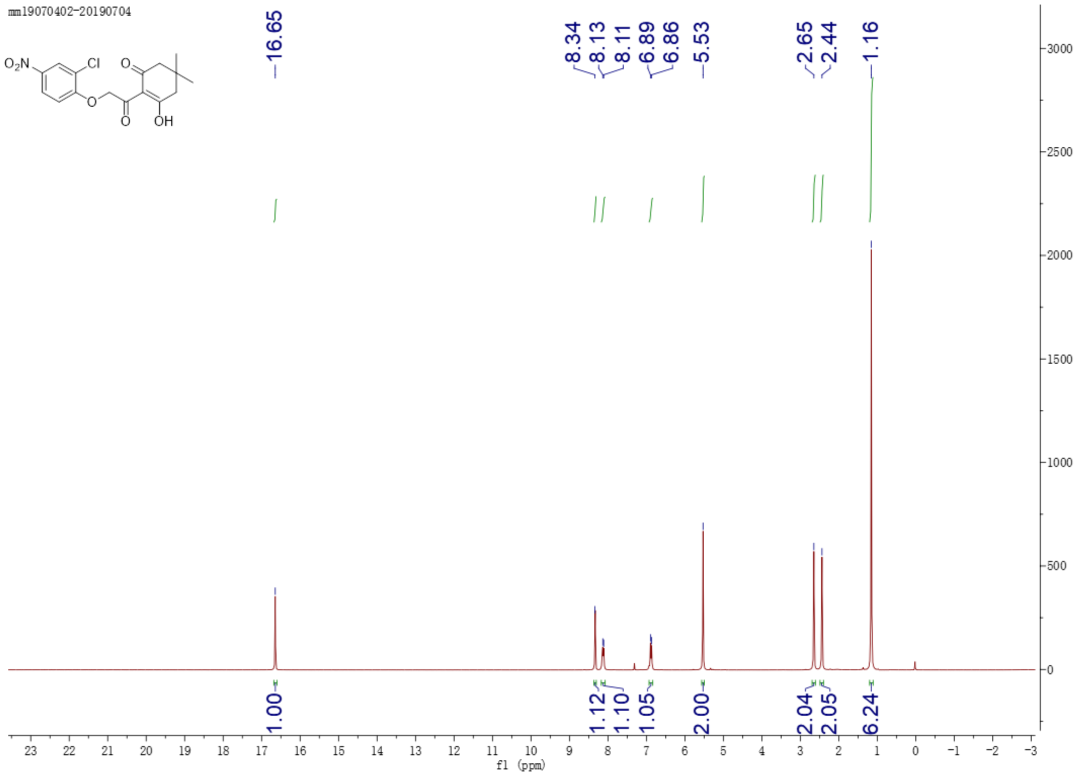

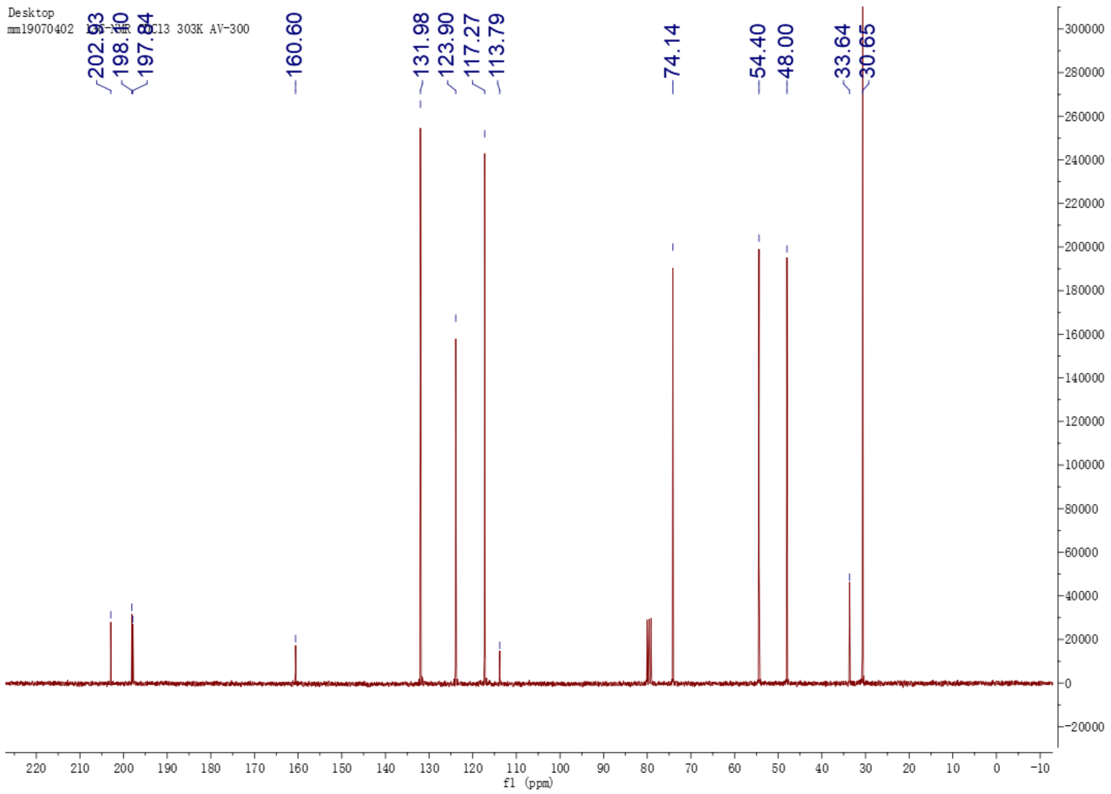

2-(2-Chloro-4-nitrophenoxy)-1-(5-hydroxy-1,3-dimethyl-1*H*-pyrazol-4-yl)ethan-1-one (I23):

Yield, 68%; white solid; ^{1}H NMR (300 MHz, DMSO) δ 8.58 (s, 1H), 8.29 (s, 1H), 8.12 (d, J = 9.0 Hz, 1H), 7.10 (d, J = 9.0 Hz, 1H), 5.50 (s, 2H), 3.38 (s, 3H), 2.31 (s, 3H). ^{13}C NMR (75 MHz, DMSO) δ 192.82, 161.65, 151.88, 134.37, 133.48, 128.57, 123.01, 120.54, 106.66, 75.31, 69.12, 34.99, 17.87. HRMS m/z: calculated 326.04655 $(C_{13}H_{12}ClN_3O_5 + H)^+$, found 326.04659 $(C_{13}H_{12}ClN_3O_5 + H)^+$.

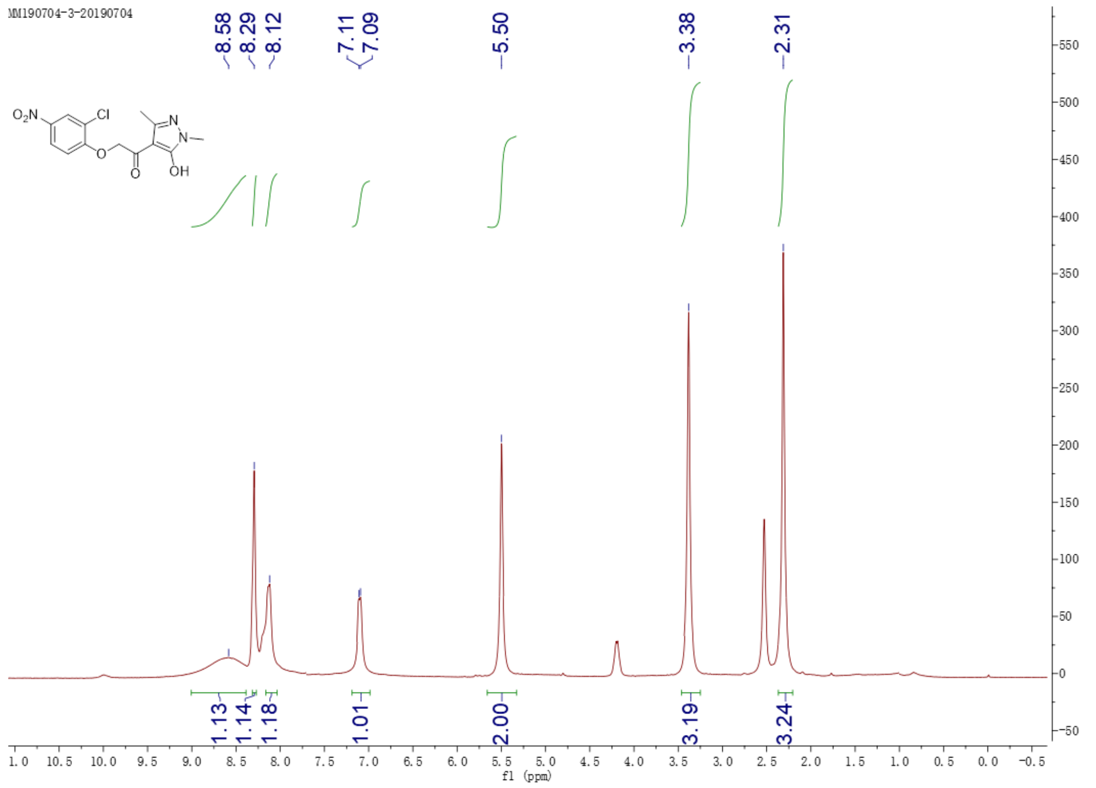

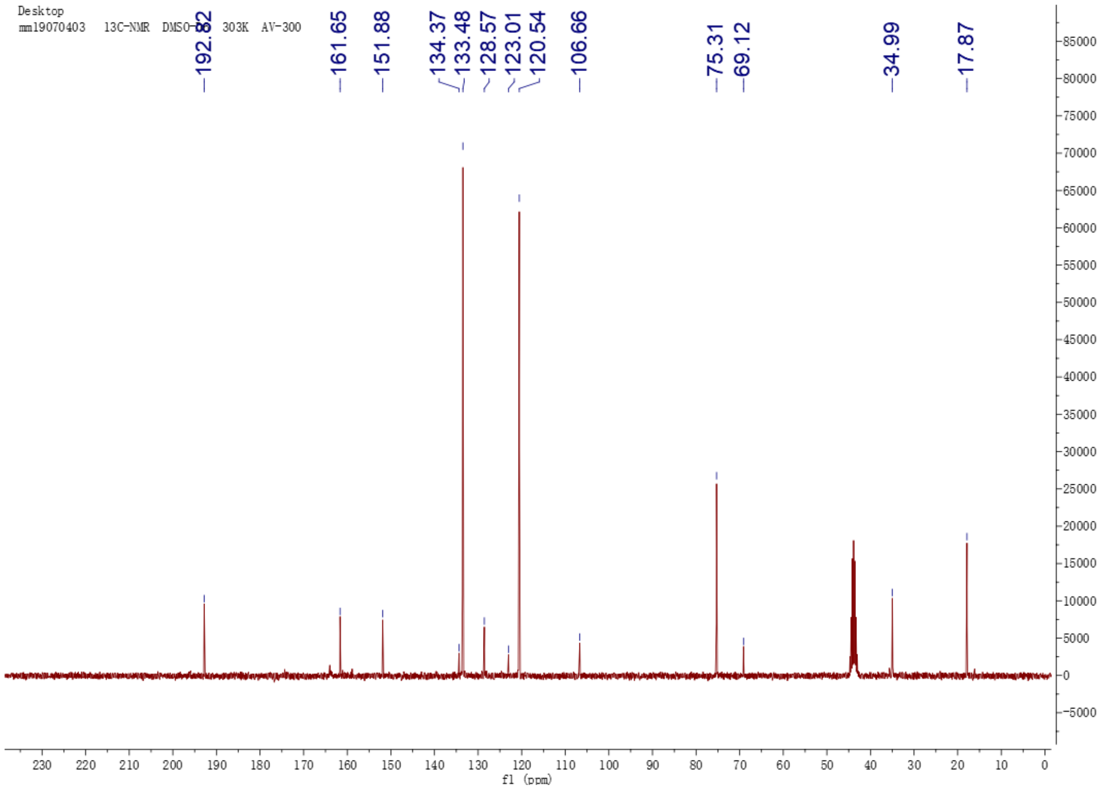

2-(2-Chloro-4-nitrophenoxy)-1-(3-cyclopropyl-5-hydroxy-1-methyl-1*H*-pyrazol-4-yl)ethan-1-one (I24):

Yield, 62%; white solid; ^{1}H NMR (300 MHz, DMSO) δ 8.34 (s, 1H), 8.17 (d, J = 9.2

Hz, 1H), 7.14 (d, J = 9.2 Hz, 1H), 5.55 (s, 2H), 3.38 (s, 2H), 2.59-2.56 (m, 1H), 1.01-0.97 (m, 2H), 0.90-0.87 (m, 1H). ^{13}C NMR (75 MHz, $CDCl_3$) δ 192.23, 160.04, 154.78, 151.84, 131.63, 128.64, 124.44, 123.45, 114.93, 102.35, 72.09, 33.56, 10.80, 7.47. HRMS m/z: calculated 352.06220 ($C_{15}H_{14}ClN_3O_5$ + H)$^+$, found 352.06219 ($C_{15}H_{14}ClN_3O_5$ + H)$^+$.

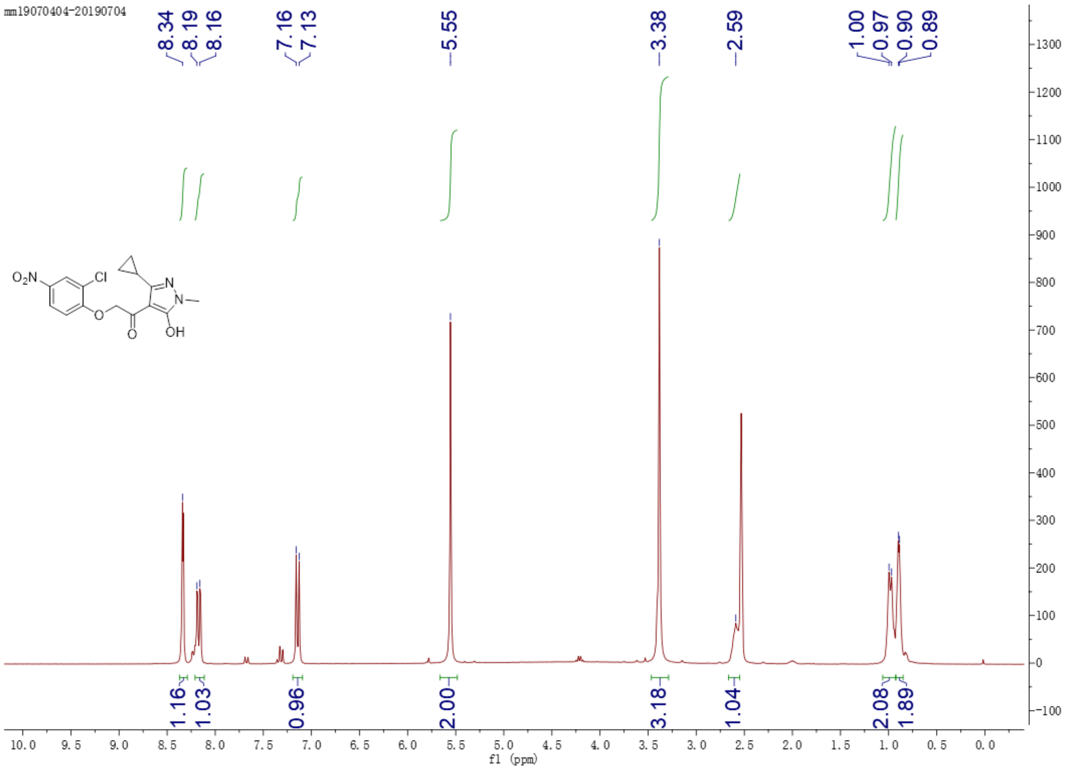

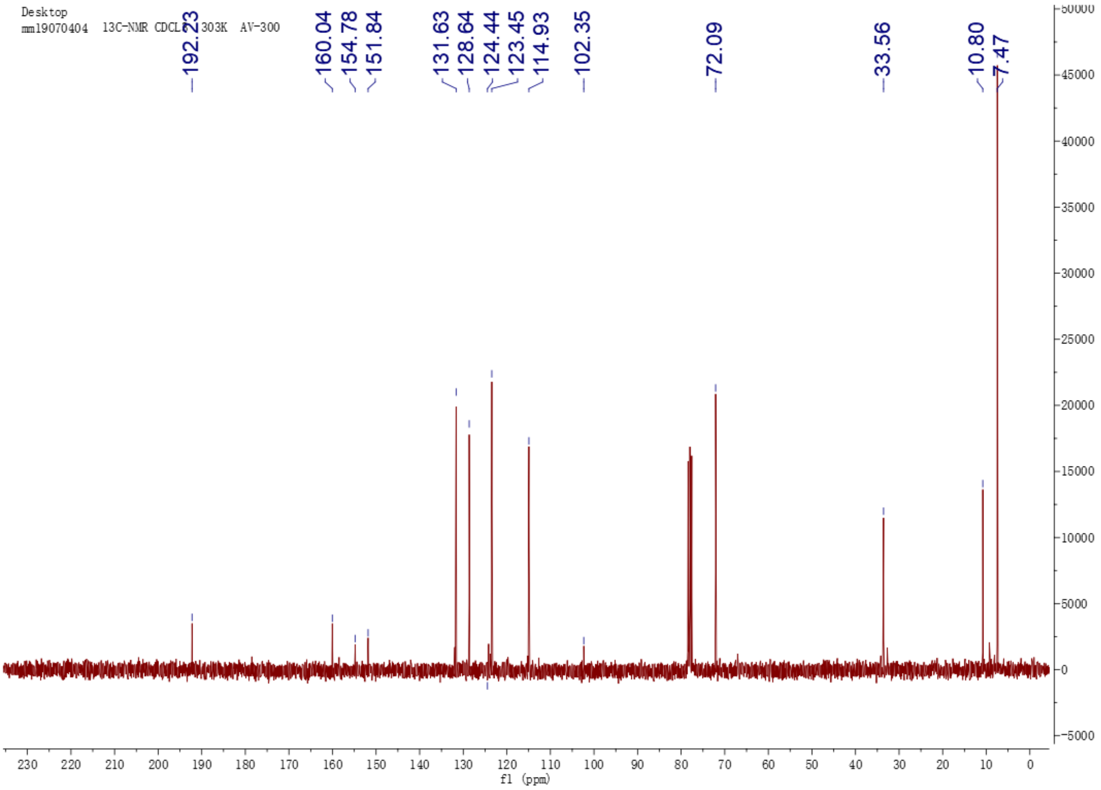

2-(2-Chloro-4-nitrophenoxy)-1-(5-hydroxy-1-methyl-3-(trifluoromethyl)-1*H*-pyrazol-4-yl)ethan-1-one (I25):

Yield, 58%; white solid; ^{1}H NMR (300 MHz, DMSO) δ 11.09 (s, 1H), 8.22 (s, 1H), 8.06 (d, J = 9.2 Hz, 1H), 7.07 (d, J = 9.2 Hz, 1H), 5.46 (s, 2H), 3.61 (s, 3H). ^{13}C NMR (75 MHz, DMSO) δ 153.42, 129.60, 128.23, 124.68, 122.51, 115.37, 114.56, 71.85, 31.06, 13.94. HRMS m/z: calculated 380.01828 $(C_{13}H_{9}ClF_{3}N_{3}O_{5} + H)^{+}$, found 380.01781 $(C_{13}H_{9}ClF_{3}N_{3}O_{5} + H)^{+}$.

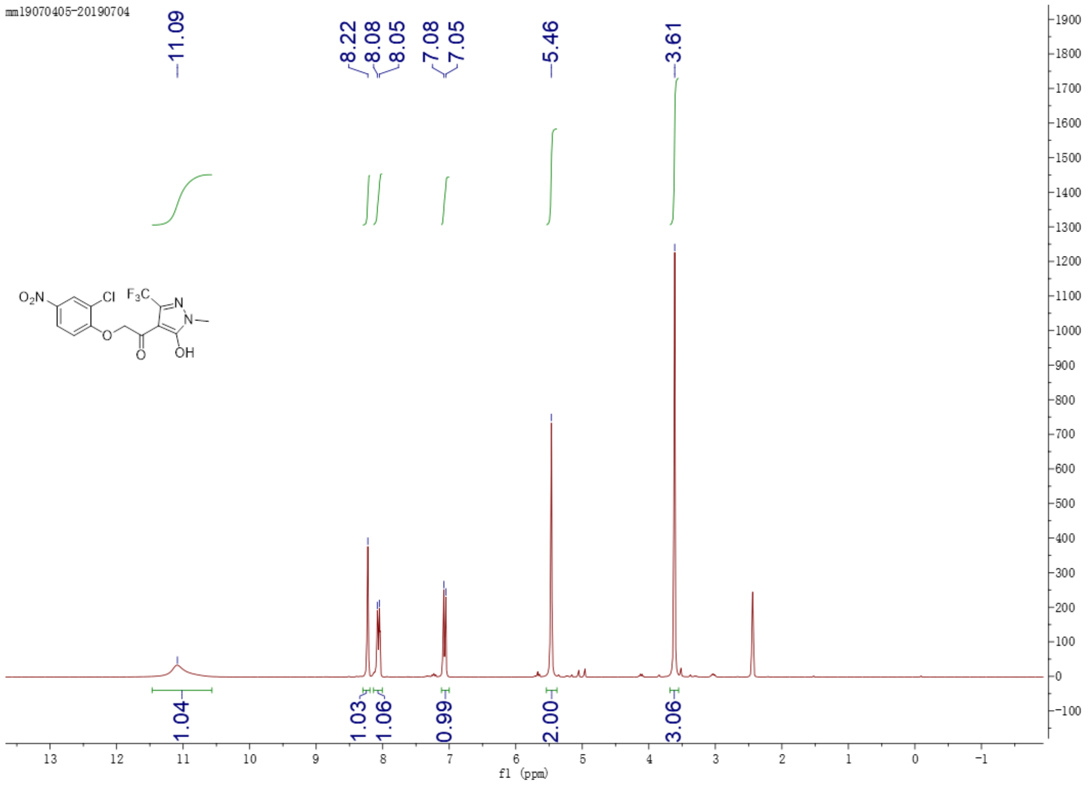

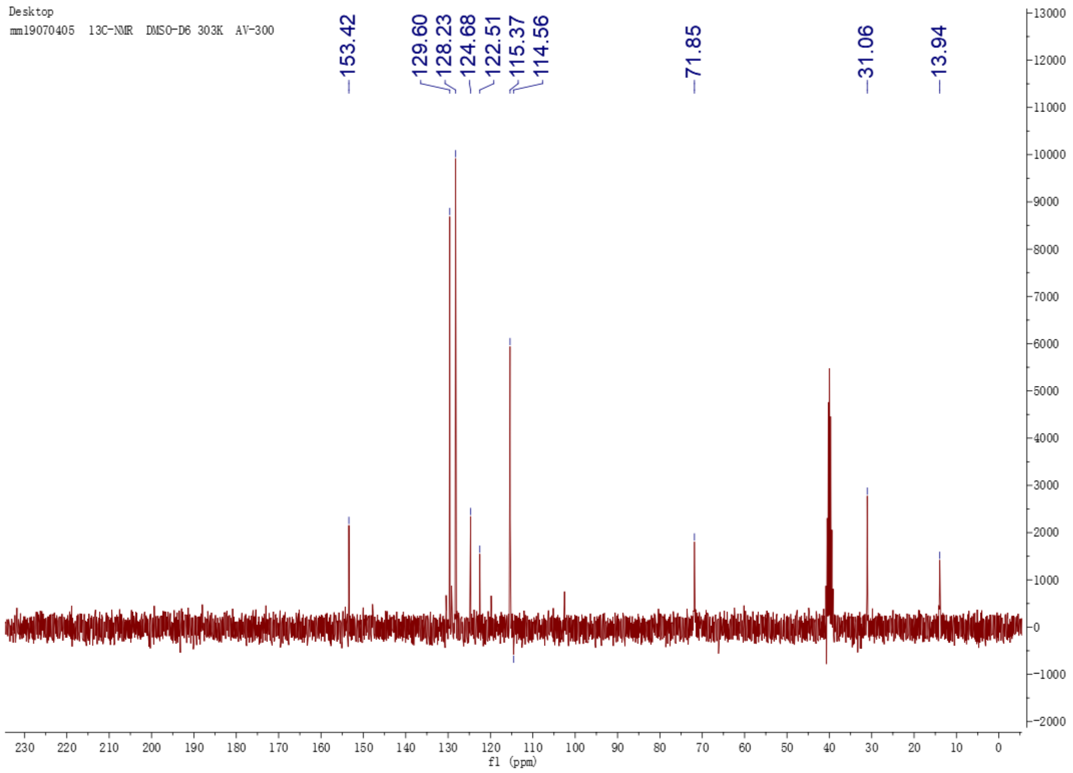

2-(2-(5-Chloro-2-(2,4-dichlorophenoxy)phenoxy)acetyl)-3-hydroxycyclohex-2-en-1-one (I26):

Yield, 77%; white solid; ^{1}H NMR (300 MHz, $CDCl_3$) δ 16.90 (s, 1H), 7.42 (d, J = 1.6

Hz, 1H), 7.13 (d, J = 8.7 Hz, 1H), 7.01 – 6.70 (m, 4H), 5.32 (s, 2H), 2.71 (t, J = 6.0 Hz, 2H), 2.50 (t, J = 6.1 Hz, 2H), 2.18 – 1.88 (m, 2H). ^{13}C NMR (75 MHz, $CDCl_3$) δ 199.69, 196.73, 195.76, 151.96, 150.12, 143.30, 130.12, 128.17, 127.76, 124.76, 121.92, 121.71, 118.88, 115.39, 112.27, 72.57, 37.90, 31.81, 19.19. HRMS m/z: calculated 440.99851 ($C_{20}H_{15}Cl_3O_5$ + H)$^+$, found 440.99878 ($C_{20}H_{15}Cl_3O_5$ + H)$^+$.

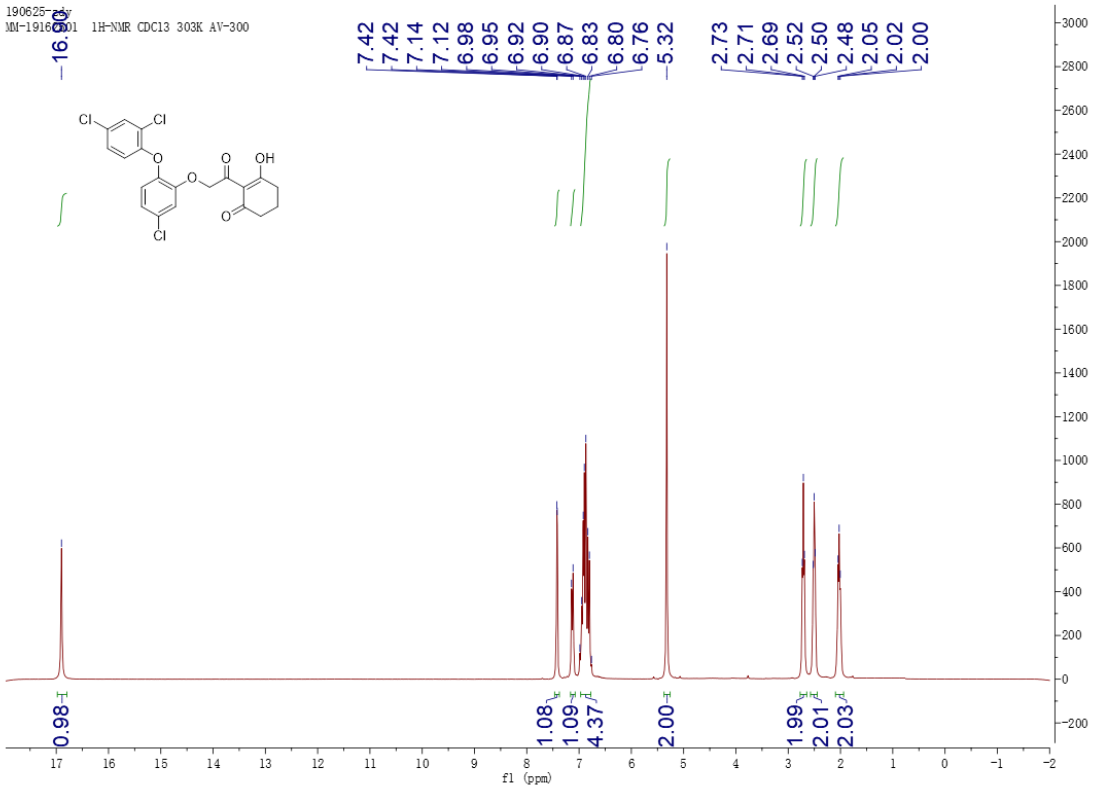

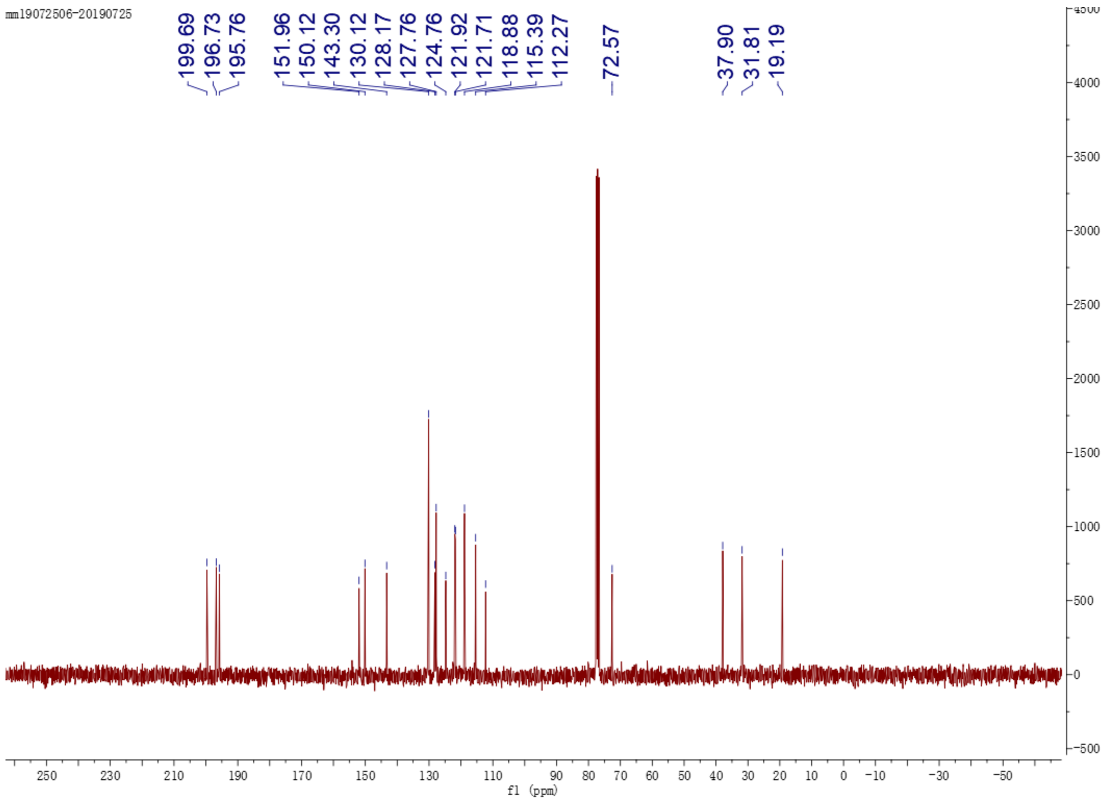

2-(2-(5-Chloro-2-(2,4-dichlorophenoxy)phenoxy)acetyl)-3-hydroxy-5,5-dimethylcyclohex-2-en-1-one (I27):

Yield, 80%; white solid; ^{1}H NMR (300 MHz, $CDCl_3$) δ 7.40 (d, J = 2.5 Hz, 1H), 7.10 (dd, J = 8.8, 2.5 Hz, 1H), 6.86 (s, 3H), 6.78 (d, J = 8.8 Hz, 1H), 5.25 (s, 2H), 2.34 (s, 4H), 1.05 (s, 6H). ^{13}C NMR (75 MHz, DMSO) δ 191.14, 172.91, 162.11, 156.43, 155.36, 154.26, 153.44, 145.29, 132.96, 131.71, 130.15, 126.53, 125.89, 121.80, 118.07, 106.12, 74.69, 48.91, 34.57, 11.96, 11.63. HRMS m/z: calculated 469.02981 $(C_{22}H_{19}Cl_3O_5 + H)^+$, found 469.02975 $(C_{22}H_{19}Cl_3O_5 + H)^+$.

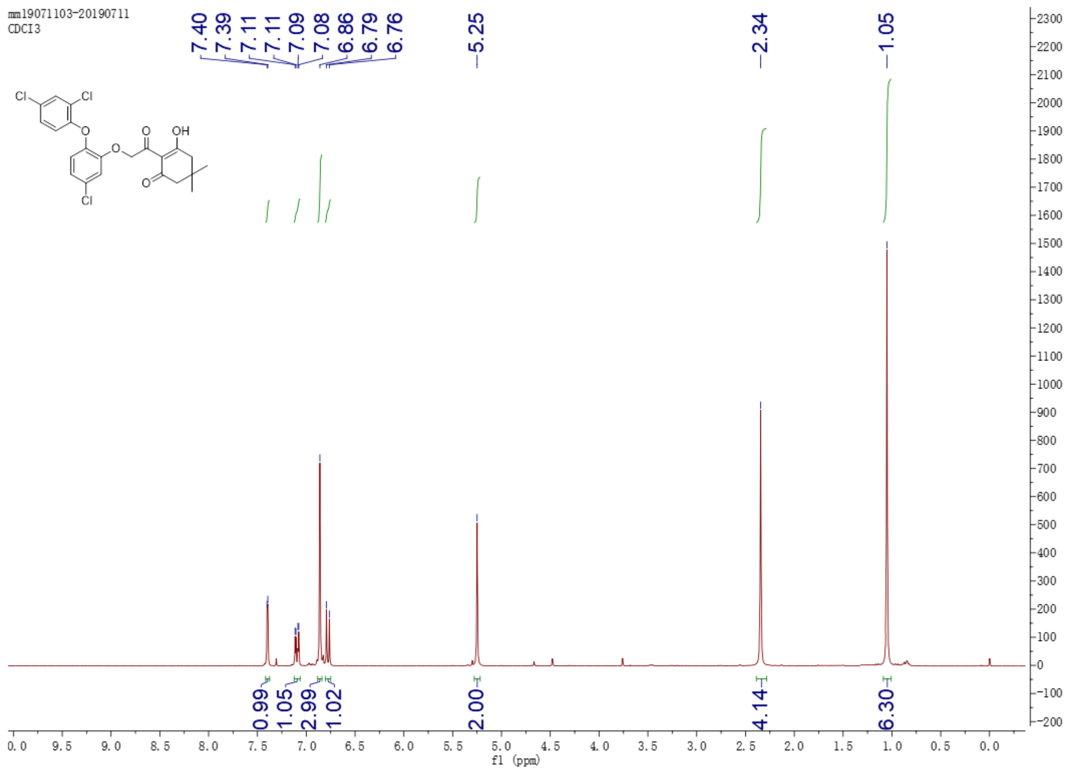

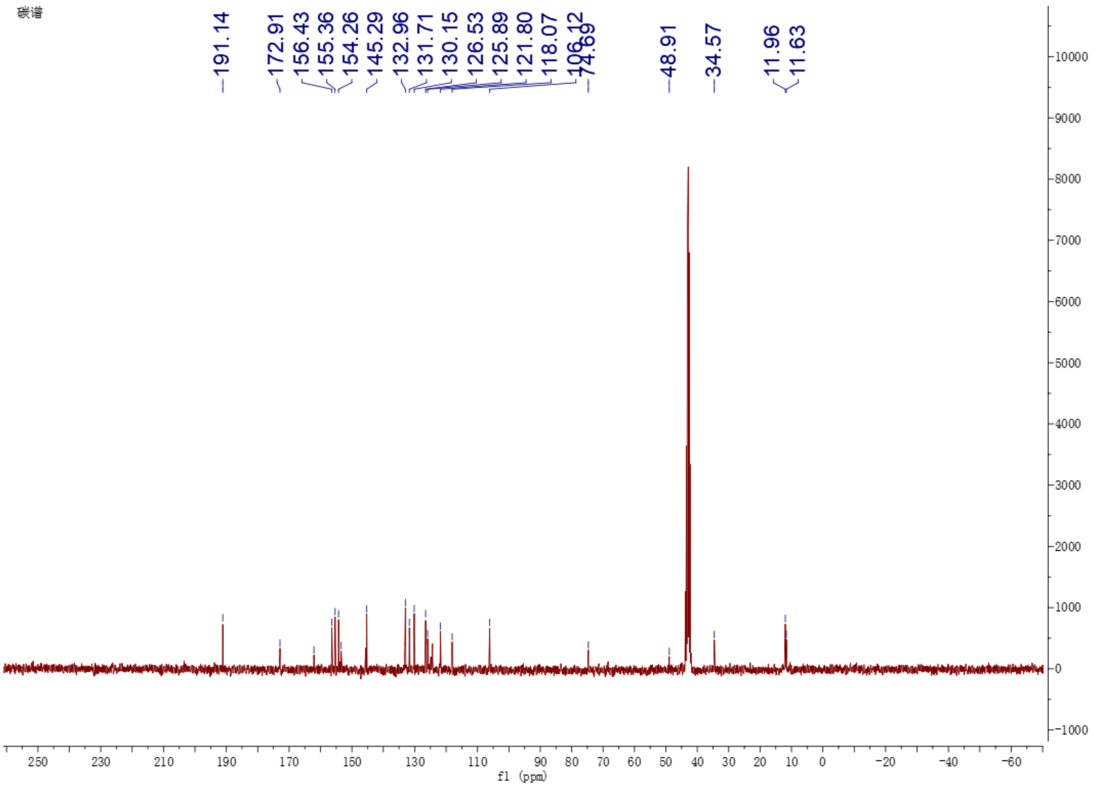

2-(5-Chloro-2-(2,4-dichlorophenoxy)phenoxy)-1-(5-hydroxy-1,3-dimethyl-1*H*-pyrazol-4-yl)ethan-1-one (I28):

Yield, 75%; white solid; ^{1}H NMR (300 MHz, DMSO) δ 9.97 (s, 1H), 7.65 (d, J = 2.3

Hz, 1H), 7.30 (dd, J = 8.8, 2.3 Hz, 1H), 7.02 (q, J = 8.1 Hz, 3H), 6.87 (d, J = 8.8 Hz, 1H), 5.25 (s, 2H), 3.37 (s, 3H), 2.30 (s, 3H). ^{13}C NMR (75 MHz, DMSO) δ 189.16, 170.94, 160.13, 154.45, 153.39, 152.28, 143.31, 129.73, 128.18, 124.55, 119.83, 104.14, 72.71, 46.93, 32.59, 9.98. HRMS m/z: calculated 441.00974 ($C_{19}H_{15}Cl_3N_2O_4$ + H)$^+$, found 441.01053 ($C_{19}H_{15}Cl_3N_2O_4$ + H)$^+$.

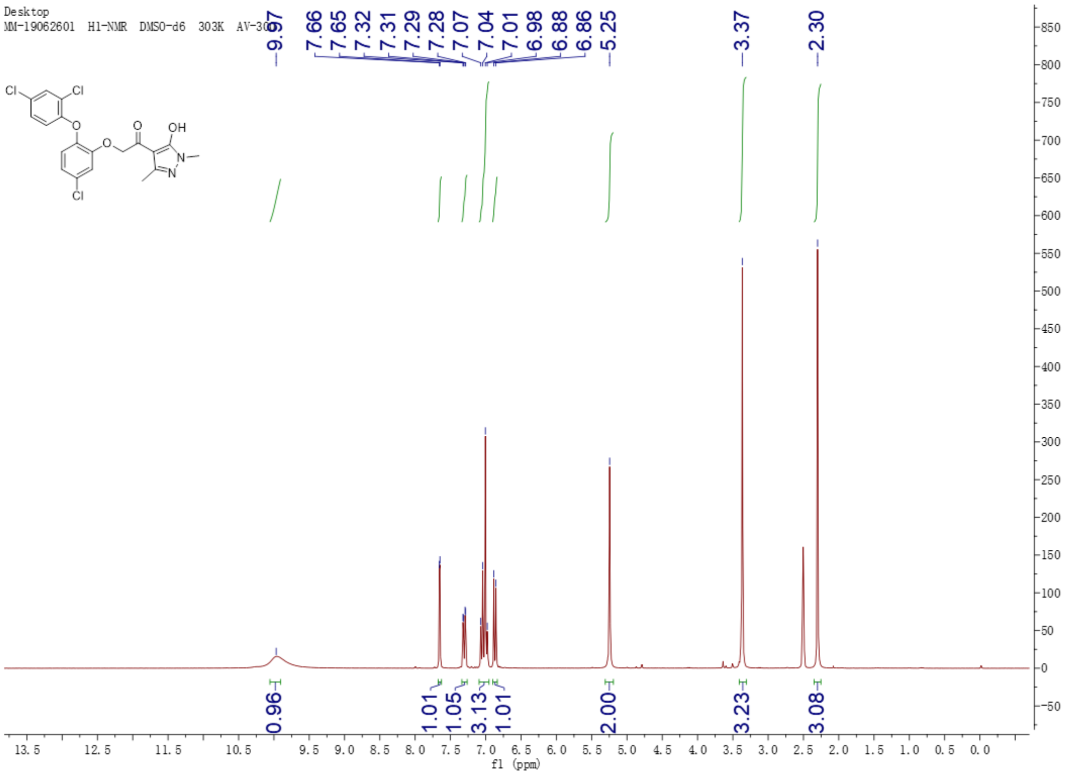

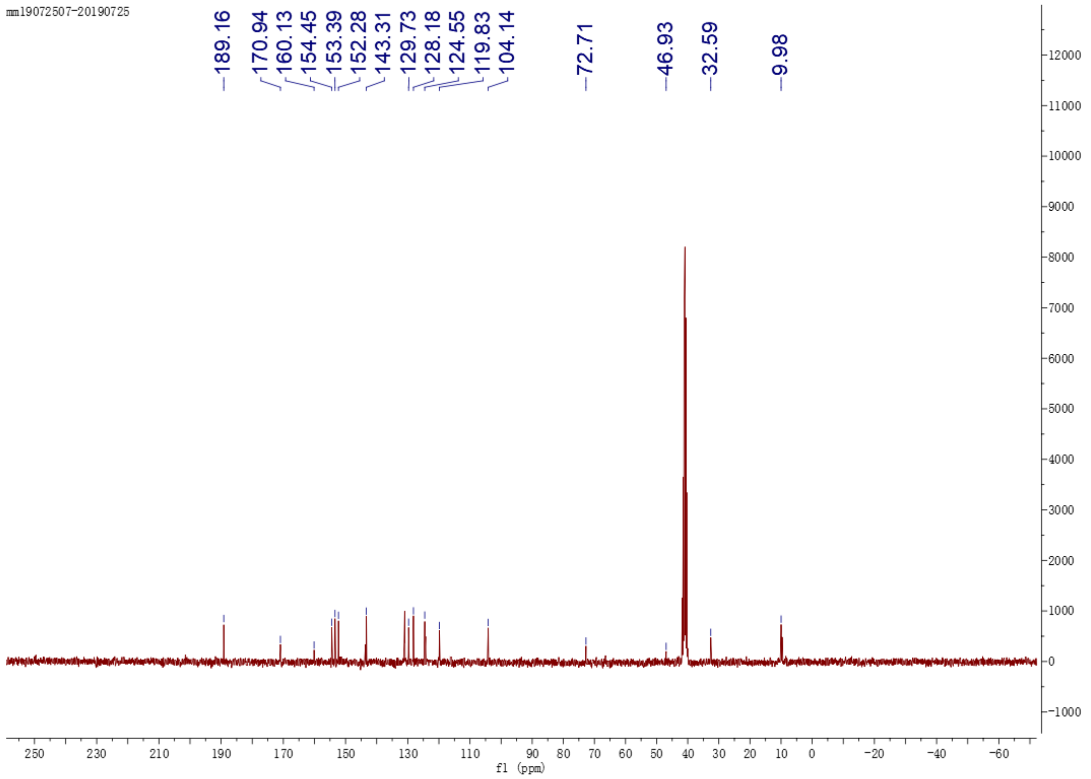

2-(5-Chloro-2-(2,4-dichlorophenoxy)phenoxy)-1-(3-cyclopropyl-5-hydroxy-1-methyl-1*H*-pyrazol-4-yl)ethan-1-one (I29):

Yield, 71%; white solid; ^{1}H NMR (300 MHz, $CDCl_3$) δ 9.78 (s, 1H), 7.45 (d, J = 2.5 Hz, 1H), 7.17 (dd, J = 8.8, 2.5 Hz, 1H), 7.04 – 6.96 (m, 3H), 6.84 (d, J = 2.0 Hz, 1H), 5.25 (s, 2H), 3.64 (s, 3H), 2.17 – 1.84 (m, 1H), 1.03 (d, J = 8.1 Hz, 4H). ^{13}C NMR (75 MHz, $CDCl_3$) δ 188.18, 169.96, 159.15, 153.47, 152.41, 151.30, 142.33, 128.75, 127.20, 123.57, 118.85, 115.11, 103.16, 71.73, 45.95, 31.61, 9.00. HRMS m/z: calculated 467.03423 ($C_{21}H_{17}Cl_3N_2O_4$ + H)$^+$, found 467.03312 ($C_{21}H_{17}Cl_3N_2O_4$ + H)$^+$

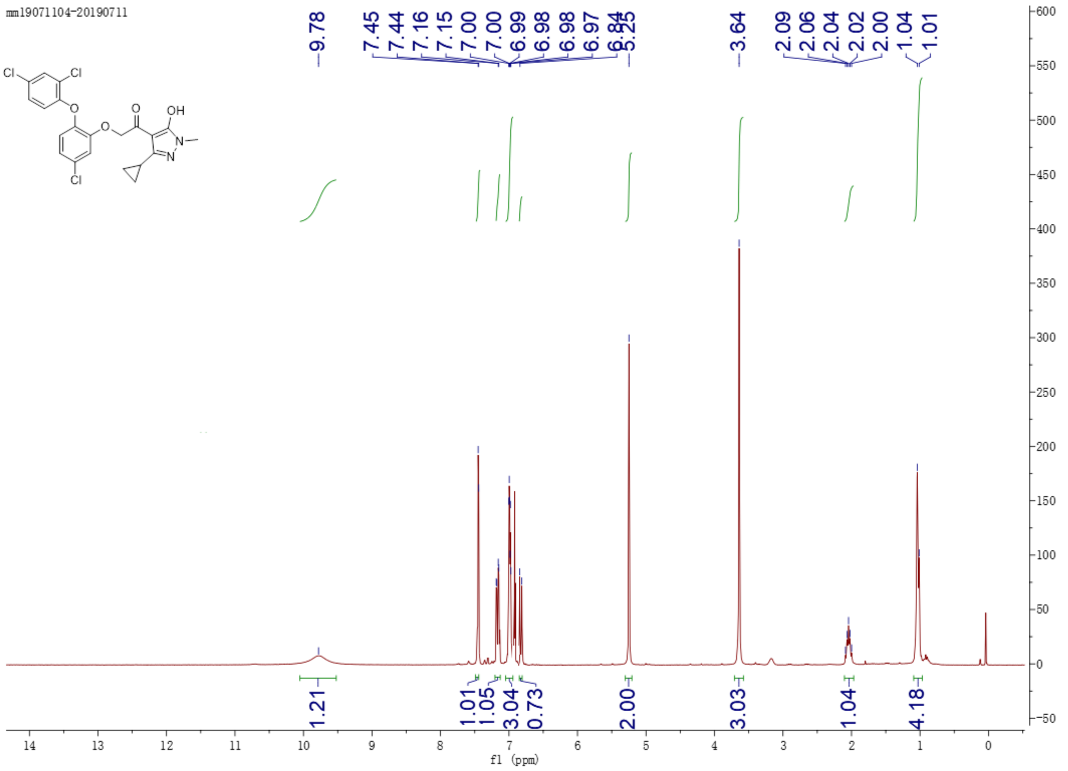

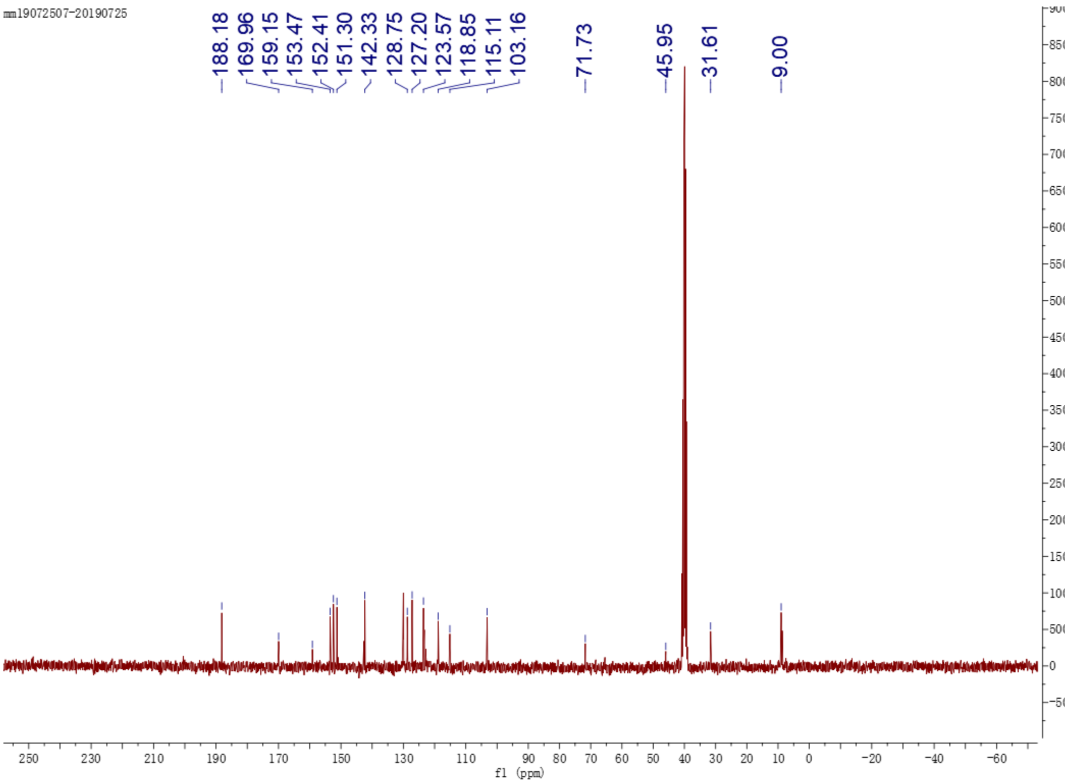

2-(2-(2,4-Dimethylphenoxy)acetyl)-3-hydroxycyclohex-2-en-1-one (I30): Yield, 85%; white solid; ^{1}H NMR (300 MHz, $CDCl_3$) δ 17.29 (s, 1H), 7.00 (s, 1H), 6.92 (d, J = 7.7 Hz, 1H), 6.63 (d, J = 8.2 Hz, 1H), 5.33 (s, 2H), 2.71 (t, J = 6.3 Hz,

2H), 2.59 – 2.48 (m, 2H), 2.32 (s, 3H), 2.28 (s, 3H), 2.12 – 1.94 (m, 2H). ^{13}C NMR (75 MHz, $CDCl_3$) δ 203.00, 197.55, 190.57, 154.64, 130.52, 130.40, 126.83, 126.40, 113.68, 108.91, 76.24, 36.74, 29.40, 21.23, 19.99, 16.02. HRMS m/z: calculated 275.12051 ($C_{16}H_{18}O_4$ + H)$^+$, found 275.12161 ($C_{16}H_{18}O_4$ + H)$^+$.

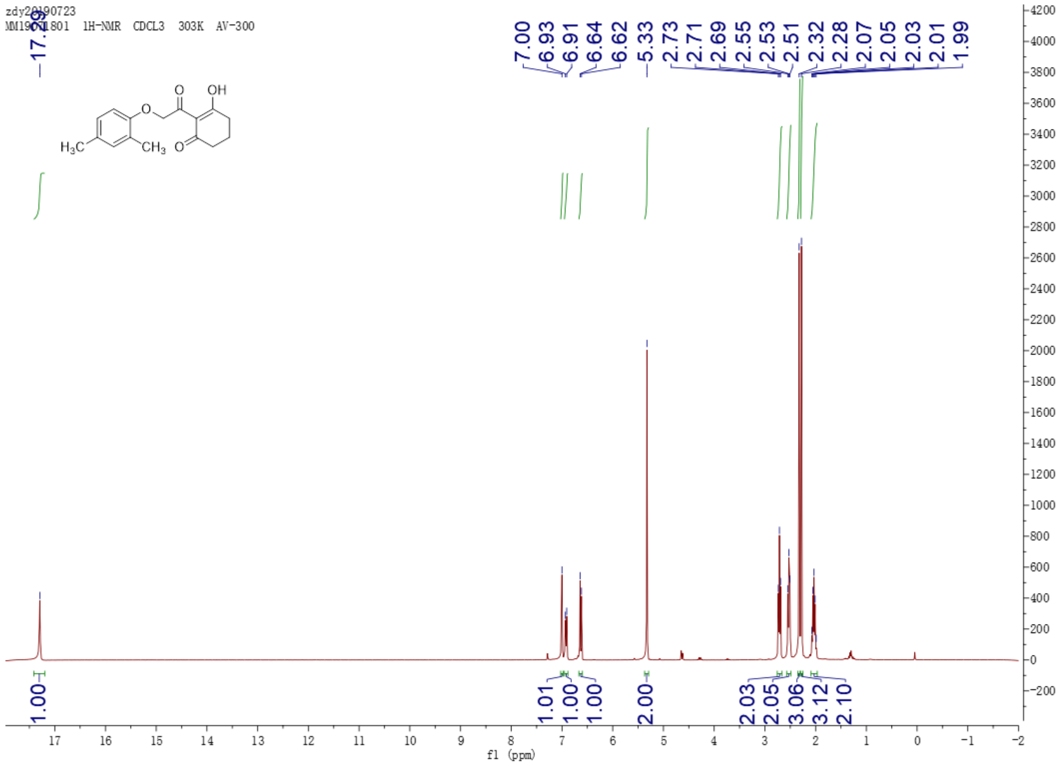

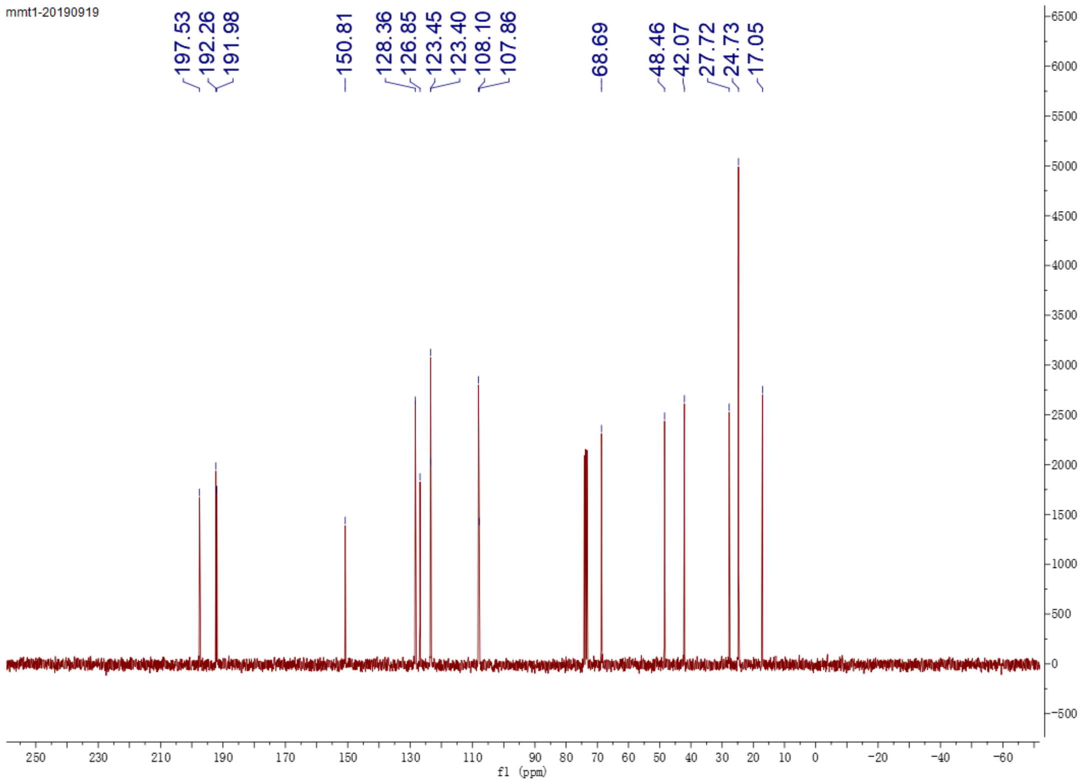

2-(2-(2,4-Dimethylphenoxy)acetyl)-3-hydroxy-5,5-dimethylcyclohex-2-en-1-one (I31):

Yield, 65%; white solid; ^{1}H NMR (300 MHz, DMSO) δ 16.92 (s, 1H), 6.93 (s, 1H), 6.86 (d, J = 8.4 Hz, 1H), 6.60 (d, J = 8.3 Hz, 1H), 5.25 (s, 2H), 2.49 (d, J = 1.6 Hz, 4H), 2.18 (s, 6H), 1.01 (s, 6H). ^{13}C NMR (75 MHz, DMSO) δ 198.55, 196.22, 190.57, 154.64, 130.52, 130.40, 126.83, 126.40, 114.17, 113.68, 76.24, 51.10, 46.62, 33.51, 28.74, 21.23, 16.02. HRMS m/z: calculated 303.15181 ($C_{18}H_{22}O_4$ + H)$^+$, found 303.15161 ($C_{18}H_{22}O_4$ + H)$^+$.

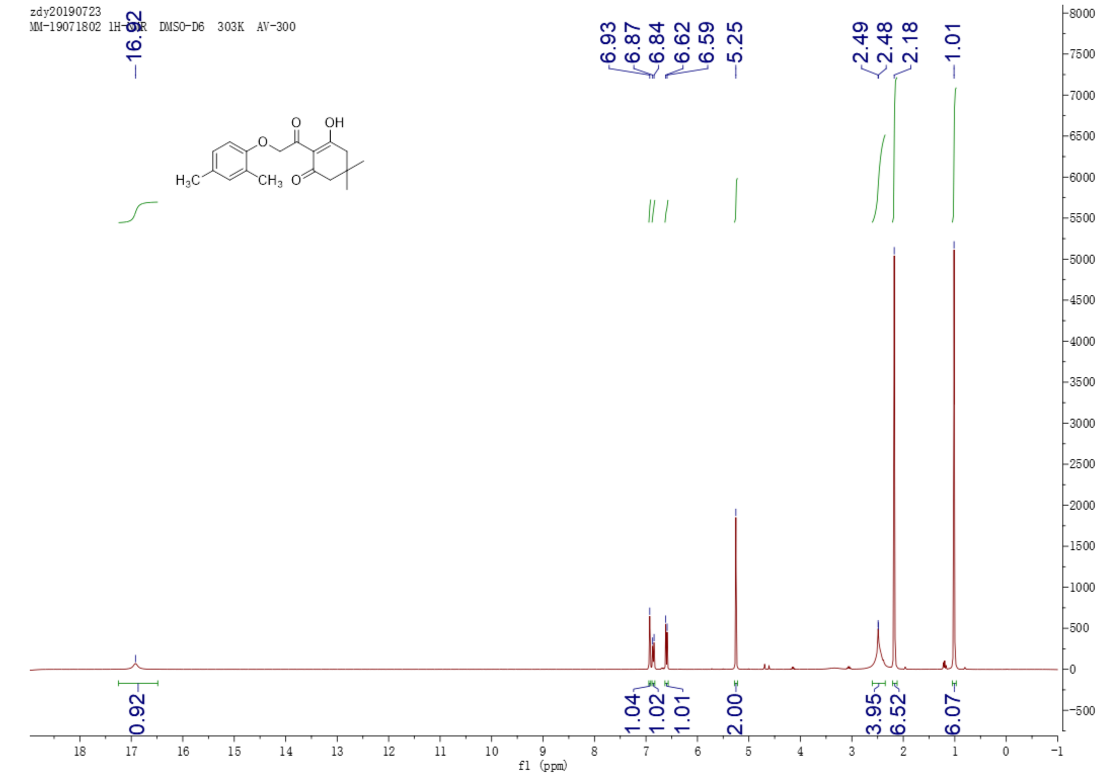

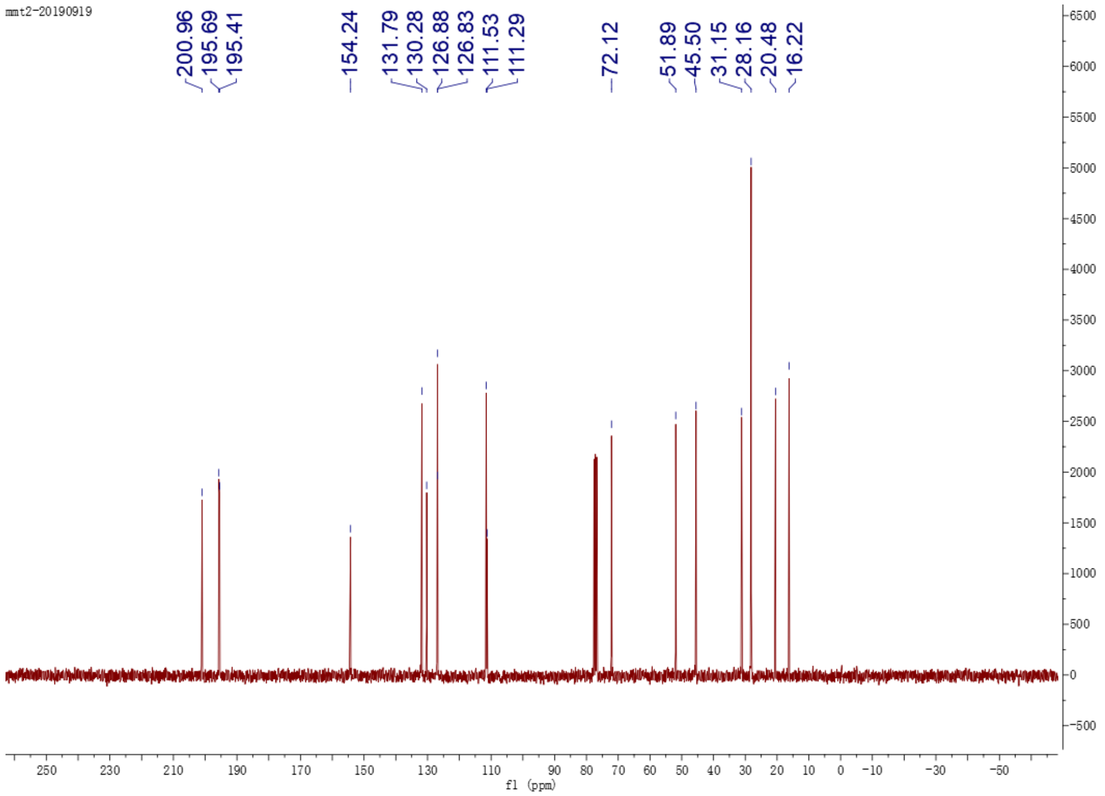

2-(2,4-Dimethylphenoxy)-1-(5-hydroxy-1,3-dimethyl-1*H*-pyrazol-4-yl)ethan-1-one (I32):

Yield, 79%; white solid; ^{1}H NMR (300 MHz, DMSO) δ 7.02 (s, 1H), 6.91 (s, 1H),

6.85 (d, J = 8.1 Hz, 1H), 6.60 (d, J = 7.8 Hz, 1H), 5.11 (s, 2H), 3.37 (s, 3H), 2.32 (s, 3H), 2.18 (s, 6H). ^{13}C NMR (75 MHz, DMSO) δ 187.85, 160.94, 154.64, 149.15, 130.52, 130.40, 126.83, 126.40, 113.68, 99.47, 76.24, 34.91, 21.23, 16.02, 14.7. HRMS m/z: calculated 275.13174 ($C_{15}H_{18}N_2O_3$ + H)$^+$, found 275.13190 ($C_{15}H_{18}N_2O_3$ + H)$^+$.

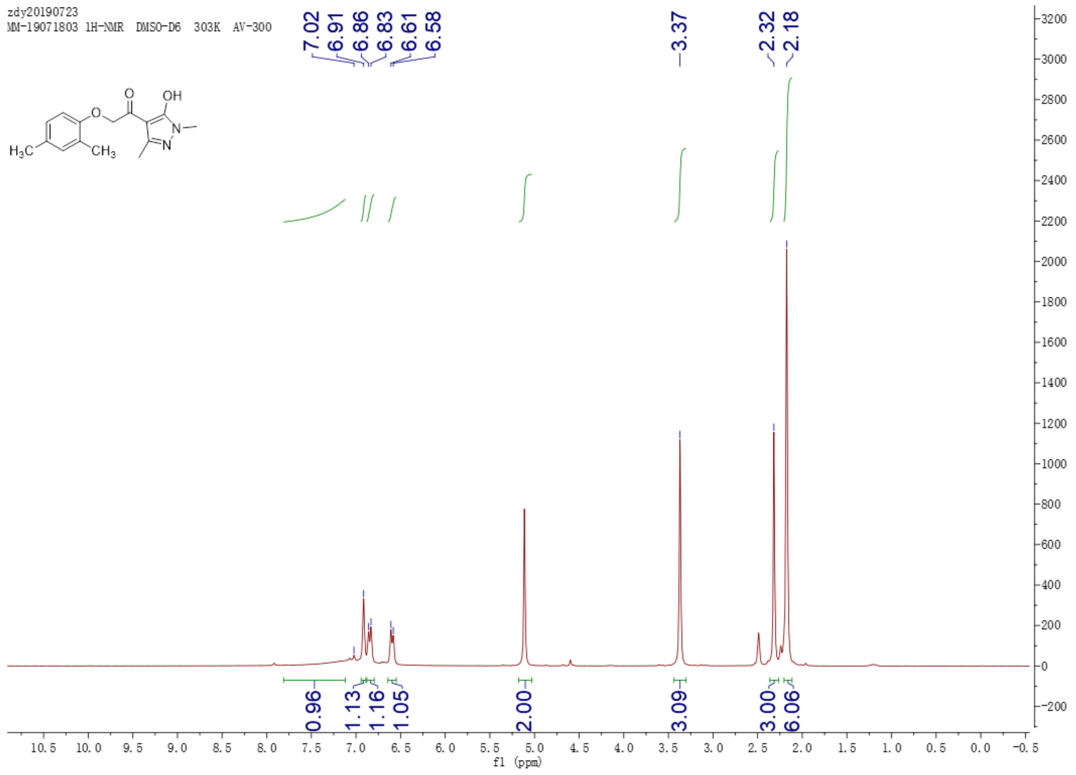

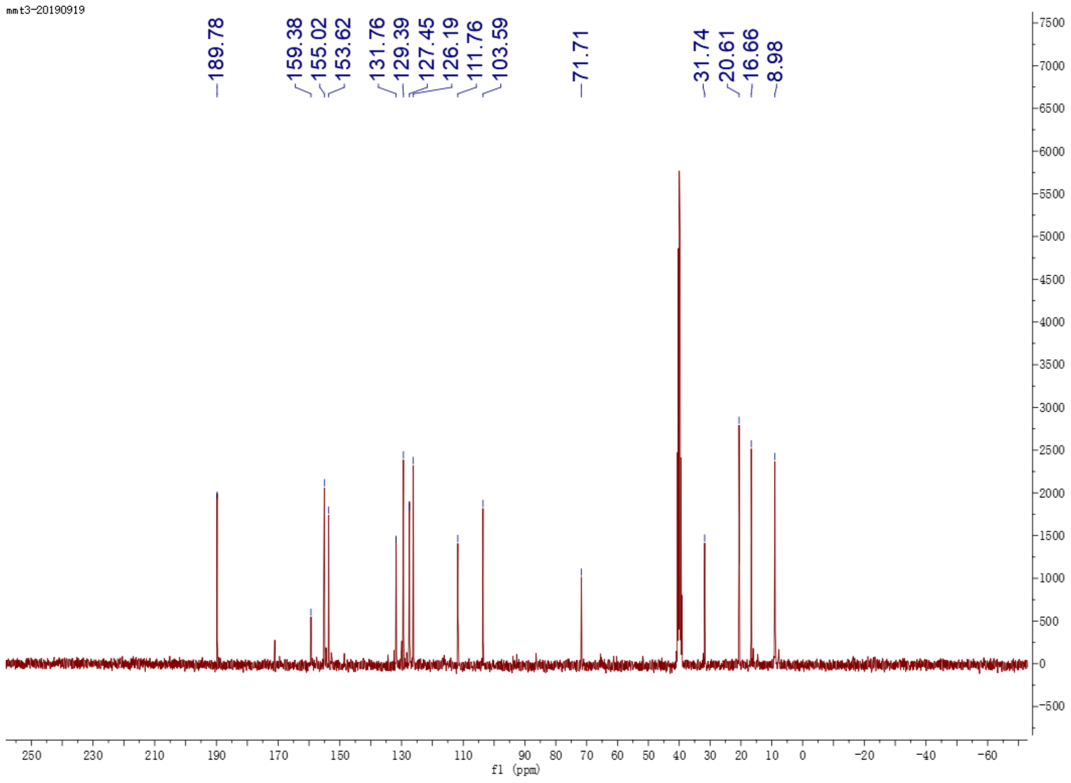

1-(3-Cyclopropyl-5-hydroxy-1-methyl-1*H*-pyrazol-4-yl)-2-(2,4-dimethylphenoxy)ethan-1-one (I33):

Yield, 75%; white solid; ^{1}H NMR (300 MHz, DMSO) δ 6.92 (s, 1H), 6.85 (d, J = 8.3 Hz, 1H), 6.57 (d, J = 8.2 Hz, 1H), 6.30 (s, 1H), 5.16 (s, 2H), 3.35 (s, 3H), 2.56 (m, 1H), 2.17 (d, J = 1.7 Hz, 6H), 1.03 – 0.78 (m, 4H). ^{13}C NMR (75 MHz, DMSO) δ 188.38, 163.23, 154.64, 149.16, 130.52, 130.40, 126.83, 126.40, 113.68, 101.82, 76.24, 34.91, 21.23, 16.02, 11.40, 9.99. HRMS m/z: calculated 301.14739 ($C_{17}H_{20}N_2O_3$ + H)$^+$, found 301.14740 ($C_{17}H_{20}N_2O_3$ + H)$^+$.

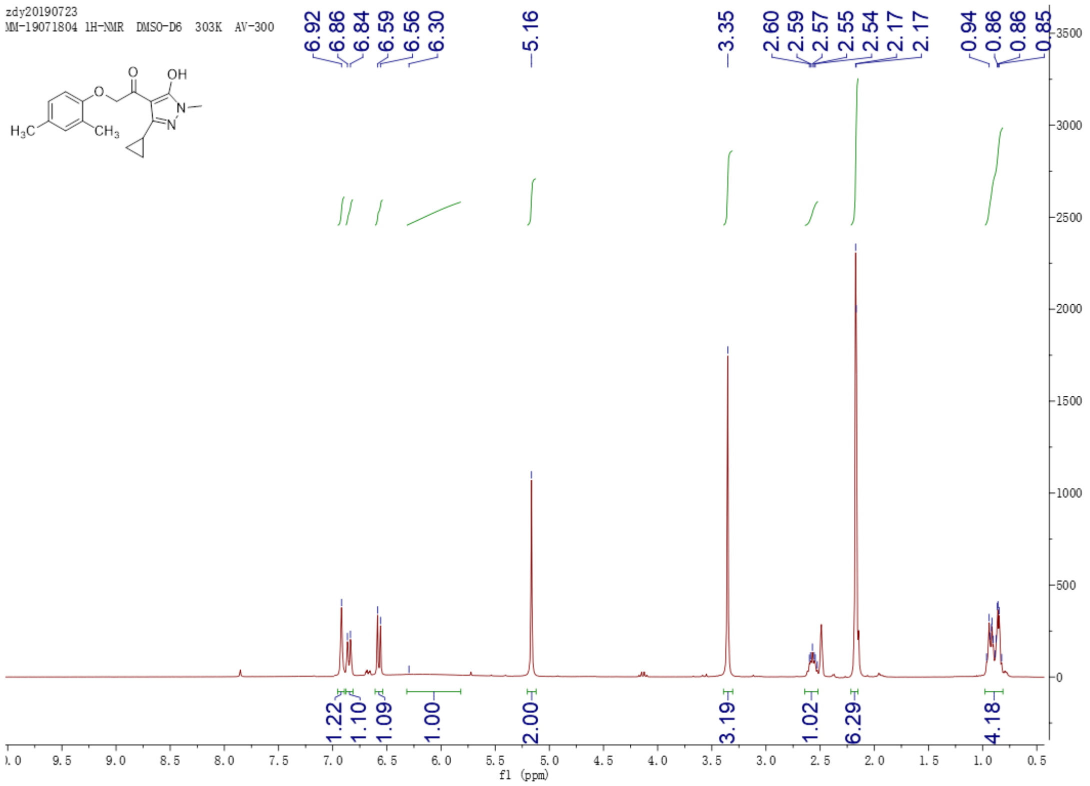

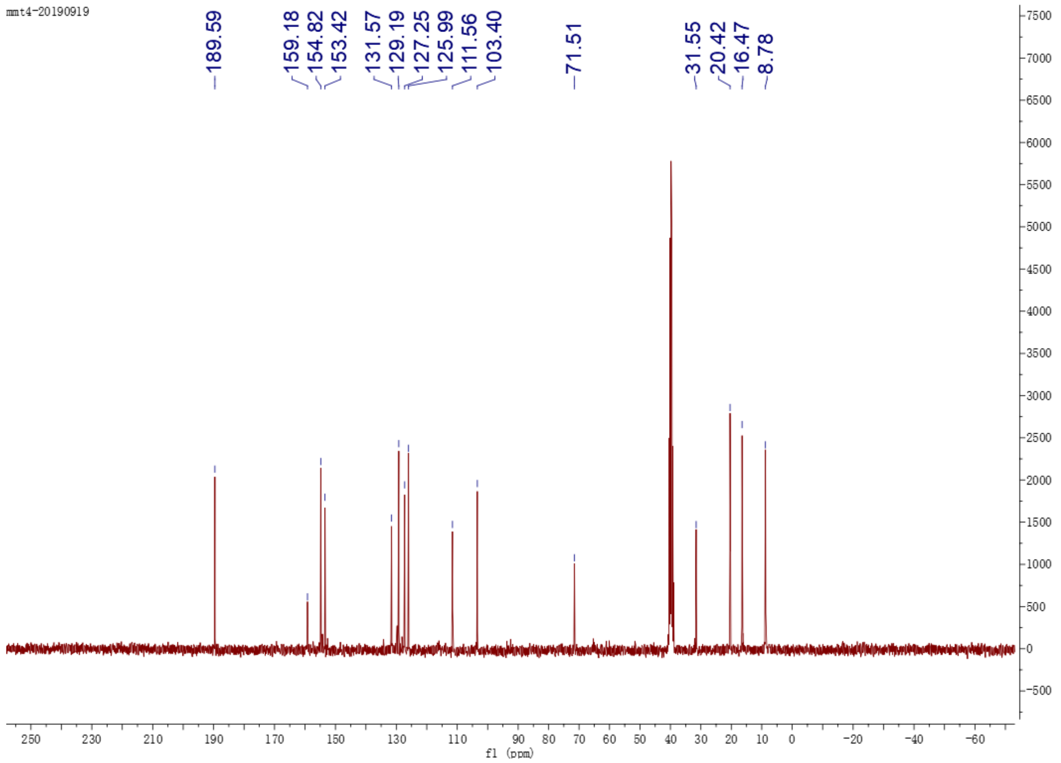

2-(2,4-Dimethylphenoxy)-1-(5-hydroxy-1-methyl-3-(trifluoromethyl)-1*H*-pyrazol-4-yl)ethan-1-one (I34):

Yield, 84%; white solid; ^{1}H NMR (300 MHz, DMSO) δ 9.70 (s, 1H), 6.93 (s, 1H),

6.85 (d, J = 8.9 Hz, 1H), 6.58 (d, J = 8.2 Hz, 1H), 5.14 (s, 2H), 3.67 (s, 3H), 2.17 (s, 6H). ^{13}C NMR (75 MHz, DMSO) δ 188.49, 187.22, 154.64, 135.73, 130.52, 130.40, 126.83, 126.40, 124.29, 113.68, 105.30, 76.24, 34.91, 21.23, 16.02. HRMS m/z: calculated 329.10348 ($C_{15}H_{15}F_3N_2O_3$ + H)$^+$, found 329.10331 ($C_{15}H_{15}F_3N_2O_3$ + H)$^+$.

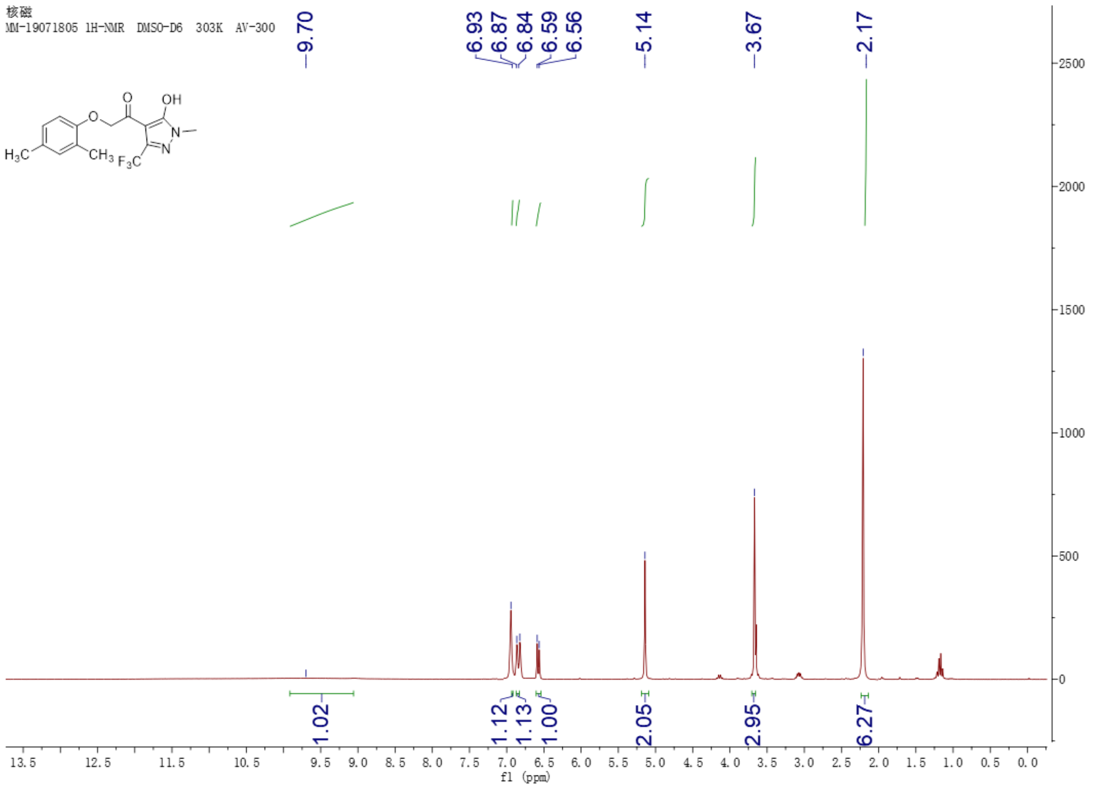

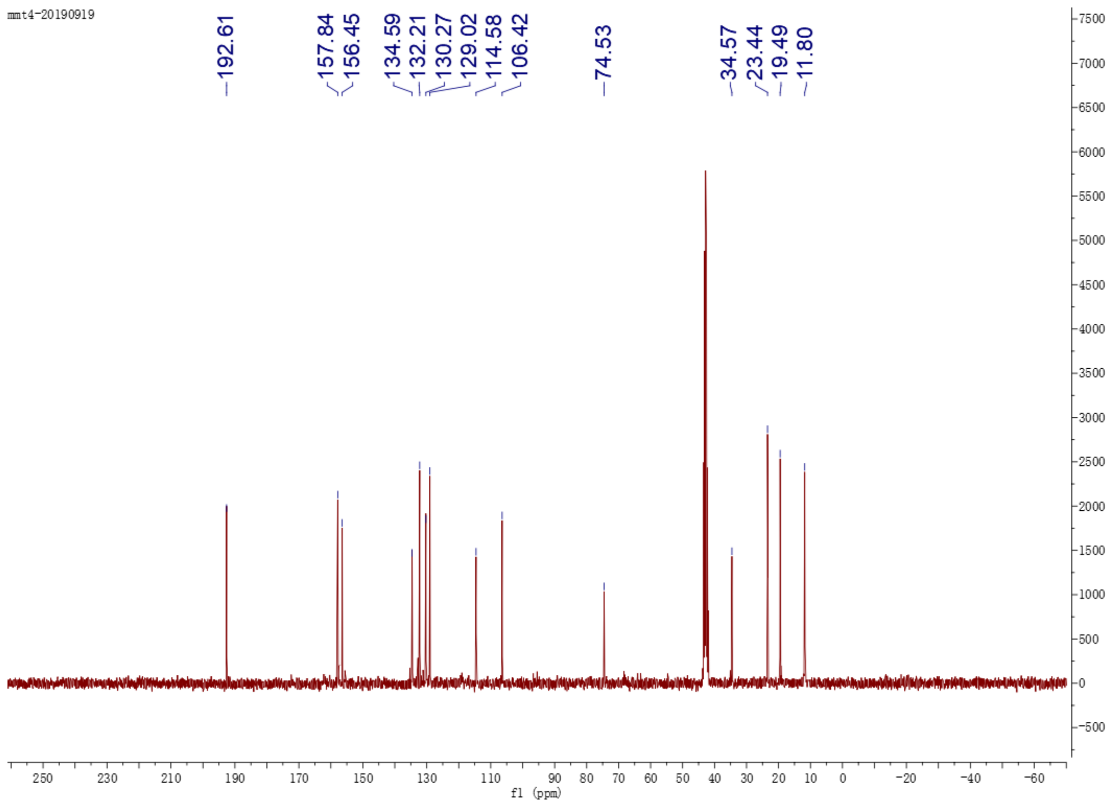

3-Hydroxy-2-(2-(2-methoxy-4-methylphenoxy)acetyl)cyclohex-2-en-1-one (I35): Yield, 77%; white solid; ^{1}H NMR (300 MHz, $CDCl_3$) δ 17.28 (s, 1H), 6.76 (d, J = 1.4 Hz, 1H), 6.72 (d, J = 8.1 Hz, 1H), 6.67 (dd, J = 8.2, 1.2 Hz, 1H), 5.36 (s, 2H), 3.91 (s, 3H), 2.73 (t, J = 6.4 Hz, 2H), 2.61 – 2.49 (m, 2H), 2.32 (s, 3H), 2.05 (p, J = 6.5 Hz, 2H). ^{13}C NMR (75 MHz, $CDCl_3$) δ 203.00, 197.55, 190.57, 150.28, 146.63, 131.75, 122.03, 116.12, 113.47, 108.91, 76.24, 56.83, 36.74, 29.40, 21.23, 19.99. HRMS m/z: calculated 291.11542 ($C_{16}H_{18}O_5$ + H)$^+$, found 291.11536 ($C_{16}H_{18}O_5$ + H)$^+$.

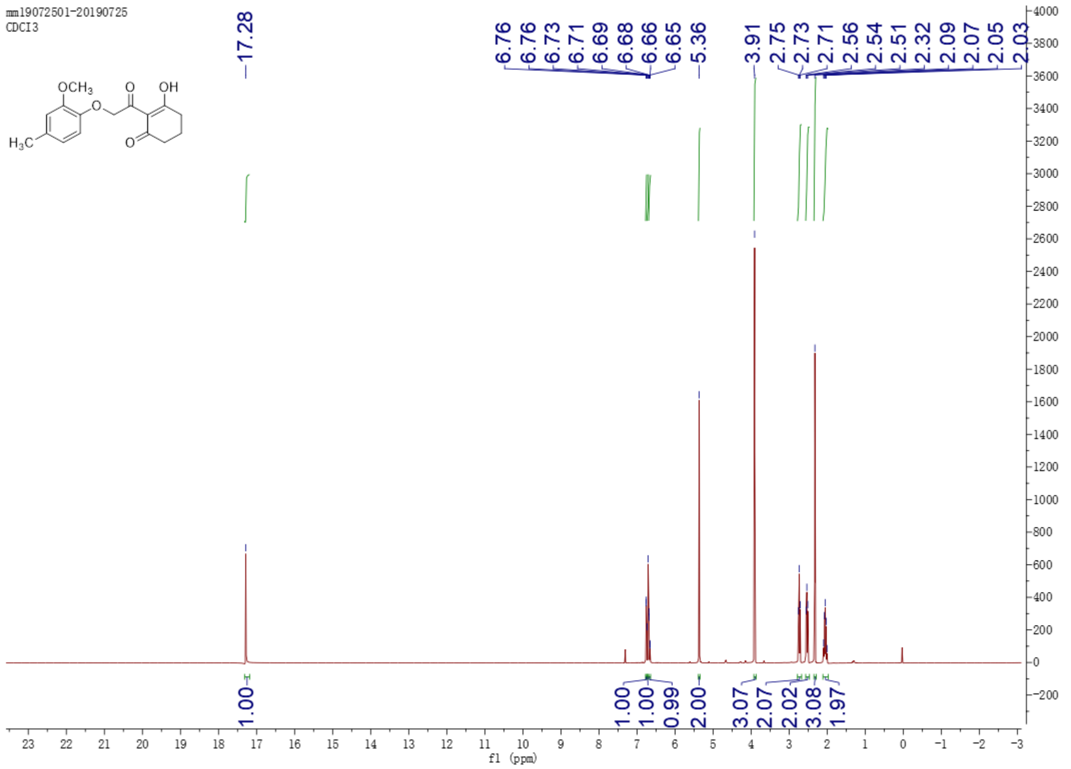

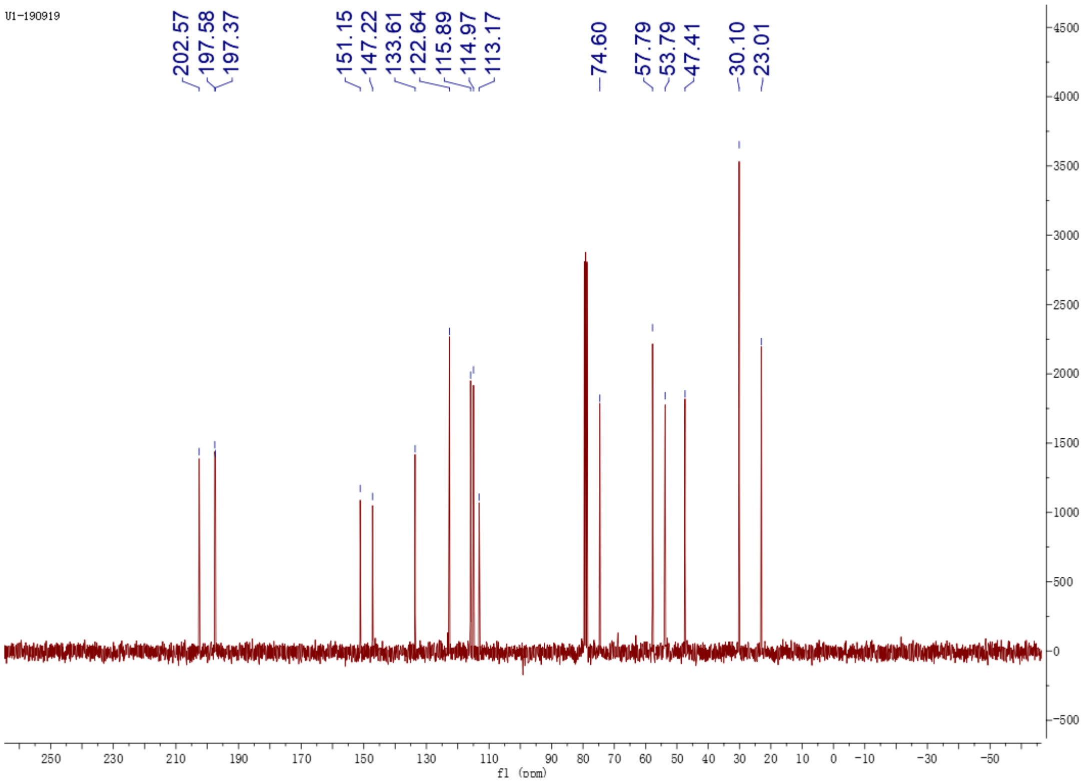

3-Hydroxy-2-(2-(2-methoxy-4-methylphenoxy)acetyl)-5,5-dimethylcyclohex-2-en-1-one (I36):

Yield, 83%; white solid; ^{1}H NMR (300 MHz, $CDCl_3$) δ 17.25 (s, 1H), 6.72 (d, J = 8.0 Hz, 2H), 6.66 (dd, J = 8.1, 1.1 Hz, 1H), 5.35 (s, 2H), 3.88 (s, 3H), 2.57 (s, 2H), 2.38

(s, 2H), 2.30 (s, 3H), 1.10 (s, 6H). ^{13}C NMR 75 MHz, $CDCl_3$) δ 198.55, 196.22, 190.57, 150.28, 146.63, 131.75, 122.03, 116.12, 114.17, 113.47, 76.24, 56.83, 51.10, 46.62, 33.51, 28.74, 21.23. HRMS m/z: calculated 319.14672 ($C_{18}H_{22}O_5$ + H)$^+$, found 319.14591 ($C_{18}H_{22}O_5$ + H)$^+$.

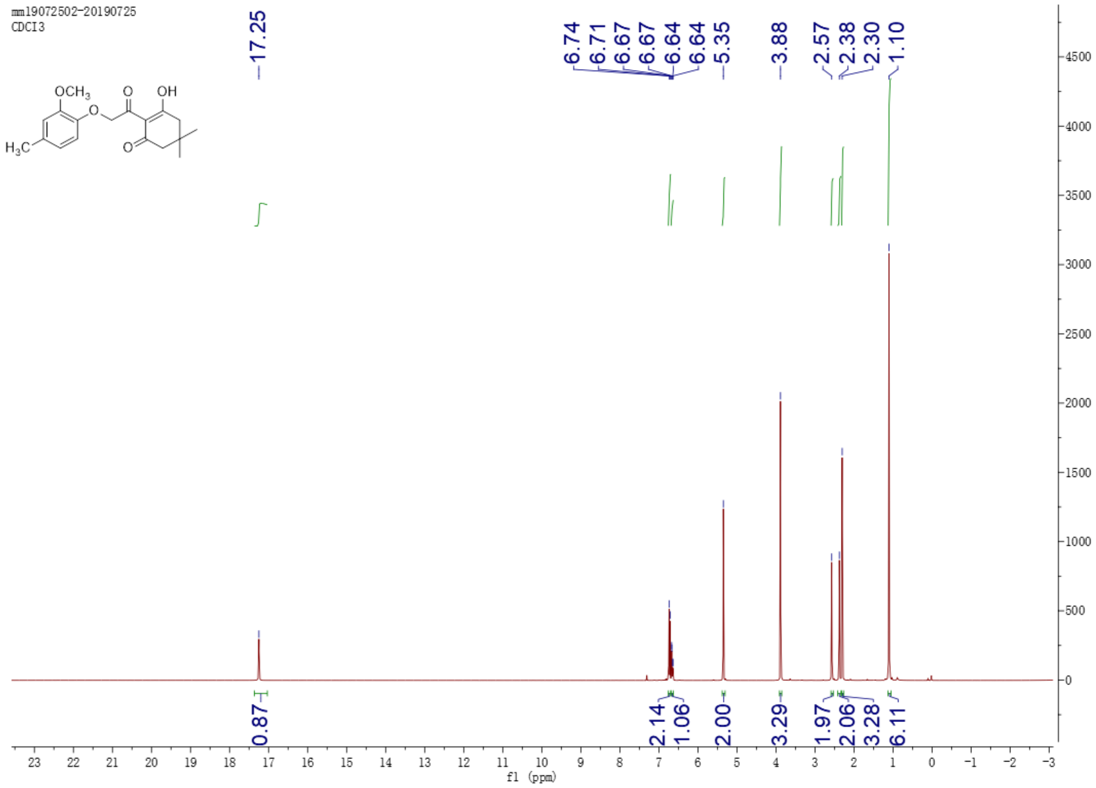

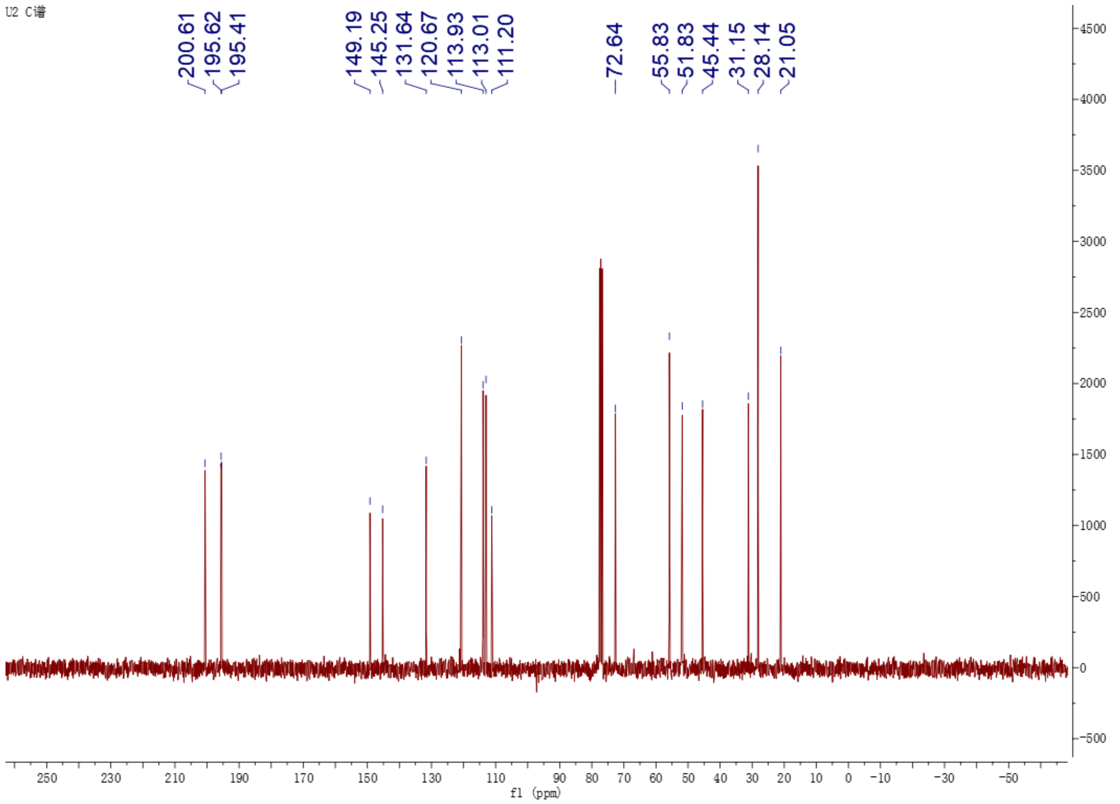

1-(5-Hydroxy-1,3-dimethyl-1*H*-pyrazol-4-yl)-2-(2-methoxy-4-methylphenoxy)ethan-1-one (I37):

Yield, 79%; white solid; ^{1}H NMR (300 MHz, DMSO) δ 6.81 (s, 1H), 6.63 (s, 2H), 5.12 (s, 2H), 3.78 (s, 3H), 3.38 (s, 3H), 2.34 (s, 3H), 2.25 (s, 3H). ^{13}C NMR (75 MHz, DMSO) δ 187.85, 160.94, 150.28, 149.15, 146.63, 131.75, 122.03, 116.12, 113.47, 99.47, 76.24, 56.83, 34.91, 21.23, 14.74. HRMS m/z: calculated 291.12666 ($C_{15}H_{18}N_2O_4$ + H)$^+$, found 291.12679 ($C_{15}H_{18}N_2O_4$ + H)$^+$.

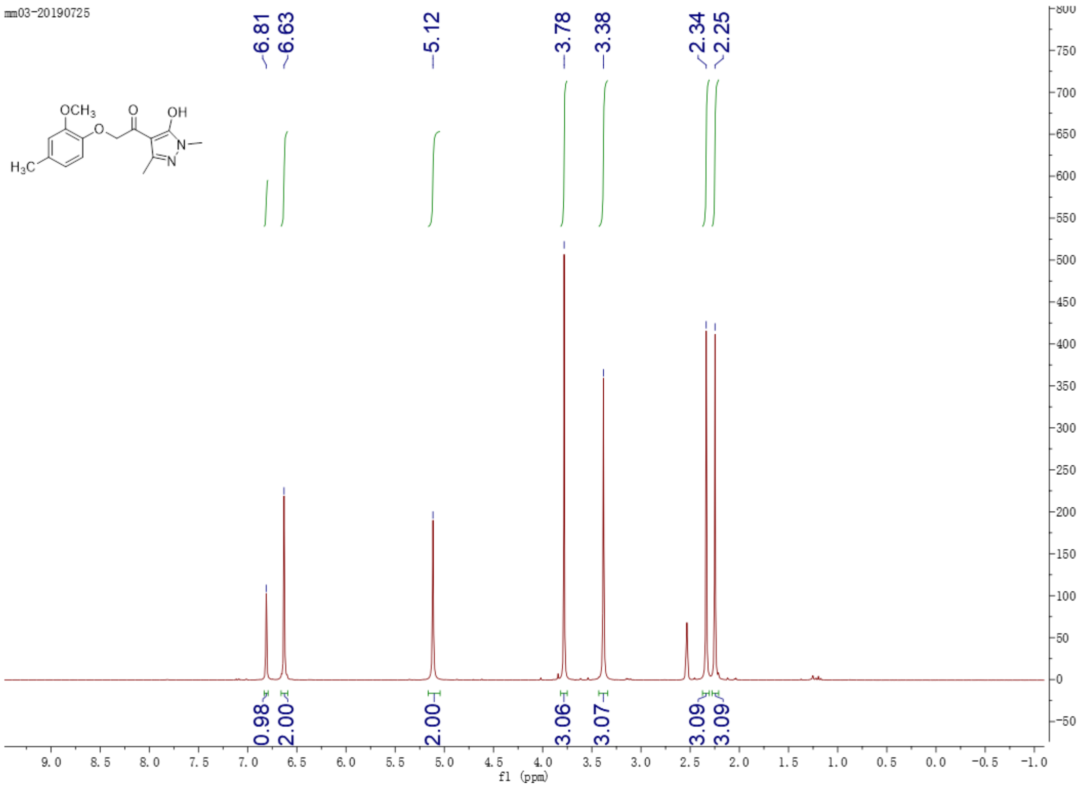

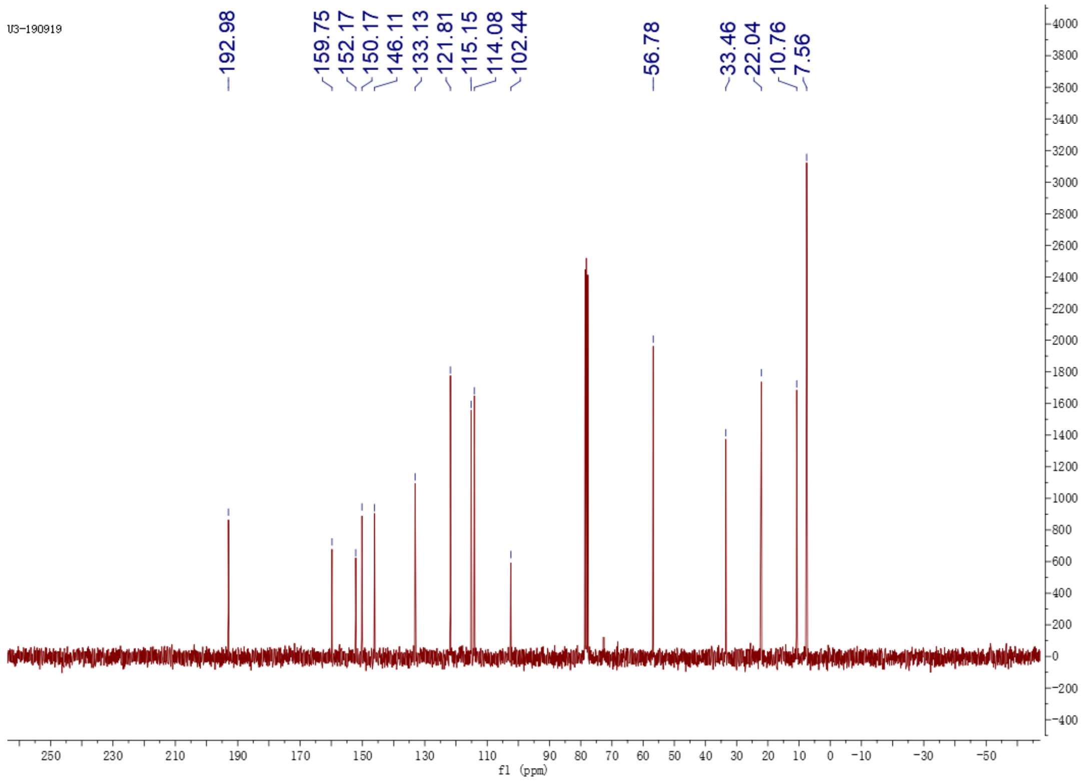

1-(3-Cyclopropyl-5-hydroxy-1-methyl-1*H*-pyrazol-4-yl)-2-(2-methoxy-4-methylphenoxy)ethan-1-one (I38):

Yield, 73%; white solid; ^{1}H NMR (300 MHz, DMSO) δ 6.82 (s, 1H), 6.74 – 6.58 (m,

2H), 5.16 (s, 2H), 3.79 (s, 3H), 3.39 (s, 3H), 2.62 (s, 1H), 2.25 (s, 3H), 1.21 – 0.75 (m, 4H). ^{13}C NMR (75 MHz, DMSO) δ 188.38, 163.23, 150.28, 149.16, 146.63, 131.75, 122.03, 116.12, 113.47, 101.82, 76.24, 56.83, 34.91, 21.23, 11.40, 9.99. HRMS m/z: calculated 317.14231 $(C_{17}H_{20}N_2O_4 + H)^+$, found 317.14267 $(C_{17}H_{20}N_2O_4 + H)^+$.

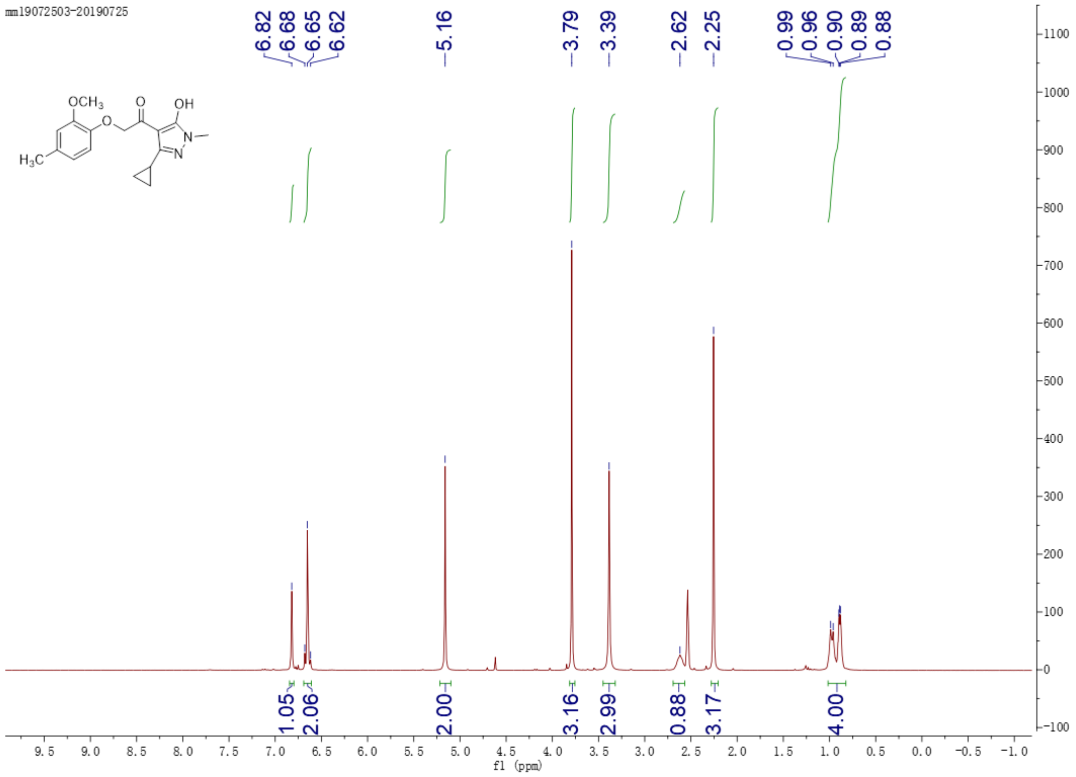

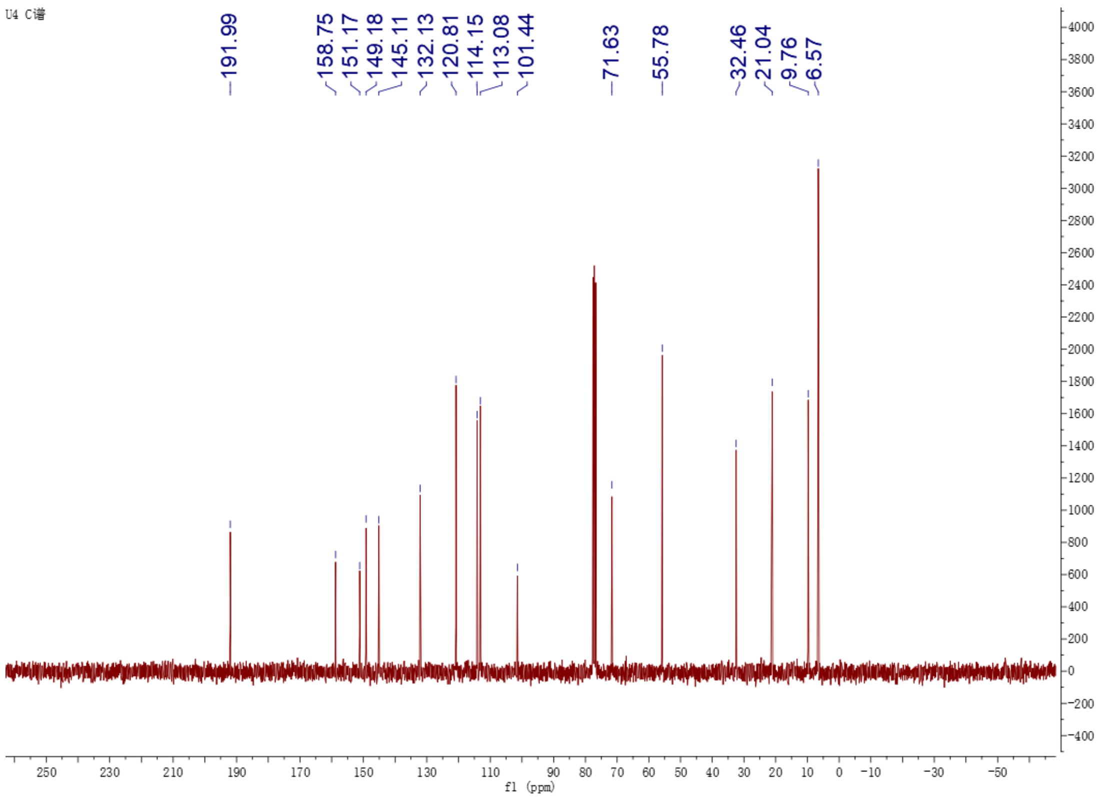

3-Hydroxy-2-(2-phenoxyacetyl)cyclohex-2-en-1-one (I39)[1]:

Yield, 81%; white solid; ^{1}H NMR (300 MHz, $CDCl_3$) δ 17.14 (s, 1H), 7.29 (t, J = 7.9 Hz, 2H), 6.99 (d, J = 7.3 Hz, 1H), 6.94 (d, J = 8.1 Hz, 2H), 5.32 (s, 2H), 2.71 (t, J = 6.3 Hz, 2H), 2.57 – 2.43 (m, 2H), 2.02 (p, J = 6.4 Hz, 2H).

^{13}C NMR (75 MHz, $CDCl_3$) δ 200.87, 196.72, 195.51, 129.45, 121.34, 114.70, 71.72, 38.01, 31.89, 19.18. MS (ESI) HRMS m/z: calculated 247.08921 $(C_{14}H_{14}O_4 + H)^+$, found 247.08921 $(C_{14}H_{14}O_4 + H)^+$.

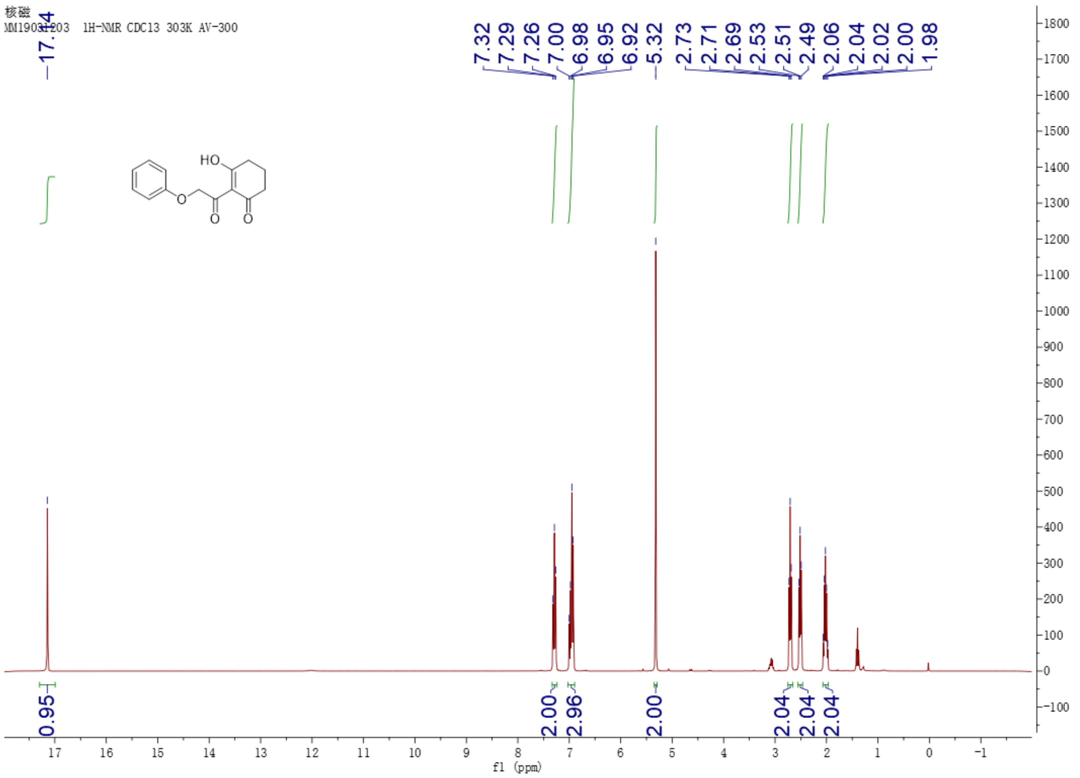

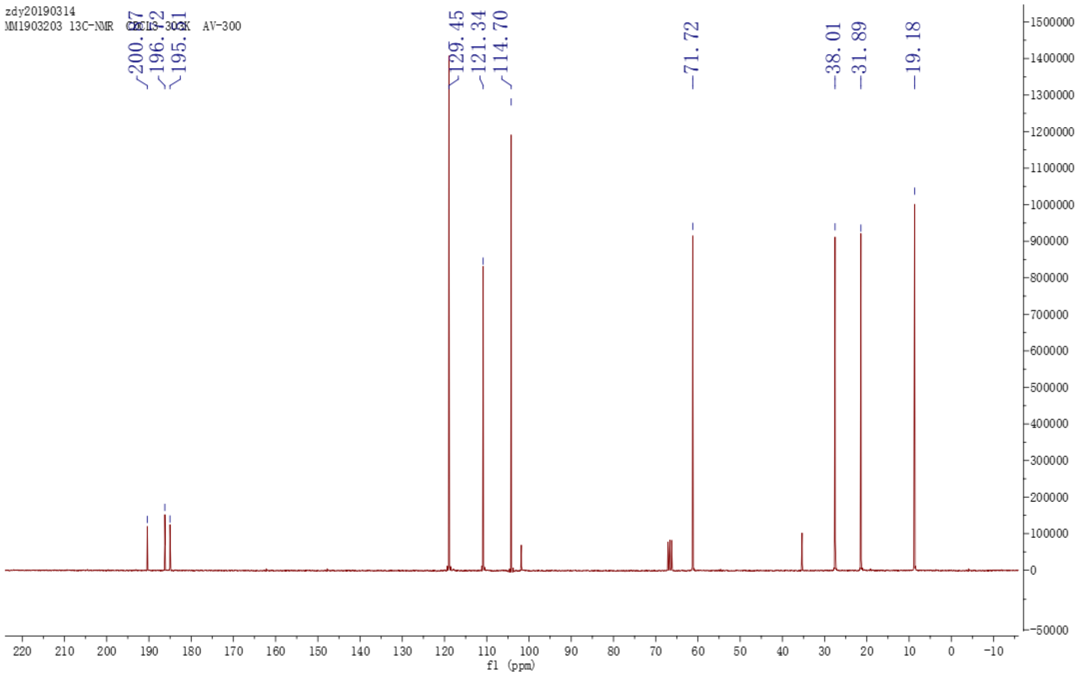

1-(5-Hydroxy-1,3-dimethyl-1H-pyrazol-4-yl)-2-phenoxyethan-1-one (I40)[22]: Yield, 70%; white solid; ^{1}H NMR (300 MHz, $CDCl_3$) δ 7.33 (t, J = 7.9 Hz, 1H), 7.04 (t, J = 7.4 Hz, 1H), 6.97 (d, J = 8.2 Hz, 1H), 4.88 (s, 1H), 3.53 (s, 1H), 2.23 (s, 1H). ^{13}C NMR (75 MHz, DMSO) δ 188.23, 157.78, 152.47, 128.76, 119.90, 113.80, 102.34, 70.03, 30.64, 7.74, 7.71. HRMS m/z: calculated 247.10044 ($C_{13}H_{14}N_2O_3$ +

H)$^+$, found 247.10142 ($C_{13}H_{14}N_2O_3$ + H)$^+$.

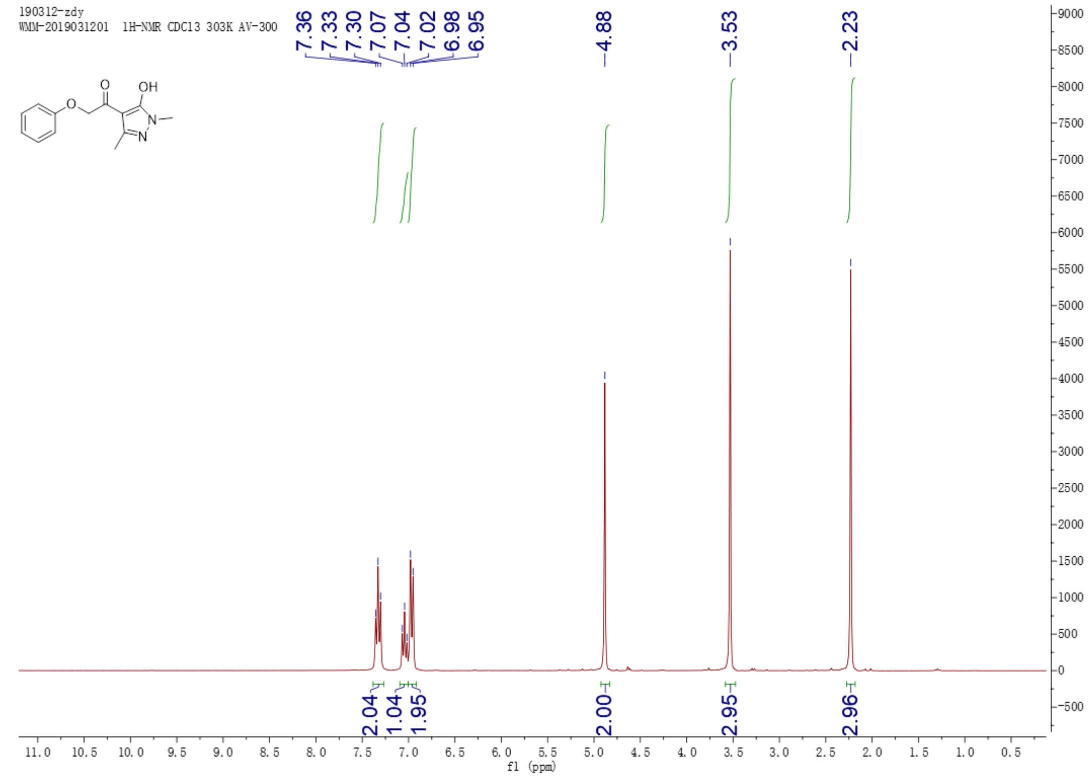

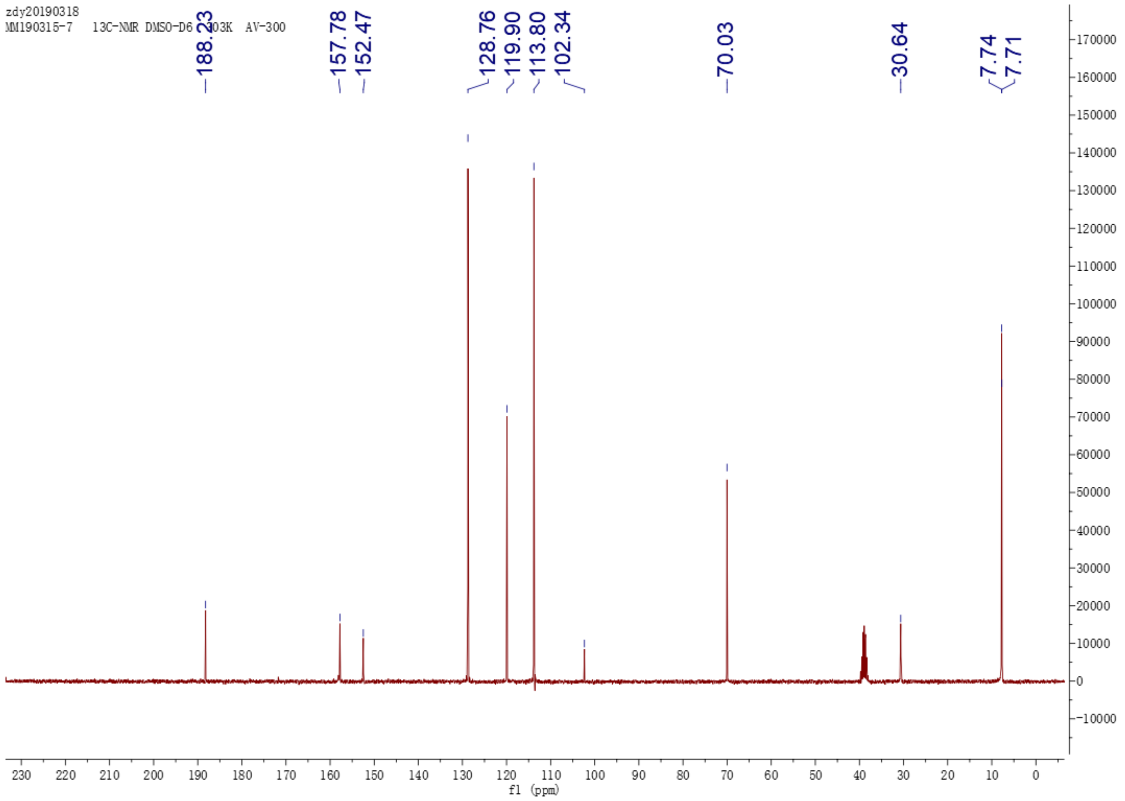

3-Hydroxy-2-(2-((3,5,6-trichloropyridin-2-yl)oxy)acetyl)cyclohex-2-en-1-one (II1):

Yield, 86%; white solid; ^{1}H NMR (300 MHz, $CDCl_3$) δ 16.72 (s, 1H), 7.77 (s, 1H),

5.65 (s, 2H), 2.73 (t, $J = 6.1$ Hz, 2H), 2.66 – 2.49 (m, 2H), 2.18 – 1.98 (m, 2H). ^{13}C NMR (75 MHz, $CDCl_3$) δ 199.09, 195.99, 195.40, 155.86, 140.14, 122.10, 116.75, 112.19, 70.26, 37.76, 31.56, 19.09. HRMS m/z: calculated 349.96754 ($C_{13}H_{10}Cl_3NO_4$ + H)$^+$, found 349.96647 ($C_{13}H_{10}Cl_3NO_4$ + H)$^+$.

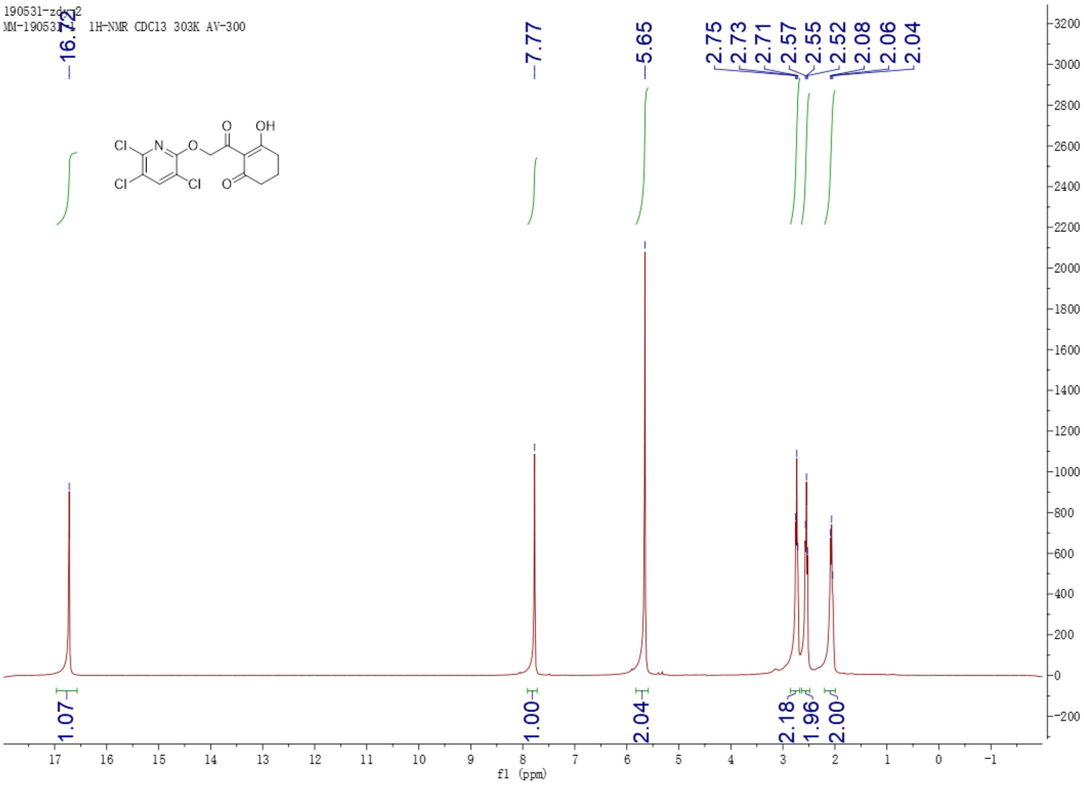

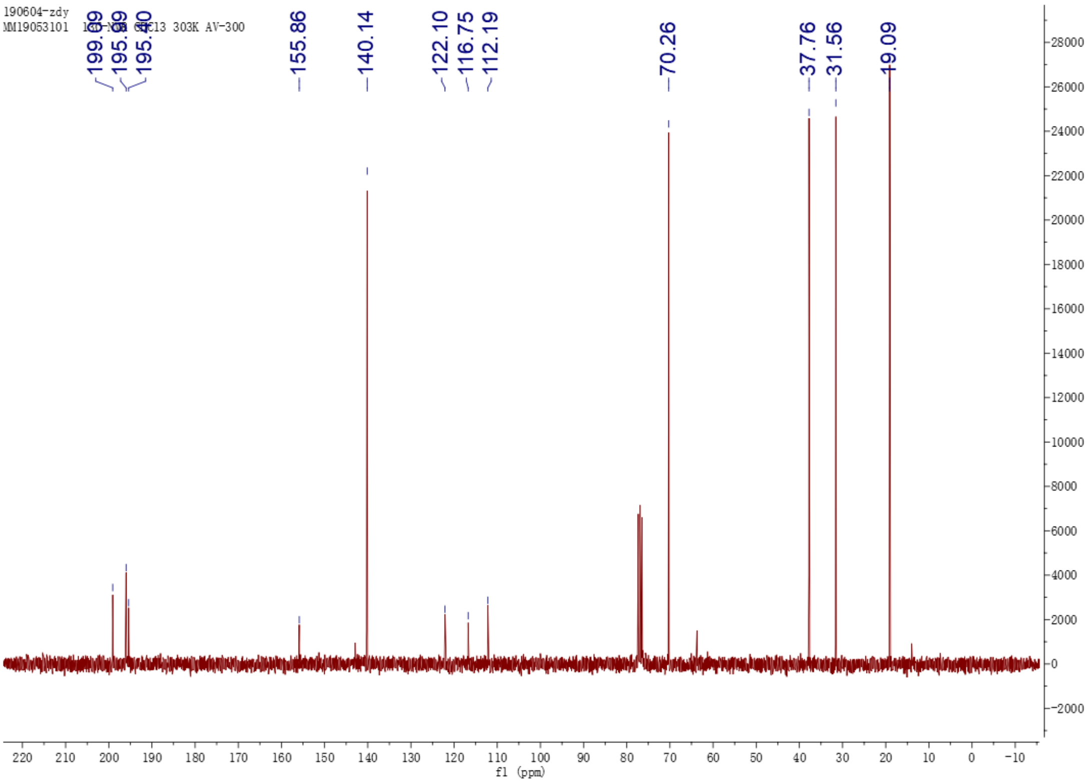

3-Hydroxy-5,5-dimethyl-2-(2-((3,5,6-trichloropyridin-2-yl)oxy)acetyl)cyclohex-2-en-1-one (II2):

Yield, 76%; white solid; ^{1}H NMR (300 MHz, $CDCl_3$) δ 16.72 (s, 1H), 7.77 (s, 1H), 5.65 (s, 2H), 2.73 (t, J = 6.1 Hz, 2H), 2.66 – 2.49 (m, 2H), 2.18 – 1.98 (m, 2H). ^{13}C NMR (75 MHz, $CDCl_3$) δ 199.09, 195.99, 195.40, 155.86, 140.14, 122.10, 116.75, 112.19, 70.26, 37.76, 31.56, 19.09. HRMS m/z: calculated 377.99884 ($C_{15}H_{14}Cl_3NO_4$ + H)$^+$, found 377.99861 ($C_{15}H_{14}Cl_3NO_4$ + H)$^+$.

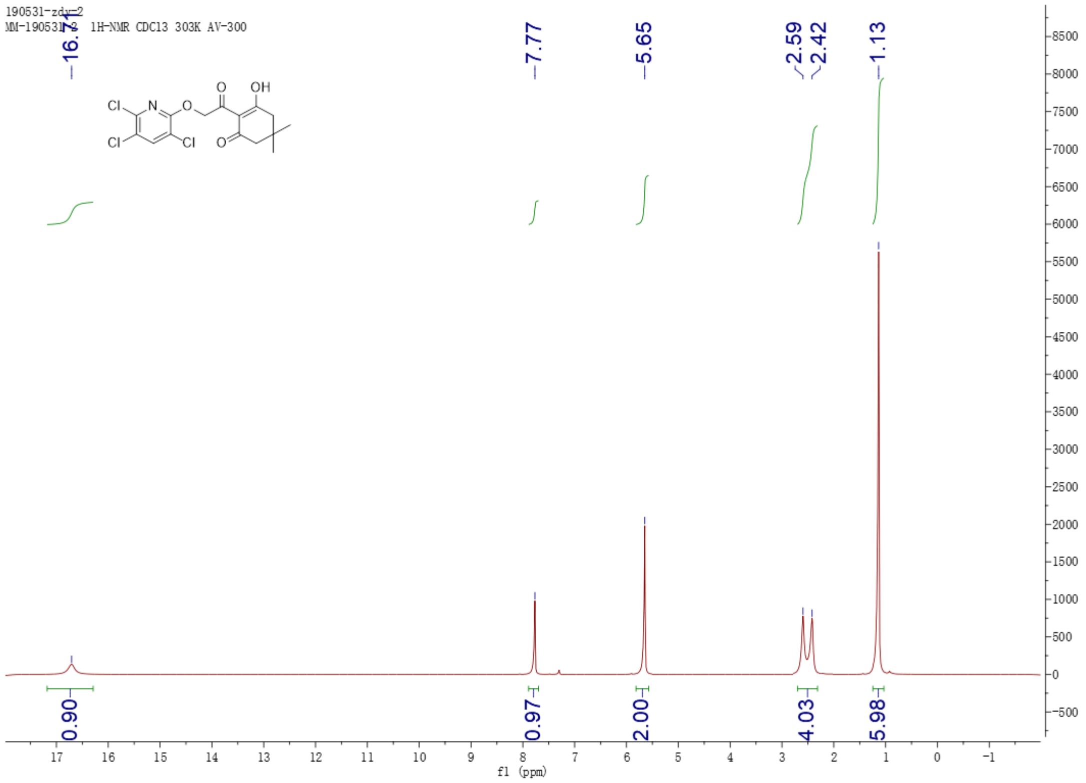

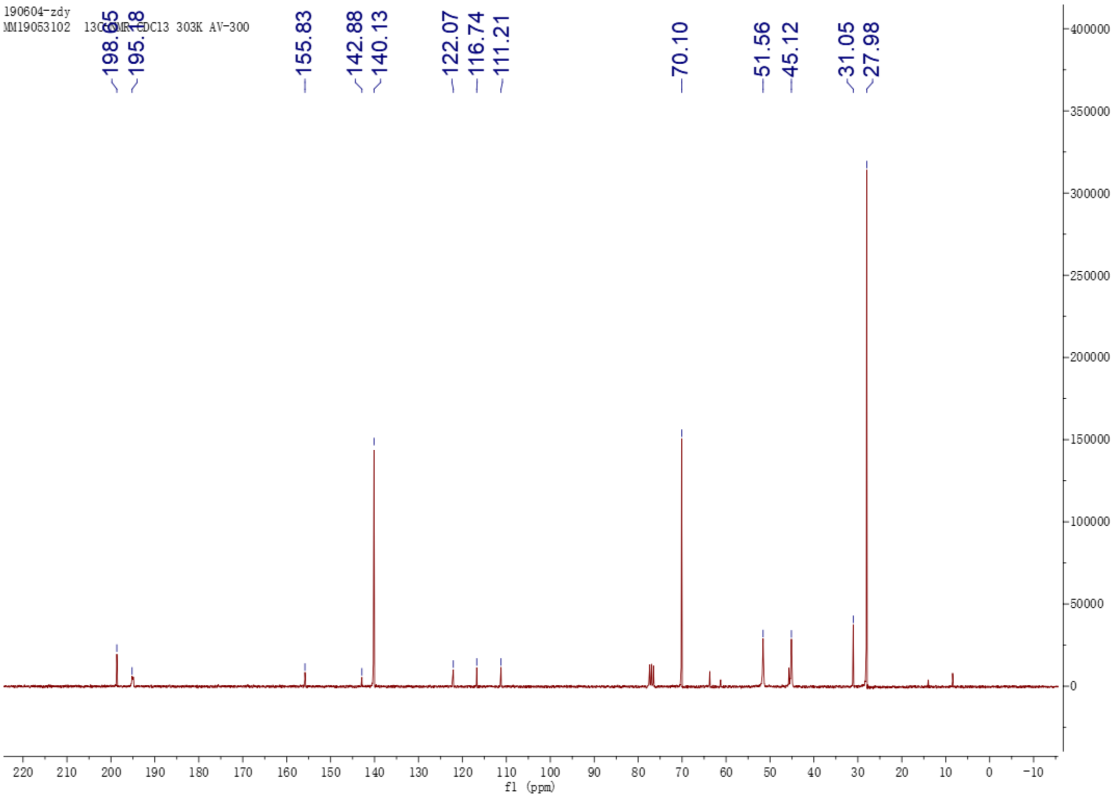

1-(5-Hydroxy-1,3-dimethyl-1*H*-pyrazol-4-yl)-2-((3,5,6-trichloropyridin-2-yl)oxy)ethan-1-one (II3):

Yield, 78%; white solid; ^{1}H NMR (300 MHz, DMSO) δ 8.31 (s, 1H), 5.46 (s, 2H),

3.37 (s, 3H), 2.29 (s, 3H). ^{13}C NMR (75 MHz, $CDCl_3$) δ 186.80, 159.93, 156.58, 147.69, 142.24, 141.14, 121.13, 116.82, 102.16, 70.67, 30.91, 13.62. HRMS m/z: calculated 349.97877 ($C_{12}H_{10}Cl_3N_3O_3$ + H)$^+$, found 349.98608 ($C_{12}H_{10}Cl_3N_3O_3$ + H)$^+$

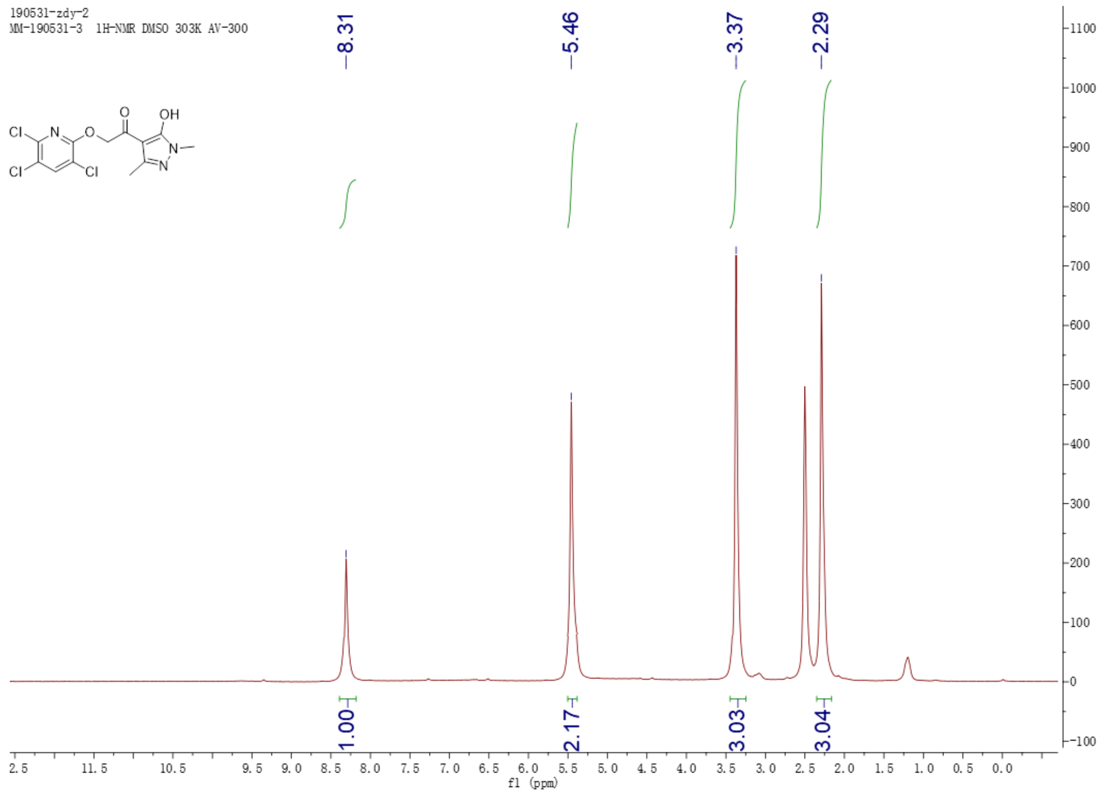

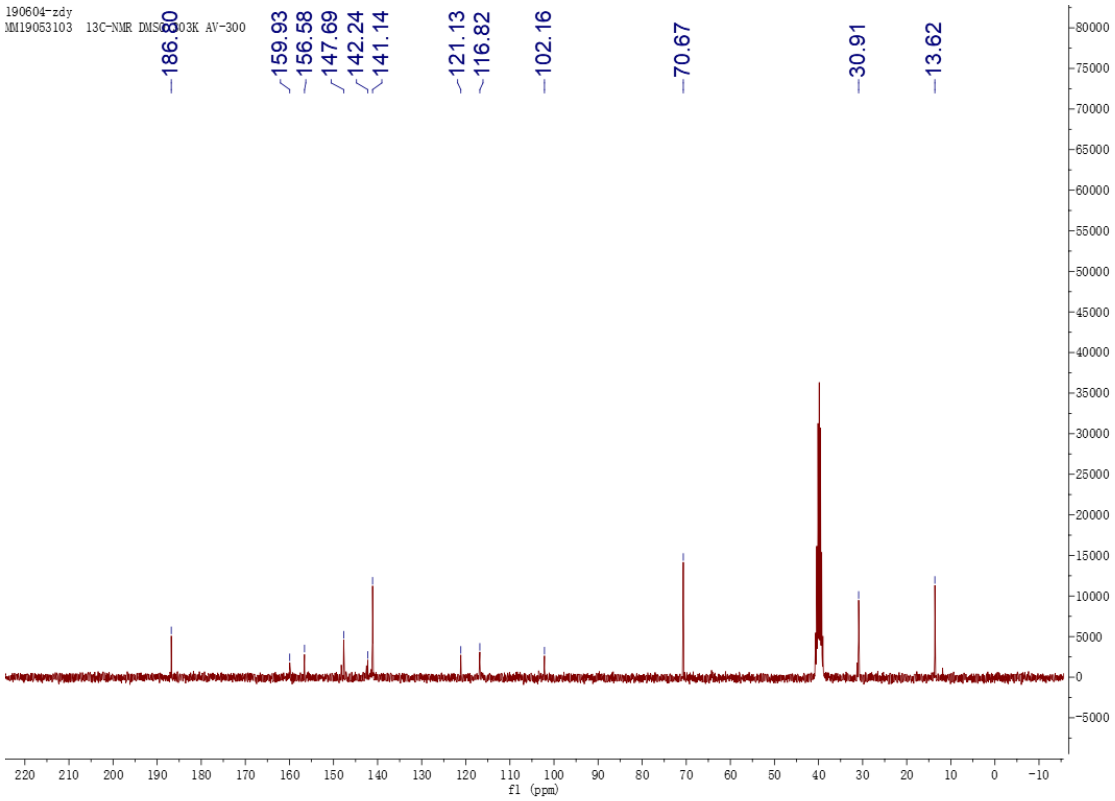

1-(3-Cyclopropyl-5-hydroxy-1-methyl-1*H*-pyrazol-4-yl)-2-((3,5,6-trichloropyridin-2-yl)oxy)ethan-1-one (II4):

Yield, 83%; white solid; ^{1}H NMR (300 MHz, DMSO) *δ* 8.32 (s, 1H), 5.50 (s, 2H), 3.36 (s, 3H), 1.19 (t, *J* = 7.0 Hz, 1H), 0.89 (dd, *J* = 22.5, 5.5 Hz, 4H). ^{13}C NMR (75 MHz, $CDCl_3$) *δ* 186.34, 156.24, 152.96, 141.91, 141.17, 140.73, 120.78, 116.47, 102.37, 70.29, 31.11, 13.91, 8.28. HRMS m/z: calculated 346.00167 ($C_{14}H_{12}Cl_3N_3O_3$ + H)$^+$, found 346.00163 ($C_{14}H_{12}Cl_3N_3O_3$ + H)$^+$

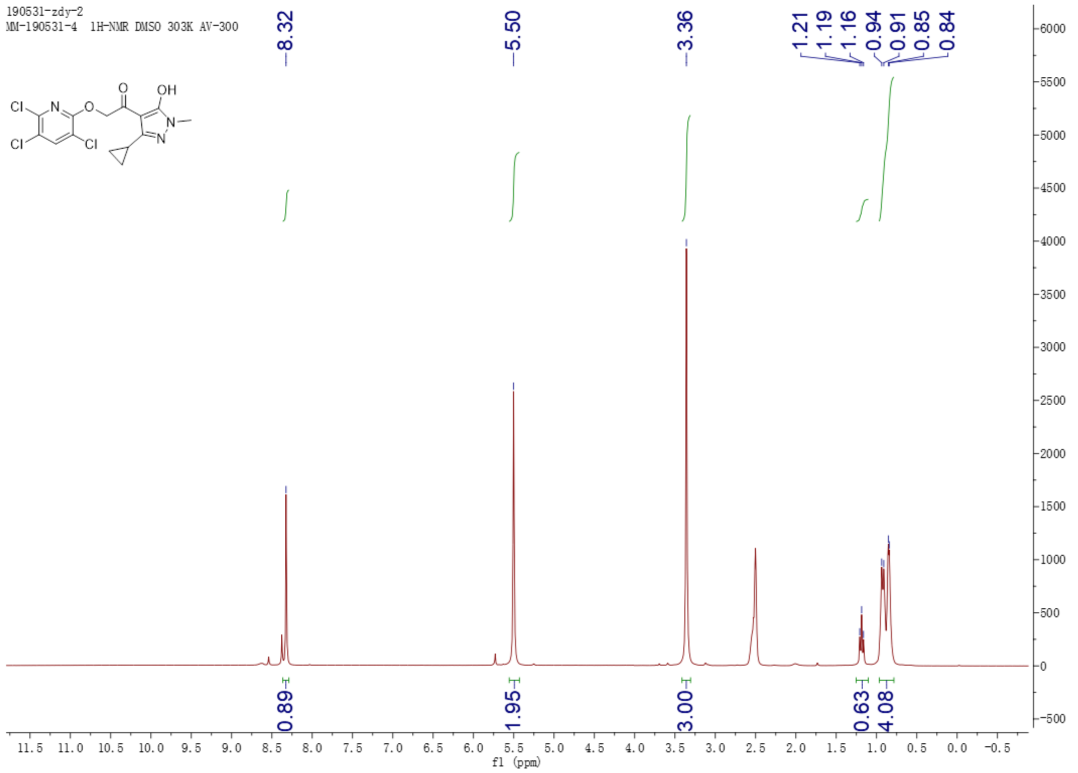

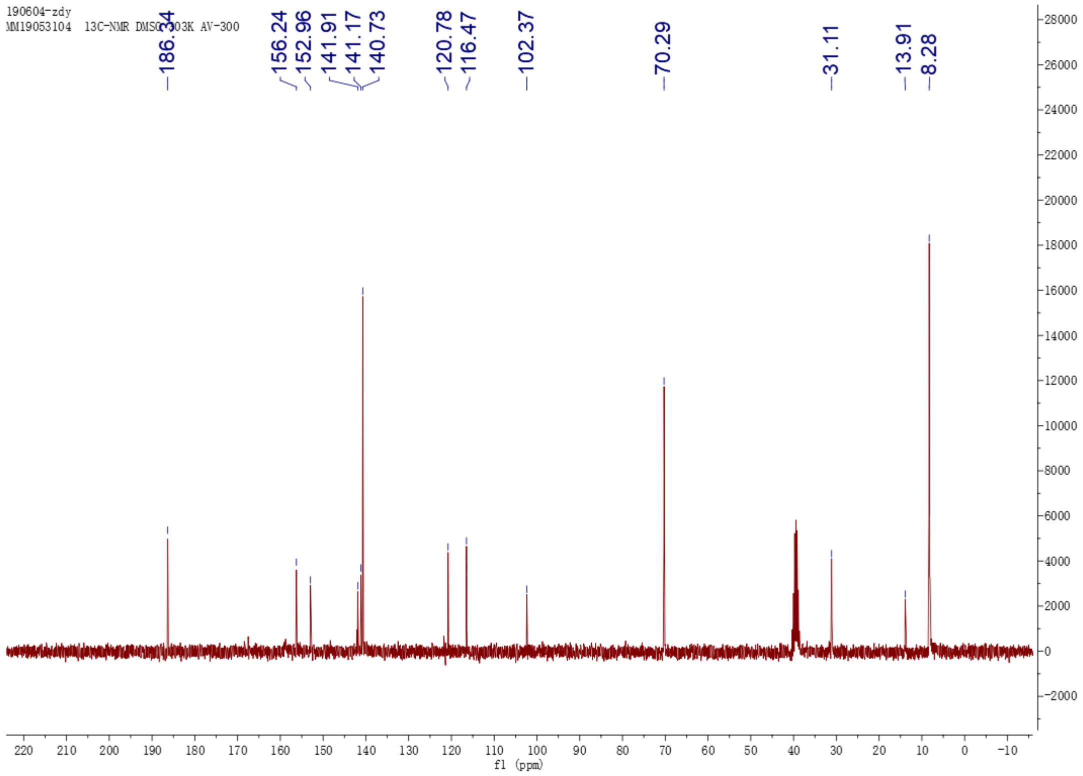

1-(5-Hydroxy-1-methyl-3-(trifluoromethyl)-1*H*-pyrazol-4-yl)-2-((3,5,6-trichloropyridin-2-yl)oxy)ethan-1-one (II5):

Yield, 75%; white solid; ^{1}H NMR (300 MHz, DMSO) δ 11.74 (s, 1H), 8.35 (s, 1H), 5.51 (s, 4H), 3.70 (s, 6H). ^{13}C NMR (75 MHz, $CDCl_3$) δ 183.81, 156.19, 155.89, 141.92, 140.87, 122.36, 121.09, 118.79, 116.45, 101.02, 69.97, 34.45. HRMS m/z: calculated 403.95771 ($C_{12}H_7Cl_3F_3N_3O_3$ + H)$^+$, found 403.95765 ($C_{12}H_7Cl_3F_3N_3O_3$ + H)$^+$

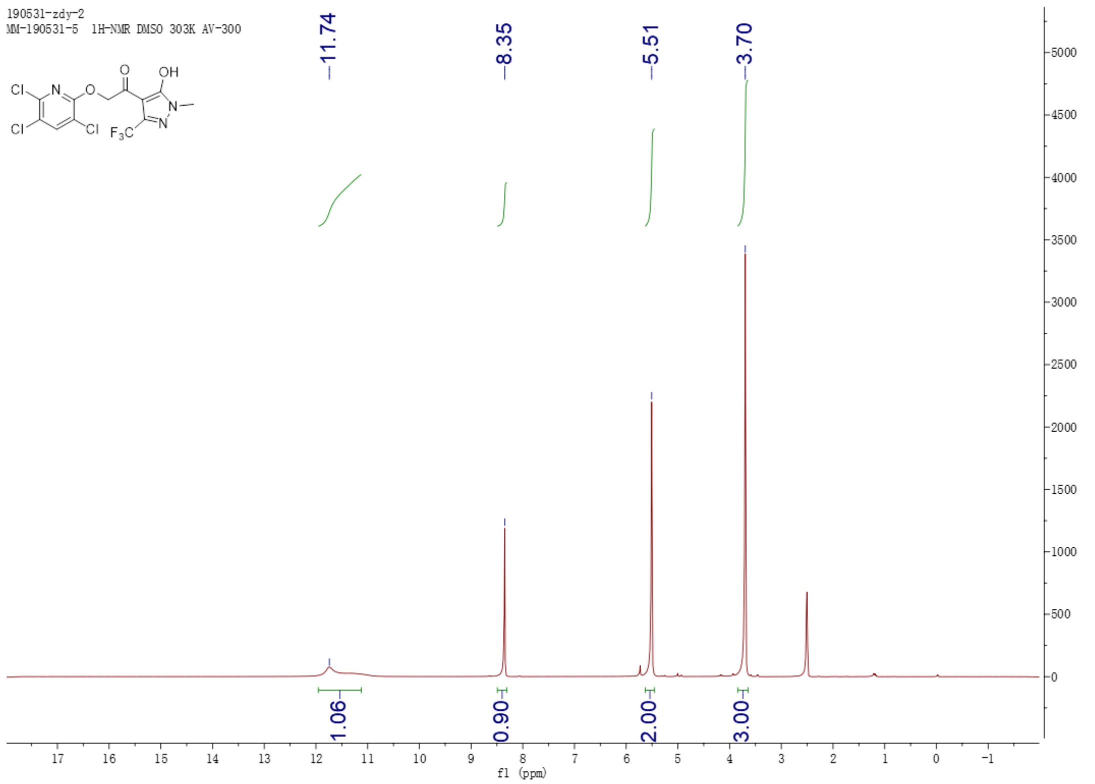

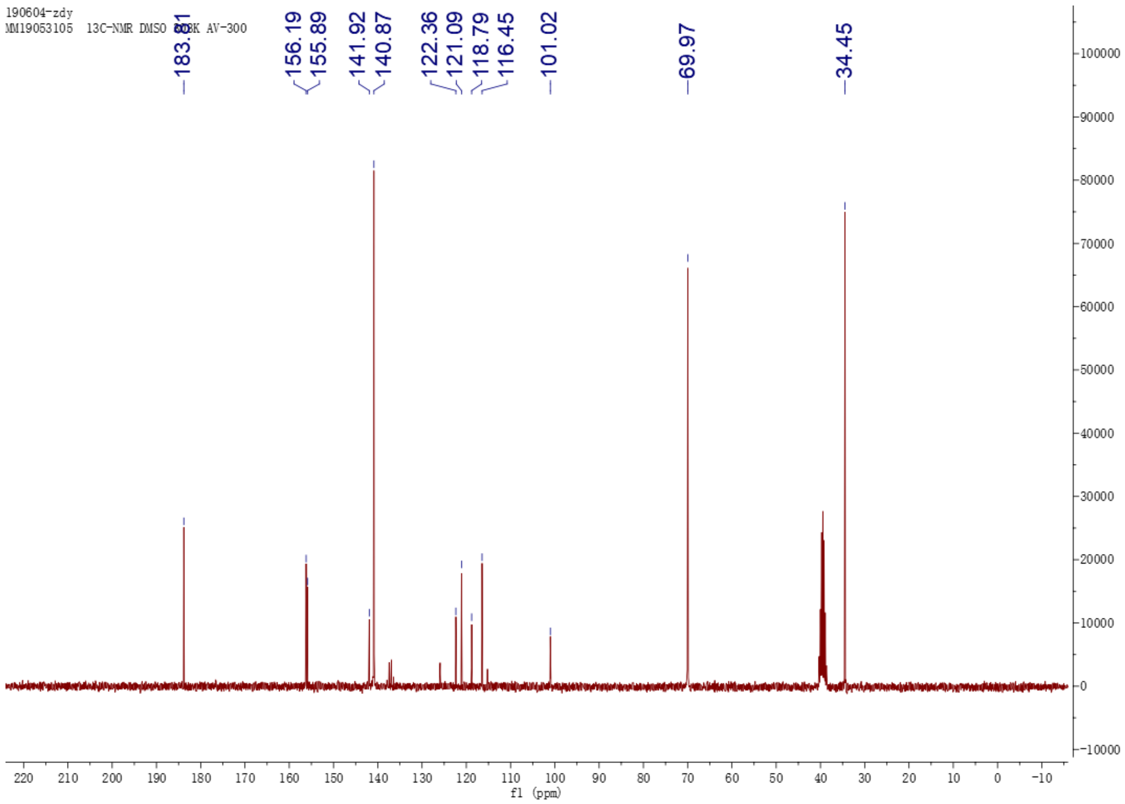

2-(2-((1-Bromonaphthalen-2-yl)oxy)acetyl)-3-hydroxy-5,5-dimethylcyclohex-2-en-1-one (III1):

Yield, 90%; white solid; ^{1}H NMR (300 MHz, $CDCl_3$) δ 16.30 (s, 1H), 8.28 (d, J = 8.6

Hz, 1H), 7.84 – 7.74 (m, 2H), 7.60 (t, J = 7.7 Hz, 1H), 7.43 (t, J = 7.4 Hz, 1H), 7.18 (d, J = 9.0 Hz, 1H), 5.55 (s, 2H), 2.49 (s, 4H), 1.13 (s, 6H). ^{13}C NMR (75 MHz, $CDCl_3$) δ 199.55, 195.47, 152.72, 133.21, 130.17, 128.80, 128.01, 127.75, 126.32, 124.62, 114.99, 111.34, 109.61, 73.24, 45.34, 31.12, 28.14, 8.32. HRMS m/z: calculated 403.04667 ($C_{20}H_{19}BrO_4$ + H)$^+$, found 403.04661 ($C_{20}H_{19}BrO_4$ + H)$^+$.

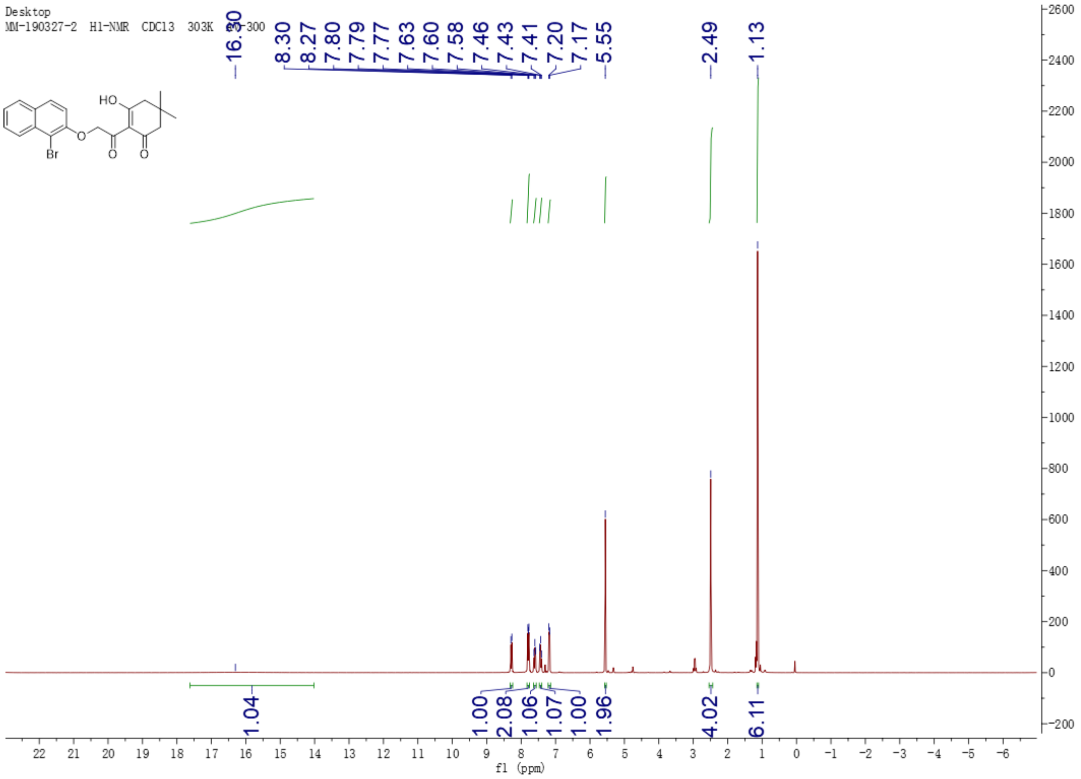

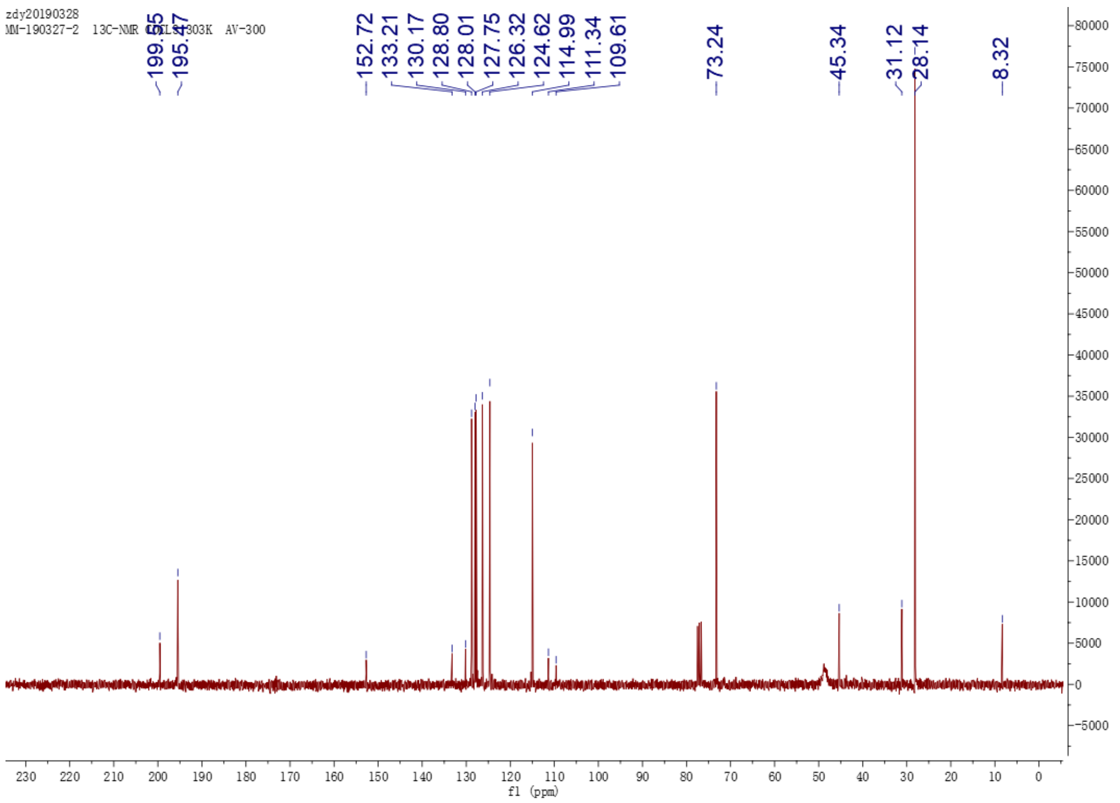

2-((1-Bromonaphthalen-2-yl)oxy)-1-(5-hydroxy-1,3-dimethyl-1*H*-pyrazol-4-yl)ethan-1-one (III2):

Yield, 85%; white solid; ^{1}H NMR (300 MHz, DMSO) δ 8.09 (d, J = 8.5 Hz, 1H), 7.89-7.86 (m, 2H), 7.61 (t, J = 7.4 Hz, 1H), 7.42 (t, J = 7.1 Hz, 1H), 7.23 (d, J = 9.0 Hz, 1H), 5.44 (s, 2H), 3.38 (s, 3H), 2.31 (s, 3H). ^{13}C NMR (75 MHz, DMSO) δ 188.19, 159.44, 153.00, 147.41, 132.31, 129.26, 128.98, 128.83, 128.15, 127.93, 125.10, 124.19, 114.93, 102.11, 71.82, 30.67, 13.44. HRMS m/z: calculated 375.02660 ($C_{17}H_{15}BrN_2O_3$ + H)$^+$, found 375.02641 ($C_{17}H_{15}BrN_2O_3$ + H)$^+$.

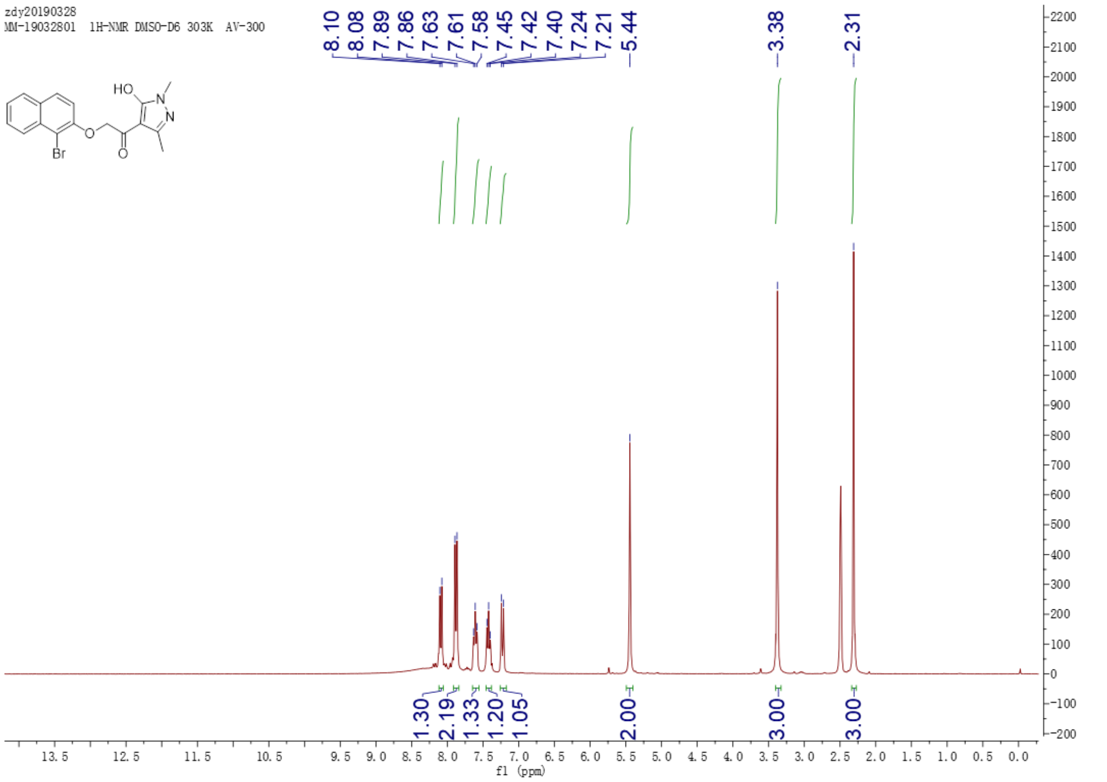

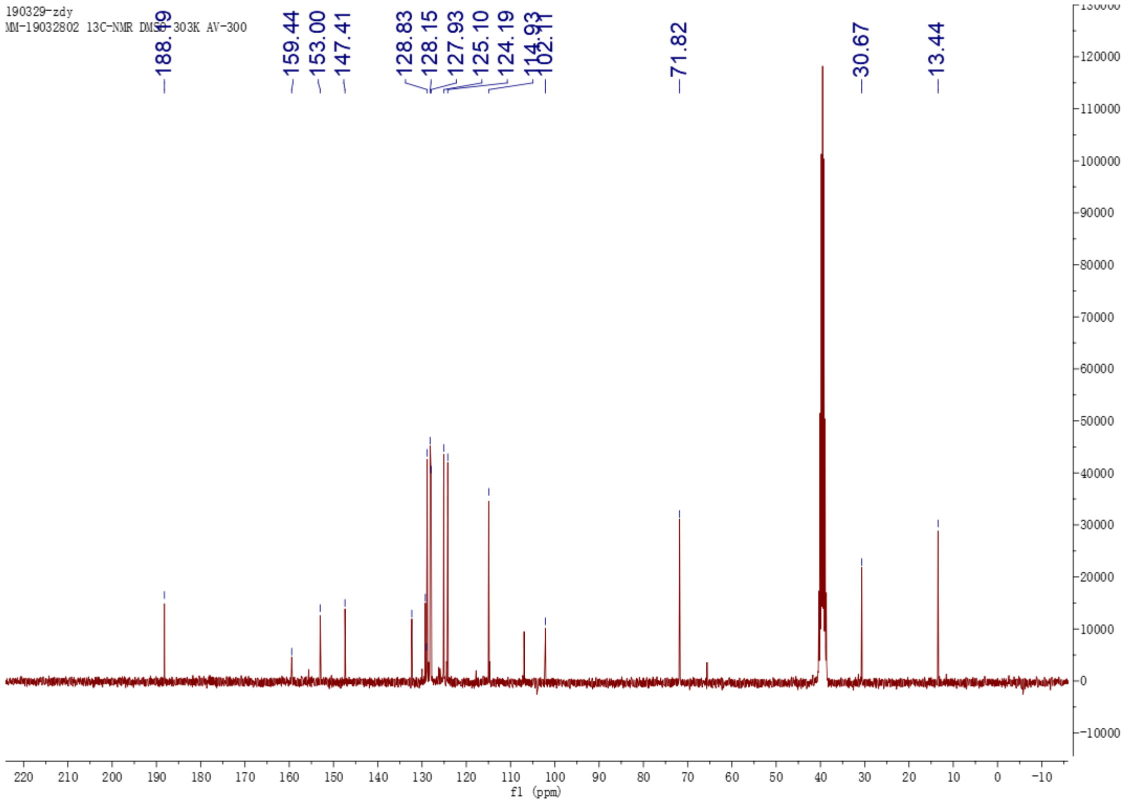

2-((1-Bromonaphthalen-2-yl)oxy)-1-(3-cyclopropyl-5-hydroxy-1-methyl-1*H*-pyrazol-4-yl)ethan-1-one (III3):

Yield, 71%; white solid; ^{1}H NMR (300 MHz, DMSO) δ 8.12 (d, J = 8.5 Hz, 1H),

7.91-7.71 (m, J = 7.4 Hz, 2H), 7.68 – 7.58 (m, 1H), 7.43 (t, J = 7.2 Hz, 1H), 7.28 (d, J = 9.1 Hz, 1H), 5.51 (s, 2H), 3.39 (s, 3H), 2.61-2.68 (m, 1H), 1.05 – 0.50 (m, 4H). ^{13}C NMR (75 MHz, DMSO) δ 188.69, 156.08, 153.53, 132.83, 129.76, 129.33, 128.64, 128.41, 125.61, 124.67, 115.44, 107.49, 103.21, 72.33, 66.12, 8.75, 7.40. HRMS m/z: calculated 401.04226 ($C_{19}H_{17}BrN_2O_3$ + H)$^+$, found 401.04234 ($C_{19}H_{17}BrN_2O_3$ + H)$^+$.

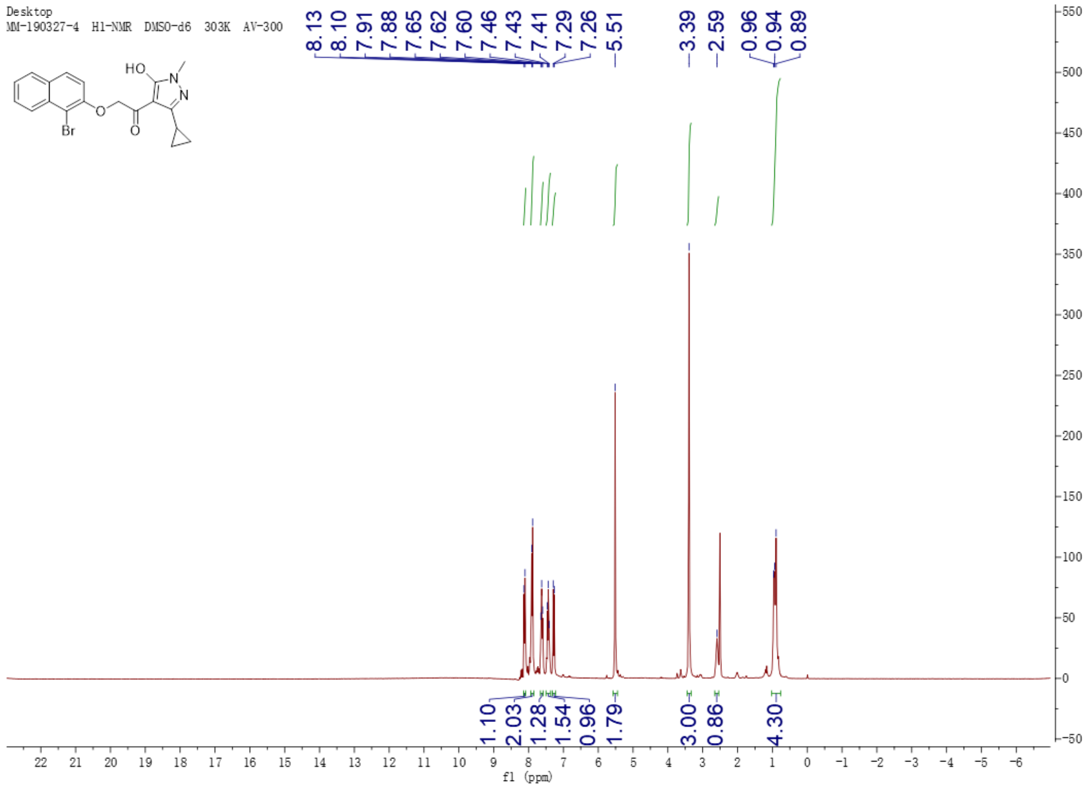

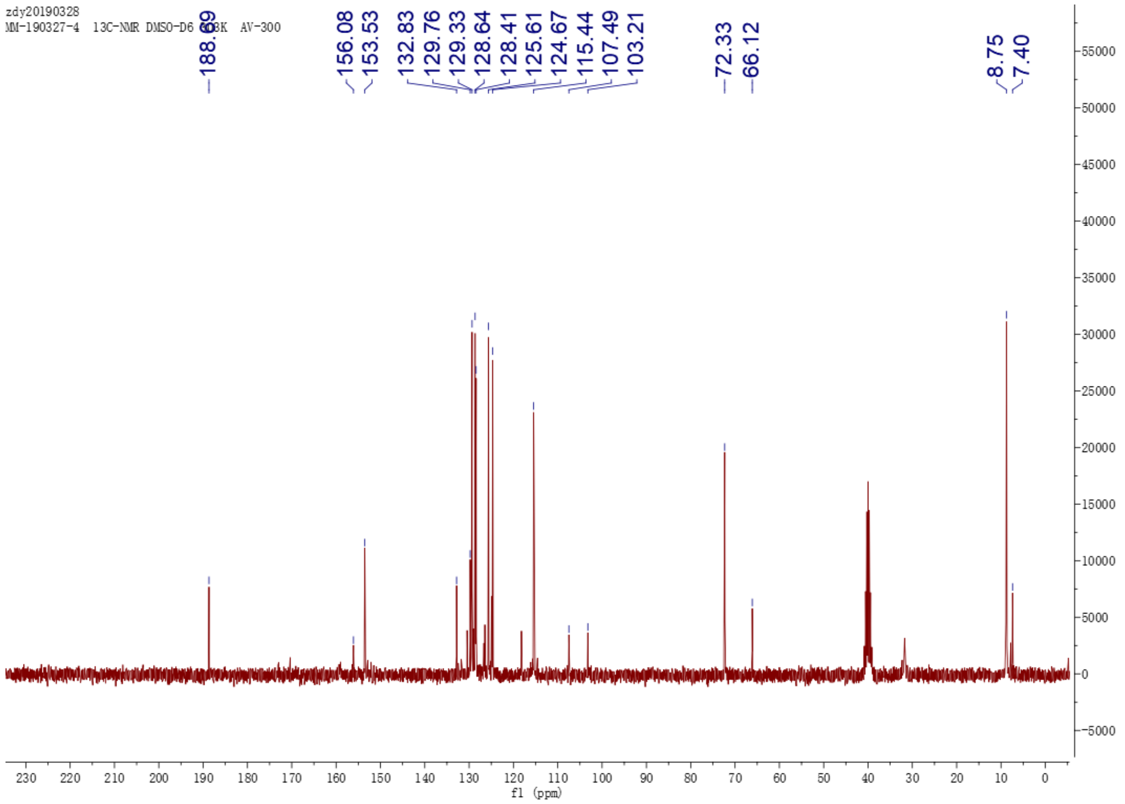

2-((1-Bromonaphthalen-2-yl)oxy)-1-(5-hydroxy-1-methyl-3-(trifluoromethyl)-1*H*-pyrazol-4-yl)ethan-1-one (III4):

Yield, 75%; white solid; ^{1}H NMR (300 MHz, $CDCl_3$) δ 9.88 (s, 1H), 8.28 (d, J = 8.6 Hz, 1H), 7.81-7.64 (m, 2H), 7.61 (t, J = 7.6 Hz, 1H), 7.45 (t, J = 7.4 Hz, 1H), 7.13 (d, J = 8.9 Hz, 1H), 5.34 (s, 2H), 3.75 (s, 3H). ^{13}C NMR (75 MHz, $CDCl_3$) δ 193.99, 162.27, 154.93, 135.81, 133.04, 131.82, 131.61, 130.67, 130.54, 129.04, 127.83, 127.58, 117.43, 112.66, 107.19, 73.95, 36.27. HRMS m/z: calculated 428.99834 ($C_{17}H_{12}BrF_3N_2O_3$ + H)$^+$, found 428.99548 ($C_{17}H_{12}BrF_3N_2O_3$ + H)$^+$.

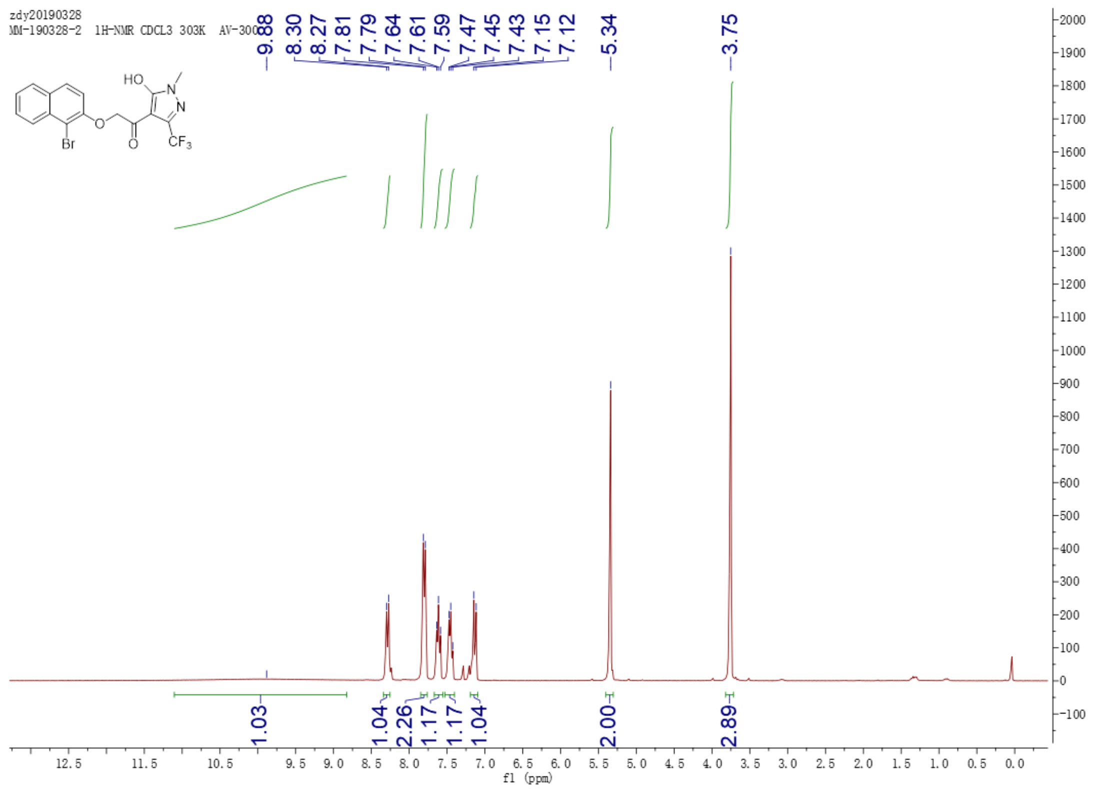

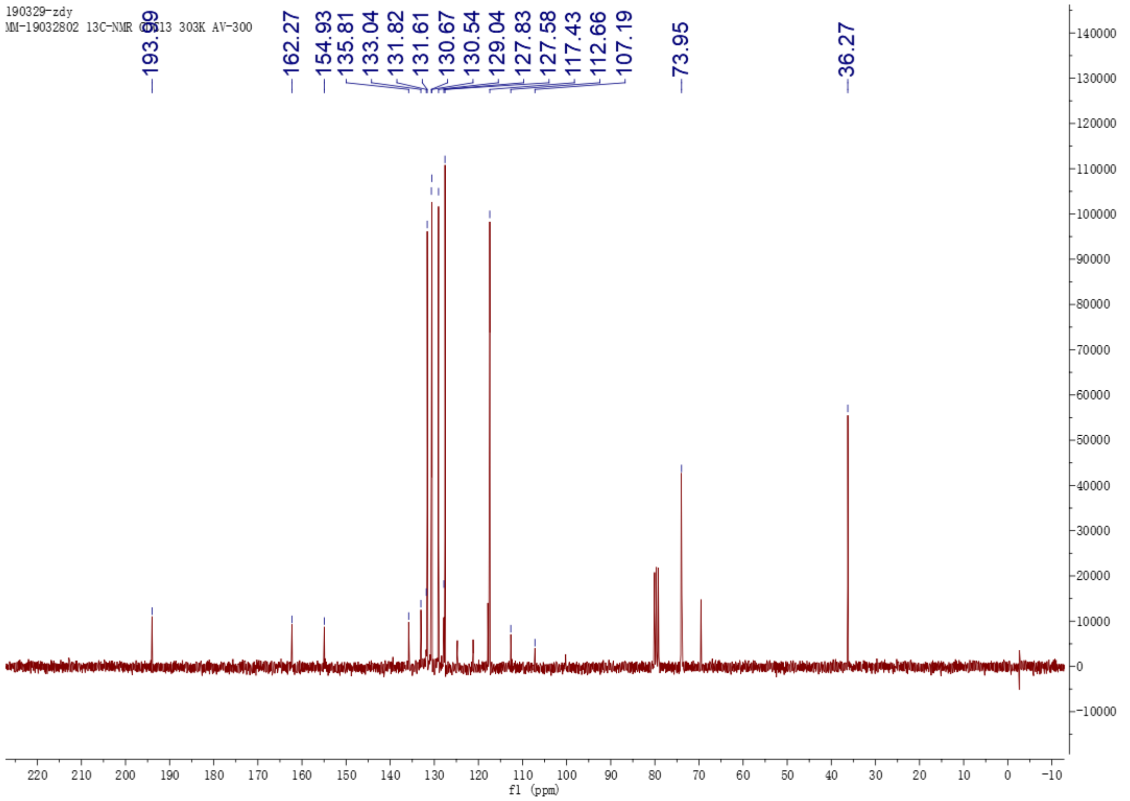

3-Hydroxy-2-(2-((2-nitropyridin-3-yl)oxy)acetyl)cyclohex-2-en-1-one (III5):

Yield, 82%; white solid; ^{1}H NMR (300 MHz, DMSO) δ 16.08 (s, 1H), 8.09 (d, J = 4.4 Hz, 1H), 7.81 (d, J = 8.5 Hz, 1H), 7.66 (dd, J = 8.4, 4.4 Hz, 1H), 5.53 (s, 2H), 2.59-

2.55 (m, 4H), 1.99 – 1.84 (m, 2H). ^{13}C NMR (75 MHz, DMSO) δ 189.29, 155.64, 150.72, 148.75, 142.15, 141.54, 131.48, 127.69, 104.97, 74.01, 33.51, 25.60, 10.86. HRMS m/z: calculated 293.06954 $(C_{13}H_{12}N_2O_6 + H)^+$, found 293.06865 $(C_{13}H_{12}N_2O_6 + H)^+$.

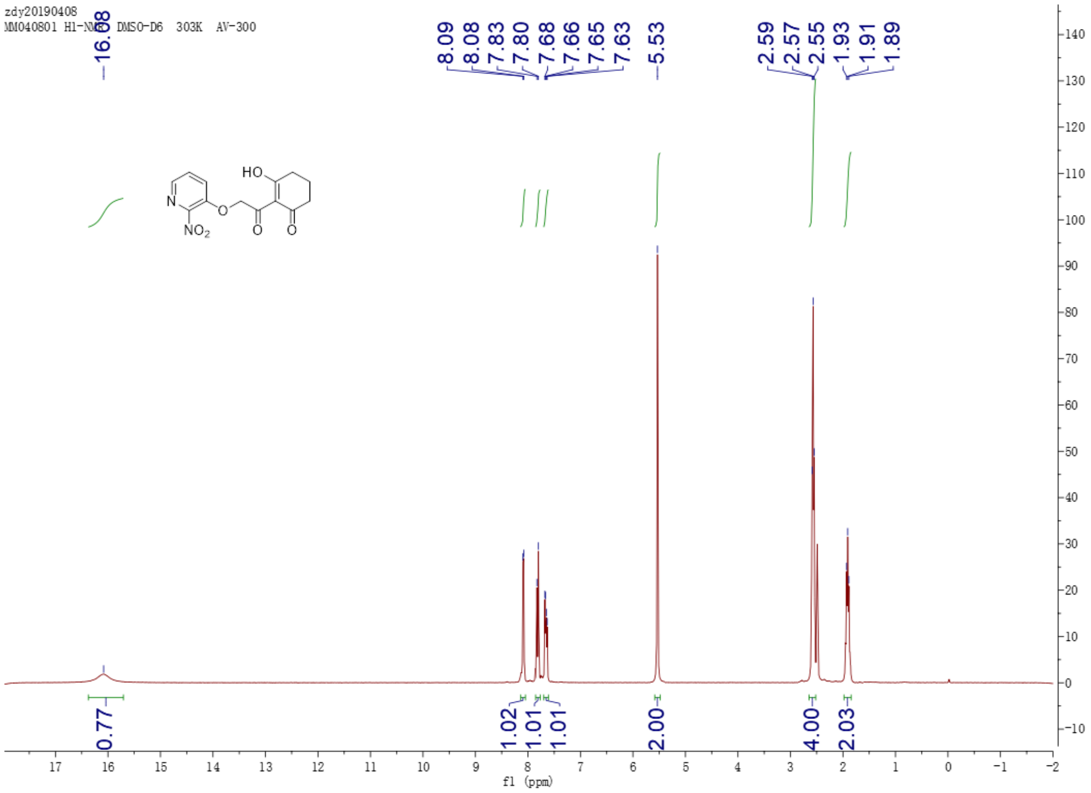

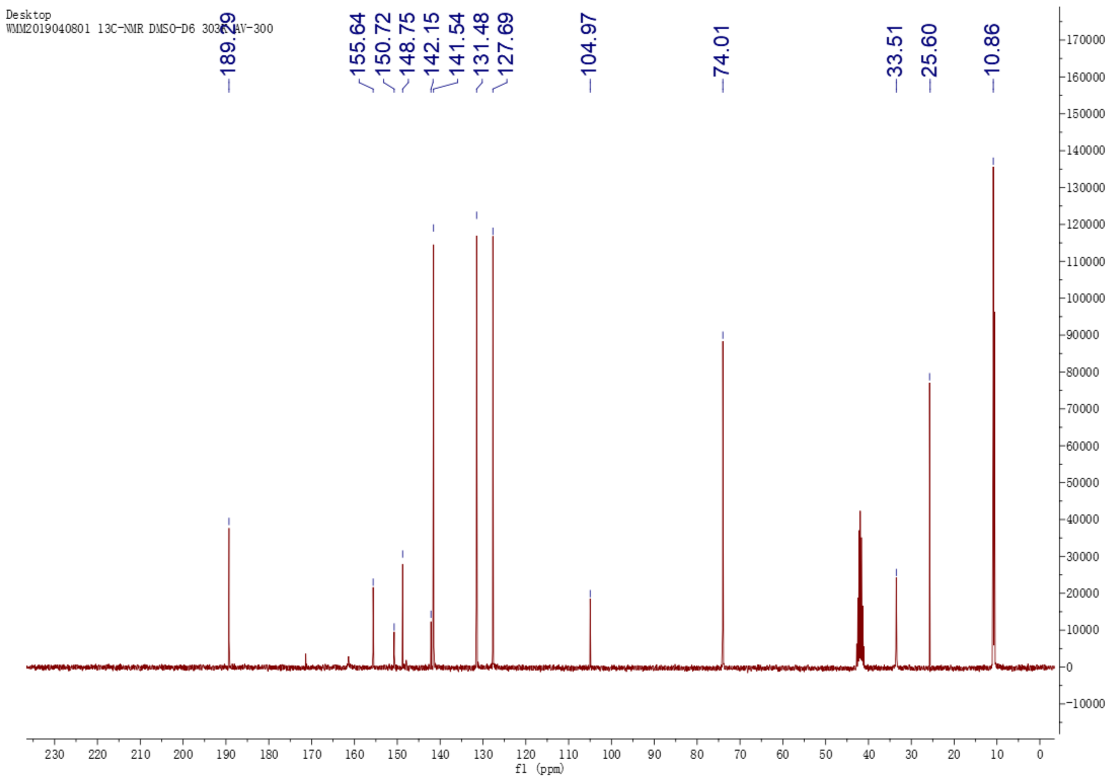

1-(3-Cyclopropyl-5-hydroxy-1-methyl-1*H*-pyrazol-4-yl)-2-((2-nitropyridin-3-yl)oxy)ethan-1-one (III6):

Yield, 84%; white solid; ^{1}H NMR (300 MHz, DMSO) δ 8.08 (d, J = 4.5 Hz, 1H), 7.75 (d, J = 8.5 Hz, 1H), 7.67 (dd, J = 8.5, 4.5 Hz, 1H), 5.49 (s, 2H), 3.35 (s, 3H), 2.57-2.54 (m, 5.3 Hz, 1H), 1.01 – 0.90 (m, 2H), 0.90 – 0.82 (m, 2H).^{13}C NMR (75 MHz, DMSO) δ 187.29, 153.64, 148.72, 146.75, 140.15, 139.54, 129.48, 125.69, 102.97, 72.01,65.76,31.51,8.86,8.62. HRMS (ESI): m/z for $C_{14}H_{14}N_{4}O_{5}Na^{+}$; calcd. 319.09642, found 319.09612.

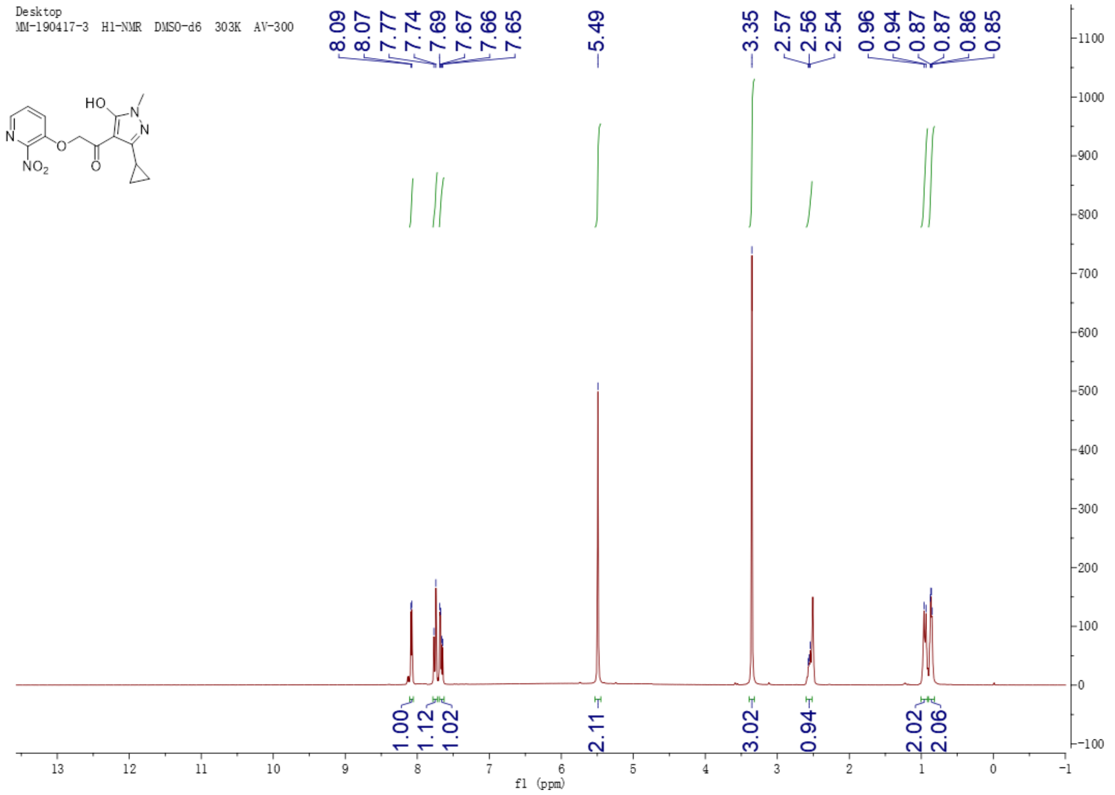
Desktop
MM-190417-3 H1-NMR DMSO-d6 303K AV-300
8.09
8.07
7.77
7.74
7.69
7.67
7.66
7.65
5.49
3.35
2.57
2.56
2.54
0.96
0.94
0.87
0.87
0.86
0.85
1.00
1.12
1.02
2.11
3.02
0.94
2.02
2.06
f1 (ppm)

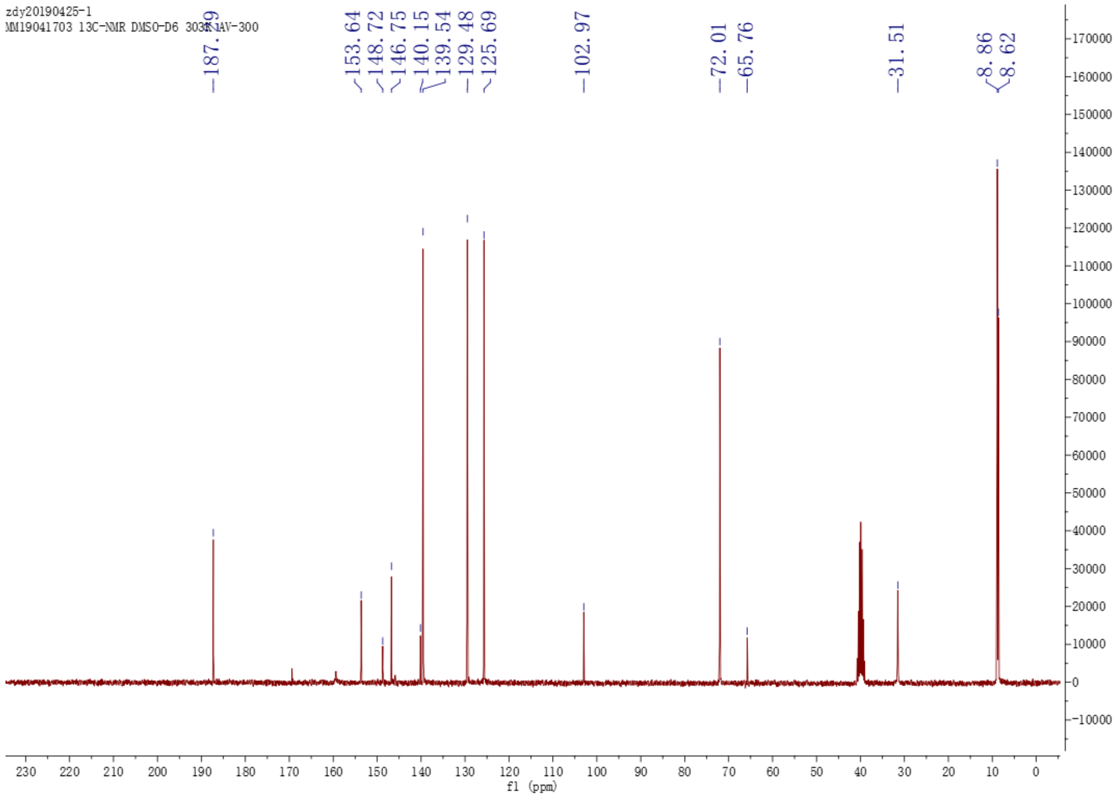
zdy20190425-1
MM19041703 13C-NMR DMSO-D6 303K AV-300
187.49
153.64
148.72
146.75
140.15
139.54
129.48
125.69
102.97
72.01
65.76
31.51
8.86
8.62
f1 (ppm)

References

1. Da-Wei Wang; Hong-Yan Lin; Bo He; Feng-Xu Wu; Tao Chen; Qiong Chen; Wen-Chao Yang; Guang-Fu Yang, J. Agric. Food Chem. 2016, 64, 8986−8993.
2. Antuganov, Dmitrii; Timofeev, Vasilii; Timofeeva, Ksenija; Antuganova, Yulija; Kondratenko, Yulia A., ChemistrySelect. 2018, 4, 12524-12527.
3. Chmil, V. D.; Sakodynskii, K. I., Zhurnal Analiticheskoi Khimii. 1984, 39, 1105-1111.
4. Larose, Richard H.; Chau, Alfred S. Y., ournal - Association of Official Analytical Chemists. 1973, 56, 1183-1187.
5. Cismesia, Megan A.; Ischay, Michael A.; Yoon, Tehshik P., Synthesis.2013,45, 2699-2705.
6. Li, Zhengqiu; Qian, Linghui; Li, Lin; Bernhammer, Jan C.; Huynh, Han Vinh; Lee, Jun-Seok; Yao, Shao Q., Angewandte Chemie, International Edition. 2016, 55, 2002-2006.
7. Ge, Yang; Wang, Changyuan; Song, Shijie; Huang, Jiaxin; Liu, Zhihao; Li, Yongming; Meng, Qiang; Zhang, Jianbin; Yao, Jihong; Liu, Kexin; Ma, Xiaodong; Sun, Xiuli. European Journal of Medicinal Chemistry. 2018, 143, 1847-1857.
8. Zhao, Dan; Huang, Shanshan; Qu, Menghua; Wang, Changyuan; Liu, Zhihao; Li, Zhen; Peng, Jinyong; Liu, Kexin; Li, Yanxia; Ma, Xiaodong; Shu, Xiaohong, European Journal of Medicinal Chemistry. 2017,126, 444-455.
9. Ding, Yili; Smith, Kenneth L.; Vara Prasad, Chamakura V. N. S.; Wang, Bingyun, Letters in Organic Chemistry. 2018, 15, 87-91.
10. Krasavin, Mikhail; Lukin, Alexei; Bakholdina, Anna; Zhurilo, Nikolay; Onopchenko, Oleksandra; Borysko, Petro; Zozulya, Sergey; Moore, Daniel; Tikhonova, Irina G., European Journal of Medicinal Chemistry. 2017, 140, 229-238.
11. Jimenez, Fabiola; Cruz, Maria del Carmen; Zuniga, Clara; Martinez, Maria A.; Chamorro, German; Diaz, Francisco; Tamariz, Joaquin, Medicinal Chemistry Research. 2010, 19, 33-57.
12. Mirk, Daniela; Waldvogel, Siegfried R., etrahedron Letters. 2004,42, 7911-7914.
13. He, H. W.; Yuan, J. L.; Peng, H.; Chen, T.; Shen, P.; Wan, S. Q.; Li, Y.; Tan, H. L.; He, Y. H.; He, J. B.; Li, Y, J. Agric. Food Chem. 2011, 59, 4801−4813.
14.Wang, W.; He, H. W.; Zuo, N.; He, H. F.; Peng, H.; Tan, X. S, J. Agric. Food Chem. 2012, 60, 7581−7587.
15. Tao, Qing-Qing; Liu, Li-Wei; Wang, Pei-Yi; Long, Qing-Su; Zhao, Yong-Liang; Jin, Lin-Hong; Xu, Wei-Ming; Chen, Yang; Li, Zhong; Yang, Song, Journal of Agricultural and Food Chemistry. 2019, 27, 7626-7639.
16. Timchalk, Charles; Campbell, James A.; Liu, Guodong; Lin, Yuehe; Kousba, Ahmed A., Toxicology and Applied Pharmacology. 2007, 219, 217-225.
17. He, H. W.; Peng, H.; Wang, T.; Wang, C.; Yuan, J. L.; Chen, T.; He, J.; Tan, X, J. Agric. Food Chem. 2013, 61, 2479−2488.
18. 4. Verma, A. K.; Rustagi, V.; Aggarwal, T.; Singh, A. P, J. Org. Chem. 2010, 75, 7691−7703.
19. 5. SuzuKi, M.; IwasaKi, H.; Fujikawa, Y.; Kitahara, M.; Sakashita, M.; Sakoda, R,

Bioorg. Med. Chem. 2001, 9, 2727− 2743.
20. Ferreira, Rafaela S.; Dessoy, Marco A.; Pauli, Ivani; Souza, Mariana L.; Krogh, Renata; Sales, Ana I. L.; Oliva, Glaucius; Dias, Luiz C.; Andricopulo, Adriano D., Journal of Medicinal Chemistry. 2014, 57, 2380-2392.
21. Lin-bo Luan; zi-jie Song; Zhi-ming Li; Quan-rui Wang, Beilstein J. Org. Chem. 2018, 14, 1826–1833.
22. Wang, Quanrui; Liu, Yuanfa; Wang, Keren，Huaxue Xuebao. 1988, 46, 394−397.
